# Heavier Alkyne‐Ni^0^ Complexes, [R_2_E_2_·Ni] (E = Sn, Pb), Exhibiting σ‐Complex Character

**DOI:** 10.1002/anie.6734116

**Published:** 2026-06-04

**Authors:** Leopold Junge, Emeric Schubert, Israel Fernández, Terrance J. Hadlington

**Affiliations:** ^1^ Fakultät für Chemie Technische Universität München Garching Germany; ^2^ Facultad de Ciencias Químicas I Centro de Innovación en Química Avanzada Universidad Complutense de Madrid Madrid Spain

**Keywords:** coordination complex, dehydrogenation, main group element, nickel, organometallic chemistry, transition metal, sigma‐complex, heavier alkyne complexes

## Abstract

Despite the ubiquitous nature of alkyne‐transition metal complexes in synthesis and catalysis, analogous examples for the heavier group 14 elements are extremely rare. Here, we describe access to the first such species for Sn and Pb in nickel(0) complexes, [(^Cy^LE)_2_·Ni] (**5** and **6**; ^Cy^L = [{Cy_2_PCH_2_Si(^i^Pr)_2_}(Dipp)N]^−^; Dipp = 2,6‐^i^Pr_2_C_6_H_3_). Although these systems cannot be accessed by direct addition of the E^I^ dimers to a Ni^0^ synthon (i.e. Ni(cod)_2_; cod = 1,5‐cyclooctadiene), we report a novel reductive group elimination pathway, forming the E^I^ dimers from E^II^ synthons in the coordination sphere of Ni, for example, through H_2_ elimination from Sn^II^ hydride compounds. Structural analyses in combination with computational studies indicate that these species are best described as σ‐complexes of E^I^ dimers with a transition metal centre, representing a new bonding mode in organometallic coordination chemistry.

## Graphical abstract

Using a reductive group elimination strategy, heavier alkyne (LEEL; E = Sn, Pb) complexes of Ni^0^ are accessed. In contrast to classical alkyne coordination, these systems are best described as σ‐complexes, driven by ligand‐induced constraints and the established poor π‐bonding in heavier alkyne analogues. This introduces a new class of bonding for group 14 coordination compounds.

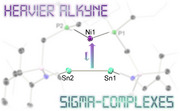

## 1 Introduction

**FIGURE 1:**
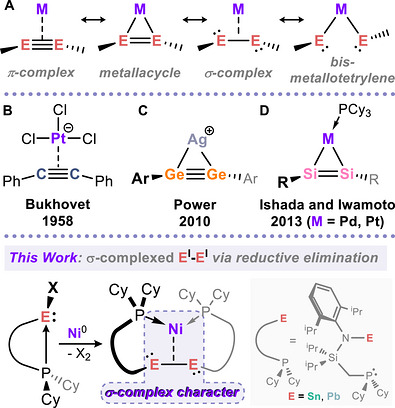
Possible resonance forms for η^2^‐(heavier)alkyne TM complexes (A); the first reported example of an alkyne‐TM complex (B); reported examples of heavier alkyne complexes (C and D); and this work, which reports examples of η^2^‐distannyne and ‐diplumbyne complexes of Ni^0^ featuring formal (R_2_E_2_)–Ni σ‐complexation. In C, Ar = 2,6‐(Dip)_2_C_6_H_3_ and Dip = 2,6‐^i^Pr_2_C_6_H_3_. In D, R = [(Me_3_Si)_2_(^t^BuH_2_C)C]^−^.

Alkene and alkyne complexes of the transition metals (TMs) represent the earliest class of organometallic compounds, now ubiquitous in catalytic transformations of those organic molecules [1]. The first such example, Zeise's salt ([Cl_3_Pt·C_2_H_4_]K], was first synthesized in 1831 [2], but was only structurally verified some 140 years later, in 1969 [3]—this established our modern concepts of alkene complexation, rooted in the Dewar–Chatt–Duncanson model [4, 5] This discovery is pivotal in our understanding of TM mediated chemistry [6], in alkene metathesis [7, 8] alkyne metathesis [9, 10], and broader functionalisation catalyses, for example, hydrogenation [11, 12] and hydroformylation [13, 14, 15] Naturally, the discovery of these all‐carbon π‐complexes has led to the exploration of heavier group 14 derivatives [16]. This is somewhat more challenging, given that E─E multiple bonding (E = Si‐Pb) is significantly less favourable than C─C bonds [17, 18]. Formally low‐valent E─E bonded systems for those heavier elements were unknown until relatively recently, with the first heavier alkene analogue reported in the 1980s [19] and an alkyne analogue in 2000 [20]. Research regarding π‐complexes has focused on heavier alkene derivatives, akin to Zieses's Pt‐alkene complex, with a swathe of Si‐based systems now known [21, 22, 23, 24, 25, 26, 27, 28] and a significantly smaller number of Ge─Ge and Sn─Sn bonded systems [25, 29, 30, 31]. This broadly results in a bonding continuum, moving from formal bis‐(*η* ^1^‐tetrylene)‐TM complexes to formal *η* ^2^‐ditetrene‐TM π‐complexes, via metallacyclopropane derivatives [16]. Moving to heavier alkyne complexes, an analogous series can also be defined (Figure 1A), moving from a formally triply‐bonded π‐complex to a bis‐(metallotetrylene). Here, the doubly E═E bonded metallacycle and the singly E─E bonded σ‐complex would be the expected intermediate tautomers. However, in this chemical space significantly fewer species are known beyond carbon (e.g., Figure 1B)—a single example of a digermyne complex of the Ag^+^ ion has been reported by the group of Power (Figure 1C) [32], whilst Ishida, Iwamoto and co‐workers reported base‐free disilyne complexes of Pd and Pt (Figure 1D) [33]. Examples featuring Sn─Sn and Pb─Pb moieties are presently unknown. This suggests that synthetic protocols for accessing such systems are distinctly lacking, limiting our knowledge of their stability, reactivity, and electronic structure.

In this work, we describe our efforts towards the synthesis and characterization of distannyne and diplumbyne complexes of Ni^0^. This relies upon the utility of a chelating ligand framework, lending a greater entropic stability to the target complexes, an effective strategy in earlier reported disilane‐Cu σ‐complexation [34]. Though the direct addition of the E^I^ dimers to Ni^0^ synthons does not lead to the desired complexes, we devise a reductive route allowing for group elimination from E^II^ synthons, yielding the target species. Mechanistic insights are provided through density functional theory (DFT) calculations, demonstrating that Ni behaves as a remote redox mediator in, for example, H_2_ elimination. Inspection of structural and computational data suggests that the formed heavier alkyne complexes are best described as η^2^ σ‐complexes of the E^I^─E^I^ single bonds with Ni^0^, marking a new entry in the coordination behaviour of the group 14 elements.

## 2 Results and Discussion

**SCHEME 1:**
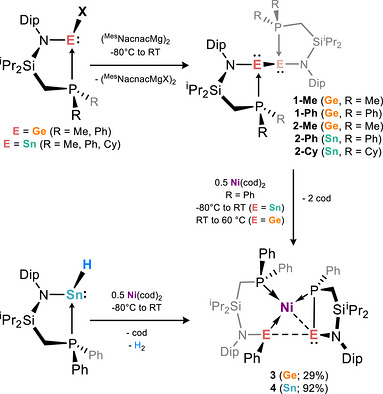
Synthesis of E^I^‐dimers (E = Ge, Sn), their reactions with Ni^0^ to form ligand‐activated complexes **3** and **4**, and the convergent formation of **4** via Ni‐mediated H_2_‐elimination from ^Ph^L(H)Sn:.

**FIGURE 2:**
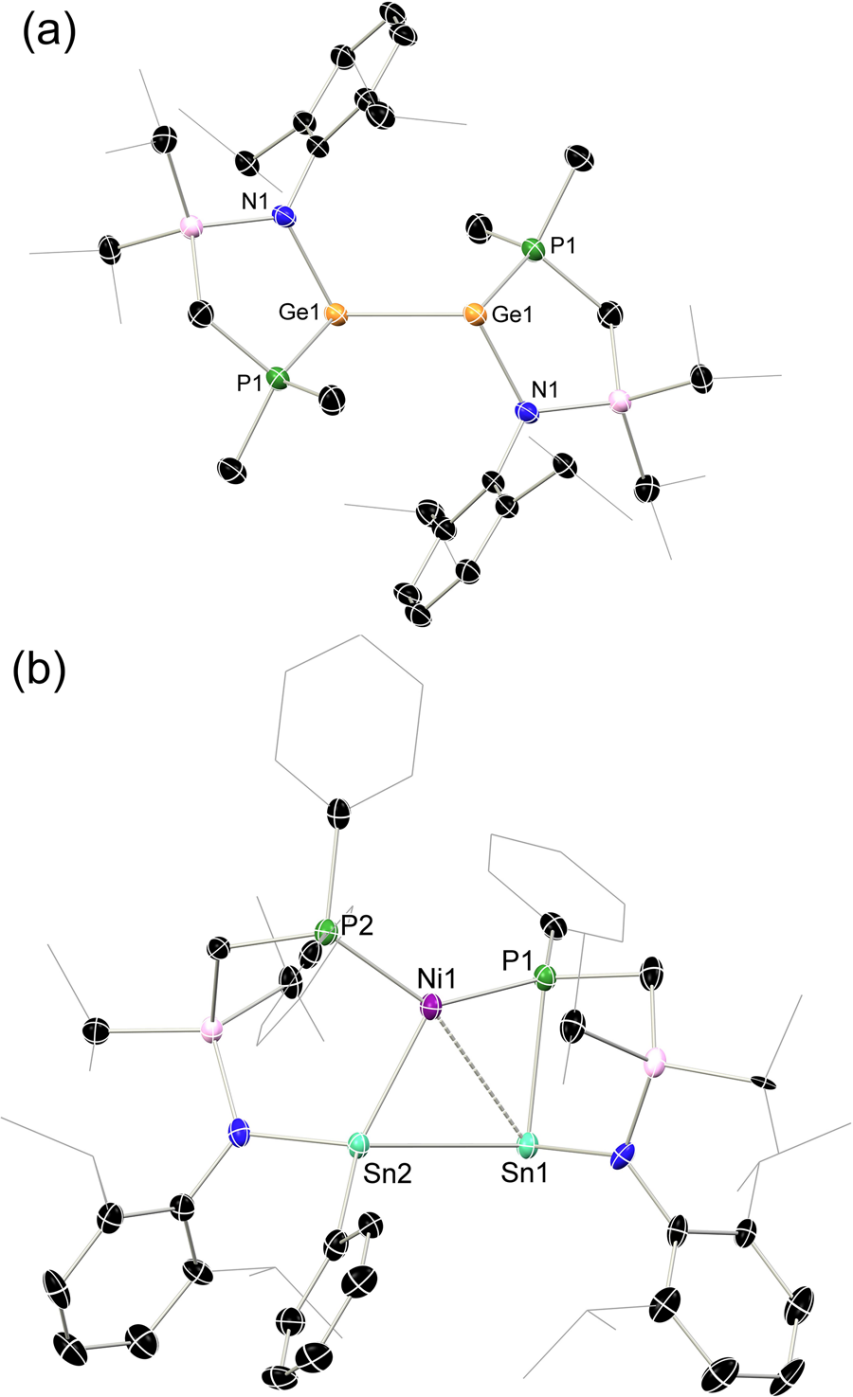
Molecular structures of (a) **1‐Me** and (b) **4**, with thermal ellipsoids at 30% probability, and hydrogen atoms removed for clarity. Selected bond lengths (Å) and angles (°) for **1‐Me**: Ge1‐Ge1’ 2.6792(9); P1‐Ge1 2.432(1); N1‐Ge1 2.033(3); N1‐Ge1‐Ge1’ 103.96(9); P1‐Ge1‐Ge1’ 90.83(3); N1‐Ge1‐P1 85.98(9). For **4**: Sn1‐Sn2 3.035(1); Sn1‐Ni1 2.983(1); Sn2‐Ni1 2.421(1); P1‐Ni1 2.158(2); P2‐Ni1 2.145(3); Sn1‐P1 2.620(2); P1‐Ni1‐P2 135.21(9); P1‐Ni1‐Sn2 125.79(7); P2‐Ni1‐Sn2 96.27(6) P1‐Ni1‐Sn1 58.70(6); Sn1‐Ni1‐Sn2 67.41(3).

We began our study by seeking a family of dimeric E^I^ species (^R^LE)_2_ (E = Ge, Sn, Pb; ^R^L = [{R_2_PCH_2_Si(^i^Pr)_2_}(Dipp)N]^−^; Dipp = 2,6‐^i^Pr_2_C_6_H_3_; R = Me, Ph, Cy), stabilised by our chelating amidophosphine ligand scaffold (i.e., ^R^L, Scheme 1). To this end, and akin to a number of reported E^I^ dimers [35, 36, 37, 38, 39, 40], the halo‐tetrylenes, ^R^L(X)E: (X = Cl, Br), were reduced with 0.5 equiv. of the Jones Mg^I^ dimer (^Mes^NacnacMg)_2_. Mixed results were observed here: no stable reduced species could be observed for Pb, and the steric bulk of the ^Cy^L ligand apparently prevents reduction of the ^Cy^L(Cl)Ge: synthon. Nevertheless, the target dimers could be observed for all Sn systems, and for ^Me^L‐ and ^Ph^L‐ligated Ge systems, demonstrated by the formation of a single new species in the corresponding ^31^P{^1^H} NMR spectra of reaction mixtures. All systems are purified by recrystallisation, allowing for structural characterisation of (^R^LGe)_2_ (R = Me, **1‐Me**; R = Ph, **1‐Ph**) and (^R^LSn)_2_ (R = Me, **2‐Me**; R = Ph, **2‐Ph**; R = Cy, **2‐Cy**) as deep orange (Ge) or deep green–orange (Sn) crystals (e.g., Figure 2a), which were isolated in yields ranging from 16% (**2‐Me**) to 91% (**2‐Cy**). This largely reflects the effects of sterics on stabilisation: the ^Me^L ligand lends little steric protection at the P‐arm, rendering its reduced complexes extremely air and moisture sensitive (i.e., decolouration of crystals under perfluorinated oil in minutes). In contrast, although **2‐Cy** is air‐ and moisture‐sensitive, the green colour of these crystals is retained for some hours under perfluorinated oil. Observed E─E bond distances in all systems align with formally singly bonded derivatives reported in the literature for both Ge (i.e., d_Ge‐Ge_ in **1‐Me**: 2.681 Å; in **1‐Ph**: 2.6402(9) Å) [41, 42] and Sn (d_Sn‐Sn_ = in **2‐Me**: 3.017(4) Å; in **2‐Ph**: 3.0556(8) Å; in **2‐Cy**: 3.085(1) Å) [36, 42, 43, 44]. The ^31^P NMR spectra for the latter species are indicative of their dimeric nature, with **2‐Ph** showing both ^1^ and ^2^ *J*_SnP_ coupling (^1^ *J*_SnP_ = 712 Hz; ^2^ *J*_SnP_ = 70 Hz), expected to be absent upon metal coordination. All complexes are strongly *trans*‐bent (*e.g*., ∠_NGeGe’_ in **1‐Me**: 103.96(9)°), or pyramidalised at the E‐centres (*e.g*., Σ of ∠@Ge in **1‐Me**: 280.77°), indicative of the presence of lone electron pairs at these centres (Figures S110–S112 in ESI).

Following the isolation of the above dimers, reaction with Ni(cod)_2_ aimed to generate the targeted metal complexes, driven by chelation. The first systems tested here utilised the least bulky ^Me^L ligand, which we expected to have the lowest barrier to complexation, on steric grounds. Both Ge‐ and Sn‐centred mixtures led to complex ^1^H and ^31^P NMR spectra, and no meaningful species could be isolated from crude reaction mixtures. The bulkier ^Cy^L ligand thwarts reactivity, whereby no new species are formed on reaction of **2‐Cy** with Ni(cod)_2_. A single new species is formed, however, on reactions involving both **1‐Ph** and **2‐Ph**, leading to the appearance of two new doublet signals in the crude ^31^P{^1^H} NMR spectra (Figures S73 and S83 in ESI). Reactivity with the former Ge^I^ species requires heating at 60°C for 12 h, whilst the Sn congener proceeds rapidly at ambient temperature. Still, the described NMR observations do not align with the expected symmetrical dimer coordination—the root of this observation is borne out by the X‐ray crystal structures of the formed species (Scheme 1): here we find that complexation of nickel does indeed occur, but is followed by ligand P─C activation, with migration of one P–*Ph* group to an adjacent E centre in the formation of **3** (Ge; Figure S113) and **4** (Sn; Figure 2b). Related ^Ph^C‐P scission has been observed by us previously, in Co^0^ and Fe^‐I^ complexes featuring the ^Ph^L ligand [45, 46]. We describe these complexes as Ni° coordination compounds, featuring one phenyl tetrylene (e.g., ^Ph^L(Ph)E:) [47, 48] and one formally P–E bonded phosphido‐tetrylene as reported in our earlier Co chemistry [46]; overall, complexes **3** and **4** are formed through oxidative scission of a C–P bond, and concomitant formation of new E–Ph and E–P bonds. This described bonding mode is borne out by long E–E distances (e.g., in **3**: 2.805(1); in **4**: 3.035(1) Å), and short E1–P1 distances (e.g., d_Ge1‐P1_ in **3**: 2.397(1) Å; d_Sn1‐P1_ = 2.620(2) Å) when compared to those in E^I^ dimers described above (e.g., in **1‐Ph**: 2.647(1) Å; in **2‐Ph**: 2.763(2) Å). Redissolved crystals of **3** and **4** yield NMR spectra in‐keeping with crude reaction mixtures, with complex ^1^H NMR spectra (Figures S70 and S80 in ESI) due to the now two unsymmetrical ligand environments, and two doublets in each ^31^P{^1^H} NMR spectrum (**3**: ^2^ *J*_PP_ = 24.4 Hz; **4**: ^2^ *J*_PP_ = 49.9 Hz). Satisfyingly, the ^119^Sn{^1^H} NMR of **4** also displays two doublets (Sn1: δ = ‐778 ppm, ^1^J_SnP_ = 1099 Hz; Sn2: δ = 792, ^2^J_SnP_ = 769 Hz).

**SCHEME 2:**
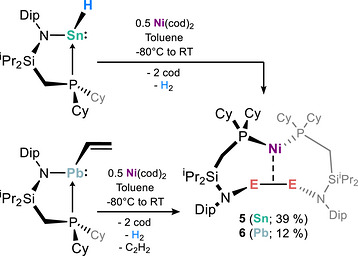
Synthesis of *η* ^2^‐(LEEL)·Ni complexes, **5** (Sn) and **6** (Pb), through Ni‐mediated group elimination from E^II^ synthons.

Driven by the observed complex formation and C–P bond scission in the reaction between dimers **1** and **2** with Ni^0^, we sought an alternative pathway towards targeted complexation, specifically through reductive group elimination from E^II^ species. This also opens the opportunity to access lead(I) complexation, where the free diplumbyne derivatives could not be synthesised. Promisingly, we find that the addition of two equiv. of the tin(II) hydride ^Ph^L(H)Sn: to Ni(cod)_2_ leads to dihydrogen elimination in the formation of the above‐described ligand activation product **4**. We were very pleased to observe that extending this protocol to bis(alkyl)phosphine‐substituted ^Cy^L(H)Sn: also leads to H_2_ elimination, now forming a stable derivative of the targeted Ni^0^ *η* ^2^–complex of a Sn^I^ dimer, in complex **5** (Scheme 2). We note here that well‐defined H_2_ reduction elimination from stannanes (i.e., Sn^IV^ hydrides) to afford stannylenes (i.e., Sn^II^ hydrides) is known [49]; the further elimination of H_2_ to form well‐defined Sn^I^ dimers is as yet unknown. The group of Power, however, has shown that spontaneous H_2_ elimination does occur in terphenyl‐substituted Sn^II^ hydride dimers, albeit to yield over‐reduced Sn clusters [50]. Compound **5** represents the first example of a Sn^I^Sn^I^ bond coordinated to any TM, thus expanding this class of species beyond C, Si, and Ge. We thus sought a synthetic pathway to gain access to the Pb derivative, so as to complete this compound class for the full series of group 14 elements. Our initial efforts in this direction sought the Pb^II^ hydride congener of ^Cy^L(H)Sn; the ^Ph^L ligand was avoided given the observed ligand activation in Ge and Sn complexes. This species, though transiently observed in the ^1^H NMR spectra of reactions between ^Cy^LPbBr and K[^s^Bu_3_BH] (i.e., a Pb‐*H* signal is observed, *δ* = 38.58 ppm), rapidly decomposes to yield dihydrogen, a protonated ligand, and metallic lead. We have recently demonstrated the utility of ethenyl‐group elimination from ethenyl plumbylenes as a pathway to Pb^II^ cations [51] through hydride abstraction and hypothesised a similar spontaneous pathway may be feasible in combination with Ni^0^. To this end, we find that 2 equiv. of ^Cy^L(C_2_H_3_)Pb: react with Ni(cod)_2_ to form the targeted chelating diplumbyne complex, **6**, albeit in poor yield. We attribute this to the concomitant formation of the protonated ligand and metallic lead, perhaps through the transient formation of the unstable hydride, ^Cy^L(H)Pb:.

**FIGURE 3:**
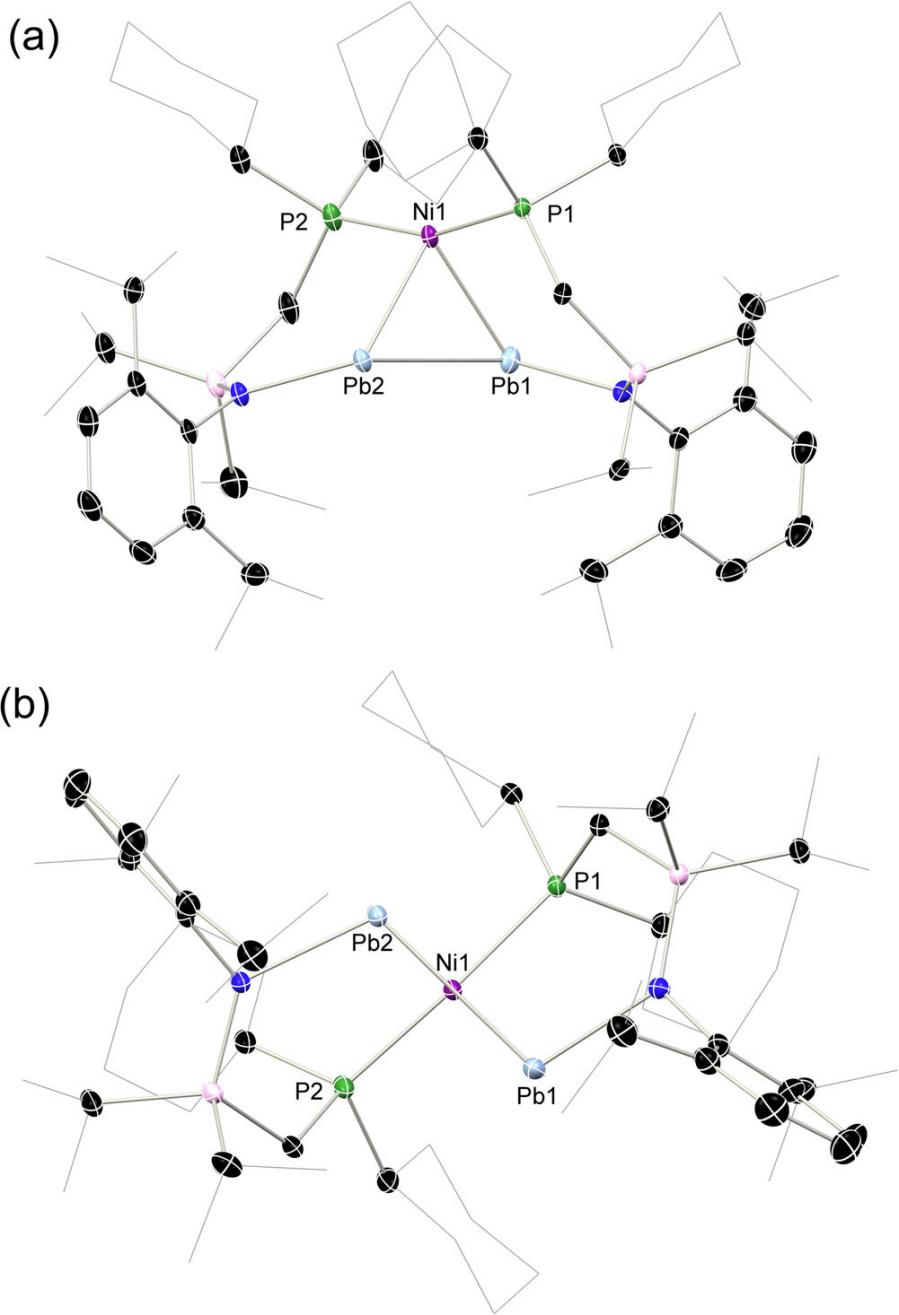
The molecular structure of **6**, viewed (a) perpendicular and (b) parallel with the [NiPb_2_] plane, with thermal ellipsoids at 30% probability and hydrogen atoms removed for clarity. Selected bond lengths (Å) and angles (°) for **6**: Pb1‐Pb2 3.262(1); Pb1‐Ni1 2.614(1); Pb2‐Ni1 2.585(1); P1‐Ni1 2.203(1); P2‐Ni1 2.209(1); Pb1‐Ni1‐Pb2 77.71(2); P1‐Ni1‐P2 159‐77(5); N1‐Pb1‐Pb2 102.06(9); N2‐Pb2‐Pb1 109.76(9).

The isolation and structural characterisation of **5** (Figure S114 in ESI) and **6** (Figure 3), as the sole examples of side‐on complexed distannyne and diplumbyne TM complexes, respectively, warrants discussion of their bonding and electronic nature. The 4‐coordinate Ni centre in both systems lies between tetrahedral and see‐saw geometries, being closer to the latter for lead (τ_4_’ for **5**: 0.67; for **6**: 0.52) [52]; the P centres sit *trans* to each other, with large obtuse PNiP angles and acute ENiE angles (in **5**: ∠_P1NiP2_ = 140.55(3)°, ∠_Sn1NiSn2_ = 82.92(3)°; in **6**: ∠_P1NiP2_ = 159.77(5)°), ∠_Pb1NiPb2_ = 77.71(2)°). These two trajectories lie perpendicular to each other, as apparent when viewing the molecular structure through the [E_2_Ni] plane (Figure 3b for **6**). An idealised see‐saw geometry would favour Ni → [E_2_] dative bonding, with a near linear [PNiP] motif [53, 54, 55]. Sn–Ni bond lengths in **5** are slightly longer than typical dative Sn → Ni interactions (i.e., 2.4557(7) and 2.481(1) Å; mean reported value: 2.43 Å), whilst no Pb–Ni bonded species have previously been crystallographically characterised beyond interstitial cluster compounds [56, 57]. Both **5** and **6** feature long E‐E bonds (*d*_Sn‐Sn_ in **5**: 3.2686(7) Å; *d*_Pb‐Pb_ in **6**: 3.262(1) Å); the former is slightly extended relative to the longest reported Sn^I^–Sn^I^ bond found in an (LSn)_2_ species of 3.1434(5) Å [36]. The latter is at the long end of known Pb^I^–Pb^I^ distances, for example, in a diaryldiplumbyne (d_PbPb_ = 3.2439(9) Å) [40], and is surprisingly shorter than its Sn counterpart. Both systems, then, are indicative of single bonds. Observed L‐Sn‐Sn’ (∠_N1Sn1Sn2_ = 106.65(5)°; ∠_N2Sn2Sn1_ = 105.34(5)°) and L–Pb–Pb’ (∠_N1Sn1Sn2_ = ∠_N2Sn2Sn1_) angles are in keeping with those observed in reported singly‐bonded distannynes and diplumbynes, whilst L–E–E–L torsion, or *cis‐*bending away from Ni, is minimal in both complexes (in **5**: 143.54(8)°; in **6**: 152.2(1)°). These latter values are in fact closer to an ideal planar geometry than the reported disilyne‐Pd and Pt complexes [33]; we assume this is due to a combination of significant lone electron pair character at both Sn and Pb centres in **5** and **6**, and geometric constraint brought about through P‐chelation. Importantly, these metrical parameters would suggest that complexes **5** and **6** are best described as σ‐complexes of RE–ER species with Ni^0^, representing a previously unobserved tautomer for TM complexes of heavier alkyne derivatives.

**FIGURE 4:**
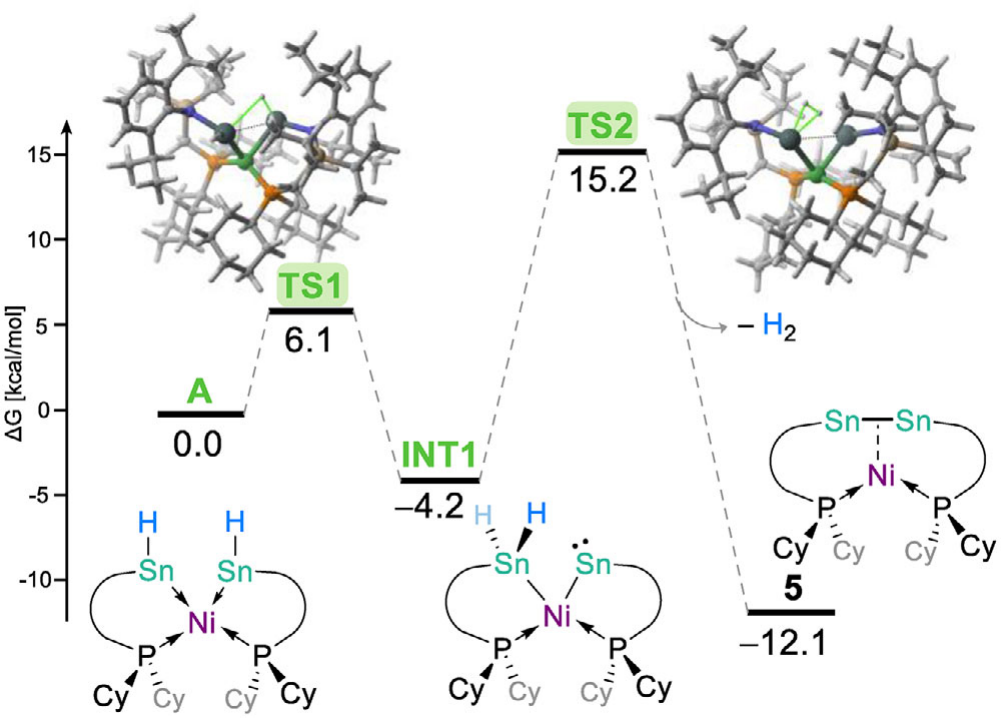
Computed reaction profile for the dihydrogen elimination reaction leading to **5**. Relative free energies (Δ*G*) were computed at the RI‐BP86‐B3BJ/def2‐SVP level.

Given the unique nature of the H_2_ elimination reaction which leads to complex **5**, the mechanism for this process was explored using DFT calculations at the dispersion‐corrected RI‐BP86‐D3BJ/def2‐SVP level (Figure 4). The transformation begins from the bis(hydridostannylene)nickel(0) complex **A**, which is transformed into intermediate **INT1** in an exergonic step (Δ*G* = −4.2 kcal·mol^−1^).

This proceeds via the Sn‐(μ‐H)‐Sn bridging hydride transition state **TS1** (barrier of 6.1 kcal mol^−1^), a saddle point associated with the hydride migration from one tin atom to the other. From this intermediate, a readily accessible dihydrogen elimination step then proceeds via **TS2** (barrier of 19.4 kcal mol^−1^), leading to the formation of **5** with an overall exergonicity of −12.1 kcal mol^−1^. This mechanism, then, does not directly chemically involve the Ni centre, which rather behaves as a redox mediator in this process.

**FIGURE 5:**
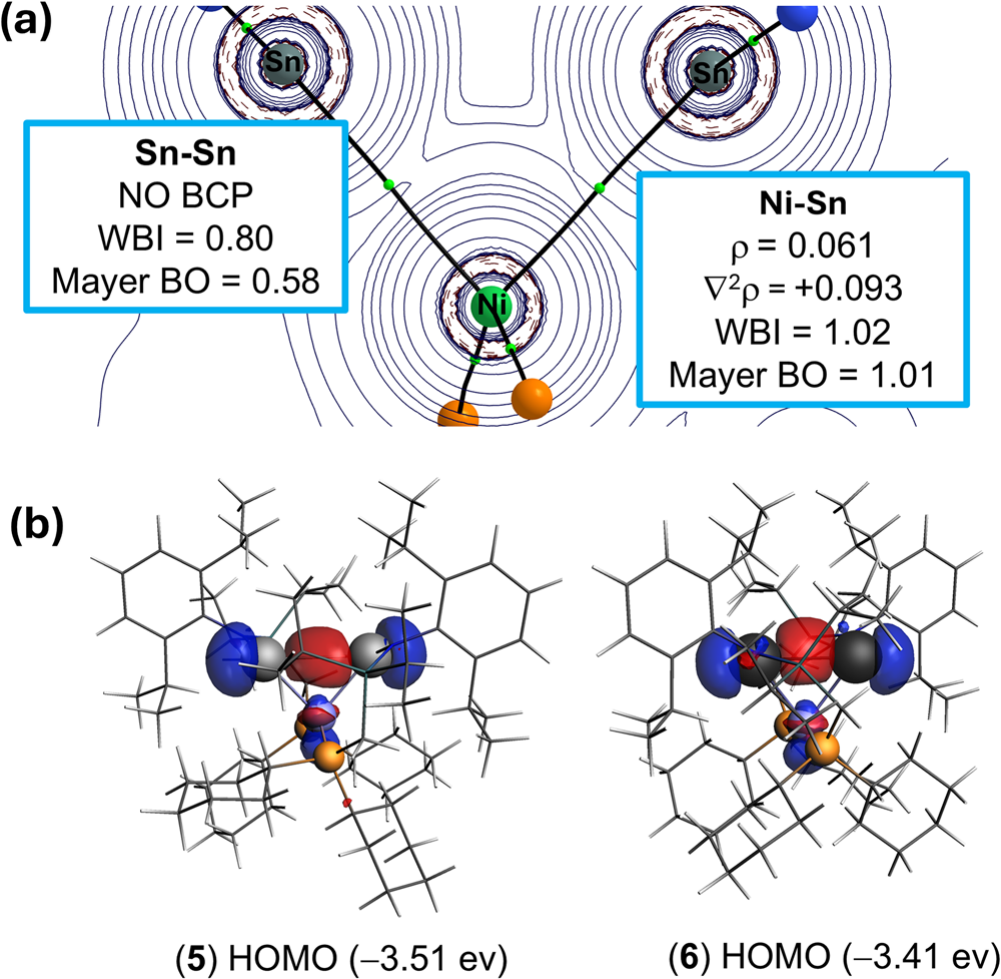
(a) Contour line diagrams ∇^2^ *ρ*(r) computed for **5** in the Sn–Ni–Sn plane. Solid lines connecting the atomic nuclei are bond paths, while the small green spheres indicate the corresponding bond critical points, respectively. (b) Visualization of the HOMO of complexes **5** and **6** (isosurface value of 0.04 a.u.).

Finally, additional DFT calculations were carried out to gain further insight into the bonding situation in the [NiE_2_] cores of **5** and **6**. The optimized geometries align well with those obtained experimentally, particularly in reproducing the E─E bond distances (3.269 Å vs. 3.2686(7) Å in **5**; and 3.216 Å vs. 3.262(1) Å for **6**), further supporting the selected computational method. As expected, QTAIM calculations on **5** (see Figure 5a) locate bond critical points and associated bond paths running between the Ni and Sn atoms. In both cases, the located Ni–Sn bond critical points feature positive values of ∇^2^ *ρ* (+0.093), therefore suggesting donor‐acceptor (that is, dative) bonds. Indeed, the second order perturbation theory (SOPT) of the natural bond orbital (NBO) method indicates that the bonding in the [NiSn_2_] core is best described mainly as a consequence of the donation from the doubly‐occupied σ(Sn─Sn) molecular orbital to the vacant s‐atomic orbital of the transition metal, with an associated SOPT stabilization energy, Δ*E* ^(2)^, of −77.3 kcal/mol. The corresponding backdonation from an occupied d‐atomic orbital to the vacant σ*(Sn–Sn) molecular orbital is comparatively much weaker (Δ*E* ^(2)^ < −5 kcal/mol). A similar bonding picture is found for the [NiPb_2_] core in **6**, although in this case, the σ(Pb─Pb) → s(Ni) donation is lower (Δ*E* ^(2)^ = −41.3 kcal/mol). This suggests that complexes **5** and **6** can be viewed as σ‐complexes rather than π‐complexes, which is consistent with their structural features commented above. In line with this, lone‐pairs are identified, for example, at the Pb centres in **6** in the described NBO analysis, with occupations of 1.94 e at each Pb centre (Figure S116 in the ESI). This is also consistent with the strongly *trans*‐bent [NEEN] (E = Sn, Pb) units in **5** and **6**. Moreover, our QTAIM calculations could not identify the corresponding bond critical point (nor the associated bond path) between the tin atoms (see Figure 5a) [58, 59]. This is somewhat surprising given the relatively high values of the Wiberg bond indices (WBIs) computed for the E–E bond (WBI = 0.80 and 0.87, for E = Sn and E = Pb, respectively) and Mayer bond orders (0.58 and 0.67, respectively), which are consistent with a E─E single bond in species **5** and **6** [60]. Indeed, simple visualization of the HOMO of both species clearly confirms the occurrence of the E–E σ‐bonds in these species (see Figure 5b). The strength of this bond can be also quantified using the natural orbital for chemical valence (NOCV) extension of the energy decomposition analysis (EDA) method (see details in the Supporting Information). According to the NOCV‐EDA calculations, the E─E σ‐bond can be described as an electron‐sharing (i.e., covalent) bond with an associated energy, ΔE(ρ), of −73.1 kcal/mol (E = Sn) and −80.6 kcal/mol (E = Pb). These values further support the relatively strong nature of the E─E interaction and indicate that the Pb–Pb bond is stronger than the Sn─Sn bond, in agreement with the shorter distance observed in the corresponding X**─**‐ray structure, the higher WBI, and the weaker σ‐donation to the Ni centre.

## 3 Conclusion

We have demonstrated pathways for the formation of previously undescribed side‐on transition metal complexes of heavier alkyne analogues. Whilst this could not be achieved by direct addition of the E^I^ dimers (E = Ge, Sn, Pb) to a Ni^0^ synthon, we find that a reductive group‐elimination strategy of tin(II) hydrides and lead(II) vinyl synthons yields the target compounds. A computational investigation of this process suggests that Ni is not directly involved in the elimination process and rather behaves as a redox‐mediator. We believe this may open new opportunities in cooperative bond activation protocols using such low‐valent E^I^ ligands.

## Author Contributions

Leopold Junge carried out most of the experimental work, while Emeric Schubert synthesised and analysed complex **4**. Israel Fernández carried out all computational aspects of the work. Terrance J. Hadlington supervised the experimental aspects of the study and conceived the study. T. J. Hadlington wrote the initial draft of the manuscript, which was subsequently edited by all authors.

## Conflicts of Interest

The authors declare no conflicts of interest.

## Supporting information




**Supporting File 1**: anie72994‐sup‐0001‐SuppMat.pdf.

**Supporting File 2**: anie72994‐sup‐0002‐SuppMat.cif.

## Data Availability

The data that supports the findings of this study are available in the supplementary material of this article.
