# Supplementary material for: Heavier Alkyne‐Ni0 Complexes, [R2E2·Ni] (E = Sn, Pb), Exhibiting σ‐Complex Character
Source: Angew Chem Int Ed Engl. 2026 Jun 4;65(32):e6734116. doi: 10.1002/anie.6734116 (PMC13427110; doi:10.1002/anie.6734116)
Supplement: Supplementary file 1 — Supporting File 1: anie72994‐sup‐0001‐SuppMat.pdf. [file ANIE-65-e6734116-s001.pdf]

Heavier Alkyne-Ni^0 Complexes, [$R_2E_2 \cdot Ni$] (E = Sn, Pb), Exhibiting σ-Complex Character

L. Junge,[a] E. Schubert,[a] I. Fernández,*,[b] and T. J. Hadlington*,[a]

[a] Fakultät für Chemie, Technische Universität München, Lichtenberg Strasse 4, 85747 Garching (Germany); E-mail: terrance.hadlington@tum.de

[b] Departamento de Química Orgánica I and Centro de Innovación en Química Avanzada, Facultad de Ciencias Químicas, Universidad Complutense de Madrid, 28040 Madrid, Spain

1. Experimental methods and data 2

General Considerations........ 2

Synthetic details........ 3

Printed spectra........ 17

3. X-ray crystallographic details........ 71

4. Computational methods and details........ 77

5. References........ 99

1. Experimental methods and data

General considerations. All experiments and manipulations were carried out under dry oxygen free argon atmosphere using standard Schlenk techniques or in an MBraun inert atmosphere glovebox containing an atmosphere of high purity argon. THF and diethyl ether were dried by distillation over a sodium/benzophenone mixture and stored over activated 4Å mol sieves. C_6D_6 was dried, degassed and stored over a potassium mirror. All other solvents were dried over activated 4Å mol sieves and degassed prior to use. ^{Ph}LK,[1] ^{Ph}L(Cl)Ge:,[1] ^{Ph}L(H)Sn: (^{Ph}L = {[$Ph_2PCH_2Si(^iPr)_2$](Dip)N}; Dip = 2,6-$^iPr_2C_6H_3$),[2] ^{Cy}LK,[3] ^{Cy}L(Cl)Ge:,[4] ^{Cy}L(C_2H_3)Pb:,[4] ^{Cy}L(H)Sn: (^{Cy}L = {[$Cy_2PCH_2Si(^iPr)_2$](Dip)N}$^-$,[5] [(^{Mes}nacnac)Mg]$_2$ (^{Mes}nacnac = [HC{MeCNMes)$_2$]$^-$; Mes = 2,4,6-$Me_3C_6H_2$),[6] DipN(H)Li,[7] and Ni(cod)$_2$,[8] were synthesized according to known literature procedures. All other reagents were used as received.

NMR

NMR spectra were recorded on a Bruker AV 400 Spectrometer. The ^{1}H and ^{13}C{^{1}H} NMR spectra were referenced to the residual solvent signals as internal standards. ^{29}Si{^{1}H} NMR spectra were externally calibrated with $SiMe_4$. ^{31}P{^{1}H} NMR spectra were externally calibrated with H_3PO_4. ^{119}Sn{^{1}H} NMR spectra were externally calibrated with $SnMe_4$. The coupling constants J are given in Hz. For signal multiplicities, the following abbreviations were used: s = singlet, d = doublet, t = triplet, q = quartet, p = pentet, sept = septet, m = multiplet, br = broad and combinations thereof.

MS

Liquid Injection Field Desorption Ionization Mass Spectrometry (LIFDI-MS) was measured directly from an inert atmosphere glovebox with a Thermo Fisher Scientific Exactive Plus Orbitrap equipped with an ion source from Linden CMS.

UV/vis

Absorption spectra (UV/vis) were recorded on an Agilent Cary 60 UV/vis spectrophotometer fitted with a fiberoptic coupler feeding into an inert atmosphere glovebox under Ar. Sample preparation was conducted in an argon-filled glovebox by dissolution of each compound in the respective solvent, and the samples collected using a fibreoptic absorption dip-probe, which is directly lowered into each sample. Dilution was conducted in the glovebox as required.

EA

Elemental analyses (C, H, N) were performed with a combustion analyzer (elementar vario EL, Bruker).

Synthetic details and data

Me_2PCH_2Li.

This was accessed via a modified literature procedure.[9]

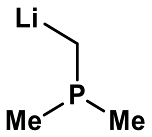

To an oven-dried and degassed 500 mL Schlenk flask equipped with a stirring bar PMe_3 (7.48 g, 10.14 mL, 98.35 mmol) was added, which was diluted in heptane (50 mL), and cooled to 0 °C. To a 500 mL Schlenk tube, ^{t}BuLi was added (1.6M in pentane, 62.03 mL, 99.26 mmol), and the solvent removed cautiously *in vacuo*, resulting in formation of solid ^{t}BuLi. The PMe_3 solution in heptane was added dropwise onto the dry ^{t}BuLi at 0 °C. Then the reaction mixture was heated to 80 °C for 16h without stirring, resulting in the precipitation of a cake of microcrystalline Me_2PCH_2Li. **Caution gas evolution! *tert*-butane is formed during this step.** The supernatant solution was removed from the solid by filtration into an additional Schlenk flask (*N.B. caution; the supernatant solution contains excess ^{t}BuLi, which should be quenched with care*), and the solid dried *in vacuo*, yielding Me_2PCH_2Li (**1**) as an off-white free flowing solid (5.98 g, 72.92 mmol, 78%).

^{1}H NMR (400 MHz, THF-d_8): δ = 0.76 (d, $^4J_{HH}$ = 1.5 Hz, 6H, P-CH_3), -0.93 (s, 2H, P-CH_2-Li).

^{13}C{^{1}H} NMR (101 MHz, THF-d_8): δ = 28.95 (P-CH$_2$-Li), 11.14 (P-CH$_3$), 10.78 (P-CH$_3$).

^{31}P{^{1}H} NMR (162 MHz, THF-d_8): δ = -41.78 (s, Me$_2$-P-CH$_2$).

The analytical data are consistent with the literature values.[9]

^{Me}LK.

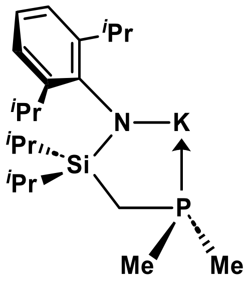

A 500 mL Schlenk flask was loaded with Me_2PCH_2Li (5.00 g, 60.97 mmol), the solid suspended in hexane (50 mL), cooled to -78 °C, and TMEDA (7.08 g, 7.55 mL, 60.97 mmol) added. Neat iPr_2SiCl_2 (11.29 g, 11.0 mL, 60.97 mmol) was then added swiftly. The suspension was allowed to warm to room temperature overnight with continuous stirring. All volatiles were then removed *in vacuo*, yielding a yellow oil. Solid DippN(H)Li (11.17 g, 60.97 mmol) was added directly to this oil, the reaction vessel

cooled to -78 °C, and 100 mL of THF added. The reaction mixture was then stirred for 1.5 h at room temperature, after which time all volatiles were removed *in vacuo*, the residue extracted in hexane (3 x 50 mL), and the extract filtered directly onto a mixture of KH (3.03 g, 75.46 mmol) and KHMDS (0.500 g, 2.5 mmol). All volatiles were again removed *in vacuo*, THF added (125 mL), and the resulting suspension was stirred overnight at room temperature. **Caution: gas evolution! H_2 is formed during this step.** The resulting dark brown suspension was subsequently filtered and all volatiles removed in vacuo, yielding a dark brown oil. This oil was subject to three cycles of adding hexane (80 mL), sonication for 3-5 min, and removing all volatiles in vacuo, resulting in the formation of a dark brown solid. The crude product was purified by washing with hexane (3 x 100 mL) and thoroughly drying in vacuo, yielding ^{MeiP}DippNK (**2**) as an offwhite powder (15.58 g, 38.59 mmol, 63%).

^{1}H NMR (400 MHz, THF-d_8): δ = 6.24 (d, $^{3}J_{HH}$ = 7.3 Hz, 2H, Dipp-^{m}C*H*), 5.68 (t, $^{3}J_{HH}$ = 7.4 Hz, 1H, Dipp-^{p}C*H*), 3.84 (p, $^{3}J_{HH}$ = 6.9 Hz, 1H, Dipp-iPr-C*H*), 3.51 (p, $^{3}J_{HH}$ = 7.0 Hz, 1H, Dipp-iPr-C*H*), 3.17 (d, $^{3}J_{HH}$ = 14.3 Hz, 2H, Si-iPr-C*H*), 0.66–0.59 (m, 24H, Dipp-iPr-CH_3, Si-iPr-CH_3), 0.56 (s, 6H, P-CH_3), 0.31 (d, $^{3}J_{HH}$ = 7.1 Hz, 2H, Si-CH_2-P).

^{13}C{^{1}H} NMR (101 MHz, THF-d_8): δ = 156.40 (Dipp-^{ipso}*C*), 140.02 (Dipp-^{o}*C*), 139.41 (Dipp-^{o}*C*), 122.33 (Dipp-^{m}*C*), 122.25 (Dipp-^{m}*C*), 110.61 (Dipp-^{p}*C*), 58.54, 26.67 (Dipp-iPr-*C*H), 26.47 (Dipp-iPr-*C*H_3/ P-*C*H_3), 26.23 (Si-iPr-*C*H/ Si-iPr-*C*H_3), 25.98 (Si-iPr-*C*H_3/ P-*C*H_3), 19.90 (d, J_{PC} = 4.0 Hz, Si-iPr-*C*H_3/ P-*C*H_3), 18.62 (d, $^{1}J_{PC}$ = 12.8 Hz, Si-*C*H_2-P), 17.96 (d, J_{PC} = 2.6 Hz, Si-iPr-*C*H_3/ P-*C*H_3).

^{29}Si{^{1}H} NMR (79 MHz, THF-d_8): δ = -31.97 (d, $^{2}J_{SiP}$ = 9.3 Hz, CH_2-*Si*-(iPr)$_2$).

^{31}P{^{1}H} NMR (162 MHz, THF-d_8): δ = -59.45 (s, *P*-Me_2).

^{Me}L(Cl)Ge:.

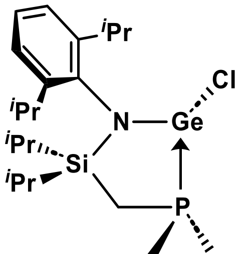

^{MeiP}DippNK (5.00 g, 12.39 mmol) was dissolved in THF (50 mL) and a solution of $GeCl_2$·dioxane (2.87 g, 12.39 mmol) in THF (25 mL) was added dropwise at -78 °C. The reaction mixture was stirred at this temperature for two hours. The reaction mixture was Whatman filtrated and subsequently, all volatiles were removed *in vacuo*. The dark orange residue was washed with hexane (3 x 20 mL) and dried *in vacuo* to yield compound **3** as an orange solid (4.13 g, 8.74 mmol, 71%).

^{1}H NMR (400 MHz, C_6D_6): δ = 7.21 (dd, $^{3}J_{HH}$ = 7.1, 2.3 Hz, 1H, Dipp-^{p}C*H*), 7.14–7.08 (m, 2H, Dipp-^{m}C*H*), 4.19 (hept, $^{3}J_{HH}$ = 6.8 Hz, 1H, Dipp-iPr-C*H*), 3.21 (hept, $^{3}J_{HH}$ = 6.9 Hz, 1H, Dipp-iPr-C*H*), 1.62 (p, $^{3}J_{HH}$ = 7.0 Hz, 2H, Si-iPr-C*H*), 1.49 (d, $^{3}J_{HH}$ = 6.7 Hz, 3H, Dipp-iPr-C*H*$_3$), 1.40 (dd, $^{3}J_{HH}$ = 15.6, 7.2 Hz, 6H, Dipp-iPr-C*H*$_3$), 1.27 (d, $^{3}J_{HH}$ = 7.0 Hz, 3H, Dipp-iPr-C*H*$_3$), 1.21 (d, $^{3}J_{HH}$ = 6.8 Hz, 3H, Si-iPr-C*H*$_3$), 1.14 (dd, $^{3}J_{HH}$ = 7.1, 3.2 Hz, 5H, Si-iPr-C*H*$_3$), 1.09 (d, $^{3}J_{HH}$ = 10.9 Hz, 3H, Si-iPr-C*H*$_3$), 0.95 (s, 3H, P-C*H*$_3$), 0.78 (d, $^{3}J_{HH}$ = 8.8 Hz, 3H, P-C*H*$_3$), 0.68 (d, $^{3}J_{HH}$ = 7.5 Hz, 2H, Si-C*H*$_2$-P).

^{13}C{^{1}H} NMR (101 MHz, C_6D_6): δ = 149.27 (d, J_{PC} = 2.2 Hz, Dipp-^{ipso}*C*), 146.31 (Dipp-^{o}*C*), 143.46 (d, J_{PC} = 12.5 Hz, Dipp-^{o}*C*), 125.02 (Dipp-^{m}*C*), 124.31 (Dipp-^{p}*C*), 123.52 (Dipp-^{m}*C*), 28.68 (Dipp-iPr-*C*H), 28.39 (Dipp-iPr-*C*H$_3$), 28.33 (Dipp-iPr-*C*H$_3$), 27.79 (Dipp-iPr-*C*H), 23.11 (Dipp-iPr-*C*H$_3$), 22.60 (Dipp-iPr-*C*H$_3$), 20.49 (Si-iPr-*C*H$_3$), 20.14 (Si-iPr-*C*H$_3$), 19.74 (Si-iPr-*C*H$_3$), 17.90 (Si-iPr-*C*H$_3$), 17.08 (d, $^{3}J_{PC}$ = 5.5 Hz, Si-iPr-*C*H), 16.06 (d, $^{3}J_{PC}$ = 1.8 Hz, Si-iPr-*C*H), 10.54 (d, $^{1}J_{PC}$ = 7.7 Hz, P-*C*H$_3$), 10.35 (d, $^{2}J_{PC}$ = 4.8 Hz, P-*C*H$_3$), 7.38 (Si-*C*H$_2$-P).

^{29}Si{^{1}H} NMR (79 MHz, C_6D_6): δ = 15.82 (d, $^{2}J_{SiP}$ = 9.3 Hz, CH$_2$-*Si*-(iPr)$_2$).

^{31}P{^{1}H} NMR (162 MHz, C_6D_6): δ = -12.24 (s, *P*-Me$_2$).

MS/LIFDI-HRMS found (calcd.) m/z: 473.1496 (473.1489) [M]$^+$

^{Me}L(Br)Sn:.

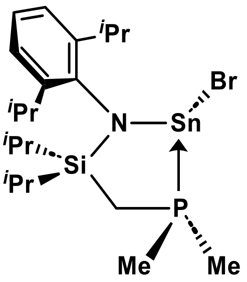

A solution of SnBr$_2$ (1.72 g, 6.19 mmol) in THF (10 mL) was added dropwise to a stirring pale brown solution of ^{MeiP}DippNK (2.50 g, 6.19 mmol) in 20 mL THF at −78 °C and stirred for one hour. Subsequently all volatiles were removed *in vacuo*, the residue extracted in hexane (40 mL), and filtered. The solvent was removed *in vacuo* to yield compound **4** as an off-white solid (3.00 g, 5.33 mmol, 86%).

^{1}H NMR (400 MHz, THF-*d*$_8$): δ = 7.07–6.98 (m, 2H, Dipp-^{m}C*H*), 6.94–6.87 (m, 1H, Dipp-^{p}C*H*), 3.73–3.60 (m, 2H, Dipp-iPr-C*H*), 1.73 (s, 5H, Dipp-iPr-C*H*$_3$), 1.59 (d, $^{3}J_{HH}$ = 15.4 Hz, 2H, Si-iPr-C*H*), 1.25 (d, $^{3}J_{HH}$ = 6.8 Hz, 6H, Dipp-iPr-C*H*$_3$), 1.21 (d, $^{3}J_{HH}$ = 6.8 Hz, 2H, Si-C*H*$_2$-P), 1.17–1.10

(m, 12H, Si-iPr-CH_3), 0.97 (s, 4H, Si-iPr-CH_3), 0.91–0.81 (m, 1H, Si-CH_2-P), 0.82 (d, $^3J_{HH}$ = 6.6 Hz, 6H, P-CH_3).

^{13}C{^{1}H} NMR (101 MHz, THF-*d*$_8$): δ = 146.36 (Dipp-^{ipso}C), 144.97 (d, J_{PC} = 8.1 Hz, Dipp-^{o}C), 123.06 (Dipp-^{m}C), 122.87 (Dipp-^{p}C), 27.50 (Si-iPr-CH$_3$), 27.15 (Dipp-iPr-CH), 22.41 (Dipp-iPr-CH$_3$), 19.67 (Si-iPr-CH$_3$), 18.19 (P-CH$_3$), 16.49 (d, J_{PC} = 2.9 Hz, Si-iPr-CH$_3$, Si-CH$_2$-P), 10.58 (d, J_{PC} = 16.6 Hz, Dipp-iPr-CH$_3$), 9.67 (Si-iPr-CH).

^{29}Si{^{1}H} NMR (79 MHz, THF-*d*$_8$): δ = 11.65 (d, $^2J_{PSi}$ = 5.9 Hz, CH$_2$-*Si*-(iPr)$_2$).

^{31}P{^{1}H} NMR (162 MHz, THF-*d*$_8$): δ = -17.75 (s, $^1J_{117SnP}$ = 778 Hz, $^1J_{119SnP}$ = 816 Hz, *P*-Me$_2$).

^{119}Sn NMR (112 MHz, C_6D_6): δ = -63.73 (d, $^1J_{SnP}$ = 1540.4 Hz, N-*Sn*-P).

MS/LIFDI-HRMS found (calcd.) m/z: 563.0749 (563.0787) $[M]^+$

Anal. calcd. $C_{21}H_{39}BrNPSiSn$: C, 44.78%; H, 6.98%; N, 2.49%; found: C, 33.83%; H, 5.25%; N, 1.98%.

N.B. A high C value was obtained for numerous samples, which we attribute to incomplete combustion, *e.g.* due to formation of silicon carbide.

^{Cy}L(Br)Sn:.

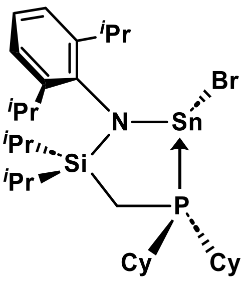

A solution of $SnBr_2$ (1.55 g, 5.56 mmol) in THF (10 mL) was added dropwise to a stirring pale brown solution of ^{CyiP}DippNK (3.0 g, 5.56 mmol) in THF (20 mL) at -78 °C. The reaction mixture was allowed to warm up to room temperature and stirred for two hours. Subsequently, all volatiles were removed *in vacuo*, the residue extracted in toluene (30 mL), and filtered. The solvent was removed *in vacuo* to yield compound **5** as an off-white solid (1.98 g, 2.83 mmol, 51%).

^{1}H NMR (400 MHz, C_6D_6): δ = 7.22 (t, $^3J_{HH}$ = 4.7 Hz, 1H, Dipp-^{p}CH), 7.11 (d, $^3J_{HH}$ = 4.4 Hz, 2H, Dipp-^{m}CH), 4.32 (hept, $^3J_{HH}$ = 6.8 Hz, 1H, Dipp-iPr-CH), 4.32 (hept, $^3J_{HH}$ = 6.8 Hz, 1H, Dipp-iPr-CH), 2.49–2.39 (m, 1H, Cy-CH-P) 1.93–1.83 (m, 2H, Si-iPr-CH), 1.66–1.59 (m, 5H, Cy-CH), 1.54 (d, $^3J_{HH}$ = 6.6 Hz, 5H, Cy-CH), 1.49–1.43 (m, 5H, Cy-CH), 1.36 (d, $^3J_{HH}$ = 6.8 Hz, 3H, Dipp-iPr-CH_3), 1.24–1.18 (m, 7H, Cy-CH), 1.16–1.08 (m, 6H, Si-iPr-CH_3), 1.06–1.03 (m, 3H, Dipp-iPr-CH_3), 0.72 (d, $^3J_{HH}$ = 6.8 Hz, 2H, Si-CH_2-P).

$^{13}C\{^1H\}$ NMR (101 MHz, C_6D_6): δ = 148.86 (d, J_{PC} = 1.45 Hz, Dipp-^{ipso}C), 145.59 (Dipp-^{o}C), 144.81 (d, J_{PC} = 7.3 Hz, Dipp-^{o}C), 124.38 (Dipp-^{m}C), 124.06 (Dipp-^{p}C), 123.73 (Dipp-^{m}C), 35.02 (d, $^3J_{PC}$ = 12.5 Hz, Si-iPr-*C*H), 34.72 (d, J_{PC} = 6.8 Hz, Cy-*C*H-P), 31.27 (d, J_{PC} = 3.0 Hz, Si-iPr-*C*H_3), 30.40 (Si-iPr-C*H*), 30.22 (Cy-*C*H), 29.45 (d, J_{PC} = 4.3 Hz, Cy-*C*H), 29.13 (Cy-*C*H), 28.90 (Cy-*C*H), 28.25 (Dipp-iPr-C*H*), 27.91 (Si-iPr-*C*H_3), 27.79 (Si-iPr-*C*H_3), 27.61 (d, J_{PC} = 2.5 Hz, Cy-*C*H), 27.51 (Si-iPr-*C*H_3), 27.42 (Cy-*C*H), 27.23 (Dipp-iPr-C*H*), 26.10 (Cy-*C*H), 23.71 (Dipp-iPr-*C*H_3), 23.13 (Dipp-iPr-*C*H_3), 20.60 (Dipp-iPr-*C*H_3), 20.44 (Dipp-iPr-*C*H_3), 20.12 (Cy-*C*H), 18.12 (Dipp-iPr-*C*H_3), 17.96 (d, $^1J_{PC}$ = 3.8 Hz, Si-*C*H_2-P), 15.95 (d, J_{PC} = 2.6 Hz, Cy-*C*H), 0.98 (d, J_{PC} = 5.9 Hz, Cy-*C*H).

$^{29}Si\{^1H\}$ NMR (79 MHz, C_6D_6): δ = 13.91 (d, $^2J_{SiP}$ = 6.4 Hz, CH_2-*Si*-$(iPr)_2$).

$^{31}P\{^1H\}$ NMR (162 MHz, C_6D_6): δ = 14.83 (s, $^1J_{117SnP}$ = 749 Hz, $^1J_{119SnP}$ = 785 Hz, *P*-Cy_2).

^{119}Sn NMR (112 MHz, C_6D_6): δ = -26.71 (d, $^1J_{SnP}$ = 1566.7 Hz, N-*Sn*-P).

MS/LIFDI-HRMS found (calcd.) m/z: 699.2024 (699.2041) $[M]^+$

Anal. calcd. $C_{31}H_{55}BrNPSiSn$: C, 53.23%; H, 7.93%; N, 2.00%; found: C, 54.55%; H, 8.10%; N, 2.03%.

$^{Ph}L(Br)Sn$:.

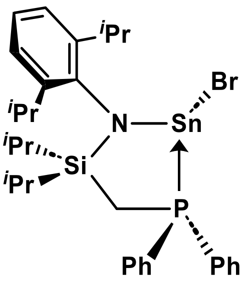

A solution of $SnBr_2$ (1.55 g, 5.56 mmol) in THF (10 mL) was added dropwise to a stirring pale brown solution of ^{PhiP}DippNK (3.0 g, 5.56 mmol) in THF (20 mL) at -78 °C. The reaction mixture was allowed to warm up to room temperature and stirred for two hours. Subsequently, all volatiles were removed *in vacuo*, the residue extracted in toluene (30 mL), and filtered. The solvent was removed *in vacuo* to yield compound **6** as an off-white solid (1.15 g, 1.67 mmol, 88%).

^1H NMR (400 MHz, C_6D_6): δ = 7.69–7.61 (m, 2H, Ar-C*H*), 7.61–7.52 (m, 1H, Ar-C*H*), 7.40–7.28 (m, 2H, Ar-C*H*), 7.12–6.94 (m, 8H, Ar-C*H*), 4.29 (hept, $^3J_{HH}$ = 6.8 Hz, 1H, Dipp-iPr-C*H*), 2.82 (hept, $^3J_{HH}$ = 7.0 Hz, 1H, Dipp-iPr-C*H*), 2.33 (dd, $^3J_{HH}$ = 14.5, 10.5 Hz, 1H, Si-CH_2-P), 2.13–1.96 (m, 1H, Si-CH_2-P), 1.61 (t, $^3J_{HH}$ = 2.0 Hz, 3H, Si-iPr-CH_3), 1.44 (dd, $^3J_{HH}$ = 6.8, 4.7 Hz, 6H, Dipp-iPr-CH_3), 1.23–1.13 (m, 6H, Dipp-iPr-CH_3), 1.01 (q, $^3J_{HH}$ = 7.3 Hz, 5H, Si-iPr-CH_3, Si-iPr-C*H*), 0.87 (dd, $^3J_{HH}$ = 13.3, 6.8 Hz, 6H, Dipp-iPr-CH_3), 0.70 (hept, $^3J_{HH}$ = 7.5 Hz, 1H, Si-iPr-C*H*), 0.60 (d, $^3J_{HH}$ = 7.2 Hz, 3H, Si-iPr-CH_3).

$^{13}C\{^1H\}$ NMR (101 MHz, C_6D_6): δ = 133.56 (d, J_{PC} = 12.1 Hz, Ph-*C*), 132.54 (d, J_{PC} = 10.3 Hz, Dipp-^{o}*C*, Dipp-^{m}*C*), 131.55 (d, J_{PC} = 2.2 Hz, Ph-*C*), 130.76 (d, J_{PC} = 2.6 Hz, Ph-*C*), 129.22 (d, J_{PC} = 9.5 Hz, Ph-*C*), 128.97 (d, J_{PC} = 9.9 Hz, Ph-*C*), 124.29 (Ph-*C*), 124.14 (Ph-*C*), 123.91 (Ph-*C*), 30.23 (Dipp-iPr-*C*H_3), 29.39 (Dipp-iPr-*C*H_3), 29.16 (Dipp-iPr-*C*H_3), 28.59 , 28.17, 27.62 (Dipp-iPr-C*H*), 27.34 (Dipp-iPr-C*H*), 23.98 (Dipp-iPr-*C*H_3), 22.99 (d, *J* = 4.0 Hz), 22.60, 20.93 (Si-iPr-*C*H_3), 20.65 (Si-iPr-*C*H_3), 20.26 (Si-iPr-*C*H_3), 18.37 (d, *J* = 2.2 Hz), 18.21 (Si-iPr-*C*H_3), 16.77 (d, $^3J_{PC}$ = 2.6 Hz, Si-iPr-C*H*), 16.26 (d, $^3J_{PC}$ = 2.9 Hz, Si-iPr-C*H*), 6.82 (d, $^1J_{PC}$ = 3.3 Hz, Si-C*H_2*-P).

$^{29}Si\{^1H\}$ NMR (79 MHz, C_6D_6): δ = 11.12 (d, $^2J_{SiP}$ = 7.3 Hz, CH_2-*Si*-(iPr)$_2$).

$^{31}P\{^1H\}$ NMR (162 MHz, C_6D_6): δ = -2.03 (s, $^1J_{117SnP}$ = 716 Hz, $^1J_{119SnP}$ = 750 Hz, *P*-Ph_2).

^{119}Sn NMR (112 MHz, C_6D_6): δ = -57.93 (d, $^1J_{SnP}$ = 1503.8 Hz, N-*Sn*-P).

MS/LIFDI-HRMS found (calcd.) m/z: 608.1901 (608.1930) $[M-Br]^+$

(^{Me}LGe)$_2$, 1-Me.

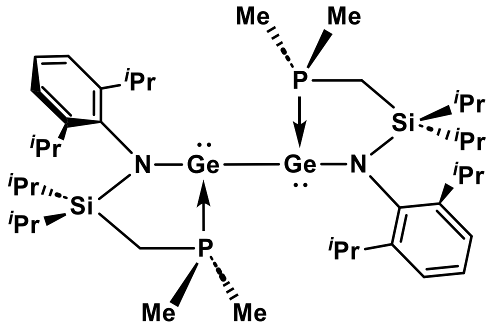

^{MeiP}DippGeCl (715 mg, 1.51 mmol, 2.0 eq.) and [Mg_2(^{Mes}nacnac)$_2$] (542 mg, 756 µmol, 1.0 eq.) were each placed in a Schlenk flask and toluene (40 mL) was added to both. The ^{MeiP}DippGeCl was cooled to -78 °C and the [Mg_2(^{Mes}nacnac)$_2$] solution was added dropwise. After stirring for one hour, all volatiles were removed *in vacuo* and the residue was extracted with hexane. Concentrating and storing the solution overnight at -35 °C, yielded compound **7** as dark orange crystals (285 mg, 326 µmol, 60%).

^1H NMR (400 MHz, C_6D_6): δ = 7.14–7.08 (m, 4H, Dipp-^{m}C*H*), 7.06–6.99 (m, 2H, Dipp-^{p}C*H*), 3.80 (dq, $^3J_{HH}$ = 26.7, 6.4 Hz, 4H, Dipp-iPr-C*H*), 1.59 (d, $^3J_{HH}$ = 6.7 Hz, 5H, Si-iPr-C*H_3*), 1.34 (d, $^3J_{HH}$ = 6.8 Hz, 12H, Dipp-iPr-C*H_3*), 1.28 (d, $^3J_{HH}$ = 6.7 Hz, 7H, Dipp-iPr-C*H_3*, Si-iPr-C*H*), 1.25 (s, 1H, Si-iPr-C*H*), 1.22 (d, $^3J_{HH}$ = 7.5 Hz, 6H, Dipp-iPr-C*H_3*), 1.17–1.11 (m, 12H, P-C*H_3*), 1.10 (d, $^3J_{HH}$ = 3.2 Hz, 1H, Si-iPr-C*H*), 1.07–1.01 (m, 18H, Si-iPr-C*H_3*), 0.97 (d, $^3J_{HH}$ = 3.3 Hz, 1H, Si-iPr-C*H*), 0.91–0.85 (m, 4H, Si-C*H_2*-P).

$^{13}C\{^1H\}$ NMR (101 MHz, C_6D_6): δ = 149.09 (Dipp-^{ipso}*C*), 147.78(Dipp-^{o}*C*), 146.96 (Dipp-^{o}*C*), 124.69 (Dipp-^{m}*C*H), 123.64 (Dipp-^{m}*C*H), 122.55 (Dipp-^{p}*C*H), 28.80 (Dipp-iPr-C*H*), 27.62 (Si-iPr-*C*H_3), 27.22 (Si-iPr-*C*H_3), 26.81 (Dipp-iPr-C*H*), 25.68 (Dipp-iPr-*C*H_3), 25.02

(Dipp-iPr-CH_3), 21.27 (Si-iPr-CH_3), 20.49 (Dipp-iPr-CH_3), 19.52 (d, J_{PC} = 10.6 Hz, Si-iPr-CH_3), 17.65 (t, J_{PC} = 11.7 Hz, P-CH_3) 16.47 (Si-iPr-CH), 15.22 (d, J_{PC} = 16.5 Hz, Si-iPr-CH), 13.38 (Si-CH_2-P).

^{29}Si{^{1}H} NMR (79 MHz, C_6D_6): δ = 4.76 (t, $^2J_{SiP}$ = 4.2 Hz, CH_2-*Si*-(iPr)$_2$).

^{31}P{^{1}H} NMR (162 MHz, C_6D_6): δ = -13.80 (s, *P*-Me_2).

λ_{max}, nm (ε, Lmol^{-1} cm^{-1}): 355 (21847), 510 (1022).

MS/LIFDI-HRMS found (calcd.) m/z: 874.3633 (874.3626) $[M]^+$

Anal. calcd. $C_{42}H_{78}Ge_2N_2P_2Si_2$: C, 57.69%; H, 8.99%; N, 3.20%; found: C, 55.51%; H, 9.04%; N, 3.35%.

(^{Me}LSn)$_2$, 2-Me.

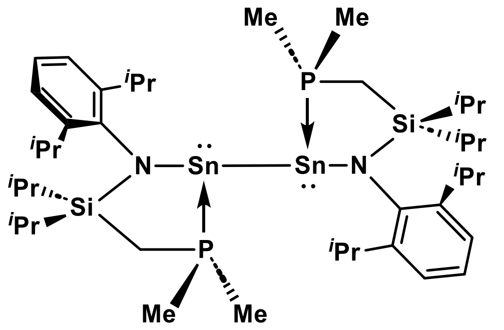

^{MeiP}DippSnBr (941 mg, 1.67 mmol, 2.0 eq.) and [Mg_2(^{Mes}nacnac)$_2$] (600 mg, 836 µmol, 1.0 eq.) were each placed in a Schlenk flask and toluene (40 mL) was added to both. The ^{MeiP}DippSnBr was cooled to -78 °C and the [Mg_2(^{Mes}nacnac)$_2$] solution was added dropwise. After stirring for one hour, all volatiles were removed *in vacuo* and the residue was extracted with hexane (30 mL). Concentrating and storing the solution overnight at -35 °C, yielded compound **8** as dark green solid (127 mg, 131.4 µmol, 16%).

^{1}H NMR (400 MHz, C_6D_6): δ = 7.13–7.08 (m, 4H, Dipp-^{m}C*H*) 7.06–7.00 (m, 2H, Dipp-^{p}C*H*), 3.97 (hept, $^3J_{HH}$ = 6.9 Hz, 2H, Dipp-iPr-C*H*), 3.82 (hept, $^3J_{HH}$ = 6.9 Hz, 2H, Dipp-iPr-C*H*), 1.61–1.49 (m, 3H, Si-iPr-C*H*), 1.44 (d, $^3J_{HH}$ = 6.8 Hz, 6H, Dipp-iPr-CH_3), 1.38 (dd, $^3J_{HH}$ = 14.1, 7.0 Hz, 12H, Dipp-iPr-CH_3), 1.27 (d, $^3J_{HH}$ = 7.4 Hz, 6H, Dipp-iPr-CH_3), 1.24–1.20 (m, 6H, Si-iPr-CH_3), 1.15 (d, $^3J_{HH}$ = 7.6 Hz, 6H, Si-iPr-CH_3), 1.12 (dd, $^3J_{HH}$ = 7.6, 3.0 Hz, 12H, Si-iPr-CH_3), 1.05 (d, $^3J_{HH}$ = 6.8 Hz, 6H, P-CH_3), 1.01 (t, $^3J_{HH}$ = 4.4 Hz, 6H, P-CH_3), 0.90 (dd, $^3J_{HH}$ = 14.1, 7.9 Hz, 4H, Si-CH_2-P).

^{13}C{^{1}H} NMR (101 MHz, C_6D_6): δ = 151.28 (s, Dipp-^{ipso}*C*), 147.04 (d, J_{PC} = 7.3 Hz, Dipp-^{o}*C*), 124.54 (Dipp-^{m}*C*), 123.54 (Dipp-^{m}*C*), 122.54 (Dipp-^{p}*C*), 28.56 (Dipp-iPr-*C*H), 27.97 (Dipp-iPr-*C*H), 27.71 (d, J_{PC} = 4.0 Hz, Dipp-iPr-*C*H_3), 24.57 (Dipp-iPr-*C*H_3), 24.12 (Dipp-iPr-*C*H_3), 21.21 (Dipp-iPr-*C*H_3), 20.70 (Si-iPr-*C*H_3), 20.23 (Si-iPr-*C*H_3), 19.54 (Si-iPr-*C*H_3), 18.78 (t, , J_{PC} = 8.4 Hz, Si-iPr-*C*H_3), 17.44 (t, J_{PC} = 2.6 Hz Si-iPr-*C*H_3), 16.59 (t, $^1J_{PC}$ = 4.2 Hz, P-*C*H_3), 16.04 (t, *J* = 3.3 Hz, P-*C*H_3), 15.31 (Si-*C*H_2-P).

$^{29}Si\{^1H\}$ NMR (79 MHz, C_6D_6): δ = -3.52 (s, CH_2-*Si*-$(iPr)_2$).

$^{31}P\{^1H\}$ NMR (162 MHz, C_6D_6): δ = -35.12 (s, $^1J_{SnP}$ = 816 Hz, *P*-Me_2).

^{119}Sn NMR (112 MHz, C_6D_6): δ = -82.26 (d, $^1J_{SnP}$ = 1519.8 Hz, N-*Sn*-P).

λ_{max}, nm (ε, $Lmol^{-1}$ cm^{-1}): 376 (28946), 600 (1267).

MS/LIFDI-HRMS found (calcd.) m/z: 966.3244 (966.3231) $[M]^+$

Anal. calcd. $C_{42}H_{78}N_2P_2Si_2Sn_2$: C, 52.19%; H, 8.13%; N, 2.90%; found: C, 51.84%; H, 8.02%; N, 2.90%.

$(^{Ph}LSn)_2$, 2-Ph.

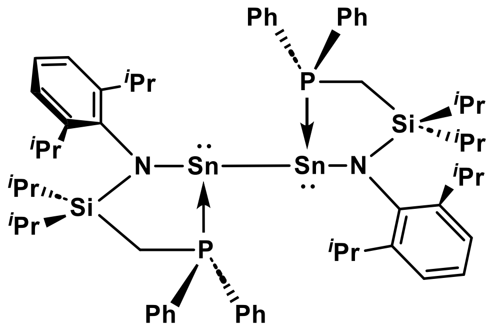

^{PhiP}DippSnBr (1.05 g, 1.53 mmol, 2.0 eq.) and $[Mg_2(^{Mes}nacnac)_2]$ (550 mg, 766 µmol, 1.0 eq.) were each placed in a Schlenk flask and toluene (40 mL) was added to both. The ^{PhiP}DippSnBr was cooled to -78 °C and the $[Mg_2(^{Mes}nacnac)_2]$ solution was added dropwise. After stirring for one hour at this temperature and one additional hour at room temperature, all volatiles were removed *in vacuo* and the residue was extracted with pentane. Concentrating and storing the solution overnight at -35 °C, yielded compound **10** as dark brown crystals (478 mg, 393 µmol, 51%).

^1H NMR (400 MHz, C_6D_6): δ = 7.64 (brs, 2H, Dipp-^{p}C*H*), 7.23 (brs, 4H, Dipp-^{m}C*H*), 7.12–7.06 (m, 20H, Ph-*H*), 3.80 (brs, 4H, Dipp-iPr-C*H*), 1.76 (d, $^3J_{HH}$ = 13.6 Hz, 13H, Dipp-iPr-C*H*$_3$), 1.64–1.60 (m, 4H, Si-iPr-C*H*), 1.21 (d, $^3J_{HH}$ = 6.9 Hz, 14H, Dipp-iPr-C*H*$_3$), 1.19–1.15 (m, 12H, Si-iPr-C*H*$_3$), 1.02 – 0.98 (m, 14H, Si-iPr-C*H*$_3$), 0.71 (d, $^3J_{HH}$ = 7.3 Hz, 1H, Si-C*H*$_2$-P), 0.60 (d, $^3J_{HH}$ = 7.1 Hz, 1H, Si-C*H*$_2$-P).

$^{13}C\{^1H\}$ NMR (101 MHz, C_6D_6): δ = 149.60 (Dipp-^{ipso}*C*), 146.57 (Dipp-^{o}*C*), 144.29 (Dipp-^{o}*C*), 135.35, 135.16, 134.36 (Dipp-^{p}*C*), 133.11, 129.91, 132.30 (Dipp-^{m}*C*), 124.17 (Ph-*C*), 123.45 (Ph-*C*), 122.85 (Ph-*C*), 34.45 (Si-iPr-*C*H$_3$), 28.59, 27.77 (Dipp-iPr-*C*H), 23.98 (Dipp-iPr-*C*H$_3$), 22.74 (Dipp-iPr-*C*H$_3$), 20.49, 19.23 (Si-iPr-*C*H$_3$), 18.36 (Si-iPr-*C*H$_3$), 17.21, 15.41 (Dipp-iPr-*C*H$_3$), 14.29 (Dipp-iPr-*C*H$_3$).

$^{29}Si\{^1H\}$ NMR (79 MHz, C_6D_6): *not observed*

$^{31}P\{^1H\}$ NMR (162 MHz, C_6D_6): δ = -0.45 (t, $^2J_{SiP}$ = 70.40 Hz, $^1J_{SnP}$ = 712 Hz, *P*-Ph_2).

^{119}Sn NMR (112 MHz, C_6D_6): *not observed*

λ_{max}, nm (ε, Lmol^{-1} cm^{-1}): 473 (5200).

MS/LIFDI-HRMS found (calcd.) m/z: 1214.3706 (1214.3864) $[M]^+$

Anal. calcd. $C_{62}H_{86}N_2P_2Si_2Sn_2$: C, 61.29%; H, 7.14%; N, 2.31%; found: C, 61.47%; H, 7.42%; N, 2.40%.

(^{Cy}LSn)$_2$, 2-Cy.

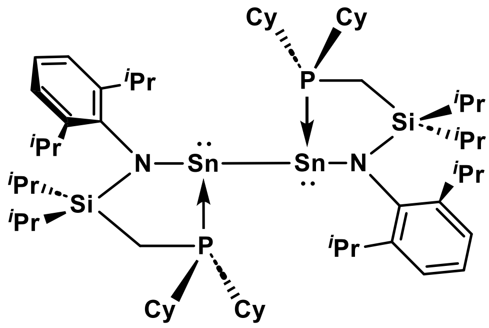

^{CyiP}DippSnBr (335 mg, 479 µmol, 2.0 eq.) and [Mg_2(^{Mes}nacnac)$_2$] (172 mg, 239 µmol, 1.0 eq.) were each placed in a Schlenk flask and toluene (40 mL) was added to both. The ^{CyiP}DippSnBr was cooled to -78 °C and the [Mg_2(^{Mes}nacnac)$_2$] solution was added dropwise. After stirring for one hour, all volatiles were removed *in vacuo* and the residue was extracted with hexane (20 mL). Concentrating and storing the solution overnight at -35 °C, yielded compound **9** as green crystals (270 mg, 218 µmol, 91%).

^{1}H NMR (400 MHz, C_6D_6): δ = 7.13 (d, $^3J_{HH}$ = 7.1 Hz, 4H, Dipp-^{m}C*H*), 6.84–6.80 (m, 2H, Dipp-^{p}C*H*), 4.07–3.81 (m, 4H, Dipp-iPr-C*H*), 2.37–2.34 (m, 3H, Si-iPr-C*H*), 2.23–2.10 (m, 5H, Dipp-iPr-C*H*$_3$), 2.06 (s, 5H, Dipp-iPr-C*H*$_3$), 1.96 (s, 5H, Dipp-iPr-C*H*$_3$), 1.79–1.58 (m, 15H, Cy-*H*), 1.50 (d, $^3J_{HH}$ = 1.3 Hz, 5H, Dipp-iPr-C*H*$_3$), 1.41 (d, $^3J_{HH}$ = 6.8 Hz, 14H, Si-iPr-C*H*$_3$), 1.35–1.28 (m, 12H, Si-iPr-C*H*$_3$), 1.24–0.94 (m, 19H, Cy-*H*), 1.04–0.89 (m, 9H, Cy-H, Si-C*H*$_2$-P).

^{13}C{^{1}H} NMR (101 MHz, THF-*d*$_8$): δ = 168.93 (Dipp-^{ipso}*C*), 145.45 (Dipp-^{o}*C*), 144.57 (Dipp-^{o}*C*), 133.79 (Dipp-^{ipso}*C*), 132.48 (Dipp-^{o}*C*), 129.55, 129.27 (Dipp-^{m}*C*), 123.37 (Dipp-^{p}*C*), 94.71, 36.48 (Cy-*C*H), 36.30 (Cy-*C*H), 31.37 (Cy-*C*H), 31.23 (Cy-*C*H), 30.28 (Cy-*C*H), 30.18 (Cy-*C*H), 30.08 (Cy-*C*H), 29.90 (Cy-*C*H), 29.77 (Cy-*C*H), 28.62 (d, J_{PC} = 1.8 Hz, Cy-*C*H), 28.31 (Cy-*C*H), 28.21 (Cy-*C*H), 28.12 (Cy-*C*H), 28.04 (Dipp-iPr-*C*H), 27.91 (Cy-*C*H), 27.63 (Cy-*C*H), 27.48 (Cy-*C*H), 27.30 (Dipp-iPr-*C*H), 26.92 (Dipp-iPr-*C*H), 26.81 (Cy-*C*H), 26.73 (Cy-*C*H), 23.98 (Si-iPr-*C*H$_3$), 23.64 (Si-iPr-*C*H$_3$), 23.17 (Dipp-iPr-*C*H$_3$), 23.01 (Dipp-iPr-*C*H$_3$), 20.86 (t, 2.94 Hz, Si-*C*H$_2$-P), 20.74 (Dipp-iPr-*C*H$_3$), 20.08, 19.64, 19.36 (Si-iPr-*C*H$_3$), 19.22 (Si-iPr-*C*H$_3$), 19.02 (Si-iPr-*C*H$_3$), 18.73 (Si-iPr-*C*H$_3$), 18.59 (dd, J_{PC} = 4.8, 2.6 Hz, Si-iPr-*C*H$_3$, Si-*C*H$_2$-P), 15.04 (d, J_{PC} = 2.2 Hz, Si-iPr-*C*H$_3$), 2.25 (Si-*C*H$_2$-P), 1.87 (Si-*C*H$_2$-P).

^{29}Si{^{1}H} NMR (79 MHz, THF-*d*$_8$): δ = 3.67 (s, CH$_2$-*Si*-(iPr)$_2$).

^{31}P{^{1}H} NMR (162 MHz, C_6D_6): δ = 6.33 (s, *P*-Me$_2$).

^{119}Sn NMR (112 MHz, C_6D_6): *not observed.*

λ_{max}, nm (ε, Lmol^{-1} cm^{-1}): 351 (29617), 664 (493).

MS/LIFDI-HRMS found (calcd.) m/z: 620.2862 (620.2863) [M-^{Cy}LSn]$^+$

Anal. calcd. $C_{62}H_{110}N_2P_2Si_2Sn_2$: C, 60.10%; H, 8.95%; N, 2.26%; found: C, 60.26%; H, 8.67%; N, 2.63%.

[(^{Ph}LGe)$_2$·Ni], 3.

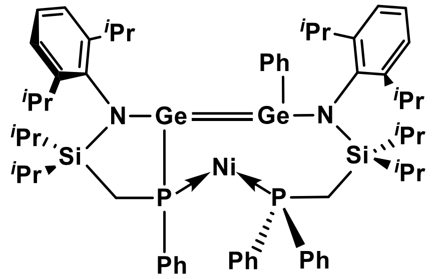

(^{PhiP}DippGe)$_2$ (715 mg, 1.51 mmol, 2.0 eq.) and Ni(cod)$_2$ (37.4 mg, 136 µmol, 1.0 eq.) were each placed in a Schlenk flask and toluene (15 mL) was added to both. The (^{PhiP}DippGe)$_2$ was cooled to -78 °C and the Ni(cod)$_2$ solution was added dropwise. After stirring for one hour at room temperature, the reaction mixture was heated to 70 °C and stirred overnight. Subsequently all volatiles were removed *in vacuo* and the residue was extracted with hexane (20 mL). Concentrating and storing the solution overnight at -35 °C, yielded compound **13** as brown crystals (45 mg, 38 µmol, 29%).

^{1}H NMR (400 MHz, C_6D_6): δ = 8.24–8.14 (m, 2H, Dipp-^{p}C*H*), 7.99–7.83 (m, 4H, Dipp-^{m}C*H*), 7.29–7.18 (m, 3H, Ph-C*H*), 7.15–6.92 (m, 15H, Ph-C*H*), 6.86 (dd, $^3J_{HH}$ = 6.6, 2.8 Hz, 1H, Ph-C*H*), 4.19 (hept, $^3J_{HH}$ = 6.9 Hz, 1H, Dipp-iPr-C*H*), 3.96 (hept, $^3J_{HH}$ = 6.8 Hz, 1H, Dipp-iPr-C*H*), 3.19 (hept, $^3J_{HH}$ = 7.0 Hz, 1H, Dipp-iPr-C*H*), 2.59 (hept, $^3J_{HH}$ = 6.8 Hz, 1H, Dipp-iPr-C*H*), 2.45–2.36 (m, 1H, Si-iPr-C*H*), 1.95–1.81 (m, 3H, Si-iPr-C*H*), 1.79 (d, $^3J_{HH}$ = 6.7 Hz, 3H, Dipp-iPr-C*H*$_3$), 1.61–1.56 (m, 6H, Dipp-iPr-C*H*$_3$, Si-iPr-C*H*$_3$), 1.46–1.41 (m, 4H, Si-C*H*$_2$-P), 1.35 (d, $^3J_{HH}$ = 6.8 Hz, 3H, Dipp-iPr-C*H*$_3$), 1.28 (d, $^3J_{HH}$ = 6.9 Hz, 3H, Dipp-iPr-C*H*$_3$), 1.01–0.95 (m, 12H, Si-iPr-C*H*$_3$), 0.93–0.81 (m, 11H, Si-iPr-C*H*$_3$), 0.74 (m, 3H, Si-iPr-C*H*$_3$), 0.65 (d, $^3J_{HH}$ = 7.1 Hz, 3H, Si-iPr-C*H*$_3$), 0.42 (d, $^3J_{HH}$ = 6.7 Hz, 3H, Dipp-iPr-C*H*$_3$), 0.35 (d, $^3J_{HH}$ = 7.4 Hz, 3H, Si-iPr-C*H*$_3$), -0.27 (d, $^3J_{HH}$ = 6.6 Hz, 3H, Si-iPr-C*H*$_3$).

^{13}C{^{1}H} NMR (101 MHz, C_6D_6): δ = 148.73, 146.97, 146.05, 145.46, 145.22, 135.25 (Ph-*C*H), 135.12 (Dipp-^{m}*C*), 132.83 (d, J_{PC} = 14.7 Hz, Dipp-^{m}*C*), 132.22 (d, J_{PC} = 13.9 Hz, Dipp-^{p}*C*), 129.35 (Ph-*C*H), 129.08 (d, J_{PC} = 8.1 Hz, Ph-*C*H), 128.45 (Ph-*C*H), 128.27 (d, J_{PC} = 3.3 Hz, Ph-*C*H), 128.17 (d, J_{PC} = 4.0 Hz, Ph-*C*H), 127.26 (Ph-*C*H), 125.00 (Ph-*C*H), 124.43 (Ph-*C*H), 123.71 (Ph-*C*H), 123.47 (d, *J* = 6.2 Hz, (Ph-*C*H), 41.27 (Si-iPr-*C*H$_3$), 29.47 (Dipp-iPr-*C*H$_3$), 29.06 (Dipp-iPr-*C*H$_3$), 28.54 (Dipp-iPr-*C*H),

28.28 (Dipp-iPr-*C*H), 27.70 (Dipp-iPr-*C*H_3, Si-iPr-*C*H_3), 27.45 (Dipp-iPr-*C*H_3, Si-iPr-*C*H_3), 27.17 (Dipp-iPr-*C*H), 26.05 (Dipp-iPr-*C*H), 25.81 (Dipp-iPr-*C*H_3), 24.78 (Si-iPr-*C*H_3), 23.95 (Si-iPr-*C*H_3), 23.08 (Dipp-iPr-*C*H_3), 22.74, 22.60, 22.43 (Dipp-iPr-*C*H_3), 20.50, 19.92 (d, J_{PC} = 7.7 Hz), 19.12, 18.61, 18.38 (Si-iPr-*C*H), 18.27 (Dipp-iPr-*C*H_3), 15.19 (d, J_{PC} = 8.1 Hz), 14.18, 13.81 (d, J_{PC} = 8.1 Hz), 11.30.

^{29}Si{^{1}H} NMR (79 MHz, C_6D_6): δ = 9.36 (d, $^2J_{SiP}$ = 3.4 Hz, CH_2-*Si*-(iPr)$_2$), 6.18 (d, $^2J_{SiP}$ = 7.8 Hz, CH_2-*Si*-(iPr)$_2$).

^{31}P{^{1}H} NMR (162 MHz, C_6D_6): δ = 35.77 (d, $^2J_{PP}$ = 28.4 Hz), 21.67 (d, $^2J_{PP}$ = 28.4 Hz).

λ_{max}, nm (ε, $Lmol^{-1}$ cm^{-1}): 335 (27067), 581 (2710).

MS/LIFDI-HRMS found (calcd.) m/z: 1122.4213 (1122.4260) $[M]^+$

Anal. calcd. $C_{62}H_{86}Ge_2N_2NiP_2Si_2$: C, 63.03%; H, 7.34%; N, 2.37%; found: C, 63.47%; H, 7.75%; N, 2.27%.

[(^{Ph}LSn)$_2$·Ni], 4.

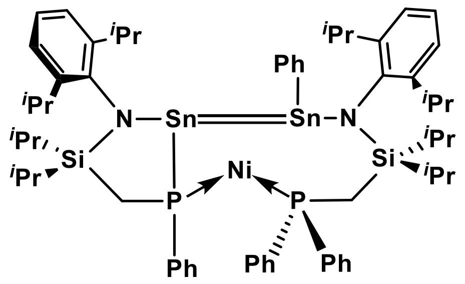

^{PhiP}DippSnH (150 mg, 247 µmol, 2.0 eq.) and Ni(cod)$_2$ (33.9 mg, 123 µmol, 1.0 eq.) were each placed in a Schlenk flask and toluene (15 mL) was added to both. The ^{PhiP}DippSnH was cooled to -78 °C and the Ni(cod)$_2$ solution was added dropwise. The suspension was allowed to warm to room temperature overnight with continuous stirring. Subsequently all volatiles were removed *in vacuo* and pentane (10 mL) added for crystallization. Concentrating and storing the solution overnight at -35 °C, yielded compound **14** as dark green crystals (144 mg, 113 µmol, 92%).

^{1}H NMR (400 MHz, C_6D_6): δ = δ 8.17 (dddd, $^3J_{HH}$ = 10.2, 8.3, 3.3, 1.8 Hz, 2H, Dipp-^{p}C*H*), 8.06–7.91 (m, 4H, Dipp-^{m}C*H*), 7.23 (dddd, $^3J_{HH}$ = 12.3, 10.4, 8.3, 6.5 Hz, 9H, Ph-C*H*), 7.15–7.02 (m, 9H, Ph-C*H*), 6.95 (dd, $^3J_{HH}$ = 7.7, 1.7 Hz, 1H, Ph-C*H*), 4.20 (hept, $^3J_{HH}$ = 7.0 Hz, 1H, Dipp-iPr-C*H*), 3.85 (hept, $^3J_{HH}$ = 6.8 Hz, 1H, Dipp-iPr-C*H*), 3.50 (hept, $^3J_{HH}$ = 6.8 Hz, 1H, Dipp-iPr-C*H*), 2.77 (hept, $^3J_{HH}$ = 6.6 Hz, 1H, Dipp-iPr-C*H*), 2.52 (dd, $^3J_{HH}$ = 13.0, 9.7 Hz, 1H, Si-iPr-C*H*), 2.17–1.93 (m, 1H, Si-iPr-C*H*), 1.93–1.83 (m, 1H, Si-C*H*$_2$-P), 1.76 (d, $^3J_{HH}$ = 6.7 Hz, 3H, Dipp-iPr-C*H*$_3$), 1.66–1.55 (m, 9H, Dipp-iPr-C*H*$_3$), 1.41 (d, $^3J_{HH}$ = 6.8 Hz, 3H, Dipp-iPr-C*H*$_3$), 1.35 (d, $^3J_{HH}$ = 6.9 Hz, 3H, Dipp-iPr-C*H*$_3$), 1.30–1.18 (m, 2H, Si-C*H*$_2$-P), 1.12 (d, $^3J_{HH}$ = 7.7 Hz, 3H, Si-iPr-C*H*$_3$), 1.06 (d, $^3J_{HH}$ = 6.7 Hz, 6H, Dipp-iPr-C*H*$_3$), 1.02 (s, 3H, Si-iPr-C*H*$_3$), 0.90 (d, $^3J_{HH}$ = 7.6 Hz, 3H, Si-iPr-C*H*$_3$), 0.82 (s, 3H, Si-

iPr-CH_3), 0.73 (d, $^3J_{HH}$ = 7.5 Hz, 1H), 0.61 (dd, $^3J_{HH}$ = 17.1, 7.1 Hz, 3H), -0.11 (d, $^3J_{HH}$ = 6.6 Hz, 3H, Si-iPr-CH_3).

^{13}C{^{1}H} NMR (101 MHz, C_6D_6): δ = 159.66, 147.60, 146.57, 145.35, 145.19, 135.74 (Ph-*C*H), 135.04 (d, J_{PC} = 12.1 Hz, Dipp-^{m}*C*), 133.19 (d, J_{PC} = 14.3 Hz, Dipp-^{m}*C*), 131.94 (d, J_{PC} = 13.6 Hz, Dipp-^{p}*C*), 129.86 (d, J_{PC} = 2.2 Hz, Ph-*C*H), 129.04 (d, J_{PC} = 2.2 Hz, Ph-*C*H), 128.75 (Ph-*C*H), 128.40 (d, J_{PC} = 2.6 Hz, Ph-*C*H), 128.25 (dt, J_{PC} = 9.9, 3.7 Hz, Ph-*C*H), 124.25 (Ph-*C*H), 124.01 (Ph-*C*H), 123.73 (Ph-*C*H), 123.31 (d, J_{PC} = 8.1 Hz, Ph-*C*H), 122.89 (Ph-*C*H), 29.01 (Dipp-iPr-*C*H_3), 28.24 (d, J_{PC} = 5.5 Hz, Dipp-iPr-*C*H), 27.58 (Dipp-iPr-*C*H), 27.30 (Dipp-iPr-*C*H), 26.91 (Dipp-iPr-*C*H_3), 25.49 (Si-iPr-*C*H_3), 24.76 (Si-iPr-*C*H_3), 23.53 (Dipp-iPr-*C*H_3), 23.22 (Dipp-iPr-*C*H_3), 22.88 (Dipp-iPr-*C*H_3), 22.76 (Dipp-iPr-*C*H_3), 22.59 (Dipp-iPr-*C*H_3), 20.57 (t, J_{PC} = 4.11 Hz, Dipp-iPr-*C*H_3), 20.44 (Si-iPr-*C*H_3), 20.12 (Si-*C*H_2-P), 19.86 (Si-iPr-*C*H), 19.06 (Si-iPr-*C*H), 18.88 (Si-iPr-*C*H_3), 18.17 (d, J_{PC} = 3.3 Hz, Si-iPr-*C*H_3), 15.40 (d, *J* = 7.3 Hz, Si-iPr-*C*H_3), 13.57 (d, J_{PC} = 7.7 Hz, Si-iPr-*C*H_3), 11.52 (d, J_{PC} = 9.5 Hz, Si-iPr-*C*H).

^{29}Si{^{1}H} NMR (79 MHz, C_6D_6): δ = 6.42 (d, $^2J_{SiP}$ = 2.4 Hz, CH_2-*Si*-(iPr)$_2$), 5.33 (d, $^2J_{SiP}$ = 7.3 Hz, CH_2-*Si*-(iPr)$_2$).

^{31}P{^{1}H} NMR (162 MHz, C_6D_6): δ = 44.27 (d, $^2J_{PP}$ = 49.9 Hz), 27.04 (d, $^2J_{PP}$ = 49.9 Hz).

^{119}Sn NMR (149 MHz, C_6D_6): 791.89 (d, $^2J_{SnP}$ = 769.0 Hz), -777.96 (d, $^2J_{SnP}$ = 1098.6 Hz).

λ_{max}, nm (ε, Lmol^{-1} cm^{-1}): 429 (9764), 489 (6478), 646 (2773).

MS/LIFDI-HRMS found (calcd.) m/z: 1272.3255 (1272.3204) [M]$^+$

Anal. calcd. $C_{62}H_{86}N_2NiP_2Si_2Sn_2$: C, 58.47%; H, 6.81%; N, 2.20%; found: C, 58.94%; H, 6.96%; N, 2.20%.

[(^{Cy}LSn)$_2$·Ni], 5.

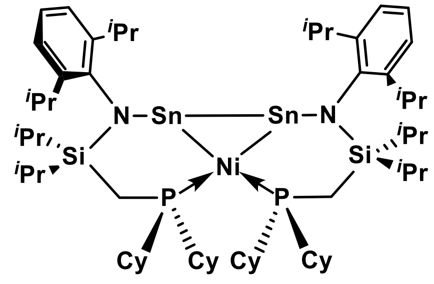

^{CyiP}DippSnH (1.00 g, 1.61 mmol, 2.0 eq.) and Ni(cod)$_2$ (542 mg, 756 µmol, 1.0 eq.) were each placed in a Schlenk flask and toluene (40 mL) was added to both. The ^{CyiP}DippSnH was cooled to -78 °C and the Ni(cod)$_2$ solution was added dropwise. After stirring for two hours, all volatiles were removed *in vacuo* and the residue was extracted with heptane. Concentrating and storing the solution overnight at -35 °C, yielded compound **11** as dark brown crystals (417 mg, 321 µmol, 39%).

^{1}H NMR (400 MHz, C_6D_6): δ = 7.27–7.17 (m, 4H, Dipp-^{m}C*H*) 7.17–7.08 (m, 2H, Dipp-^{p}C*H*), 4.01 (hept, $^{3}J_{HH}$ = 6.9 Hz, 2H, Dipp-iPr-C*H*), 3.80 (hept, $^{3}J_{HH}$ = 7.0 Hz, 2H, Dipp-iPr-C*H*), 2.47 (h, $^{3}J_{HH}$ = 6.9 Hz, 2H, Si-iPr-C*H*), 2.25 (d, $^{3}J_{HH}$ = 13.4 Hz, 2H, Si-iPr-C*H*), 2.11 (d, $^{3}J_{HH}$ = 6.5 Hz, 4H, Cy-*H*), 1.94–1.75 (m, 14H, Cy-*H*), 1.70–1.52 (m, 7H, Cy-*H*), 1.49 (d, $^{3}J_{HH}$ = 6.8 Hz, 6H, Dipp-iPr-C*H*$_3$), 1.44 (d, $^{3}J_{HH}$ = 6.8 Hz, 6H, Dipp-iPr-C*H*$_3$), 1.41–1.37 (m, 4H, Cy-*H*), 1.34 (d, $^{3}J_{HH}$ = 7.3 Hz, 12H, Dipp-iPr-C*H*$_3$), 1.33–1.23 (m, 6H, Cy-*H*), 1.22 (d, $^{3}J_{HH}$ = 7.2 Hz, 12H, Si-iPr-C*H*$_3$), 1.19–1.16 (m, 3H, Cy-*H*), 1.14 (d, $^{3}J_{HH}$ = 6.7 Hz, 6H, Si-iPr-C*H*$_3$), 1.11 (d, $^{3}J_{HH}$ = 6.8 Hz, 6H, Si-iPr-C*H*$_3$), 0.87 (d, $^{3}J_{HH}$ = 5.5 Hz, 6H, Si-C*H*$_2$-P).

^{13}C{^{1}H} NMR (101 MHz, C_6D_6): δ = 154.87 (Dipp-^{ipso}*C*), 147.10 (Dipp-^{o}*C*), 144.48 (Dipp-^{o}*C*), 124.31 (Dipp-^{m}*C*), 123.40 (Dipp-^{p}*C*), 43.84 (t, J_{PC} = 7.2 Hz, Cy-*C*H), 42.88 (t, J_{PC} = 7.2 Hz, Cy-*C*H), 34.48 (d, $^{3}J_{PC}$ = 7.3 Hz, Si-iPr-*C*H), 34.05 (Si-iPr-*C*H), 32.01 (Cy-*C*H), 30.82 (Cy-*C*H), 29.41 (Cy-*C*H), 28.82 (Dipp-iPr-*C*H), 28.08 (t, J_{PC} = 4.8 Hz, Si-iPr-C*H*$_3$), 27.79 (t, J_{PC} = 6.6 Hz, Si-iPr-C*H*$_3$), 27.65 (Cy-*C*H), 27.17 (Dipp-iPr-*C*H), 26.78 (Si-iPr-*C*H$_3$), 26.66 (Dipp-iPr-*C*H), 24.21 (Dipp-iPr-*C*H$_3$), 23.38 (Dipp-iPr-*C*H$_3$), 22.73 (Dipp-iPr-*C*H$_3$), 20.65 (Si-iPr-C*H*$_3$), 19.66 (Si-iPr-C*H*$_3$), 19.21 (Dipp-iPr-*C*H$_3$), 18.78 (Dipp-iPr-*C*H$_3$), 17.81 (Si-*C*H$_2$-P), 16.01 (Si-iPr-*C*H), 14.28, 11.83 (Si-iPr-*C*H$_3$).

^{29}Si{^{1}H} NMR (79 MHz, C_6D_6): δ = 4.89 (s, CH$_2$-*Si*-(iPr)$_2$).

^{31}P{^{1}H} NMR (162 MHz, C_6D_6): δ = 22.86 (s, *P*-Cy$_2$).

^{119}Sn NMR (112 MHz, C_6D_6): *not observed*

λ_{max}, nm (ε, Lmol^{-1} cm^{-1}): 379 (17658), 519 (5340).

MS/LIFDI-HRMS found (calcd.) m/z: 1296.5137 (1296.5082) [M]$^{+}$

Anal. calcd. $C_{62}H_{110}N_2NiP_2Si_2Sn_2$: C, 57.38%; H, 8.54%; N, 2.16%; found: C, 57.68%; H, 8.76%; N, 2.17%.

[(^{Cy}LPb)$_2$·Ni], 6.

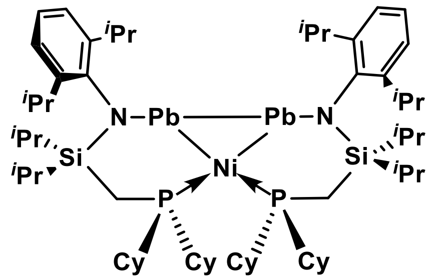

^{CyiP}DippPb vinyl (200 mg, 272 µmol, 2.0 eq.) and Ni(cod)$_2$ (37.4 mg, 136 µmol, 1.0 eq.) were each placed in a Schlenk flask and toluene (15 mL) was added to both. The ^{CyiP}DippPb vinyl was cooled to -78 °C and the Ni(cod)$_2$ solution was added dropwise. After stirring for one hour, all volatiles were

removed *in vacuo* and the residue was extracted with pentane. Concentrating and storing the solution overnight at -35 °C, yielded compound **12** as dark brown crystals (27 mg, 18.2 μmol,12%).

^{1}H NMR (400 MHz, C_6D_6): δ = 7.38 (dd, $^3J_{HH}$ = 7.6, 1.8 Hz, 2H, Dipp-^{m}C*H*), 7.28 (dd, $^3J_{HH}$ = 7.6, 1.8 Hz, 2H, Dipp-^{m}C*H*), 6.96 (t, $^3J_{HH}$ = 7.5 Hz, 2H, Dipp-^{p}C*H*), 3.99 (hept, $^3J_{HH}$ = 6.7 Hz, 2H, Dipp-iPr-C*H*), 3.69 (hept, $^3J_{HH}$ = 6.6 Hz, 2H, Dipp-iPr-C*H*), 2.42 (brs, 2H, Si-iPr-C*H*), 2.23–2.14 (m, 2H, Si-iPr-C*H*), 2.12–1.69 (m, 26H, Cy-*H*), 1.66 (d, $^3J_{HH}$ = 6.9 Hz, 12H, Dipp-iPr-CH_3), 1.62–1.52 (m, 7H, Cy-*H*), 1.51–1.45 (m, 17H, Dipp-iPr-CH_3, Cy-*H*), 1.44–1.34 (m, 7H, Cy-*H*), 1.30 (d, $^3J_{HH}$ = 6.8 Hz, 7H, Si-iPr-CH_3), 1.04 (d, $^3J_{HH}$ = 7.4 Hz, 6H, Si-iPr-CH_3), 0.99 (d, $^3J_{HH}$ = 7.1 Hz, 6H, Si-iPr-CH_3), 0.93 (d, $^3J_{HH}$ = 7.3 Hz, 6H, Si-iPr-CH_3), 0.64 (d, $^3J_{HH}$ = 7.1 Hz, 5H, Si-CH_2-P).

^{13}C{^{1}H} NMR (101 MHz, C_6D_6): δ = 167.06, 149.70 (Dipp-^{ipso}*C*), 145.69 (Dipp-^{o}*C*), 143.65 (Dipp-^{o}*C*), 123.95 (Dipp-^{m}*C*), 123.75 (Dipp-^{m}*C*), 123.50 (Dipp-^{m}*C*), 123.04 (d, J_{PC} = 10.3 Hz, Dipp-^{m}*C*, Dipp-^{p}*C*), 65.13 (Dipp-iPr-*C*H$_3$), 64.60 (Dipp-iPr-*C*H$_3$), 51.82 (Si-iPr-*C*H), 49.39, 40.17, 38.85 (Dipp-iPr-*C*H$_3$), 36.11, 35.94, 33.79 (Dipp-iPr-*C*H$_3$), 31.81 (Cy-*C*H), 31.06 (Cy-*C*H), 30.91 (Cy-*C*H), 29.91 (Cy-*C*H), 29.81 (Cy-*C*H), 29.58 (Cy-*C*H), 29.43 (Cy-*C*H), 28.49 (Cy-*C*H), 28.40 (Cy-*C*H), 28.14 (Dipp-iPr-*C*H), 28.00 (Cy-*C*H), 27.90 (Cy-*C*H), 27.82 (Cy-*C*H), 27.67 (Cy-*C*H), 27.43 (Cy-*C*H), 27.01 (d, J_{PC} = 5.9 Hz), 26.80, 26.46 (Dipp-iPr-*C*H), 25.35 (Cy-*C*H), 24.75 (Dipp-iPr-*C*H$_3$), 24.10 (Si-iPr-*C*H$_3$), 23.16 (Si-iPr-*C*H$_3$), 22.81 (Si-iPr-*C*H$_3$), 21.28 (Si-iPr-*C*H$_3$), 20.93 (Si-iPr-*C*H$_3$), 20.49 (Si-iPr-*C*H$_3$), 19.63, 18.67, 18.65, 18.62, 18.21, 14.76 (d, J_{PC} = 2.2 Hz), 13.97.

^{29}Si{^{1}H} NMR (79 MHz, C_6D_6): δ = 13.57 (s, CH$_2$-*Si*-(iPr)$_2$).

^{31}P{^{1}H} NMR (162 MHz, C_6D_6): δ = 8.67 (s, *P*-Cy$_2$)

λ_{max}, nm (ε, Lmol^{-1} cm^{-1}): 462 (19049).

MS/LIFDI-HRMS found (calcd.) m/z: 1474.6627 (1474.6564) [M]$^+$

Anal. calcd. $C_{62}H_{110}N_2NiP_2Pb_2Si_2$: C, 50.49%; H, 7.52%; N, 1.90%; found: C, 51.20%; H, 7.91%; N, 1.88%.

Printed Spectra

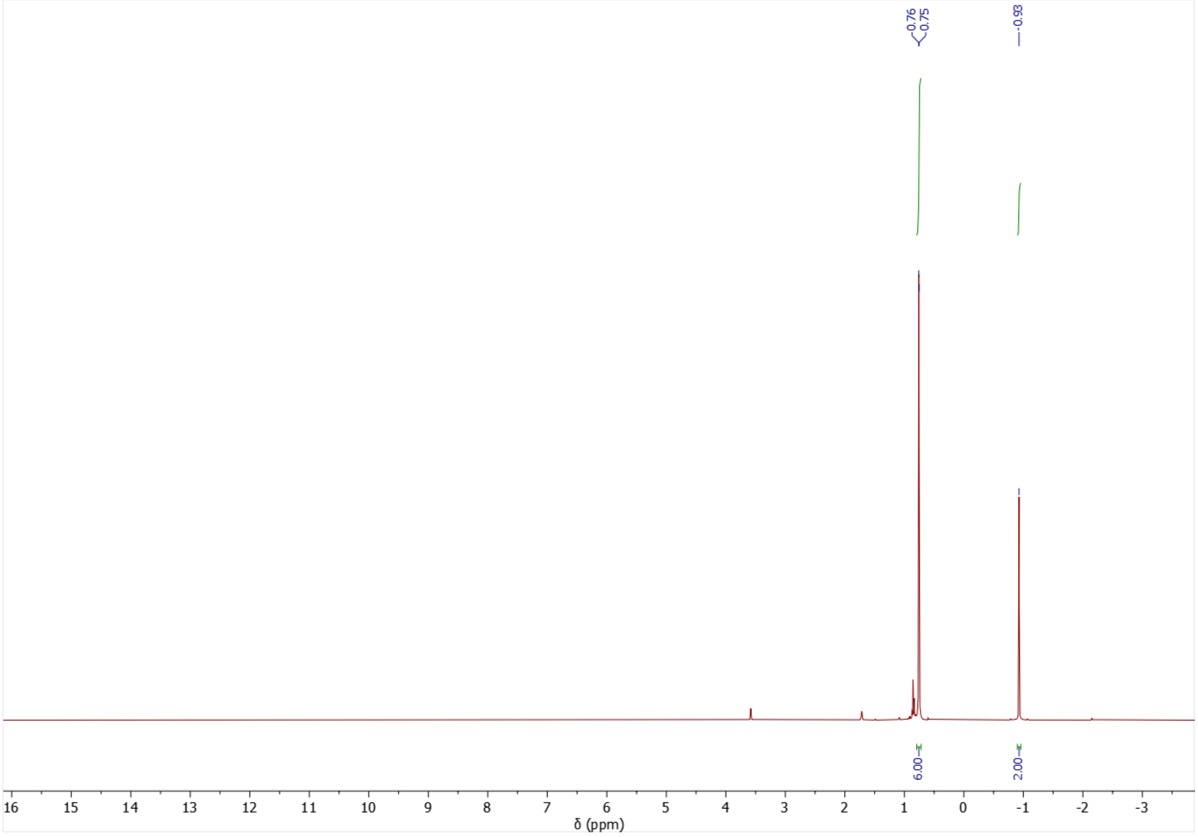

Figure S1. ^{1}H NMR spectrum of compound Me_2PCH_2Li as a solution in THF-d_8 at ambient temperature.

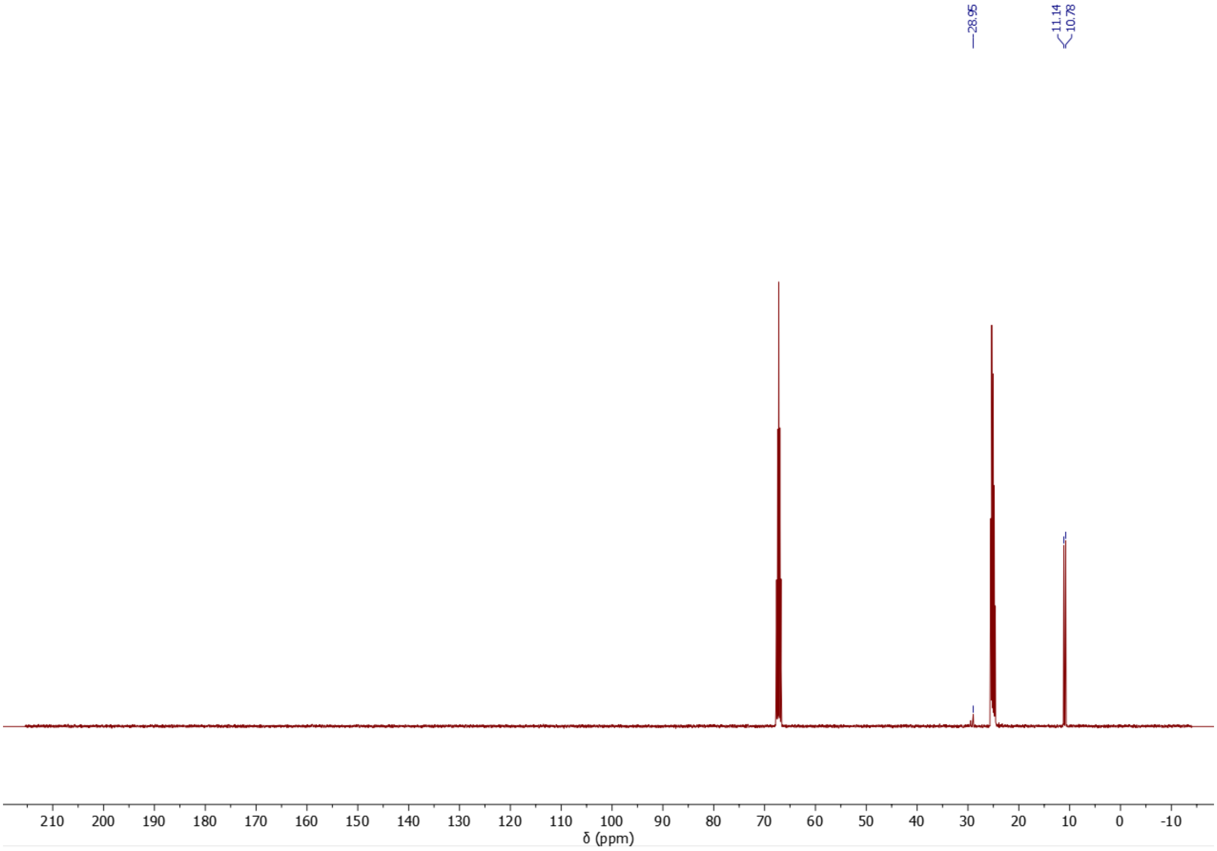

Figure S2. ^{13}C NMR spectrum of compound Me_2PCH_2Li as a solution in THF-d_8 at ambient temperature.

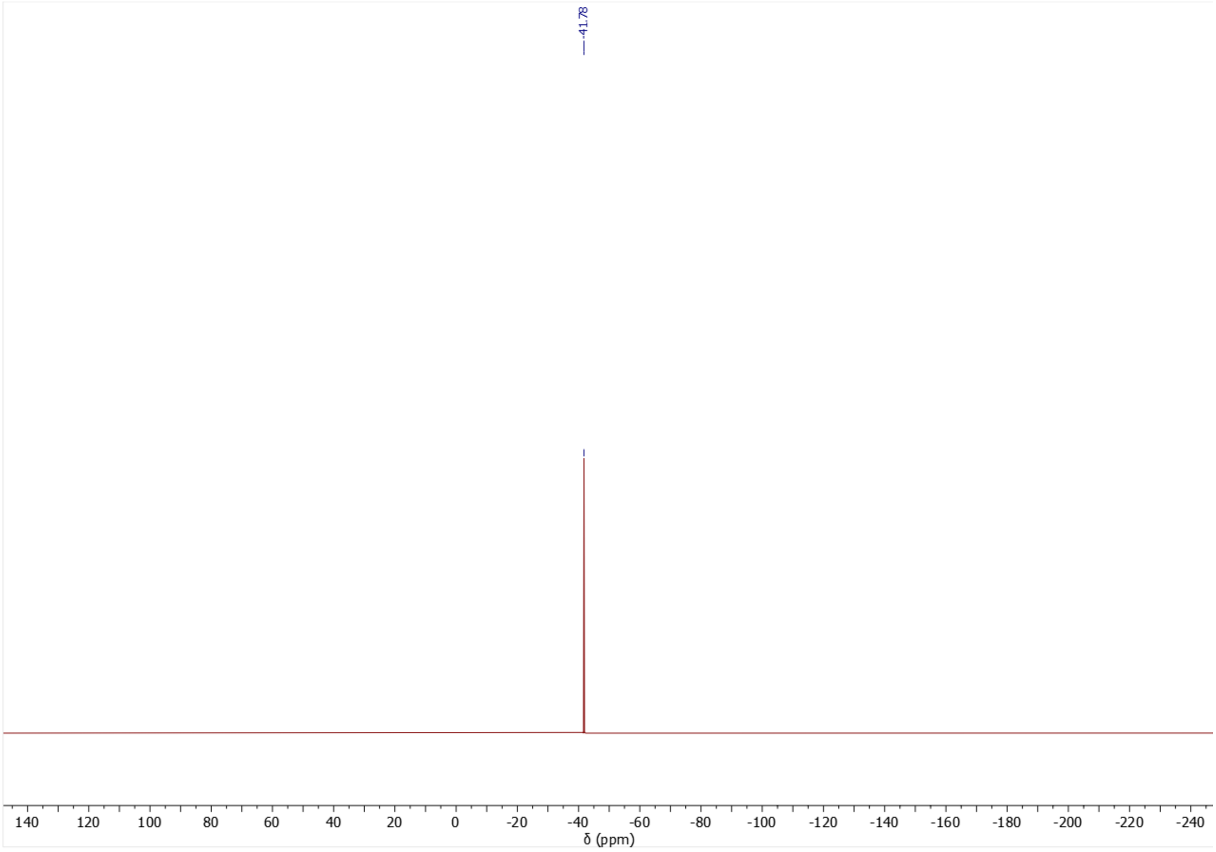

Figure S3. ^{31}P NMR spectrum of compound Me_2PCH_2Li as a solution in THF-d_8 at ambient temperature.

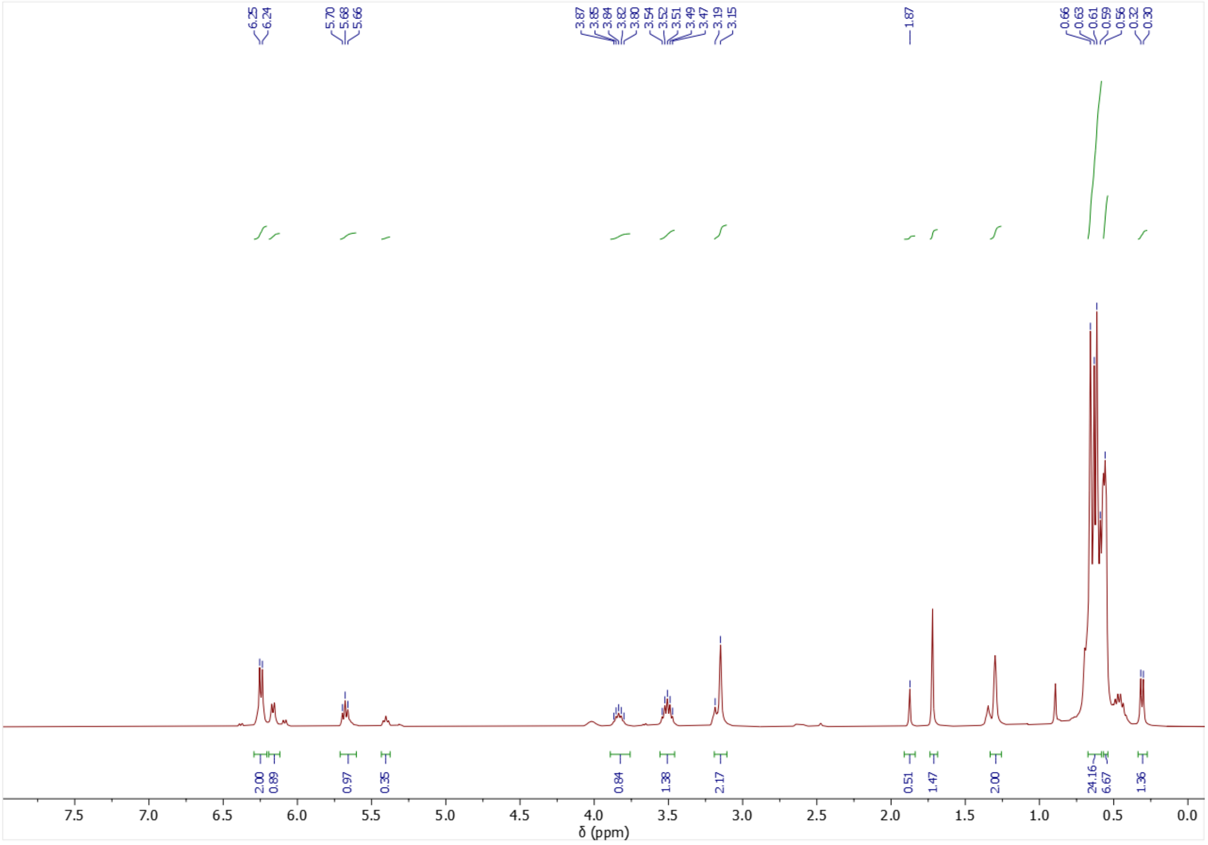

Figure S4. ^{1}H NMR spectrum of compound ^{Me}LK as a solution in THF-d_8 at ambient temperature.

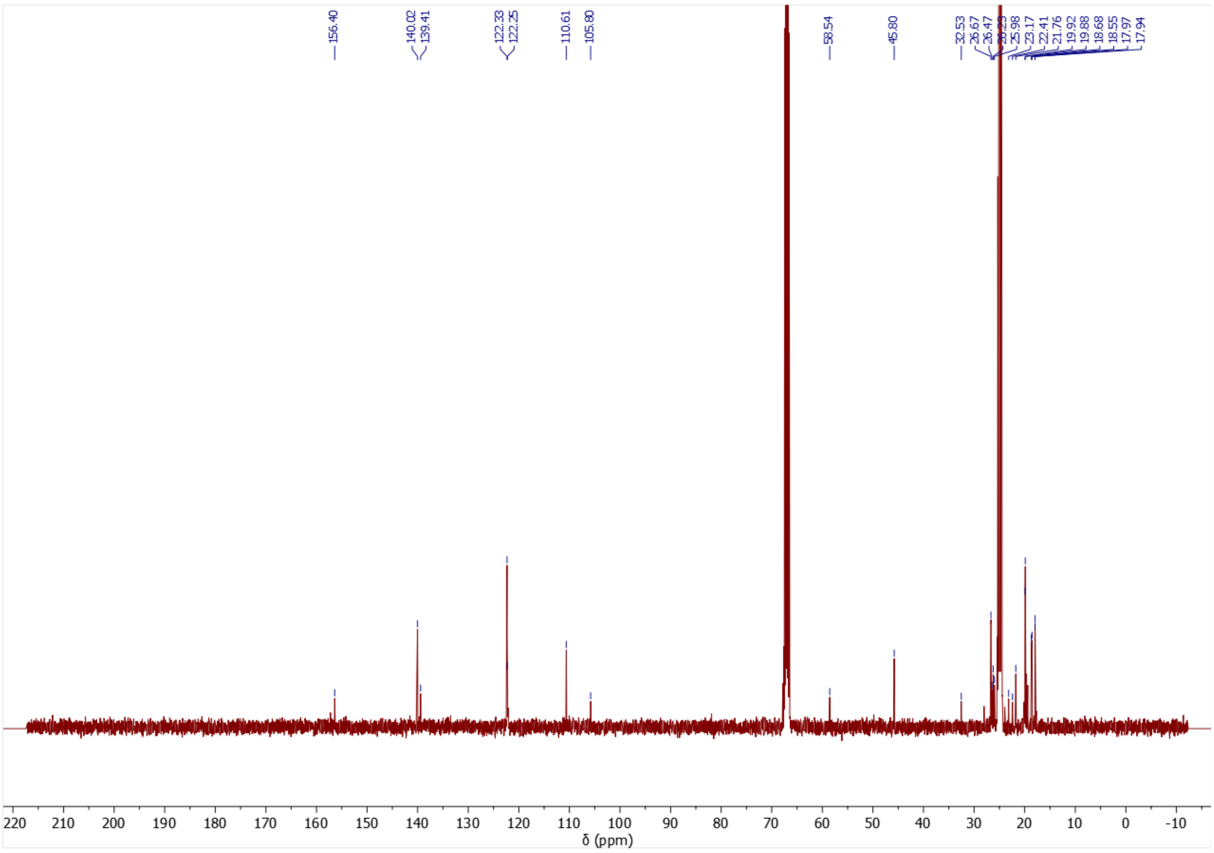

Figure S5. ^{13}C NMR spectrum of compound ^{Me}LK as a solution in THF-d_8 at ambient temperature.

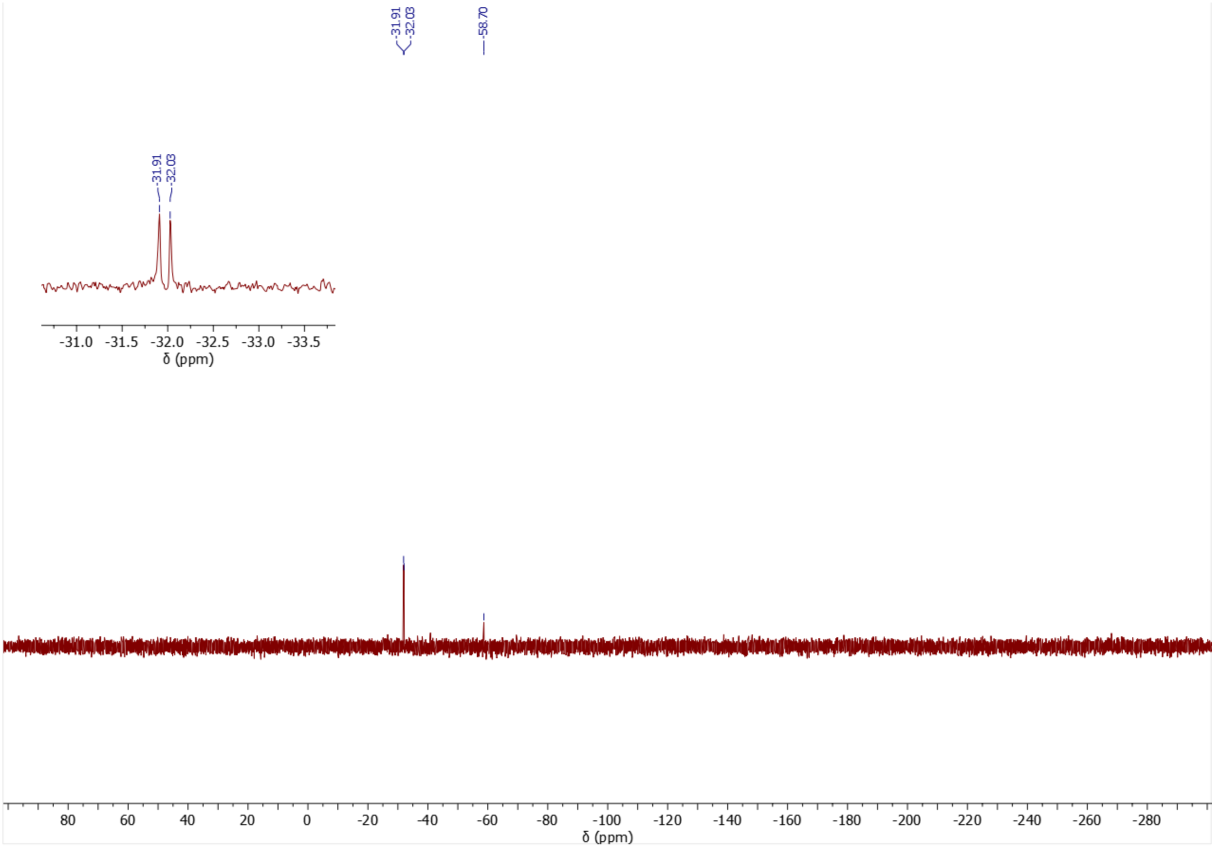

Figure S6. ^{29}Si NMR spectrum of compound ^{Me}LK as a solution in THF-d_8 at ambient temperature.

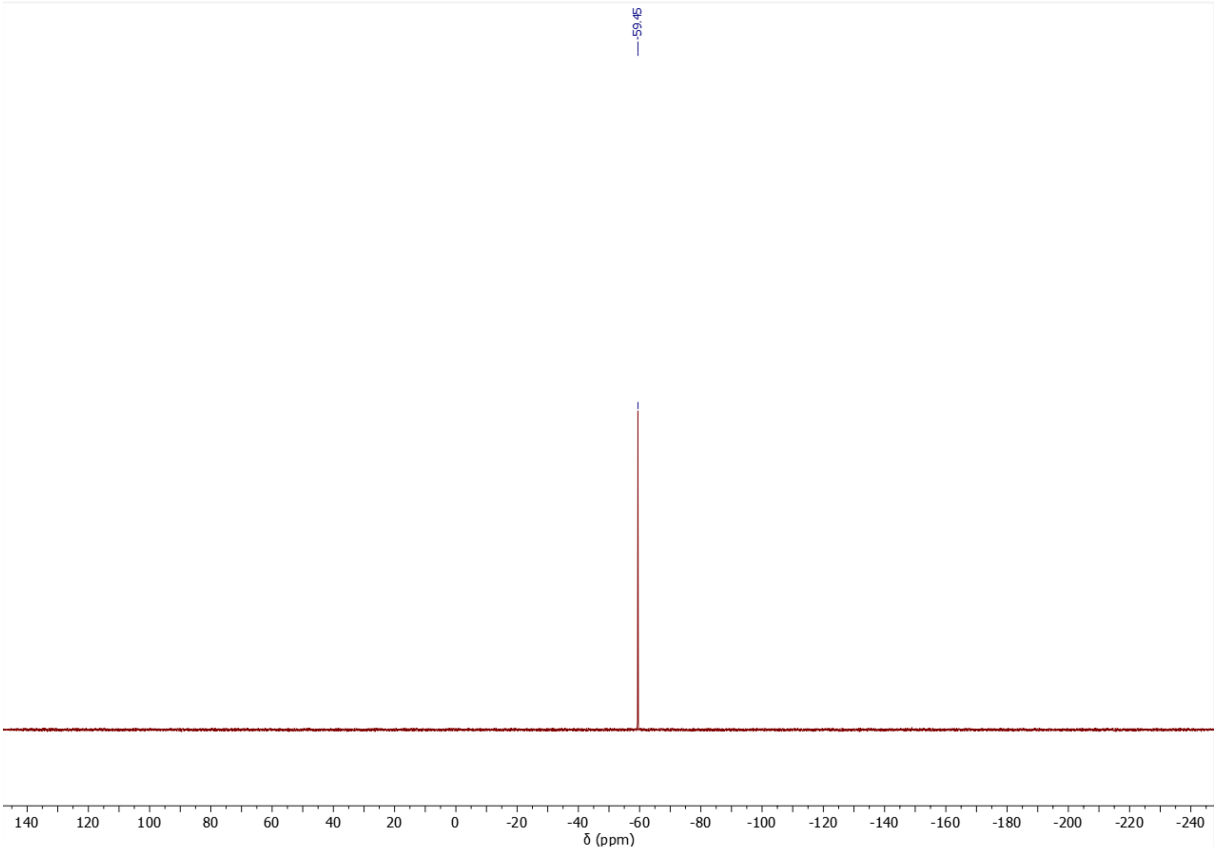

Figure S7. ^{31}P NMR spectrum of compound ^{Me}LK as a solution in THF-d_8 at ambient temperature.

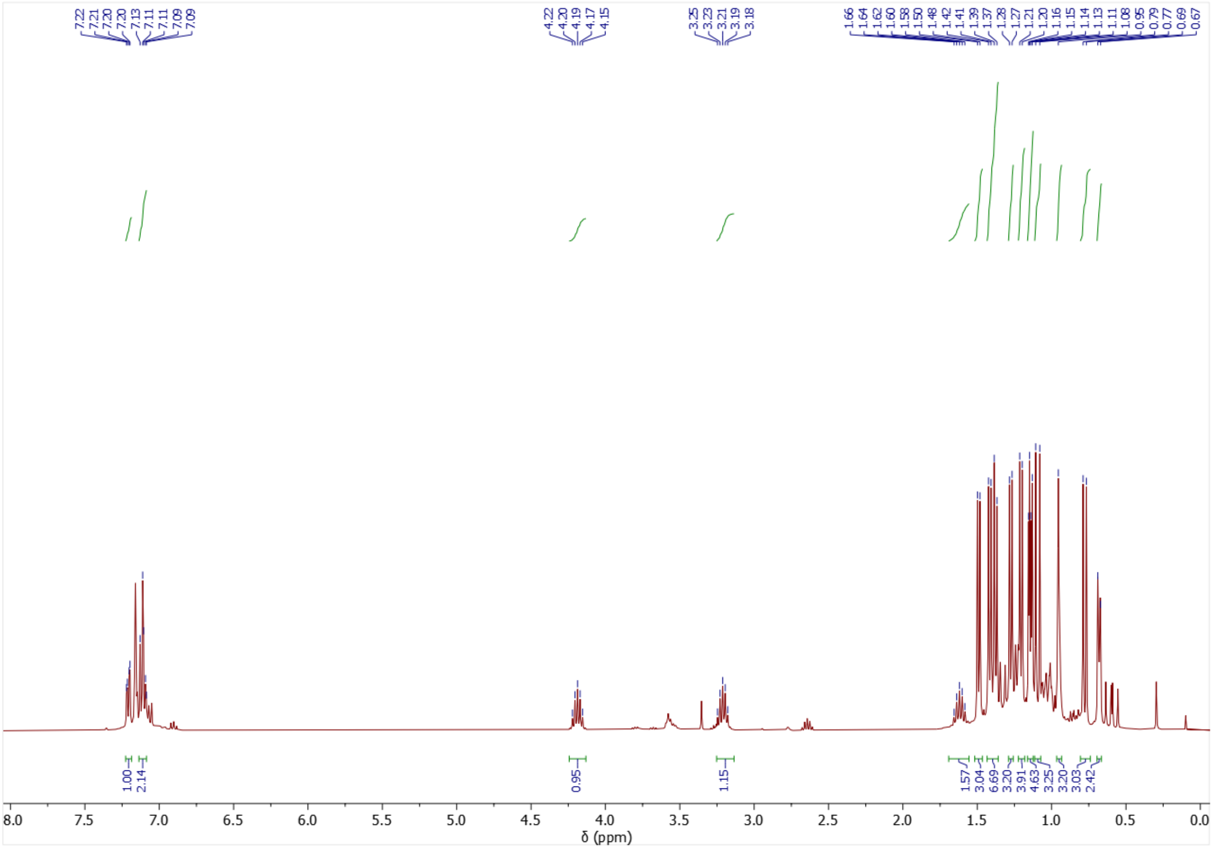

Figure S8. ^{1}H NMR spectrum of compound ^{Me}L(Cl)Ge: as a solution in C_6D_6 at ambient temperature.

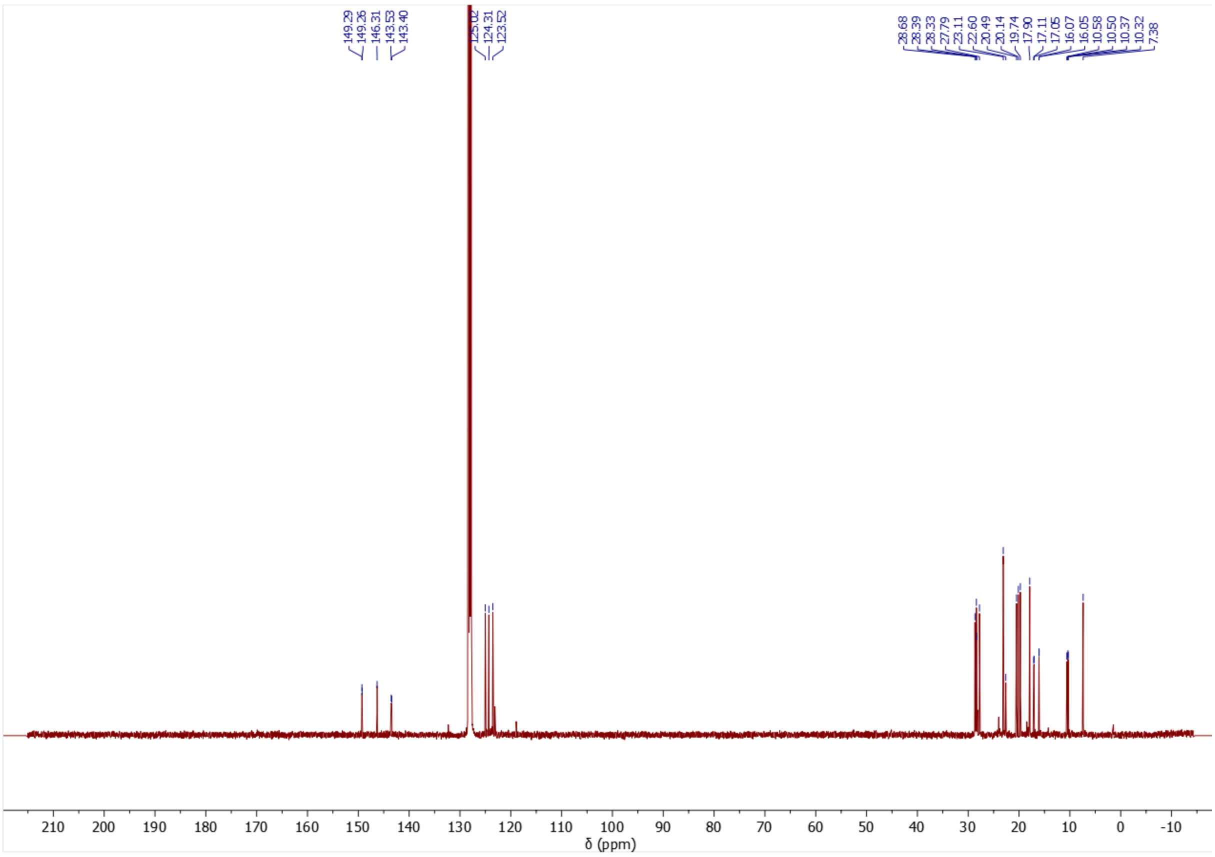

Figure S9. ^{13}C NMR spectrum of compound $^{Me}L(Cl)Ge$: as a solution in C_6D_6 at ambient temperature.

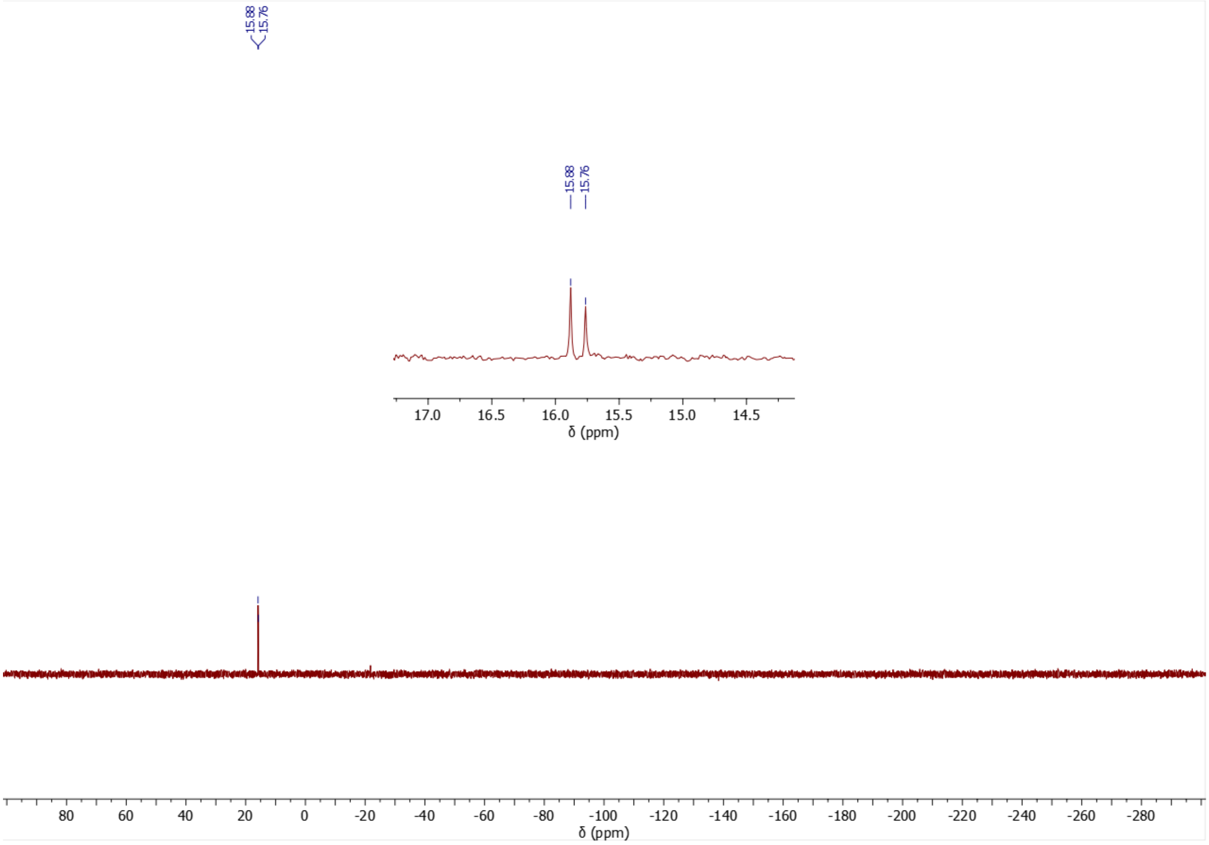

Figure S10. ^{29}Si NMR spectrum of compound $^{Me}L(Cl)Ge$: as a solution in C_6D_6 at ambient temperature.

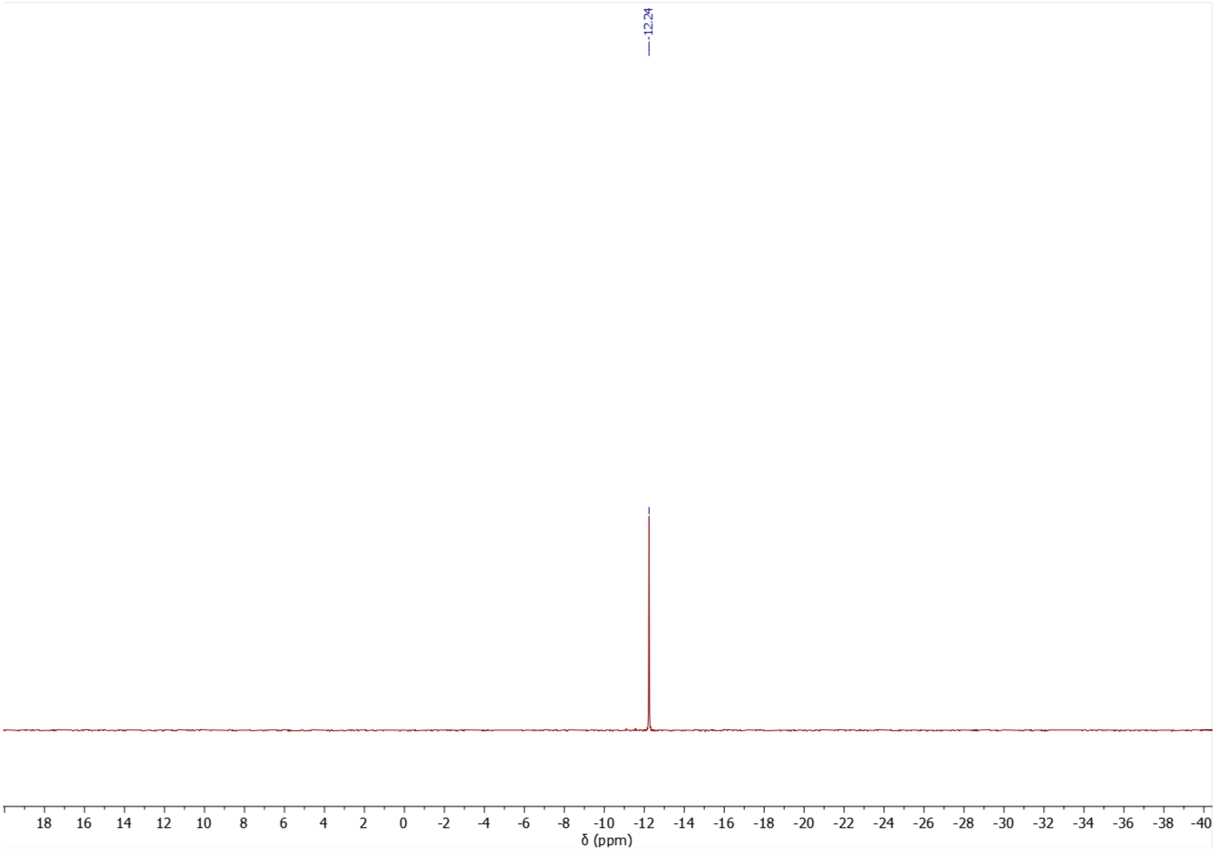

Figure S11. ^{31}P NMR spectrum of compound $^{Me}L(Cl)Ge$: as a solution in C_6D_6 at ambient temperature.

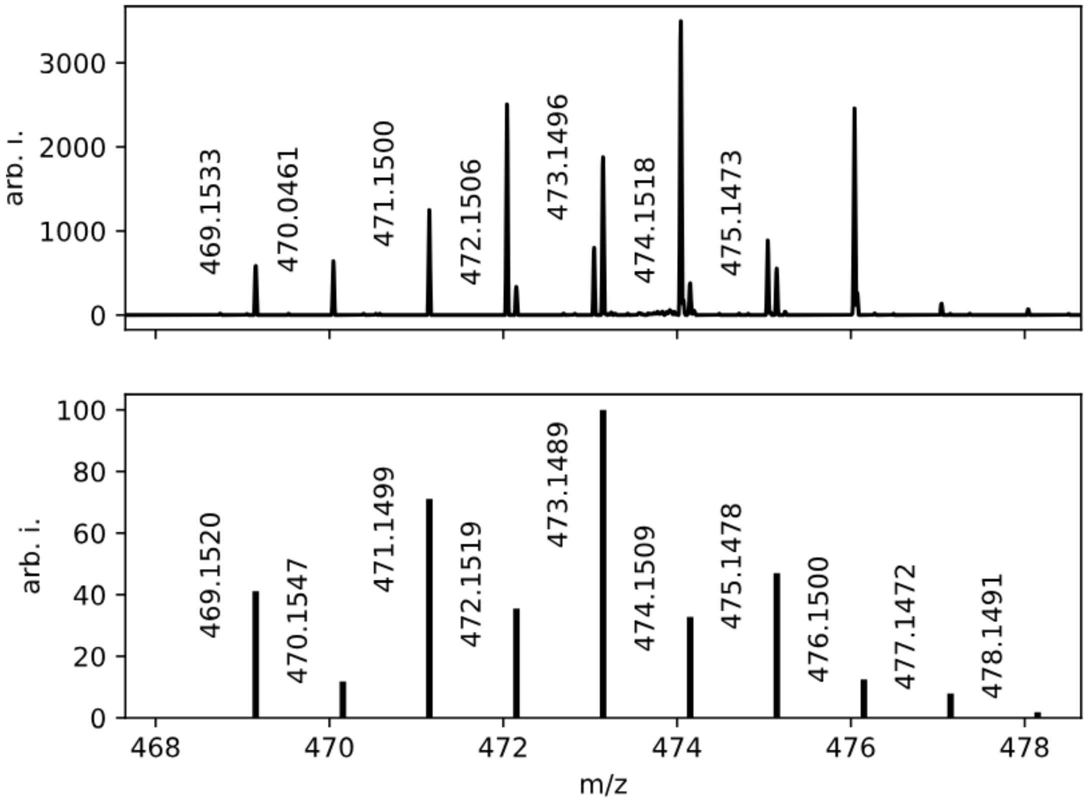

Figure S12. Cutout from LIFDI/MS of compound $^{Me}L(Cl)Ge$:; Top. found MS for $[M]^+$; Bottom. Calculated MS spectrum of $[M]^+$.

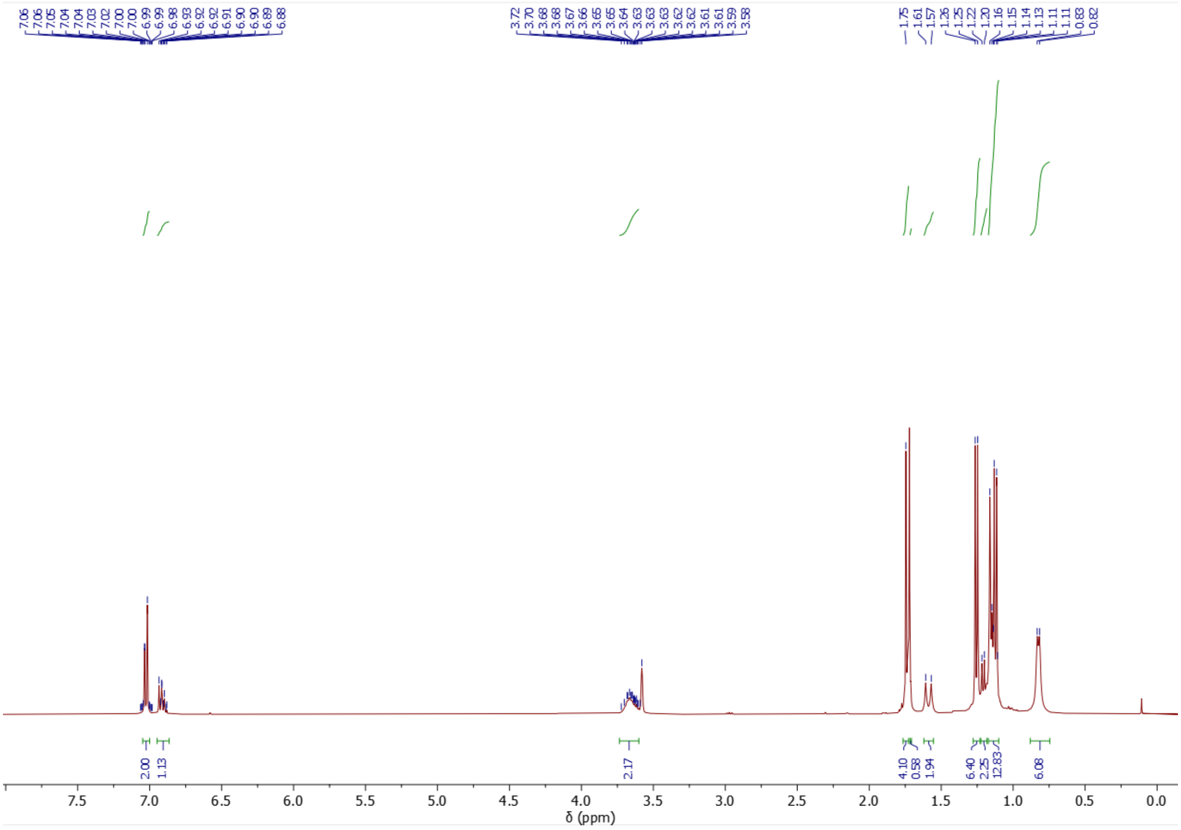

Figure S13. ^{1}H NMR spectrum of compound ^{Me}L(Br)Sn: as a solution in THF-d_8 at ambient temperature.

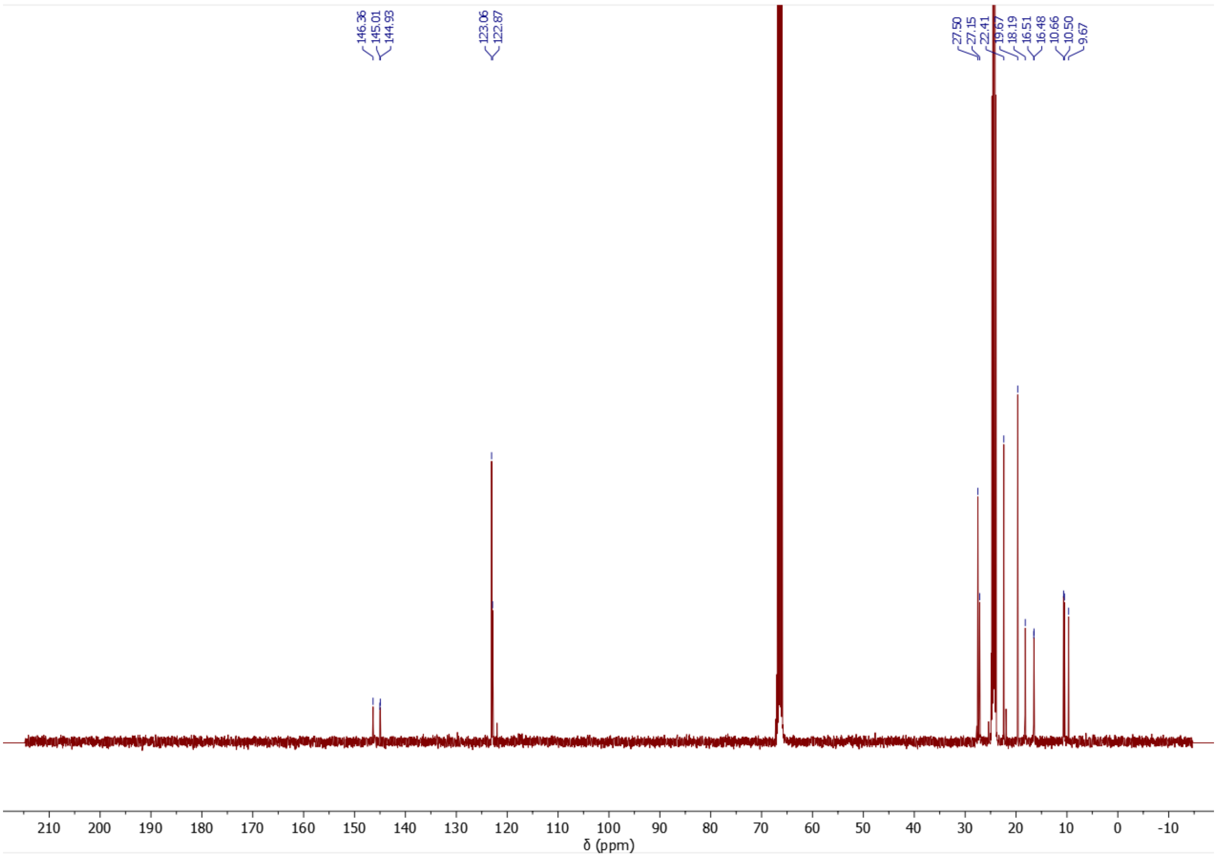

Figure S14. ^{13}C NMR spectrum of compound ^{Me}L(Br)Sn: as a solution in THF-d_8 at ambient temperature.

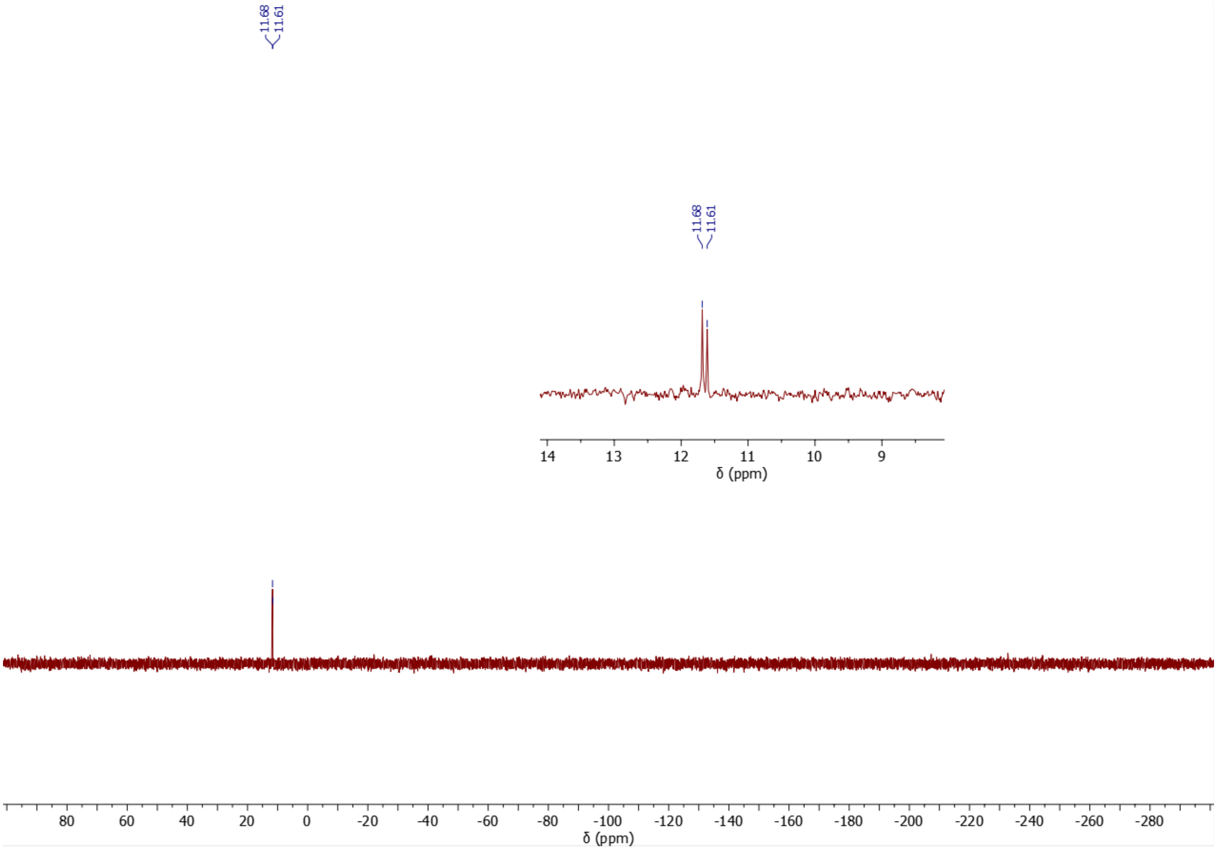

Figure S15. ^{29}Si NMR spectrum of compound ^{Me}L(Br)Sn: as a solution in THF-d_8 at ambient temperature.

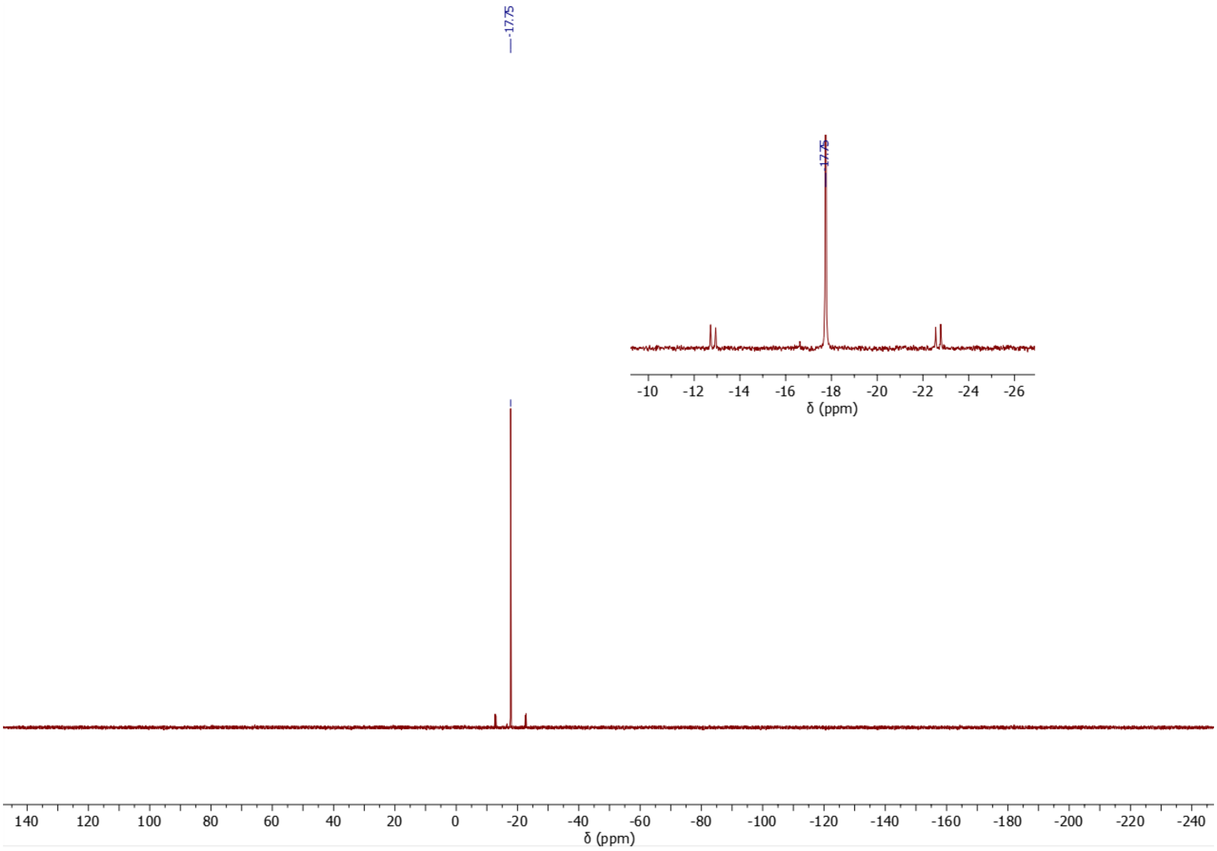

Figure S16. ^{31}P NMR spectrum of compound ^{Me}L(Br)Sn: as a solution in THF-d_8 at ambient temperature.

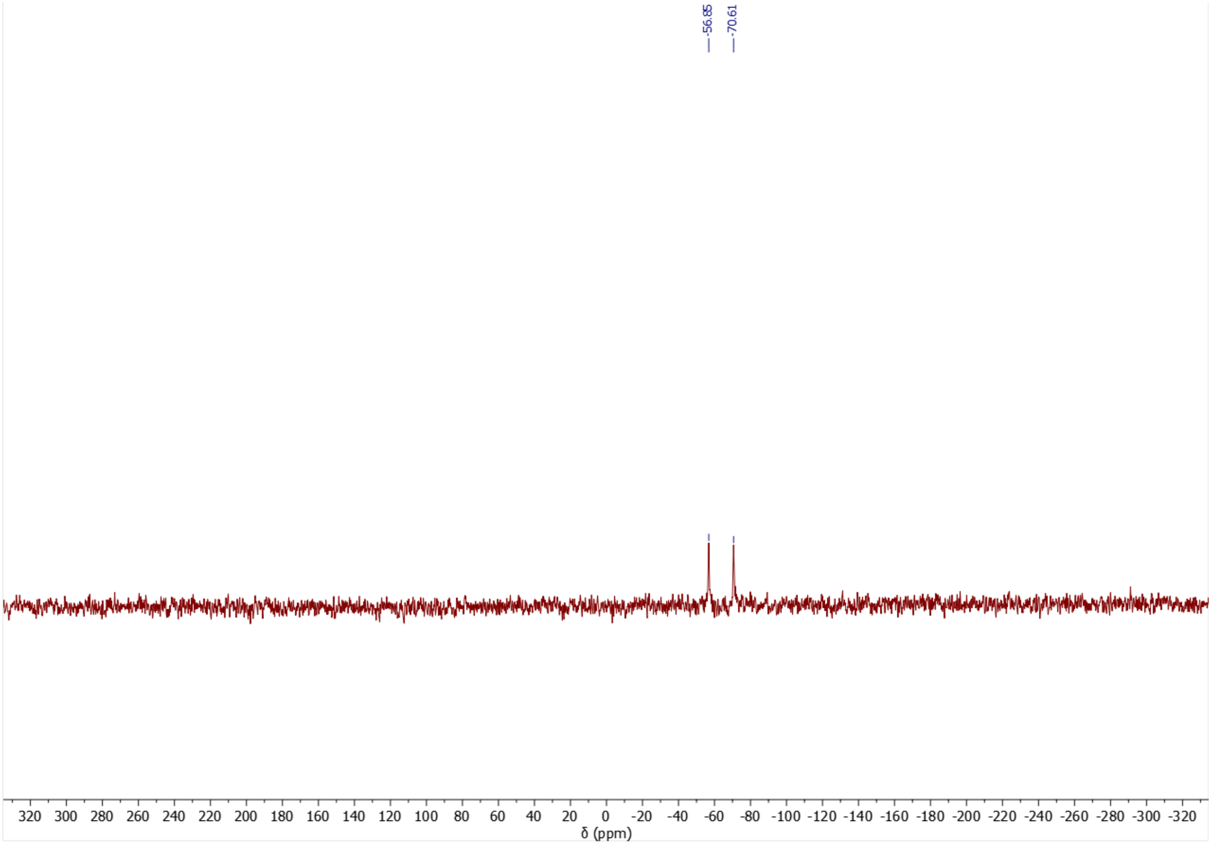

Figure S17. ^{119}Sn NMR spectrum of compound ^{Me}L(Br)Sn: as a solution in C_6D_6 at ambient temperature.

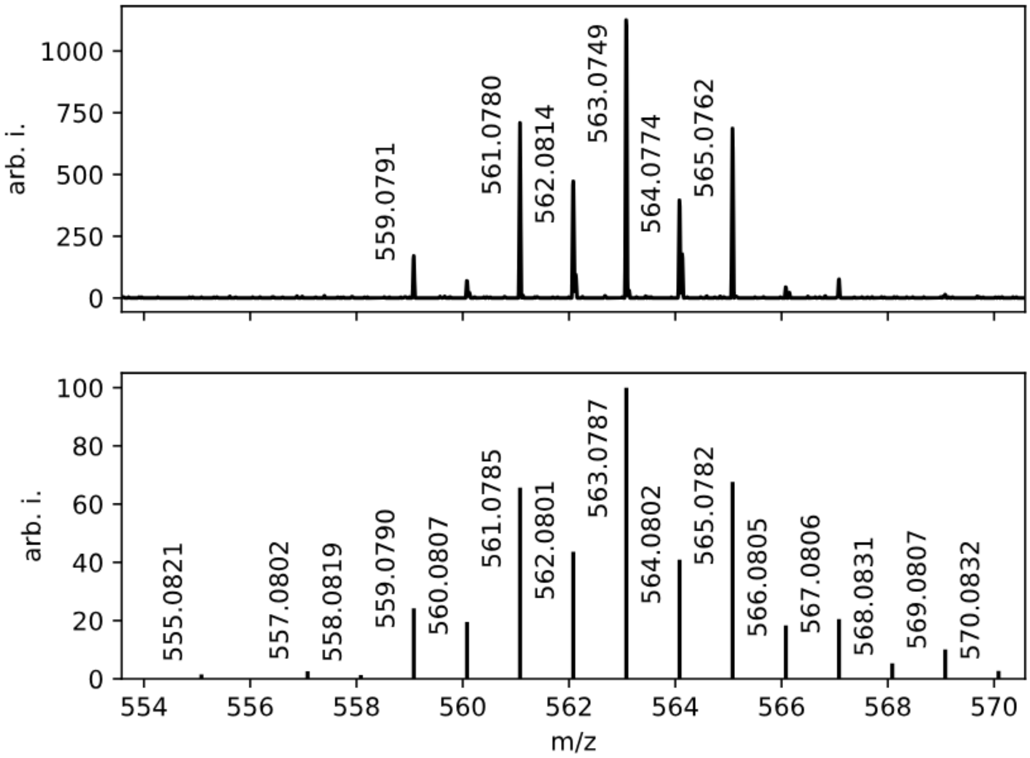

Figure S18. Cutout from LIFDI/MS of compound ^{Me}L(Br)Sn:; Top. found MS for $[M]^+$; Bottom. Calculated MS spectrum of $[M]^+$.

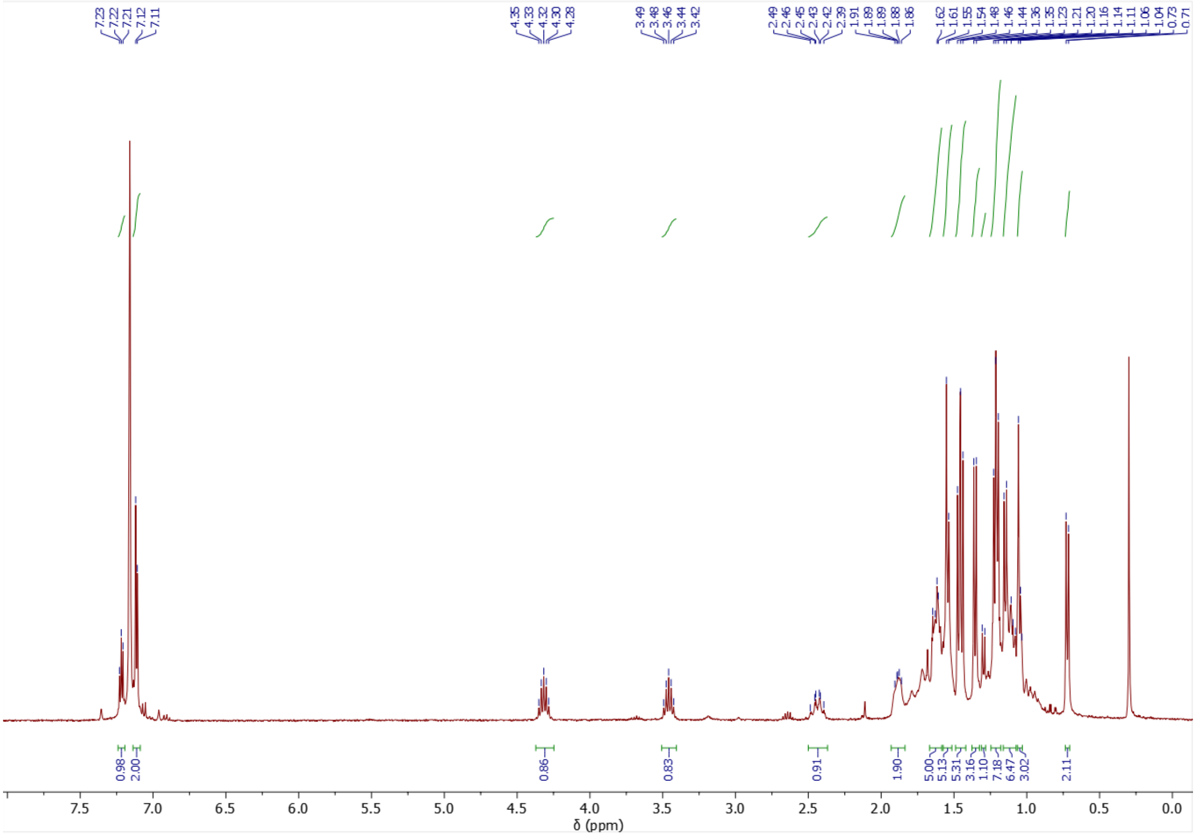

Figure S19. ^{1}H NMR spectrum of compound ^{Cy}L(Br)Sn: as a solution in C_6D_6 at ambient temperature.

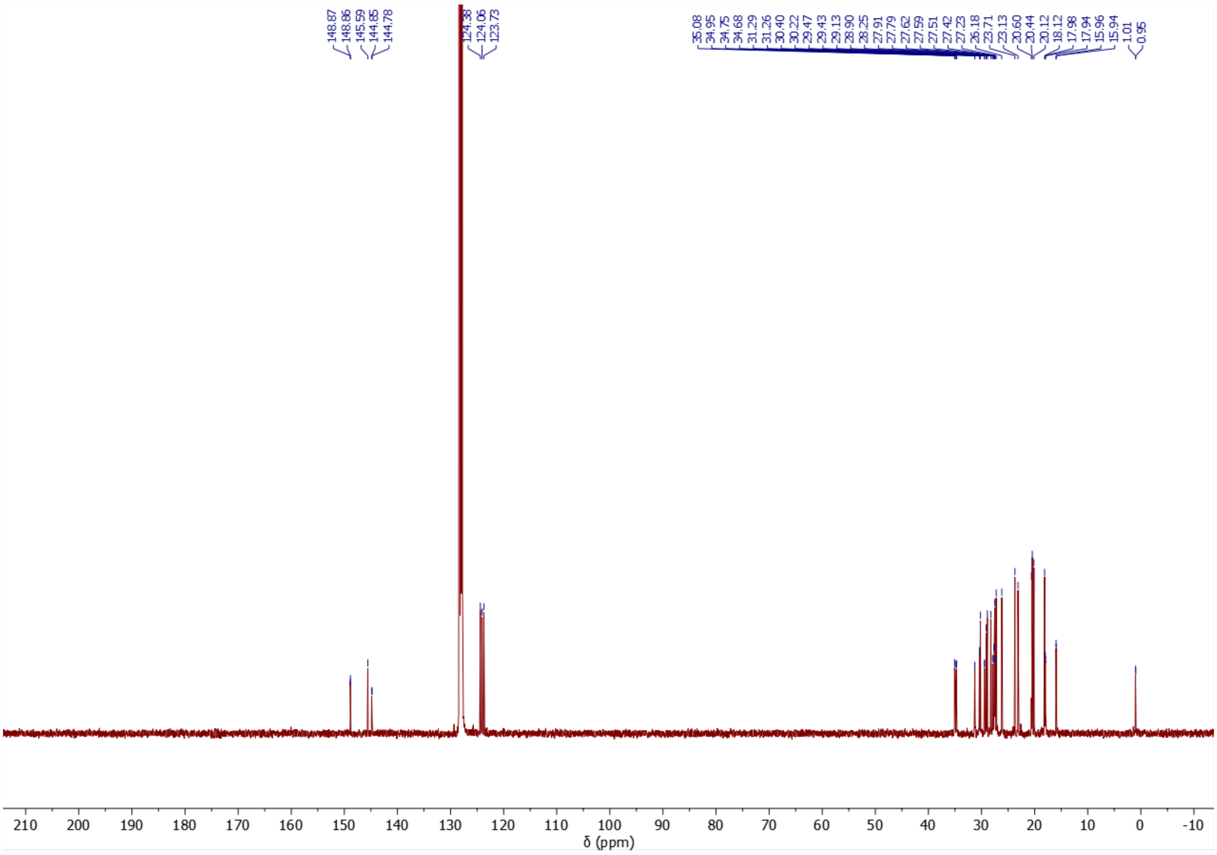

Figure S20. ^{13}C NMR spectrum of compound ^{Cy}L(Br)Sn: as a solution in C_6D_6 at ambient temperature.

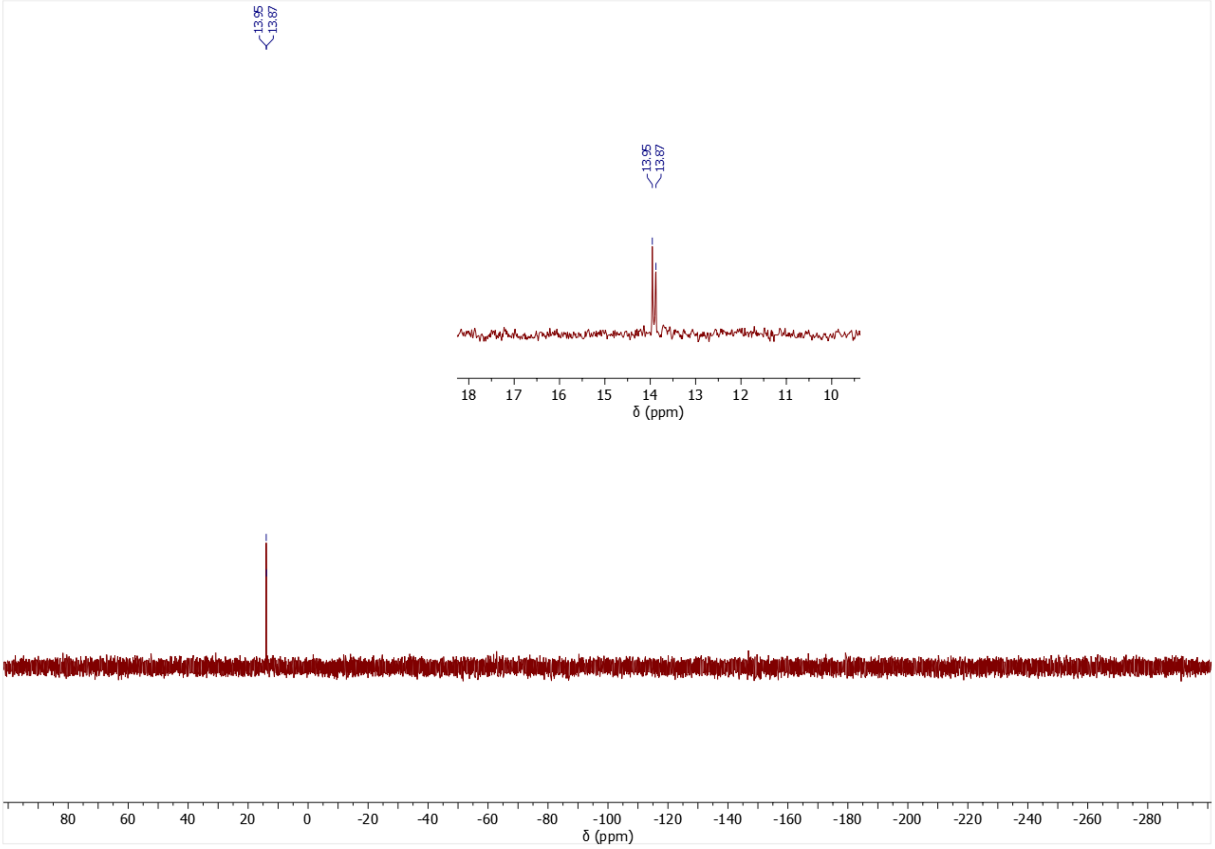

Figure S21. ^{29}Si NMR spectrum of compound $^{Cy}L(Br)Sn$: as a solution in C_6D_6 at ambient temperature.

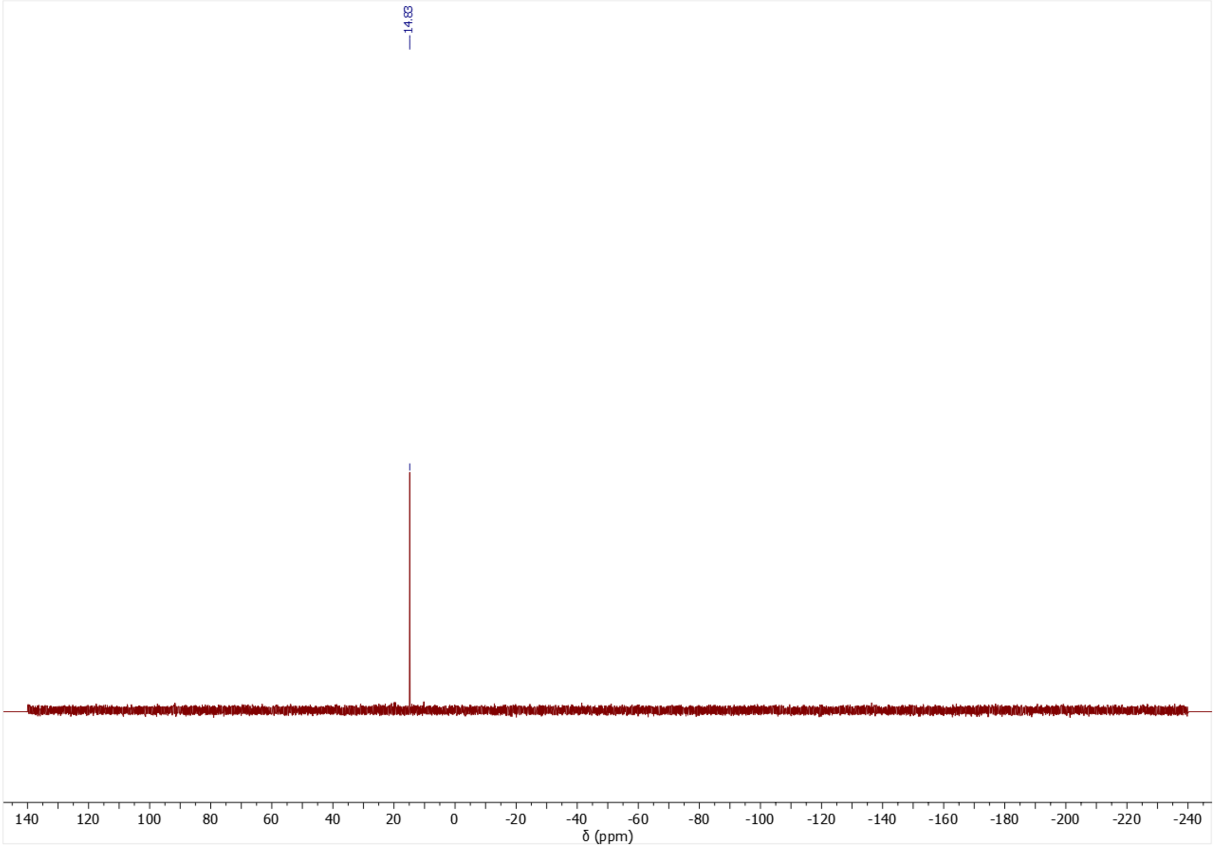

Figure S22. ^{31}P NMR spectrum of compound $^{Cy}L(Br)Sn$: as a solution in C_6D_6 at ambient temperature.

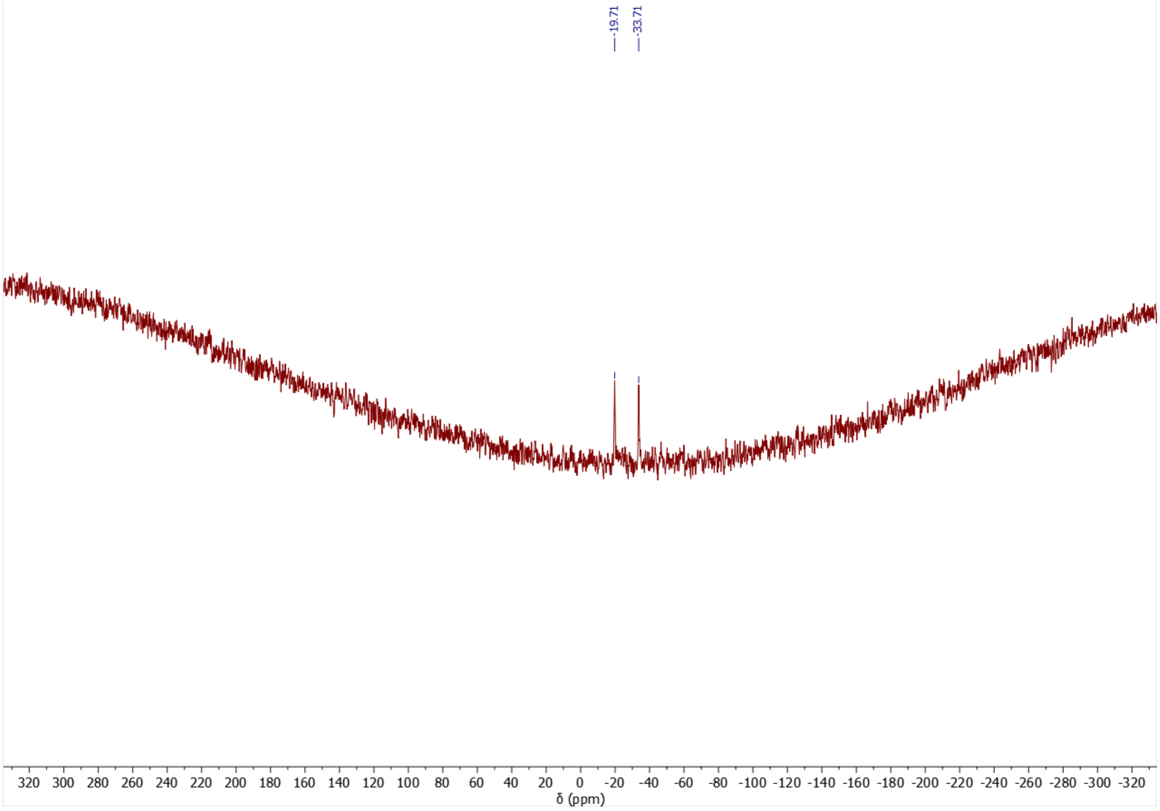

Figure S23. ^{119}Sn NMR spectrum of compound ^{Cy}L(Br)Sn: as a solution in C_6D_6 at ambient temperature.

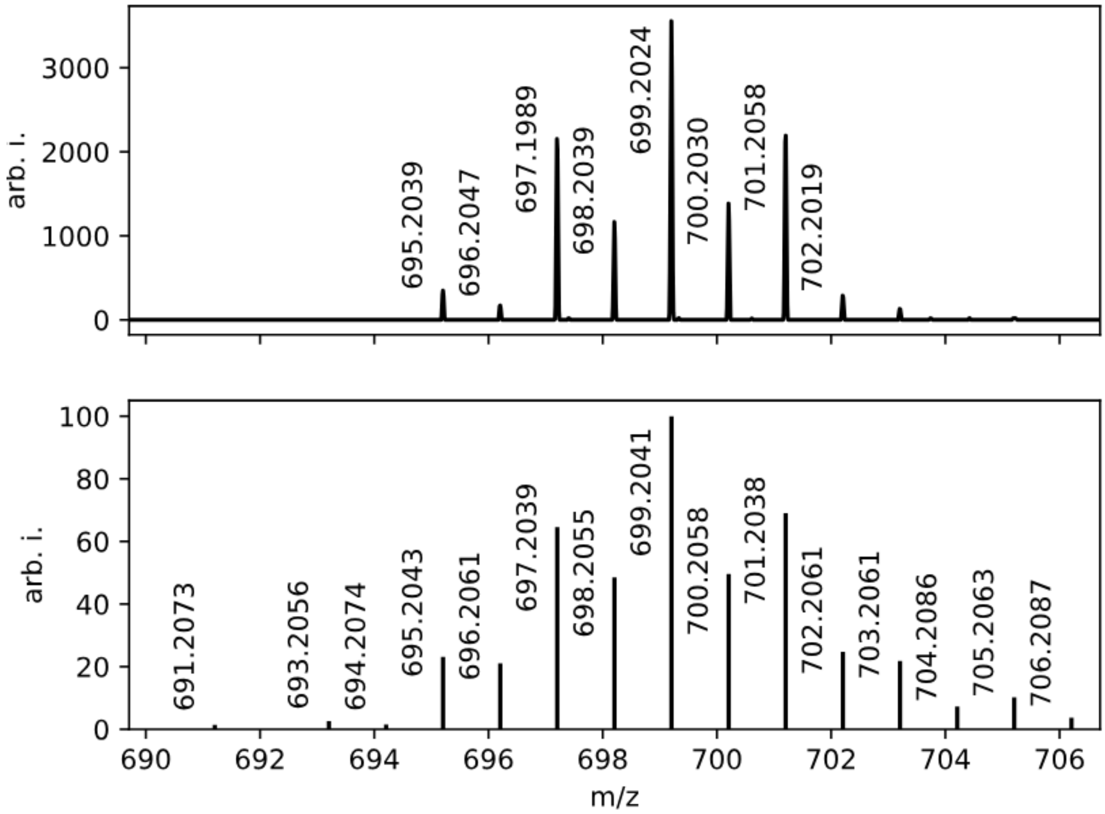

Figure S24. Cutout from LIFDI/MS of compound ^{Cy}L(Br)Sn:; Top. found MS for $[M]^+$; Bottom. Calculated MS spectrum of $[M]^+$.

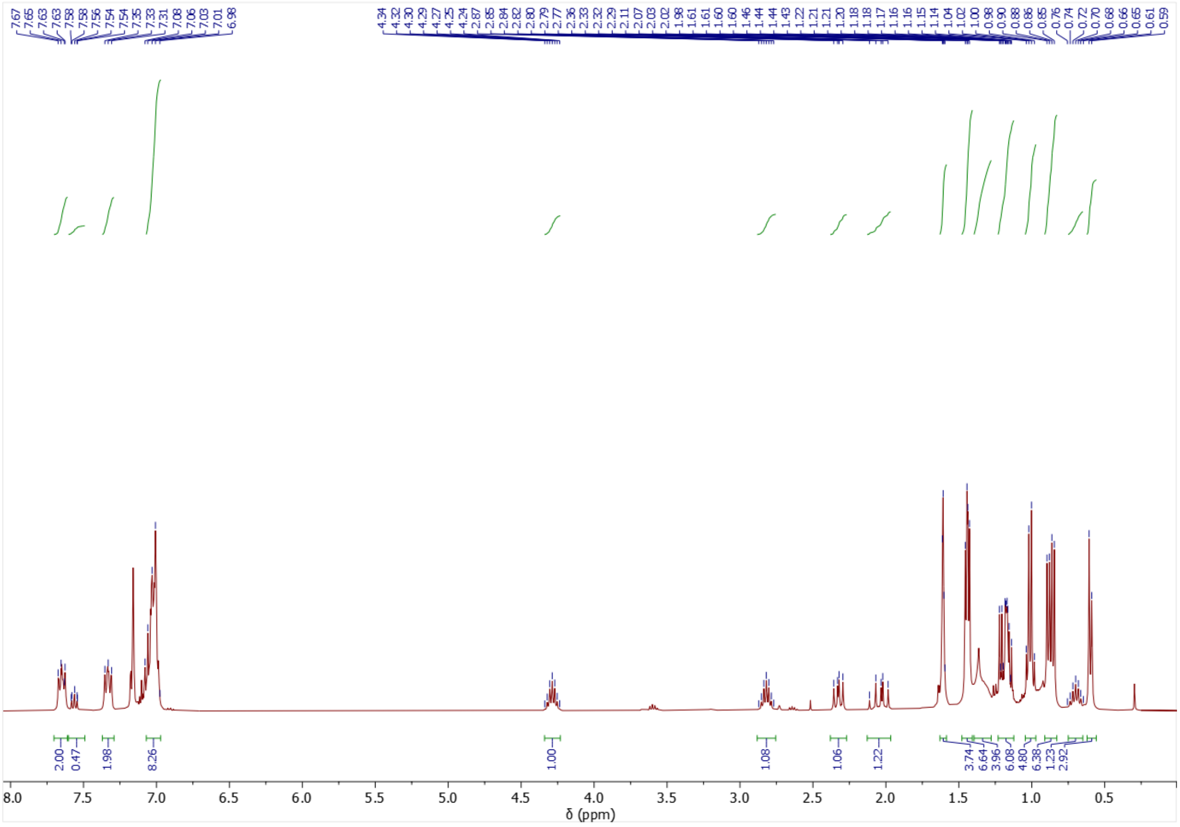

Figure S25. ^{1}H NMR spectrum of compound $^{Ph}L(Br)Sn$: as a solution in C_6D_6 at ambient temperature.

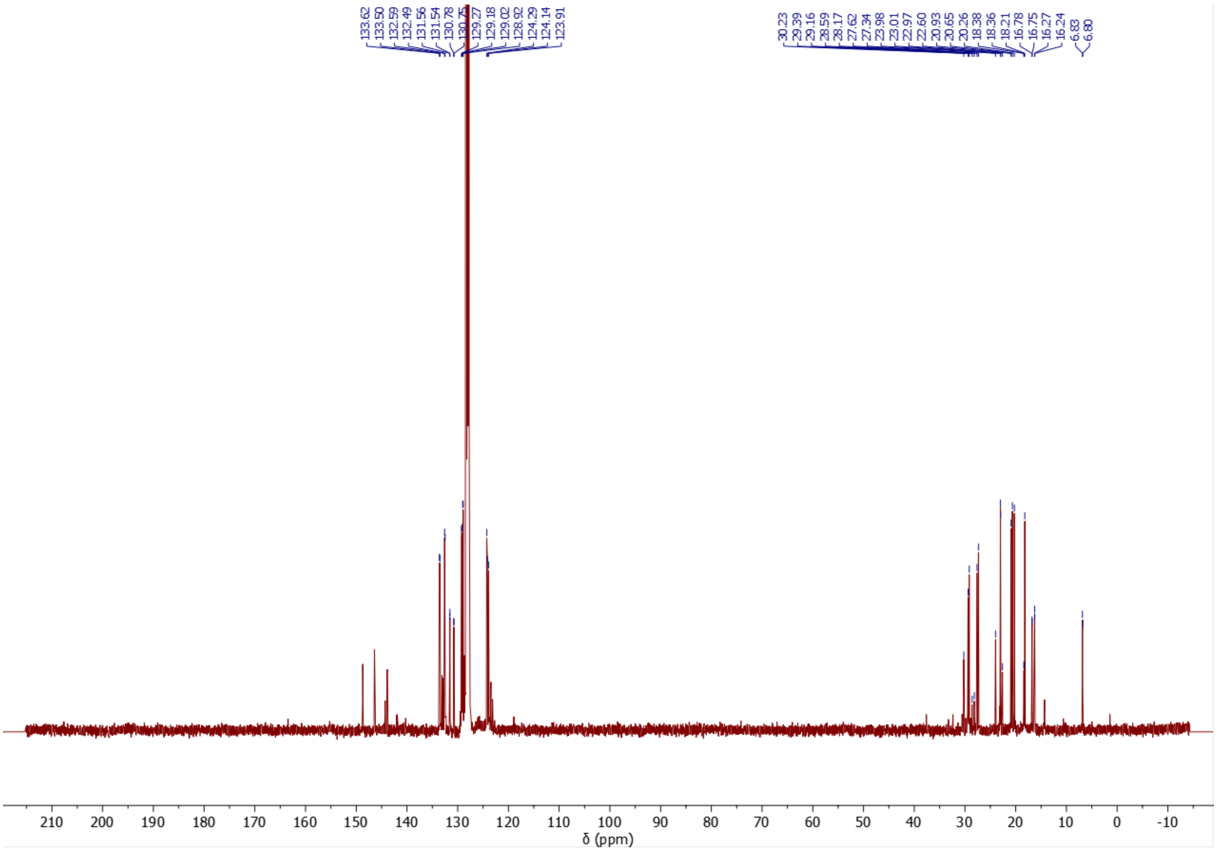

Figure S26. ^{13}C NMR spectrum of compound $^{Ph}L(Br)Sn$: as a solution in C_6D_6 at ambient temperature.

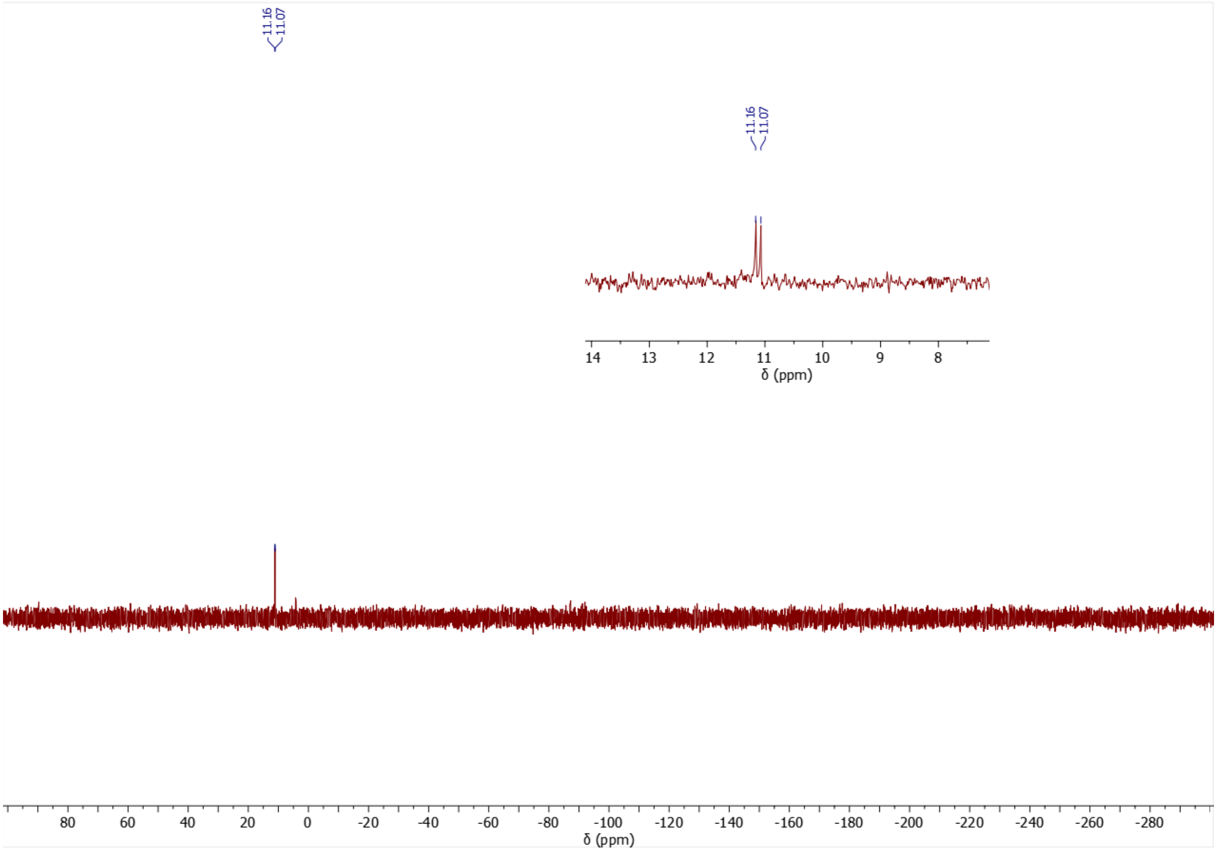

Figure S27. ^{29}Si NMR spectrum of compound ^{Ph}L(Br)Sn: as a solution in C_6D_6 at ambient temperature.

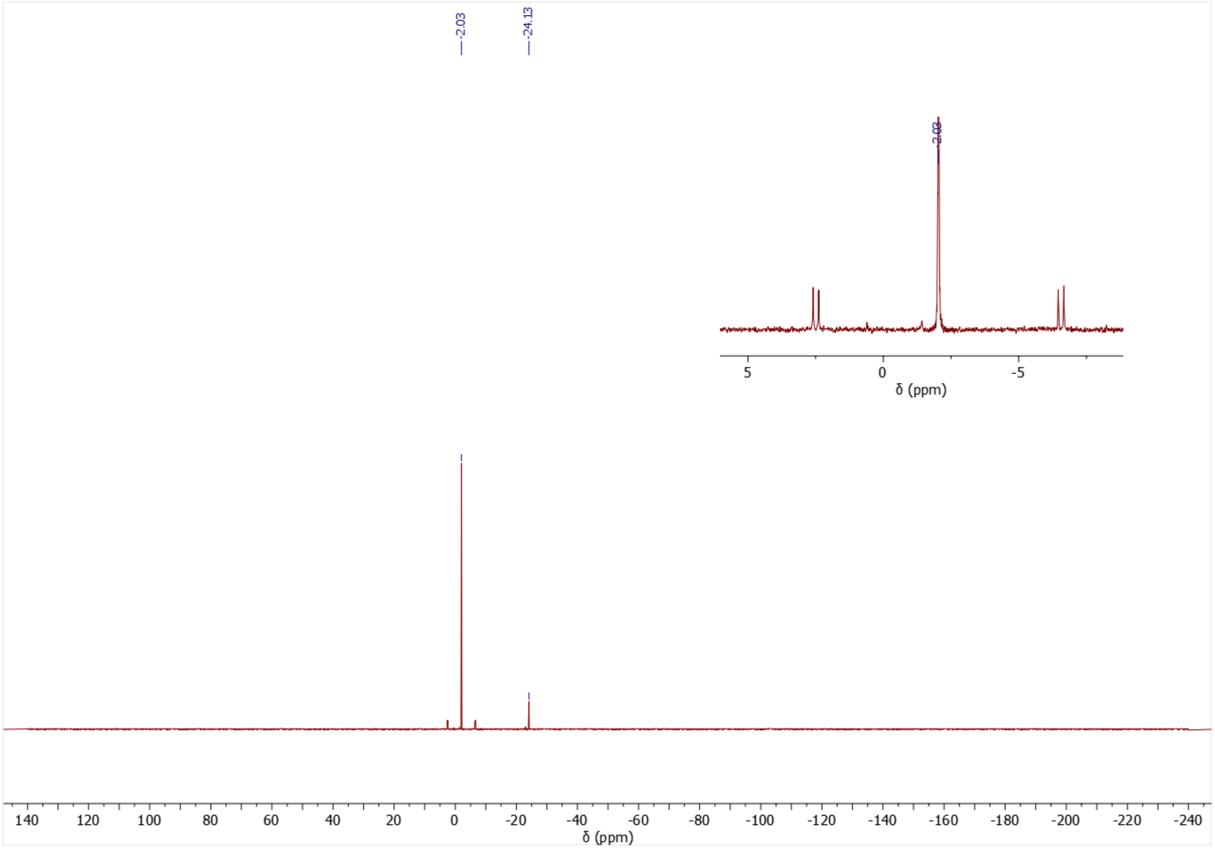

Figure S28. ^{31}P NMR spectrum of compound ^{Ph}L(Br)Sn: as a solution in C_6D_6 at ambient temperature.

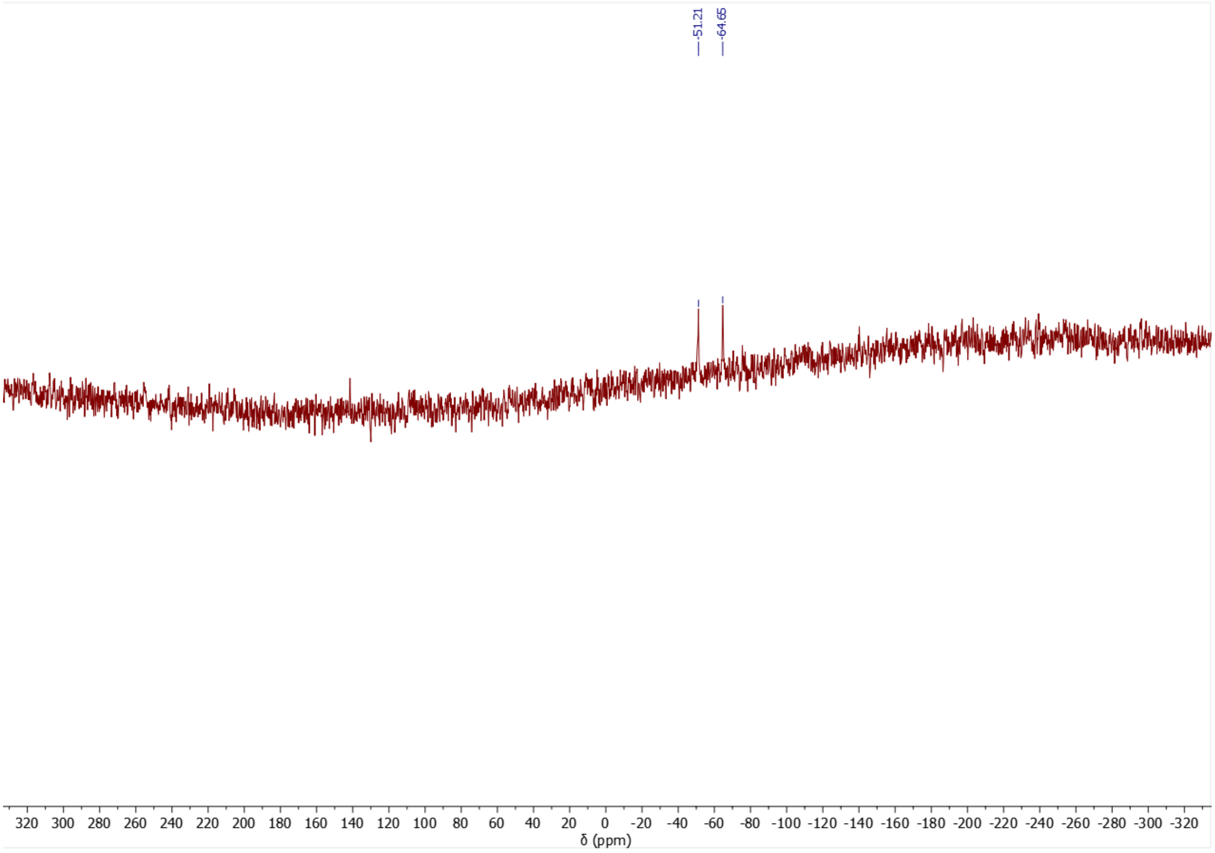

Figure S29. ^{119}Sn NMR spectrum of compound ^{Ph}L(Br)Sn: as a solution in C_6D_6 at ambient temperature.

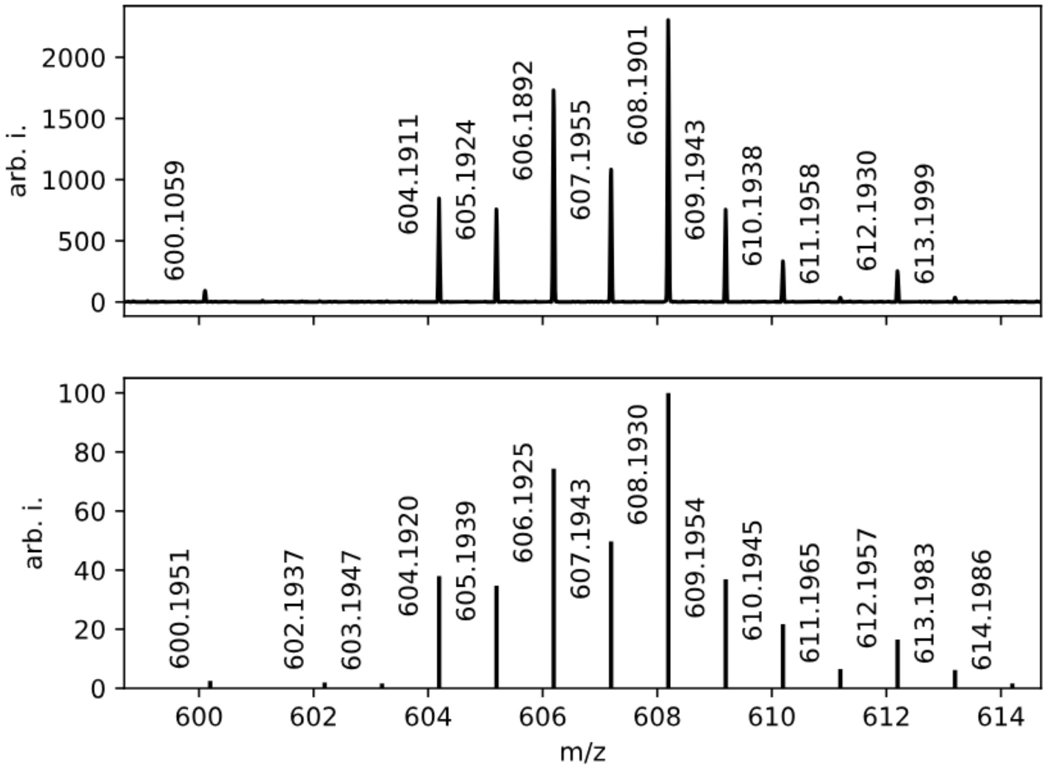

Figure S30. Cutout from LIFDI/MS of compound ^{Ph}L(Br)Sn:; Top. found MS for $[M\text{-}Br]^+$; Bottom. Calculated MS spectrum of $[M\text{-}Br]^+$.

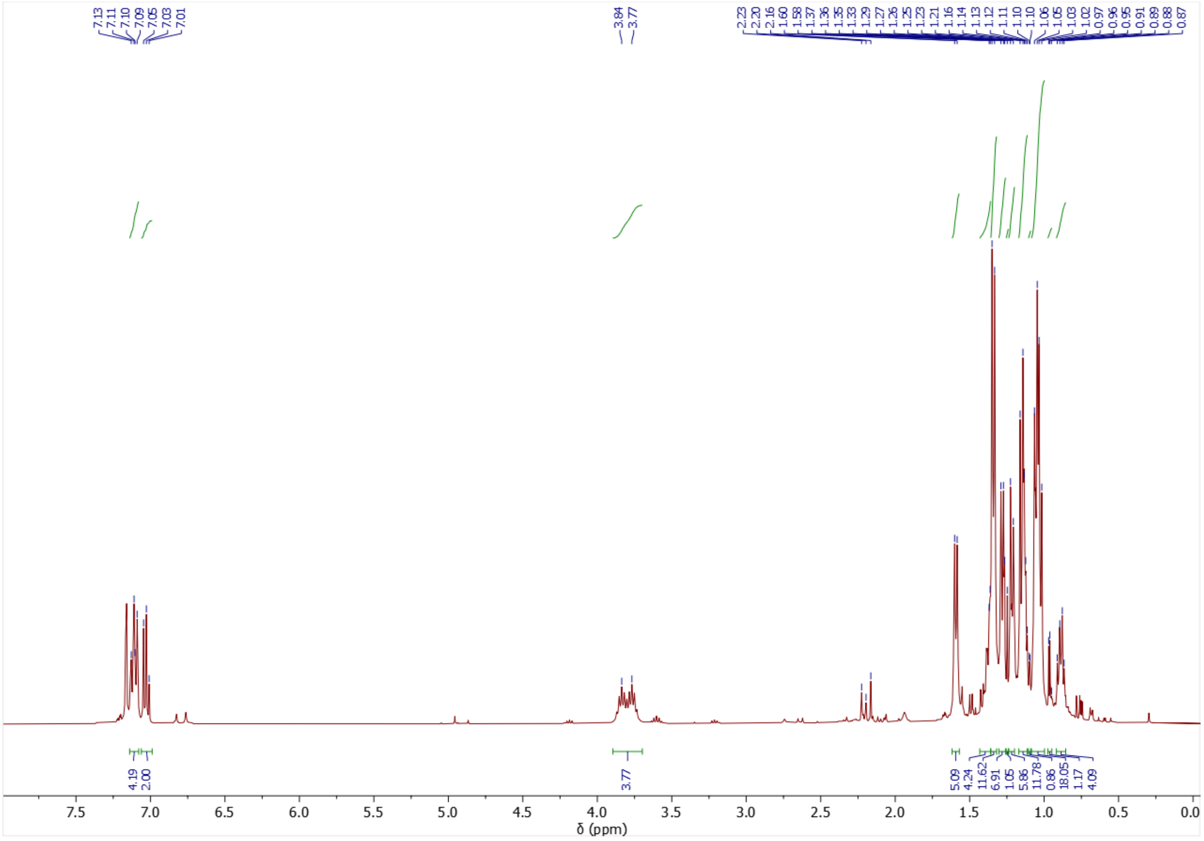

Figure S31. ^{1}H NMR spectrum of compound **1-Me** as a solution in C_6D_6 at ambient temperature.

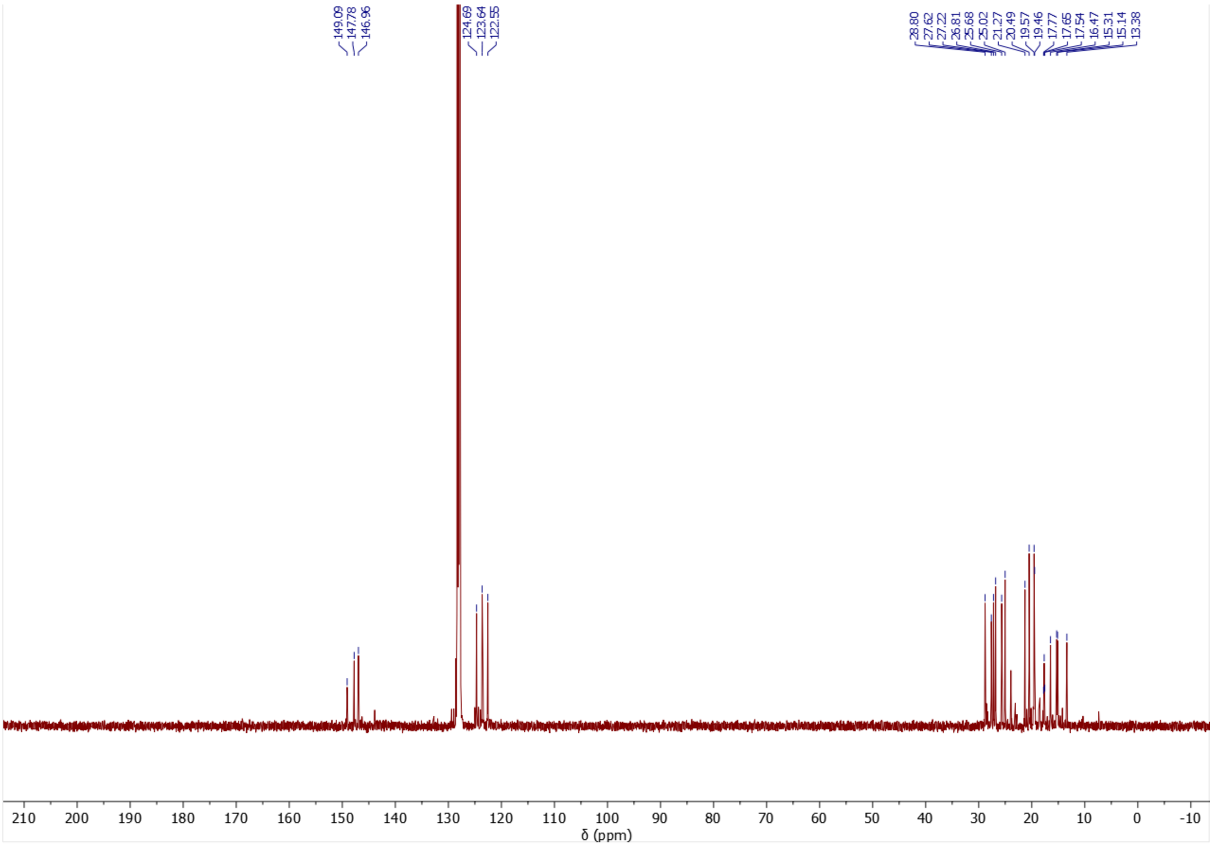

Figure S32. ^{13}C NMR spectrum of compound **1-Me** as a solution in C_6D_6 at ambient temperature.

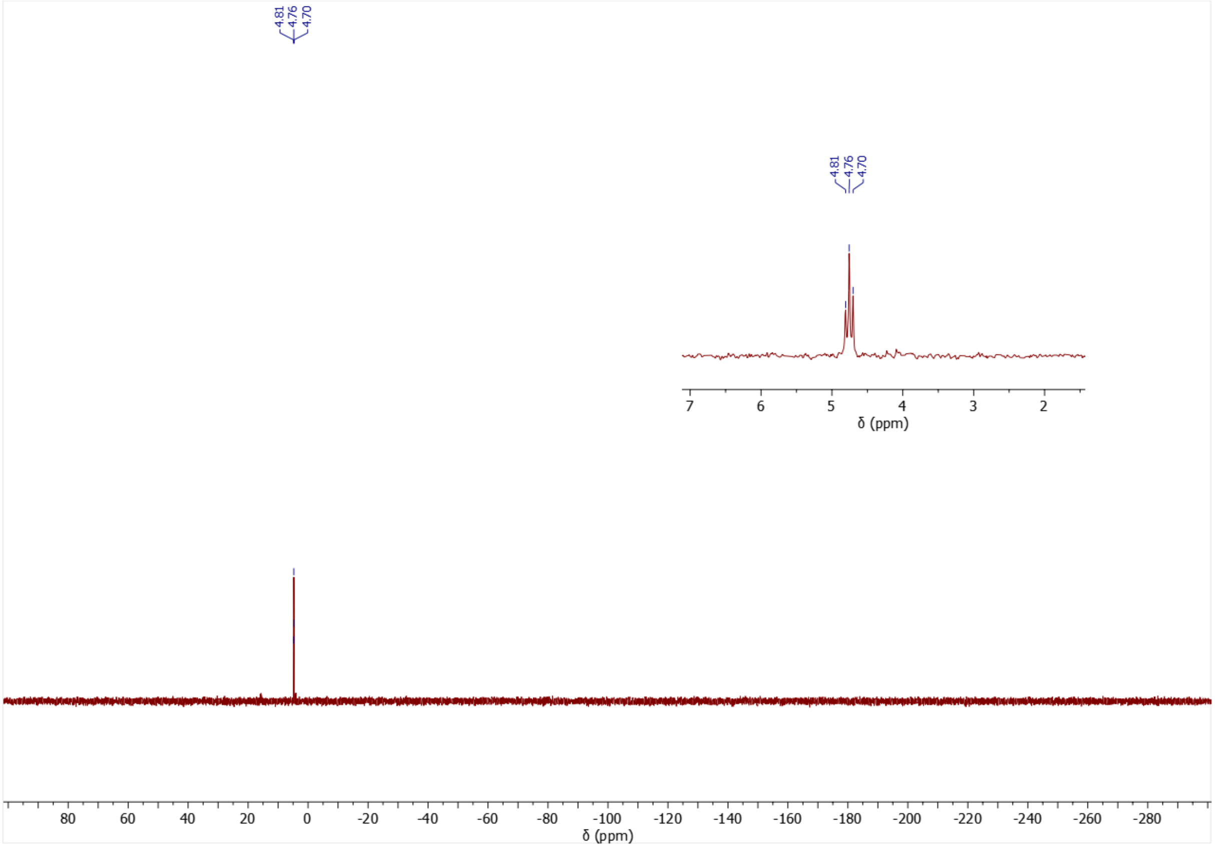

Figure S33. ^{29}Si NMR spectrum of compound **1-Me** as a solution in C_6D_6 at ambient temperature.

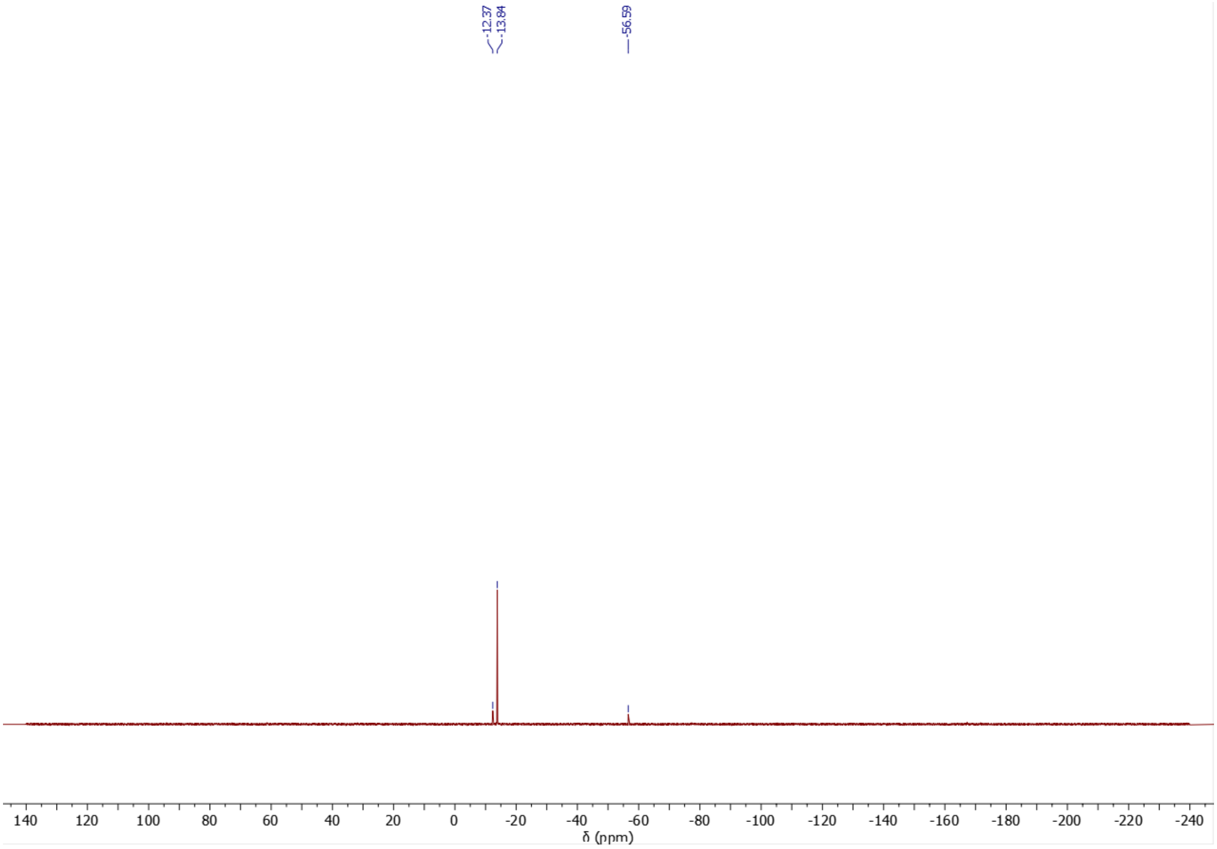

Figure S34. ^{31}P NMR spectrum of compound **1-Me** as a solution in C_6D_6 at ambient temperature.

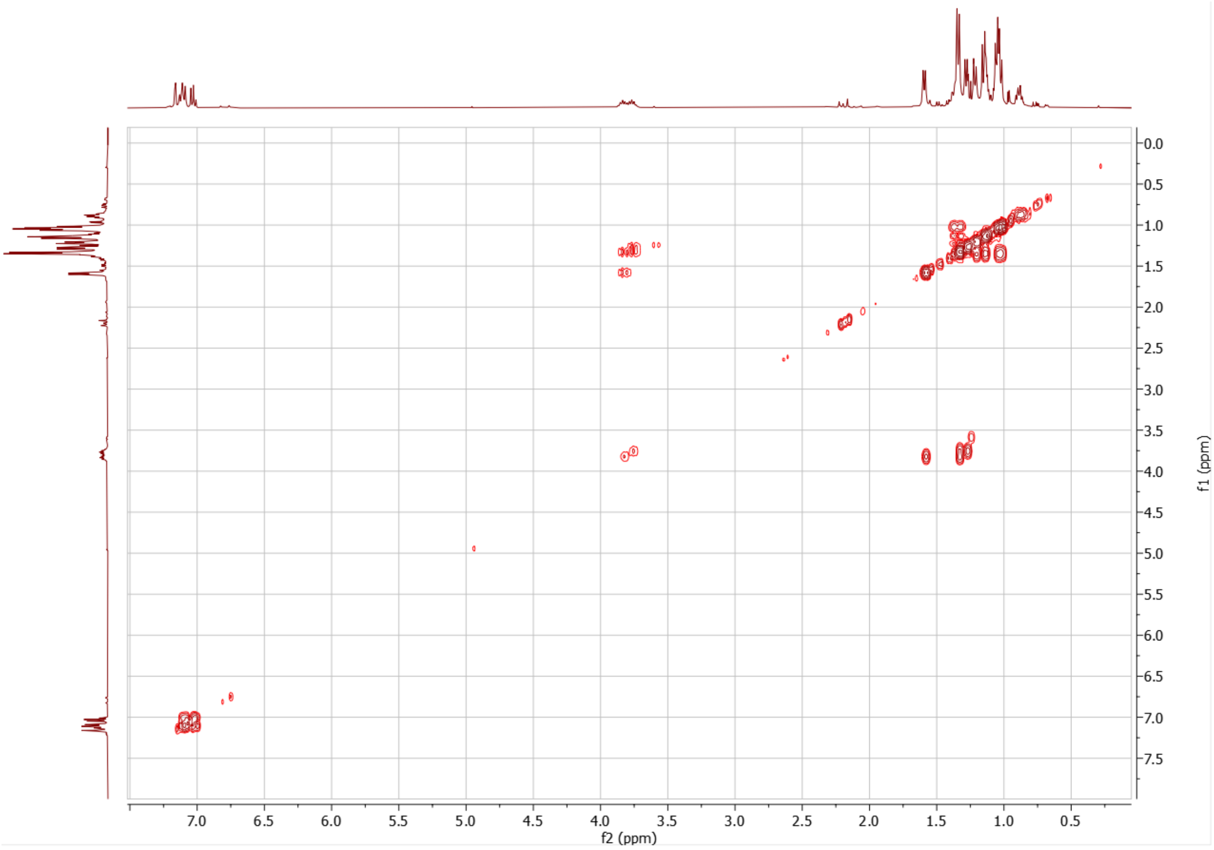

Figure S35. COSY NMR spectrum of compound **1-Me** as a solution in C_6D_6 at ambient temperature.

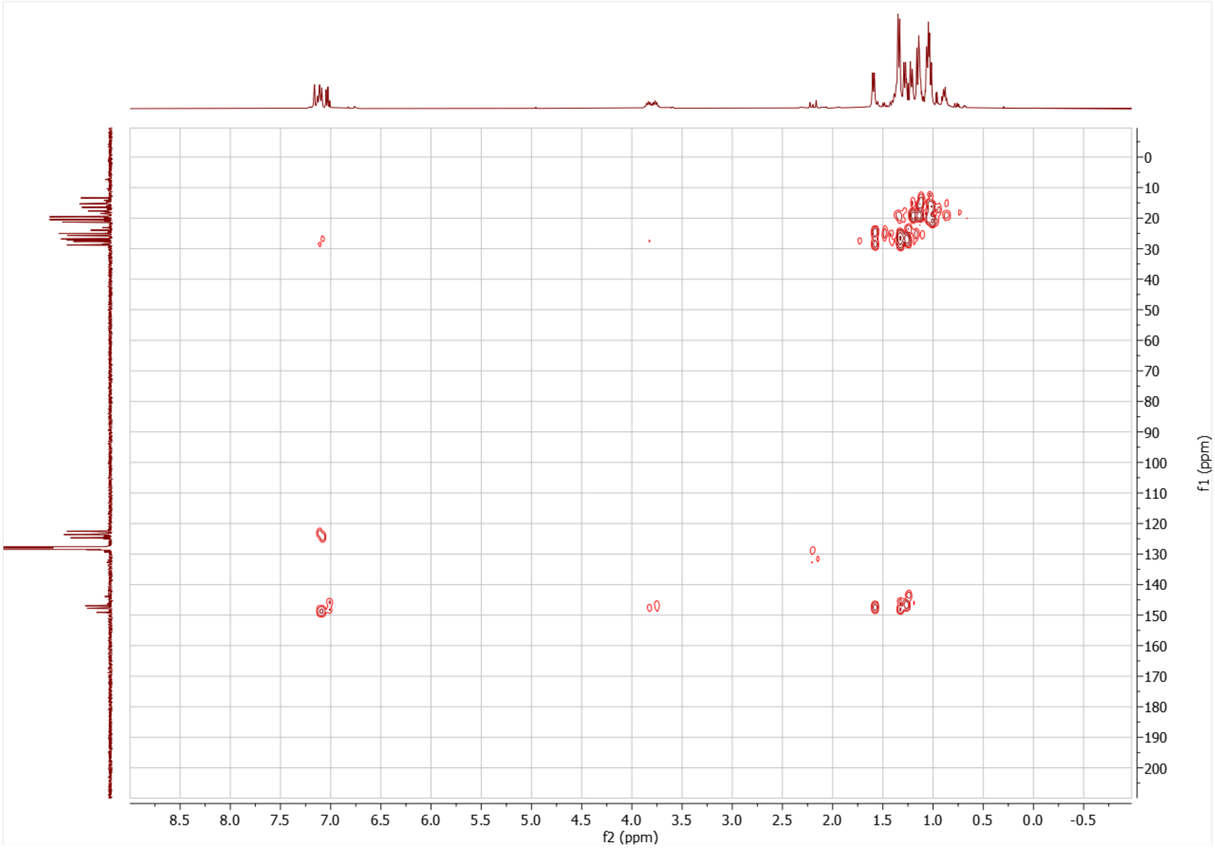

Figure S36. HMBC NMR spectrum of compound **1-Me** as a solution in C_6D_6 at ambient temperature.

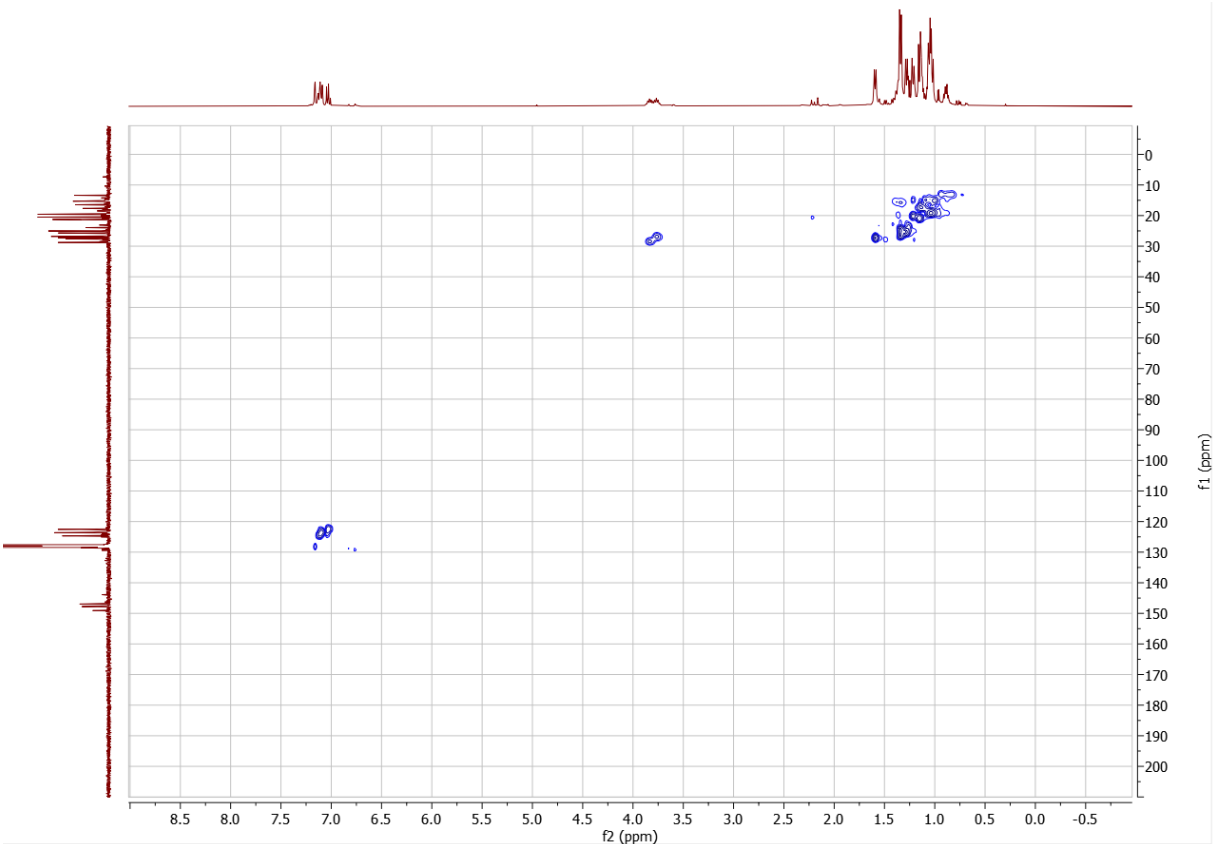

Figure S37. HSQC NMR spectrum of compound **1-Me** as a solution in C_6D_6 at ambient temperature.

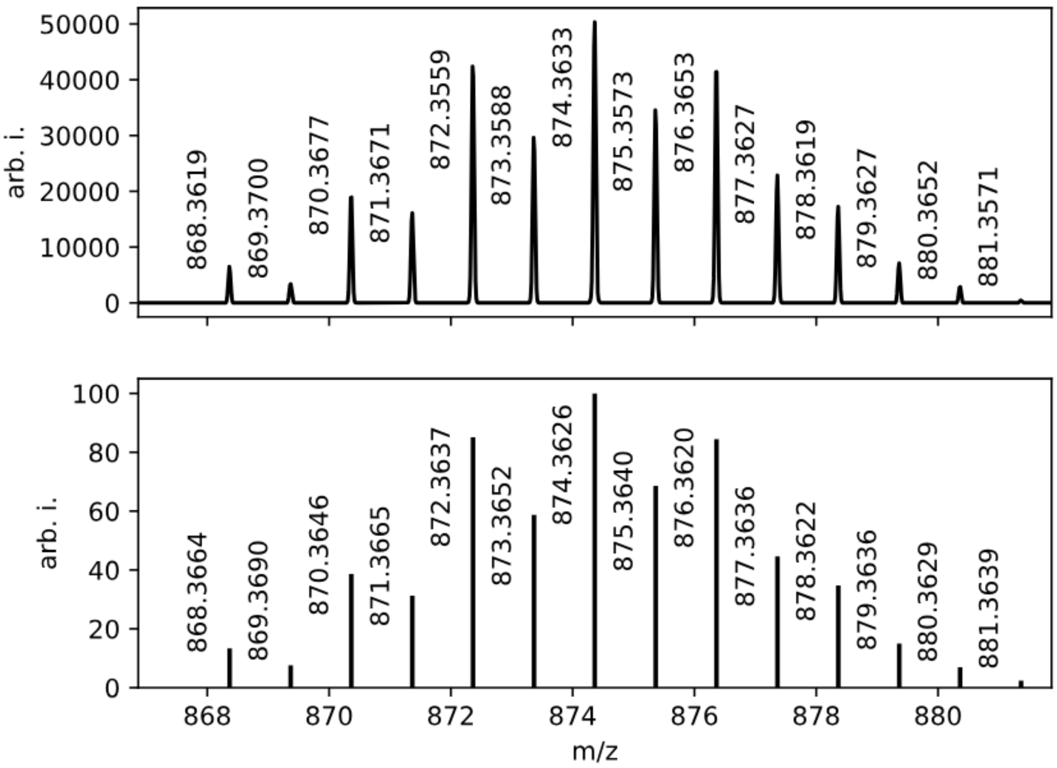

Figure S38. Cutout from LIFDI/MS of compound **1-Me**; Top. found MS for $[M]^+$; Bottom. Calculated MS spectrum of $[M]^+$.

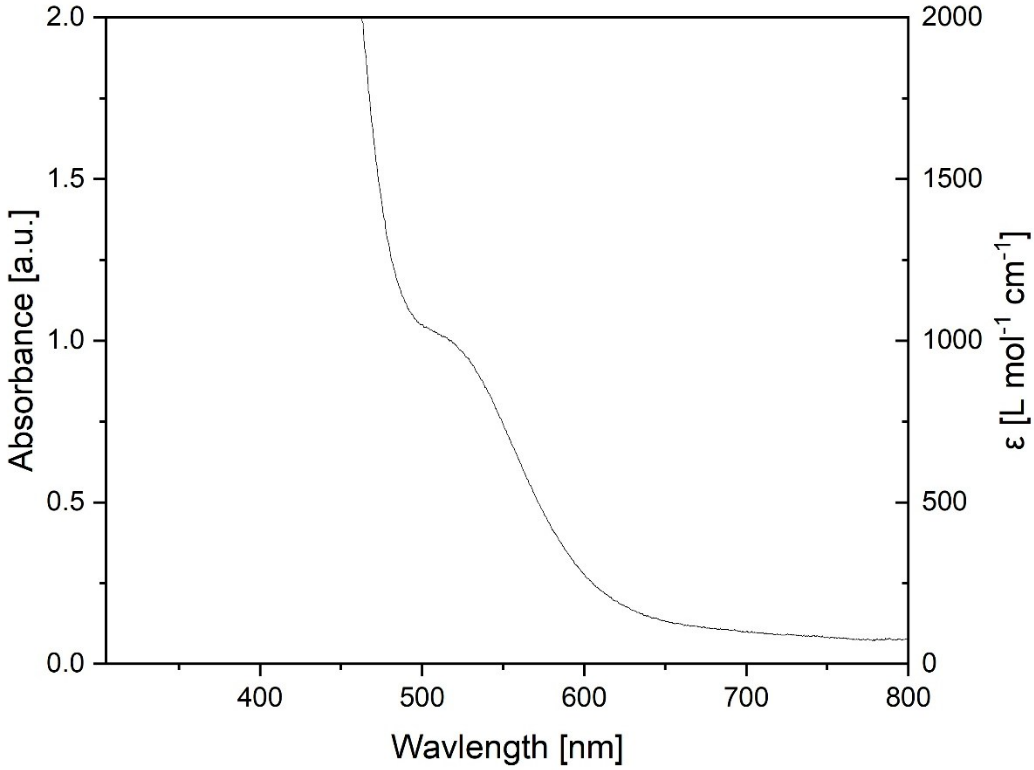

Figure S39. UV/vis spectrum of a 1.0x10^{-3} M solution of compound **1-Me** in toluene at ambient temperature.

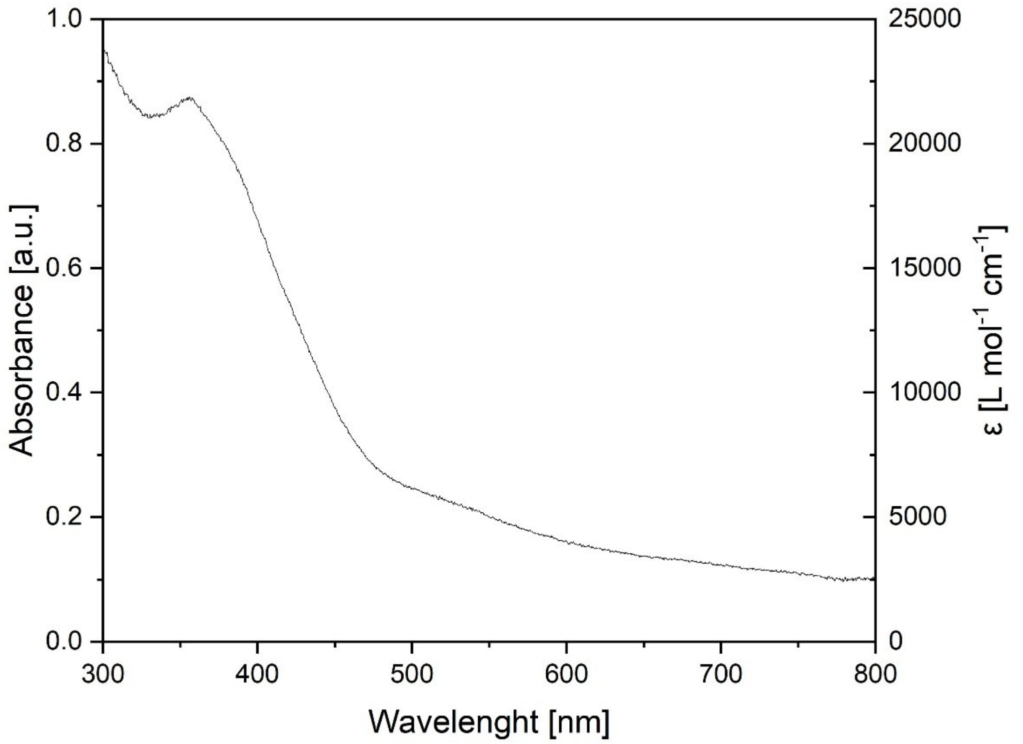

Figure S40. UV/vis spectrum of a 4.0x10^{-5} M solution of compound **1-Me** in toluene at ambient temperature.

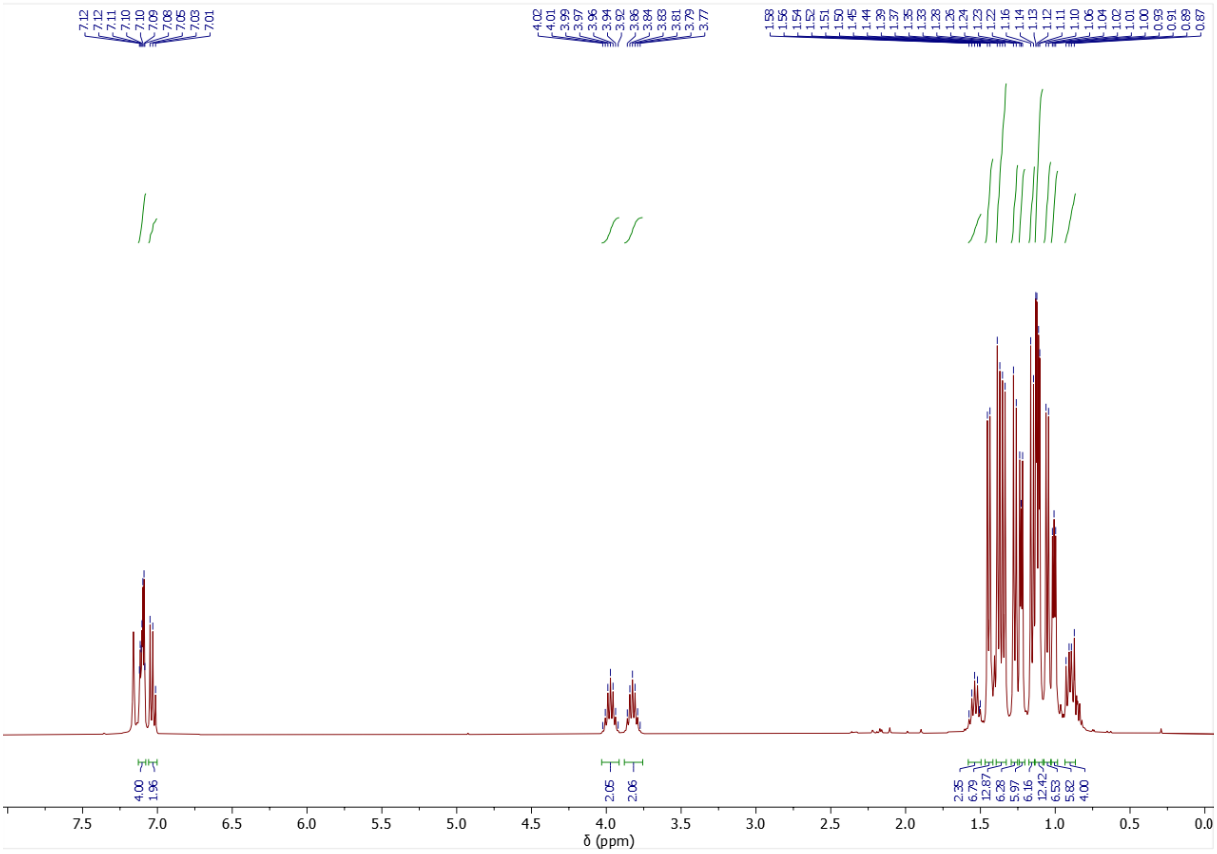

Figure S41. ^{1}H NMR spectrum of compound **2-Me** as a solution in C_6D_6 at ambient temperature.

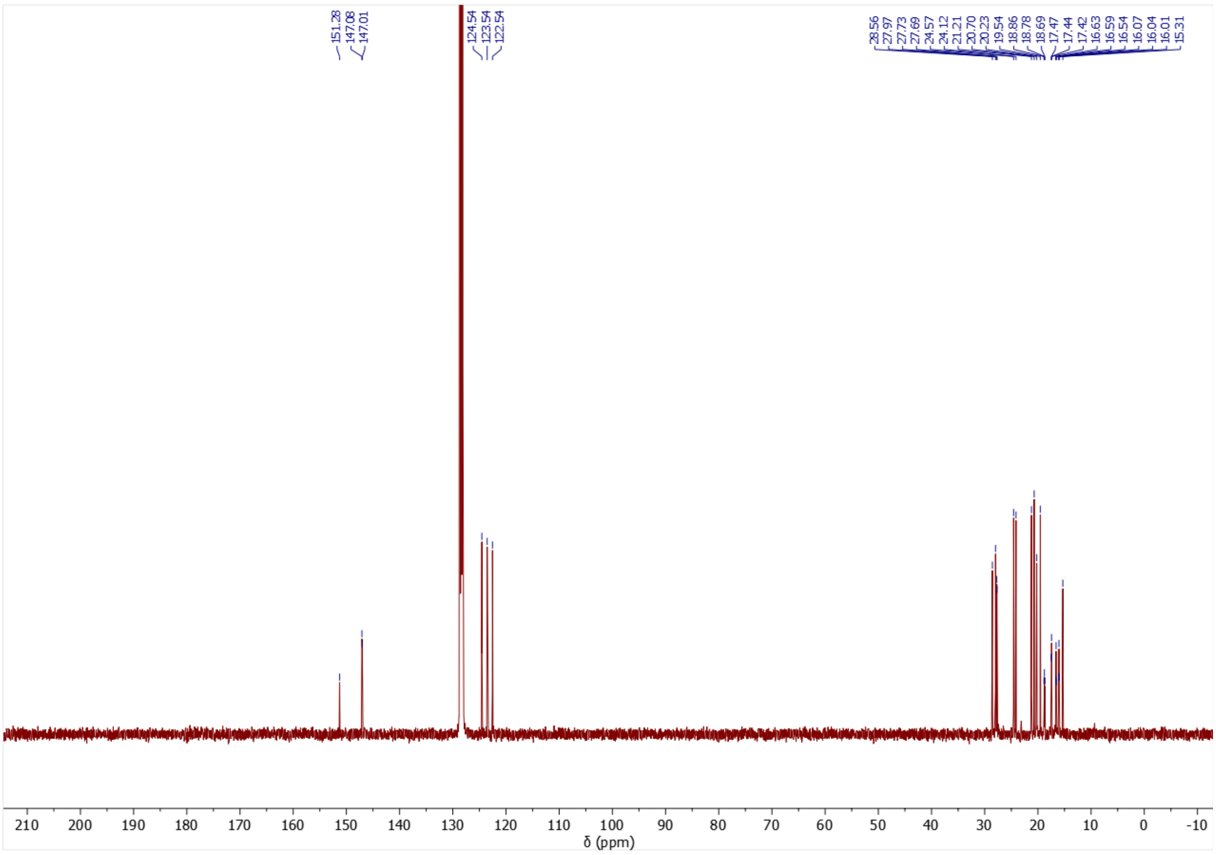

Figure S42. ^{13}C NMR spectrum of compound **2-Me** as a solution in C_6D_6 at ambient temperature.

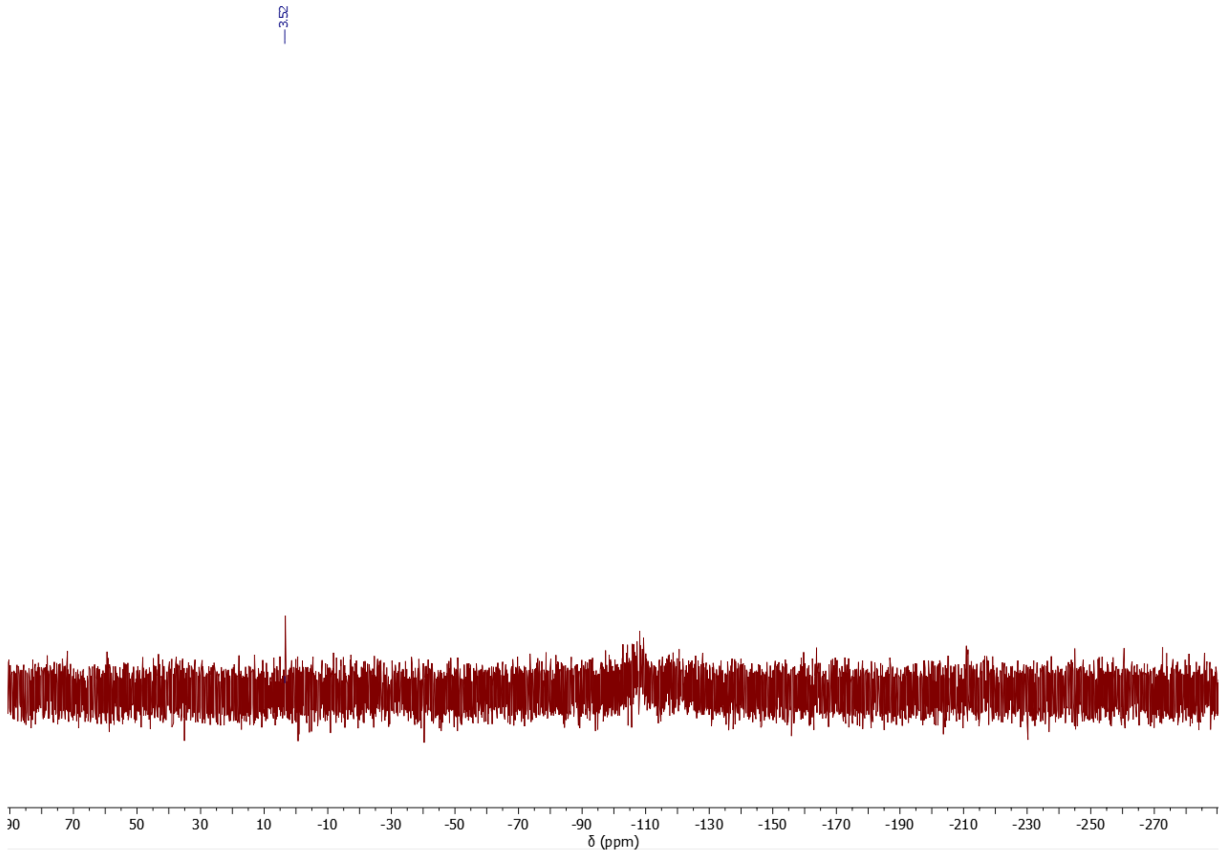

Figure S43. ^{29}Si NMR spectrum of compound **2-Me** as a solution in C_6D_6 at ambient temperature.

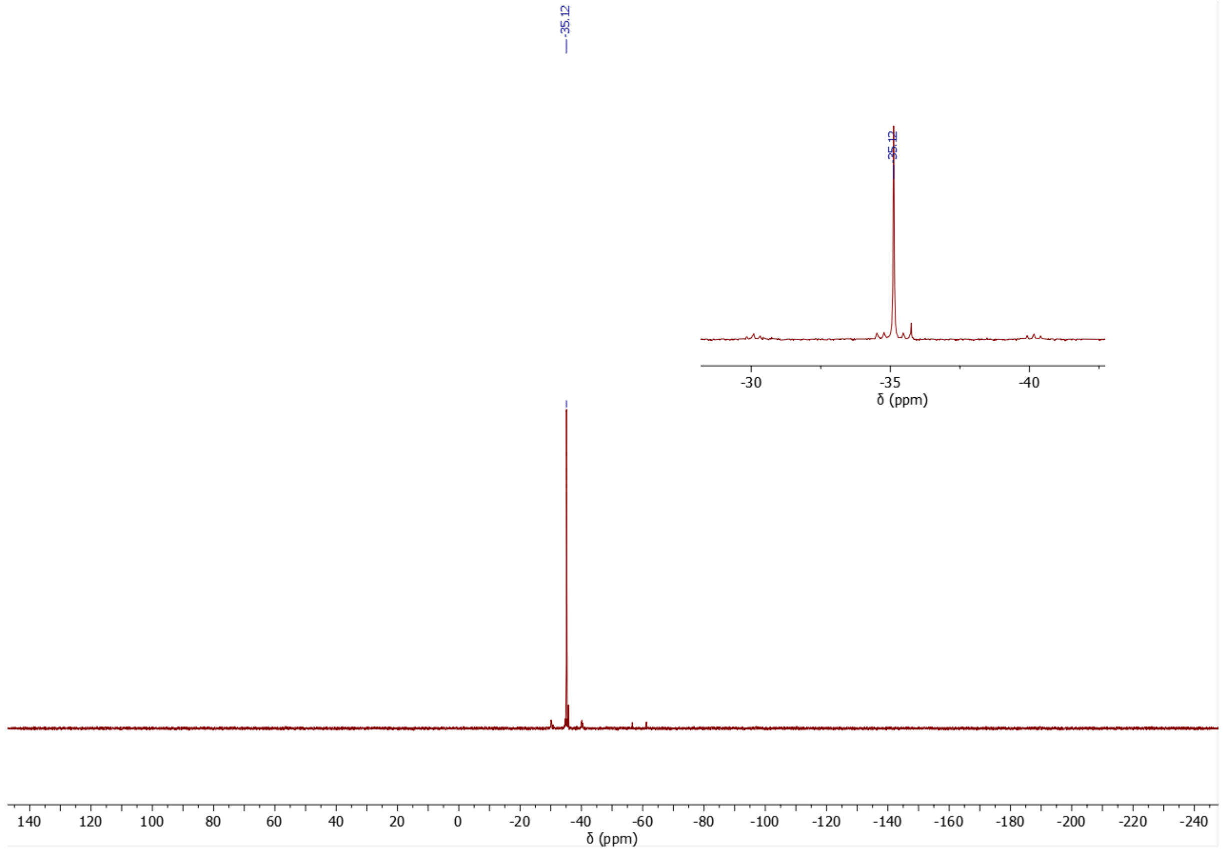

Figure S44. ^{31}P NMR spectrum of compound **2-Me** as a solution in C_6D_6 at ambient temperature.

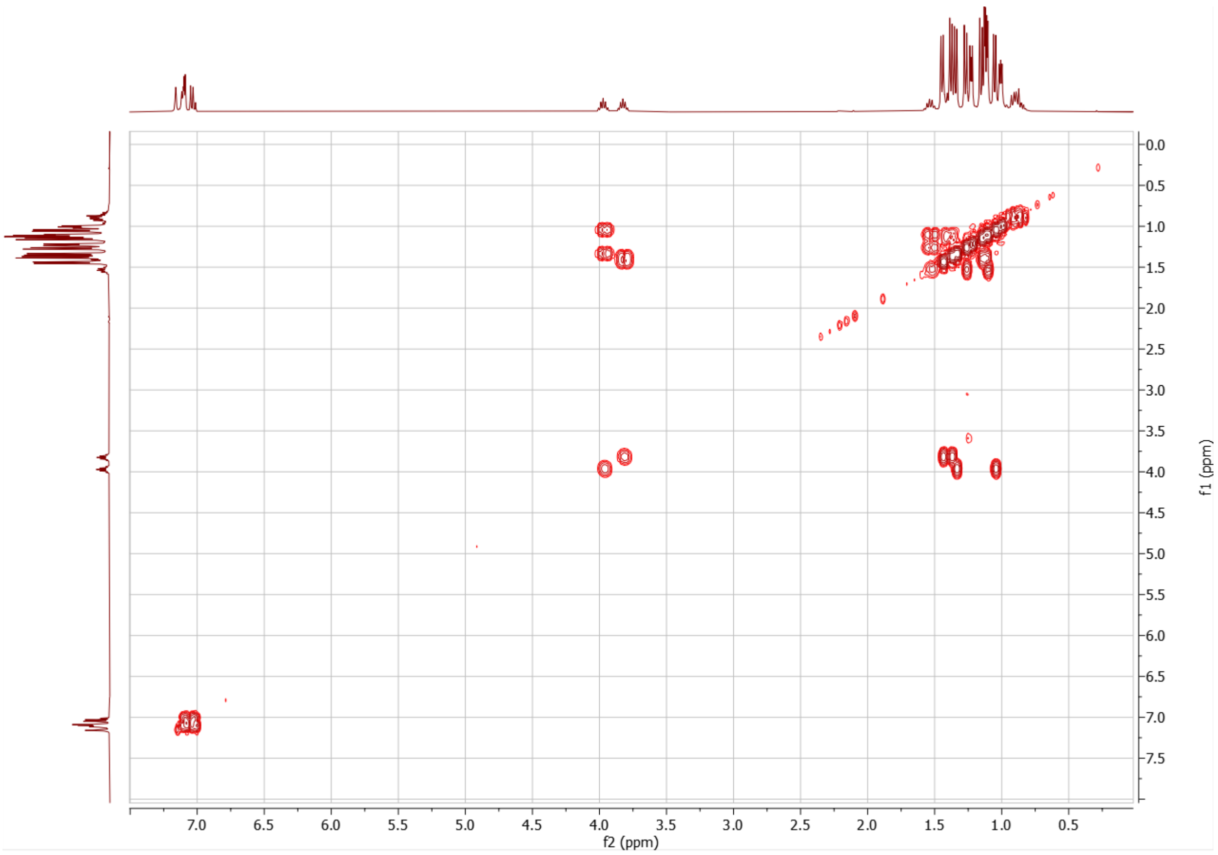

Figure S45. COSY NMR spectrum of compound **2-Me** as a solution in C_6D_6 at ambient temperature.

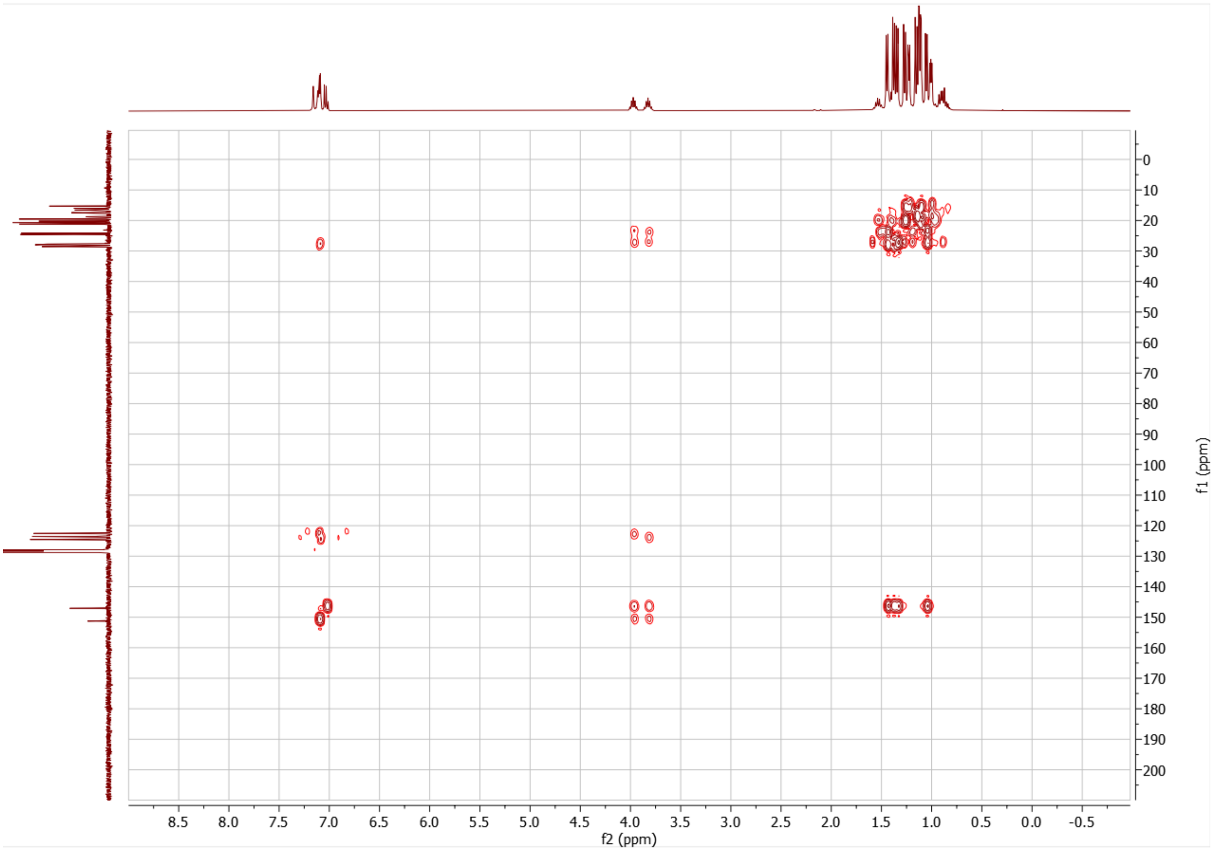

Figure S46. HMBC NMR spectrum of compound **2-Me** as a solution in C_6D_6 at ambient temperature.

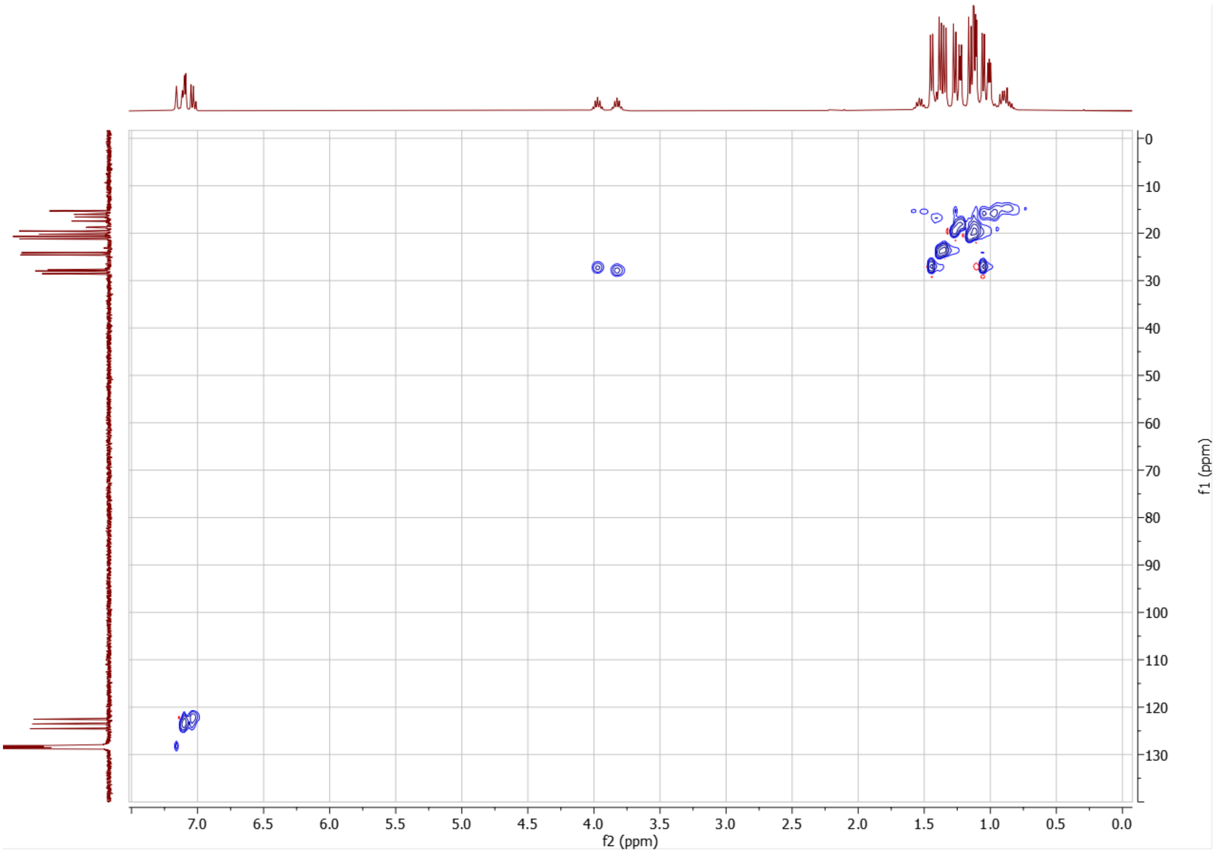

Figure S47. HSQC NMR spectrum of compound **2-Me** as a solution in C_6D_6 at ambient temperature.

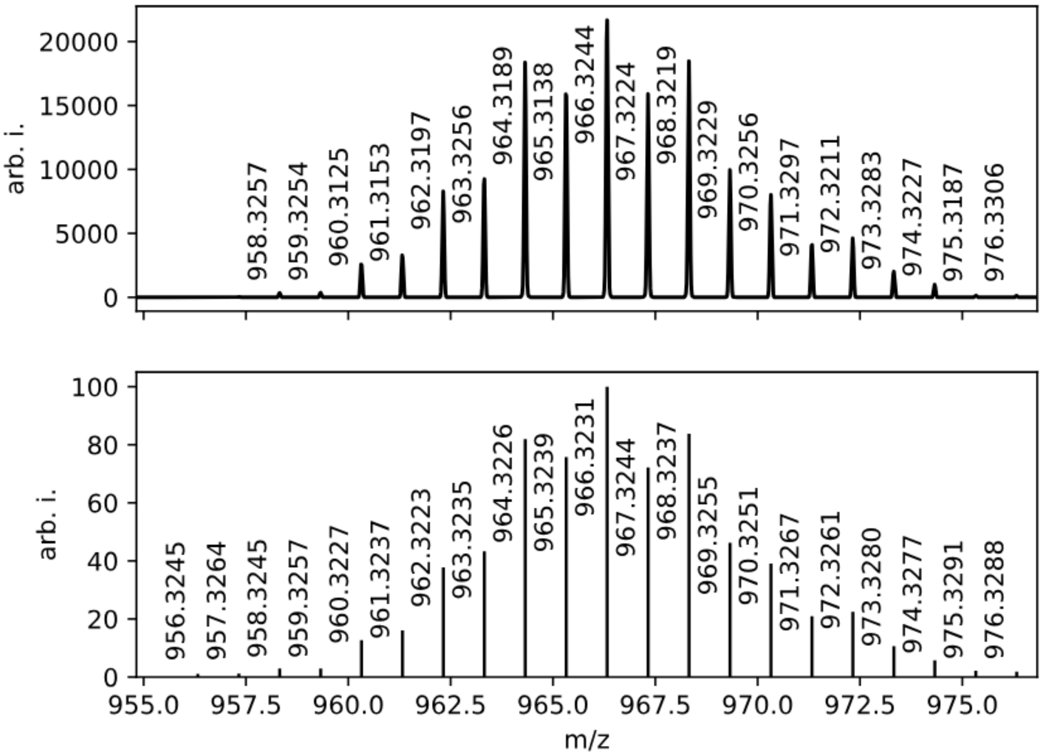

Figure S48. Cutout from LIFDI/MS of compound **2-Me**; Top. found MS for $[M]^+$; Bottom. Calculated MS spectrum of $[M]^+$.

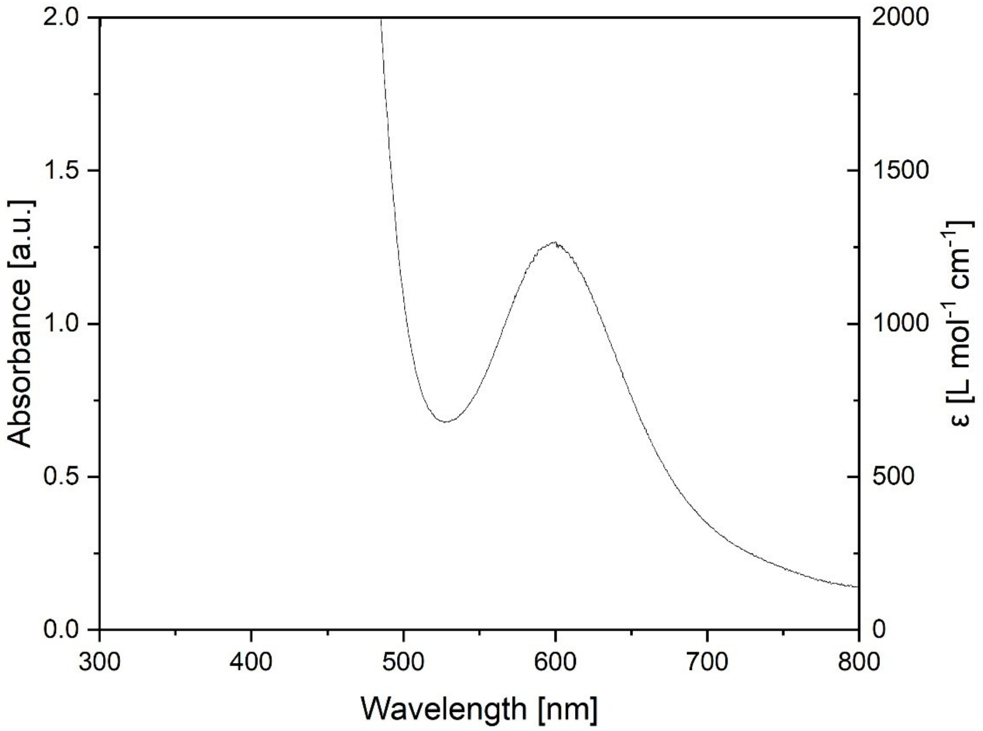

Figure S49. UV/vis spectrum of a 1.0x10^{-3} M solution of compound **2-Me** in toluene at ambient temperature.

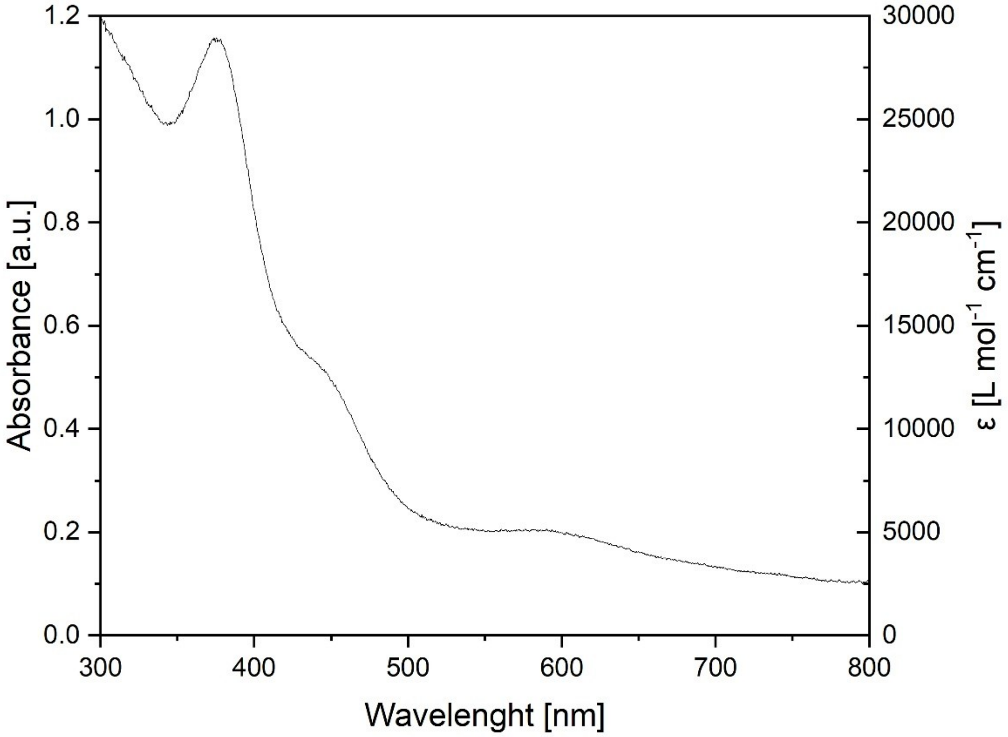

Figure S50. UV/vis spectrum of a 4.0x10^{-5} M solution of compound **2-Me** in toluene at ambient temperature.

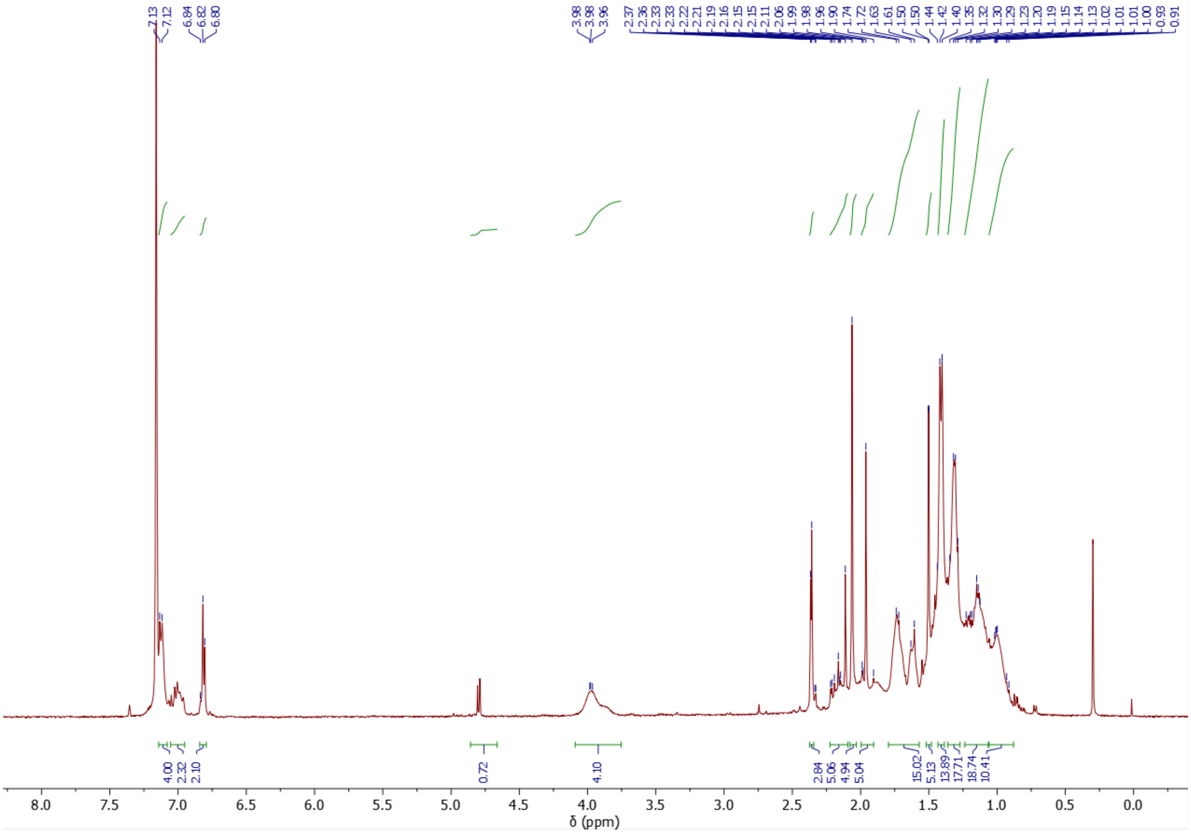

Figure S51. ^{1}H NMR spectrum of compound **2-Cy** as a solution in C_6D_6 at ambient temperature.

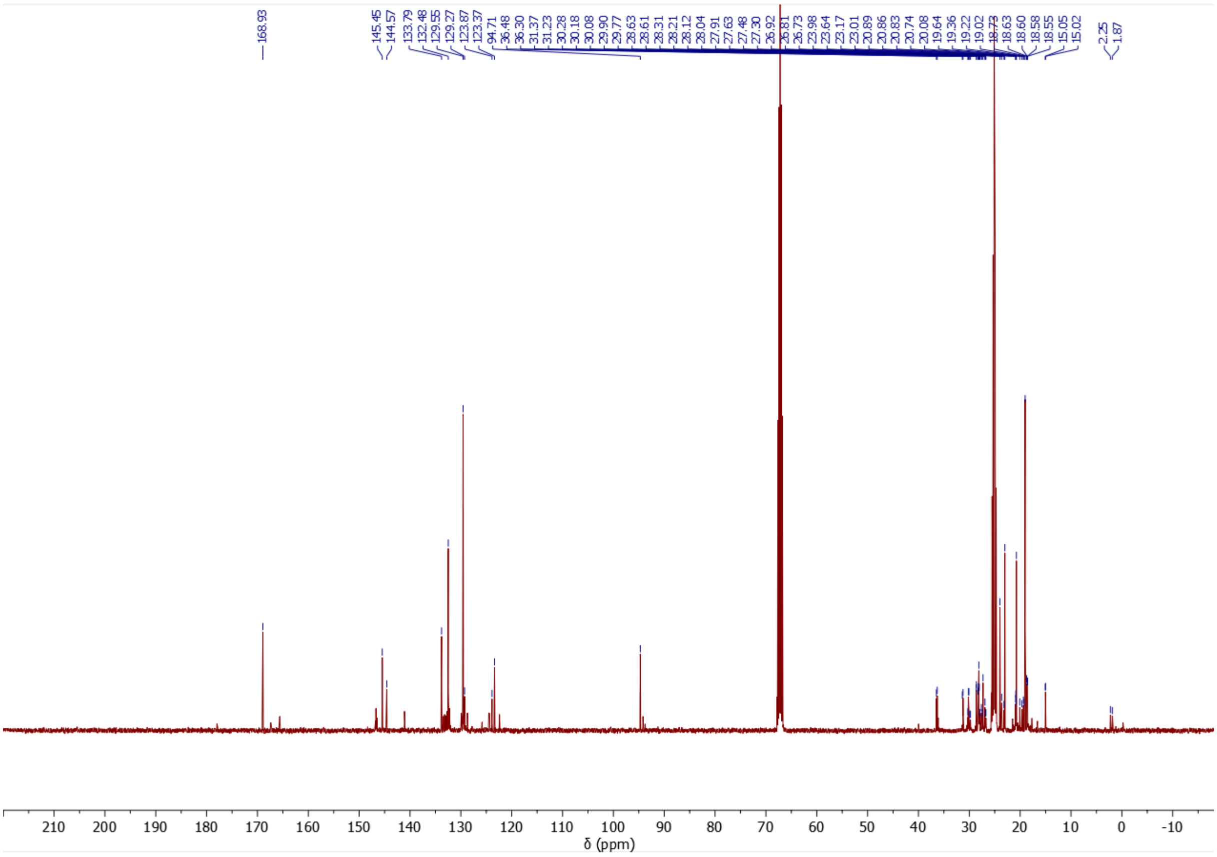

Figure S52. ^{13}C NMR spectrum of compound **2-Cy** as a solution in THF-d_8 at ambient temperature.

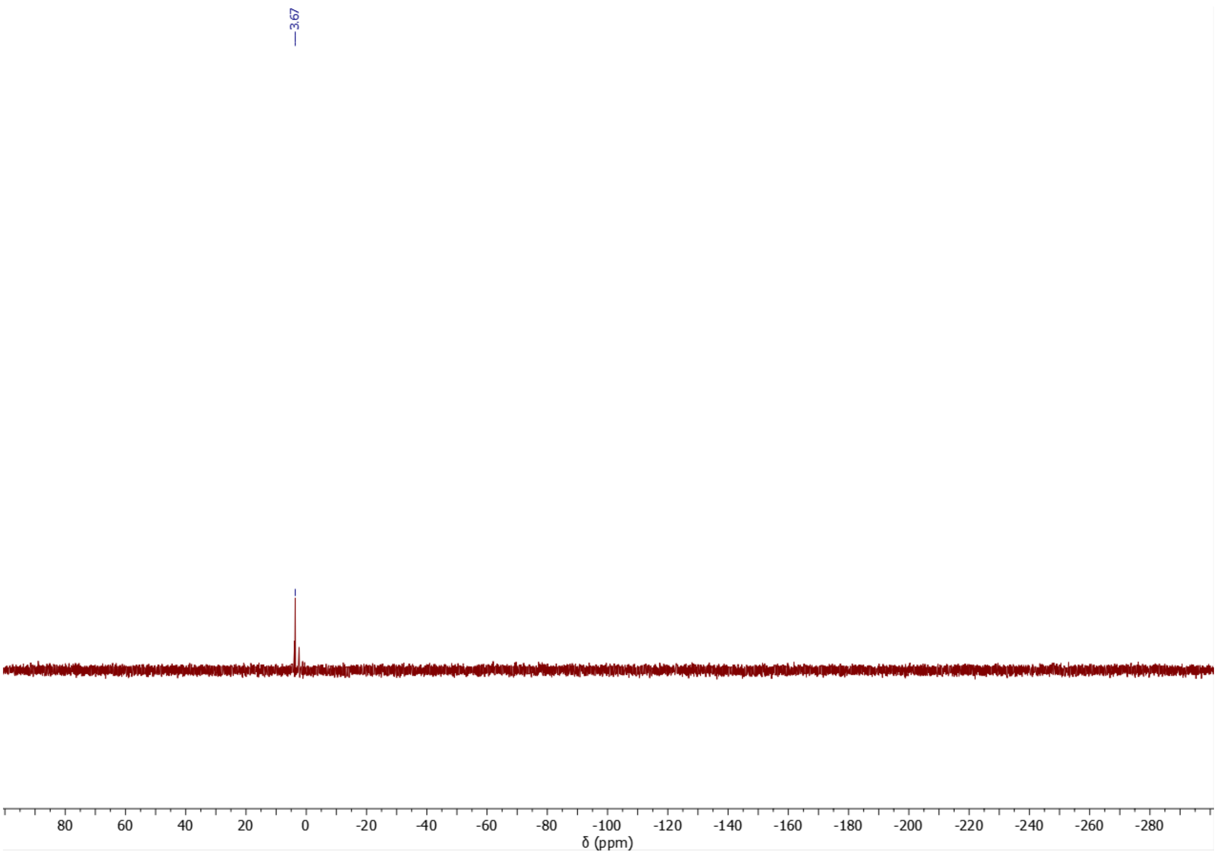

Figure S53. ^{29}Si NMR spectrum of compound **2-Cy** as a solution in THF-d_8 at ambient temperature.

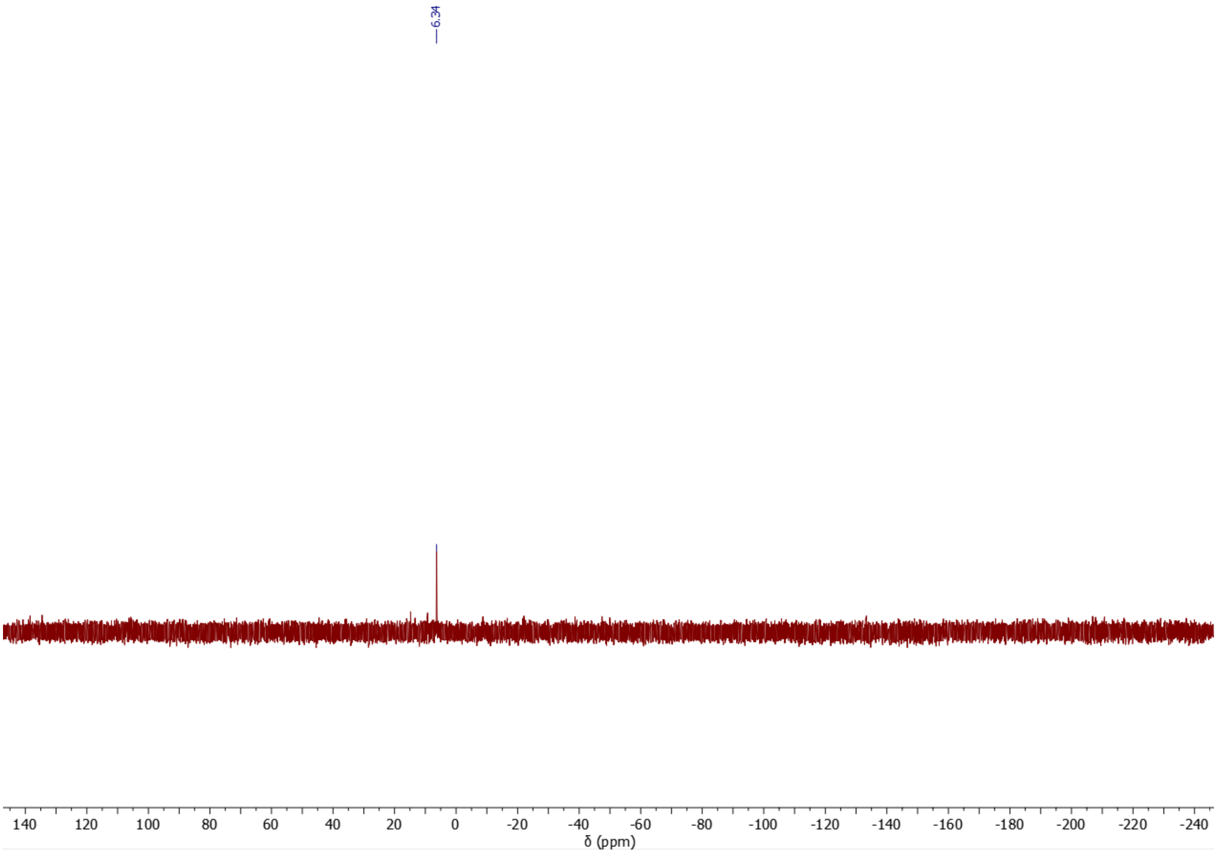

Figure S54. ^{31}P NMR spectrum of compound **2-Cy** as a solution in C_6D_6 at ambient temperature.

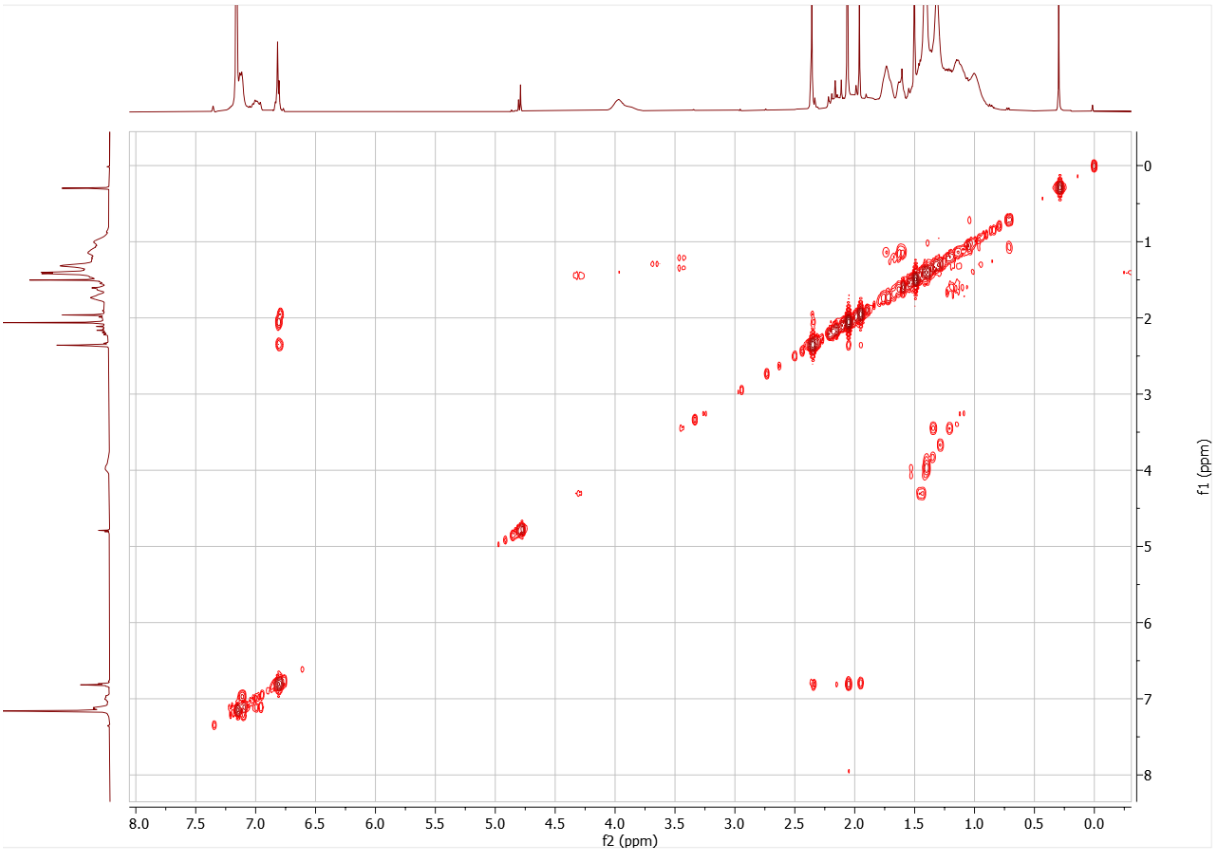

Figure S55. COSY NMR spectrum of compound **2-Cy** as a solution in C_6D_6 at ambient temperature.

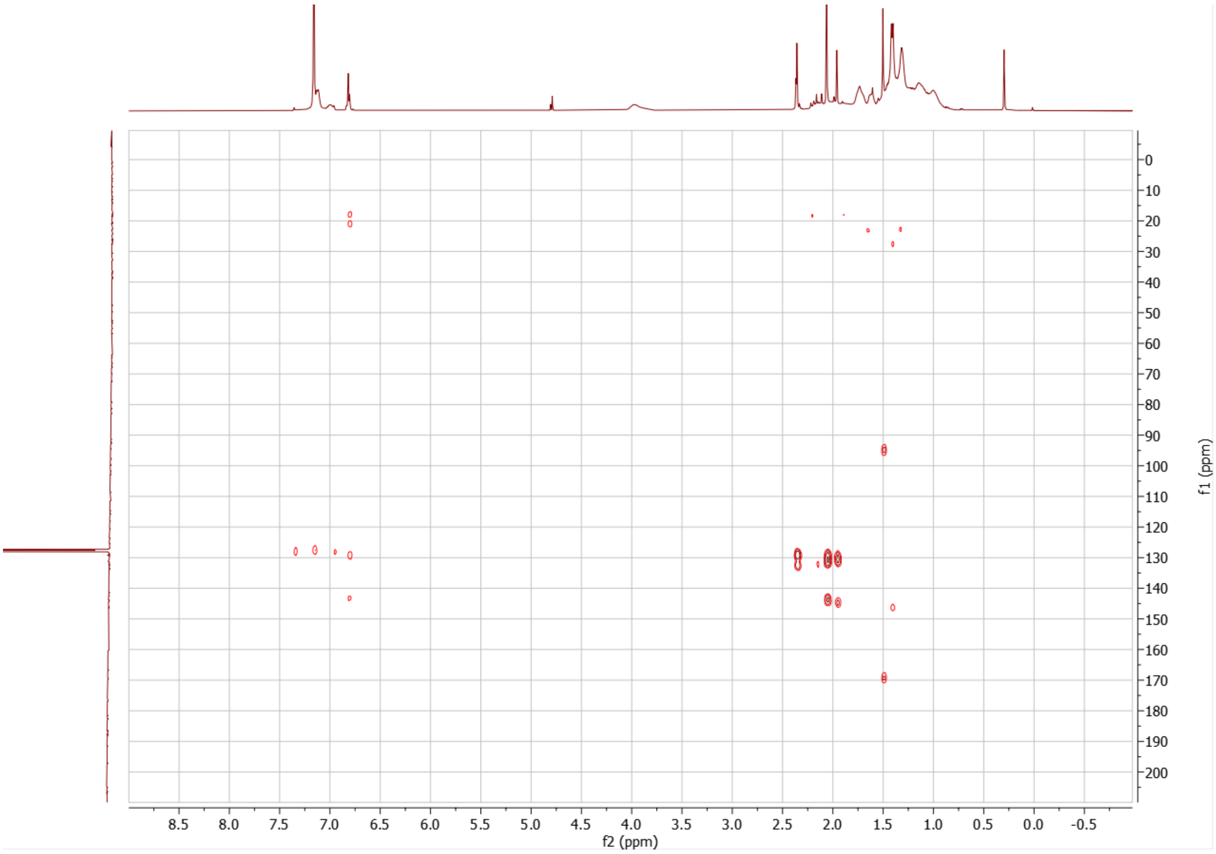

Figure S56. HMBC NMR spectrum of compound **2-Cy** as a solution in C_6D_6 at ambient temperature.

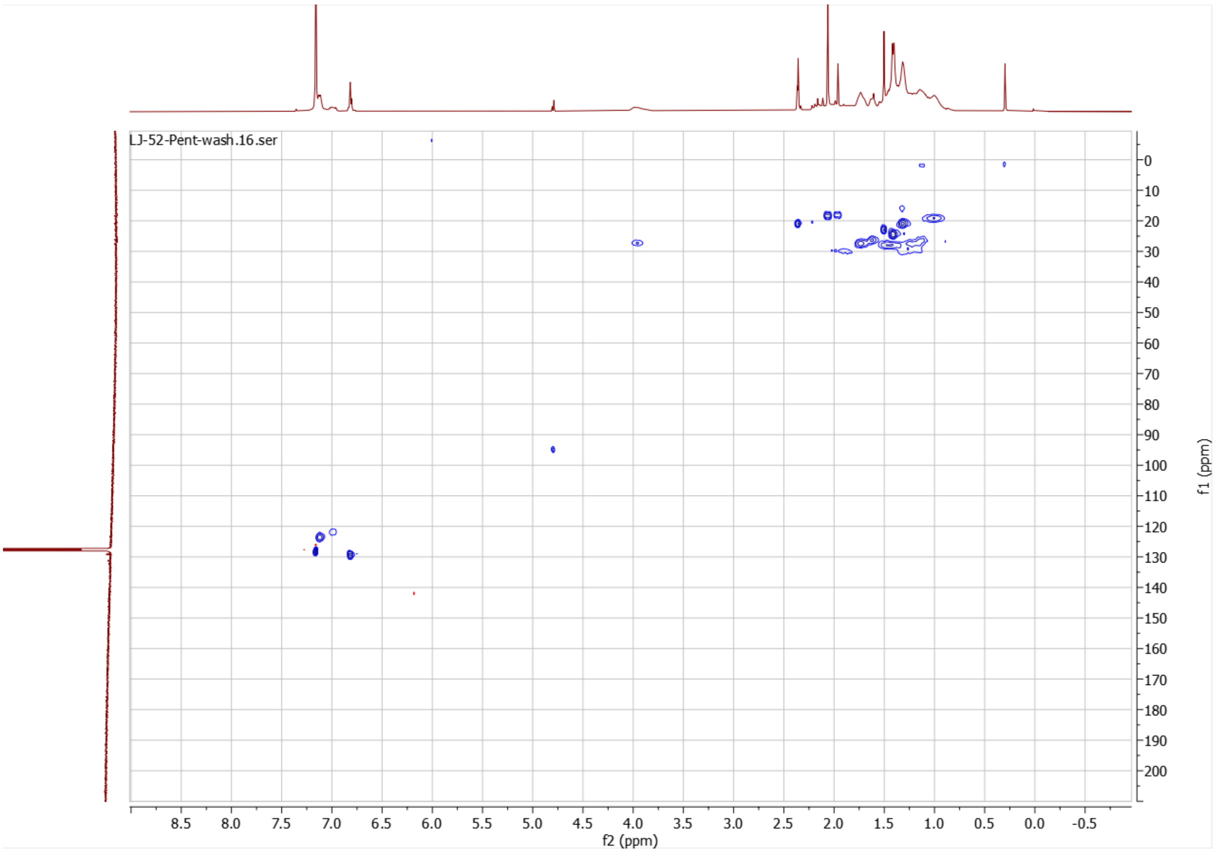

Figure S57. HSQC NMR spectrum of compound **2-Cy** as a solution in C_6D_6 at ambient temperature.

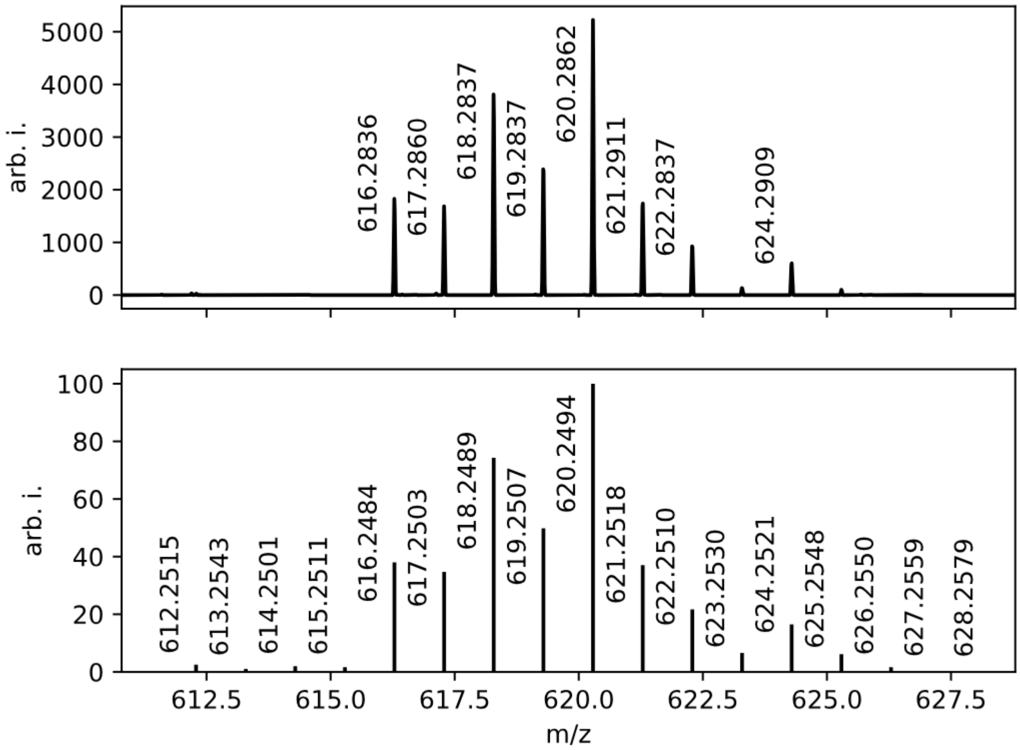

Figure S58. Cutout from LIFDI/MS of compound **2-Cy**; Top. found MS for $[M-^{Cy}LSn]^+$; Bottom. Calculated MS spectrum of $[M-^{Cy}LSn]^+$.

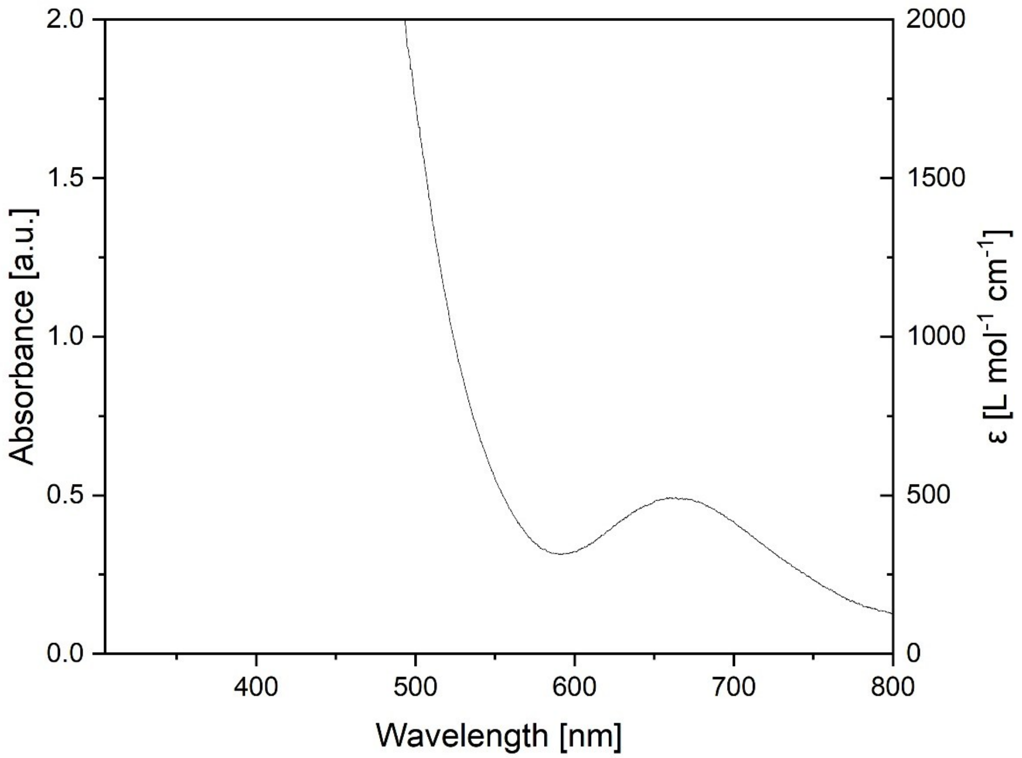

Figure S59. UV/vis spectrum of a 1.0x10^{-3} M solution of compound **2-Cy** in toluene at ambient temperature.

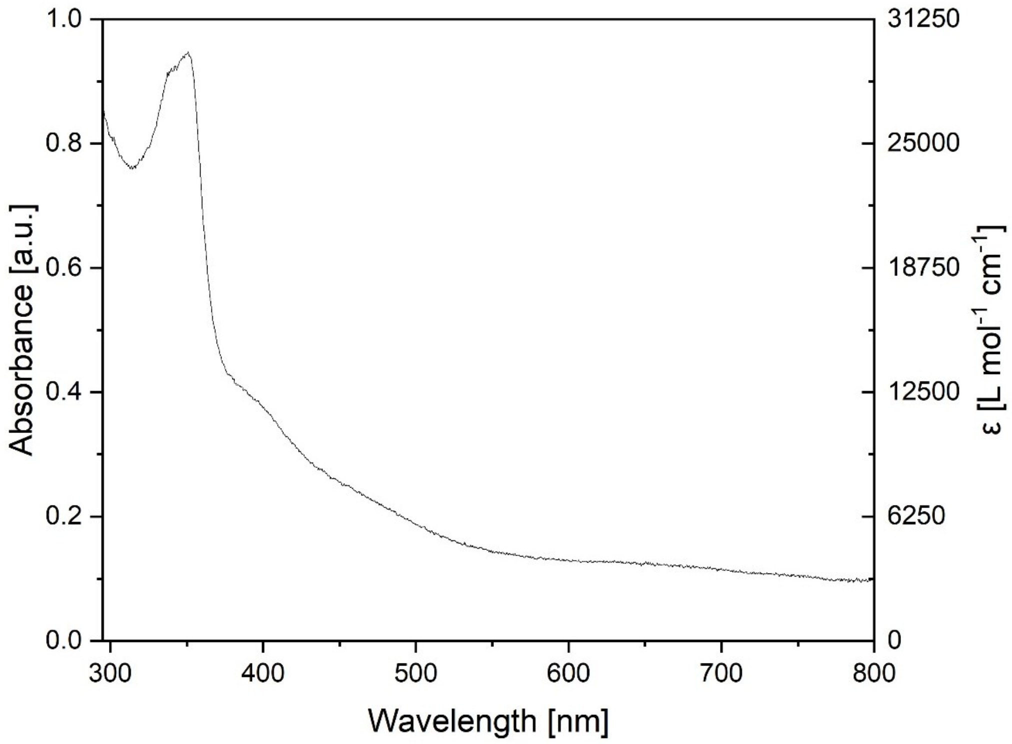

Figure S60. UV/vis spectrum of a 3.2x10^{-5} M solution of compound **2-Cy** in toluene at ambient temperature.

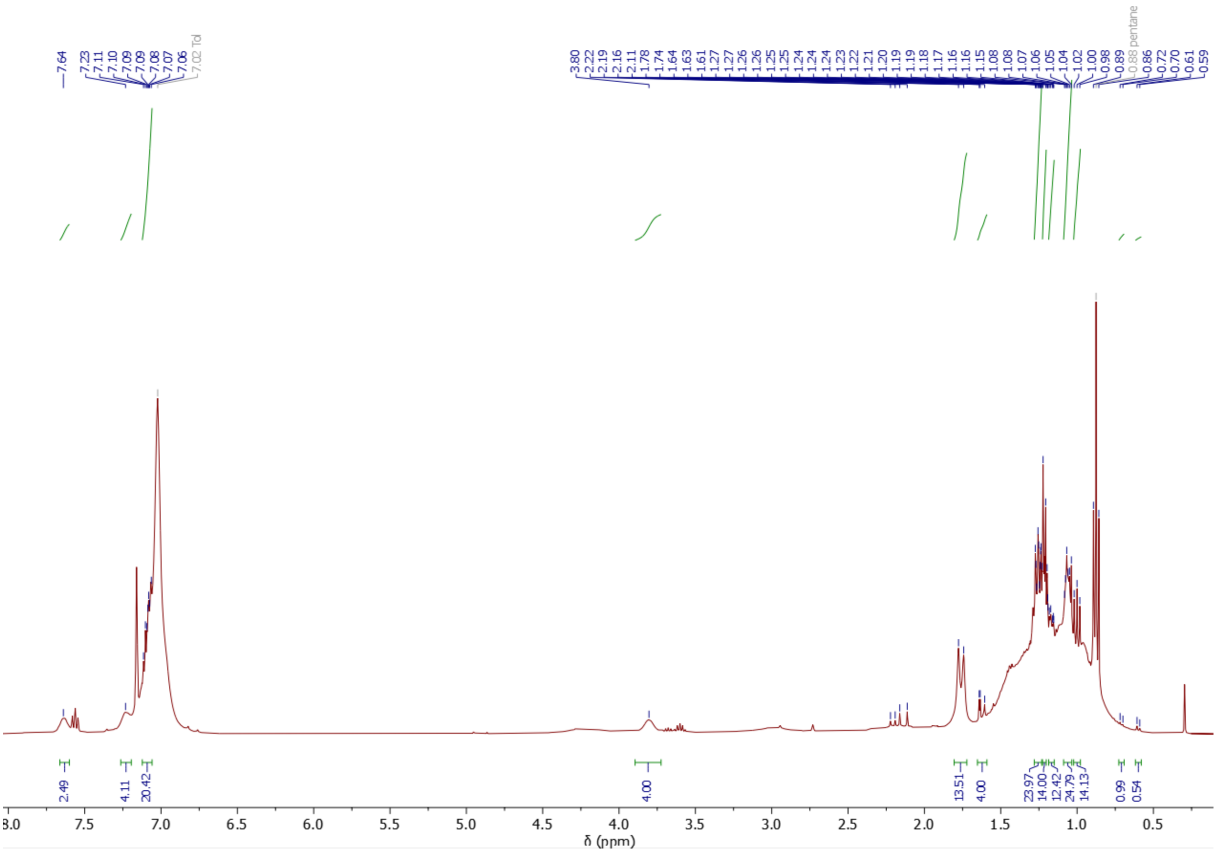

Figure S61. ^{1}H NMR spectrum of compound **2-Ph** as a solution in C_6D_6 at ambient temperature.

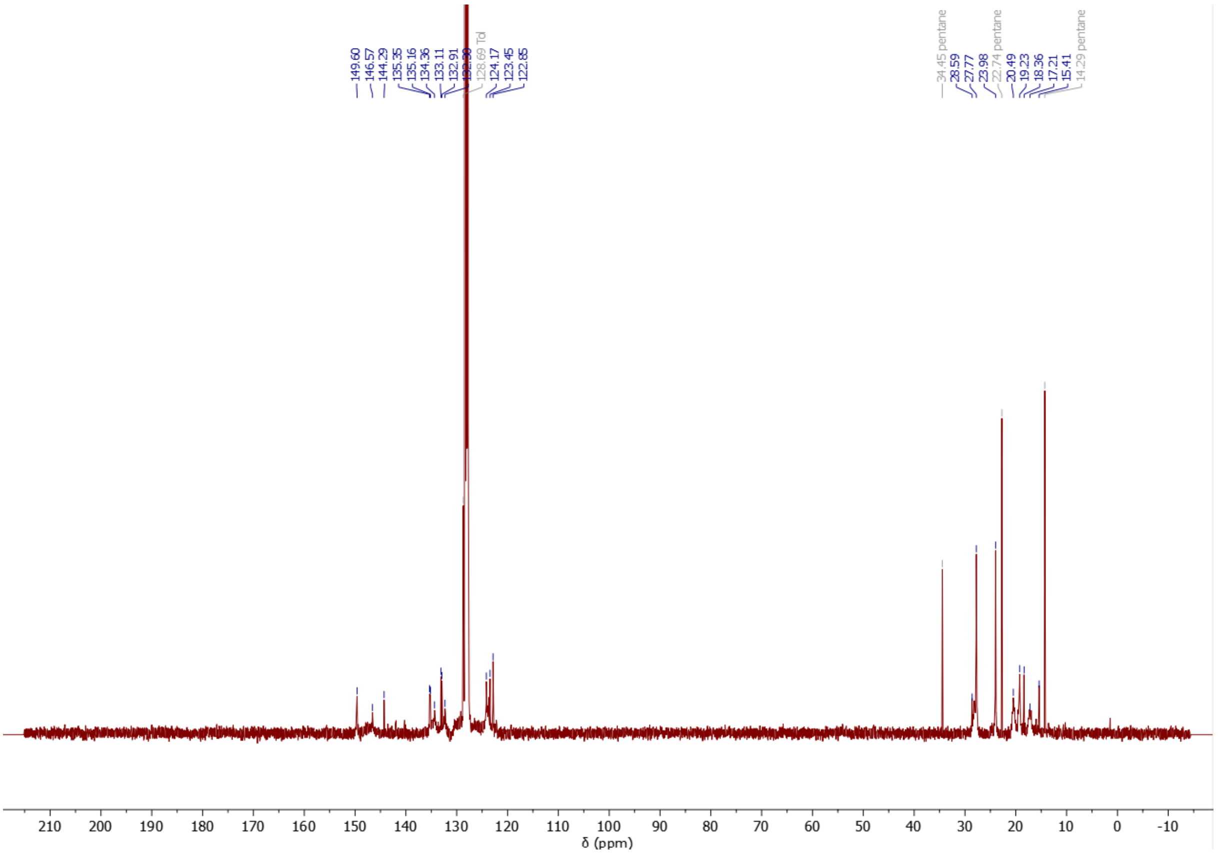

Figure S62. ^{13}C NMR spectrum of compound **2-Ph** as a solution in C_6D_6 at ambient temperature.

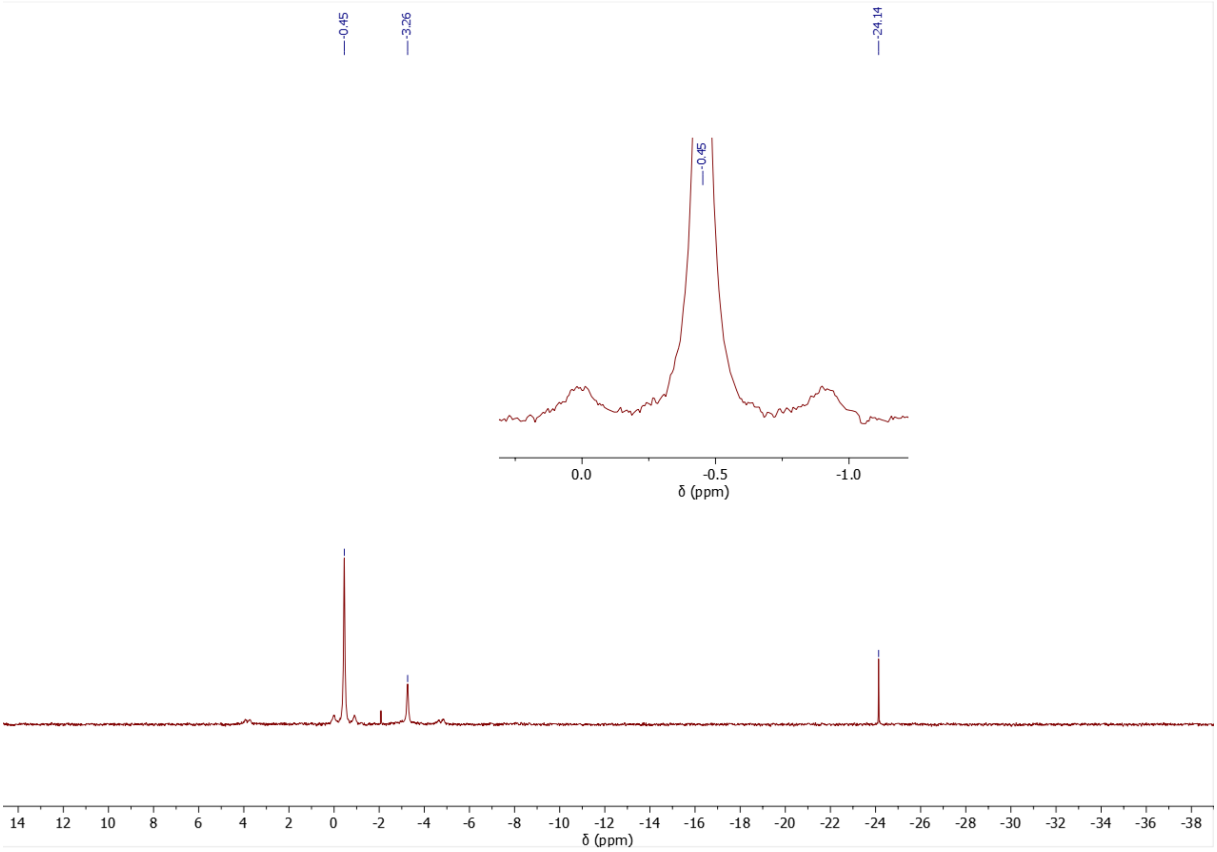

Figure S63. ^{31}P NMR spectrum of compound **2-Ph** as a solution in C_6D_6 at ambient temperature.

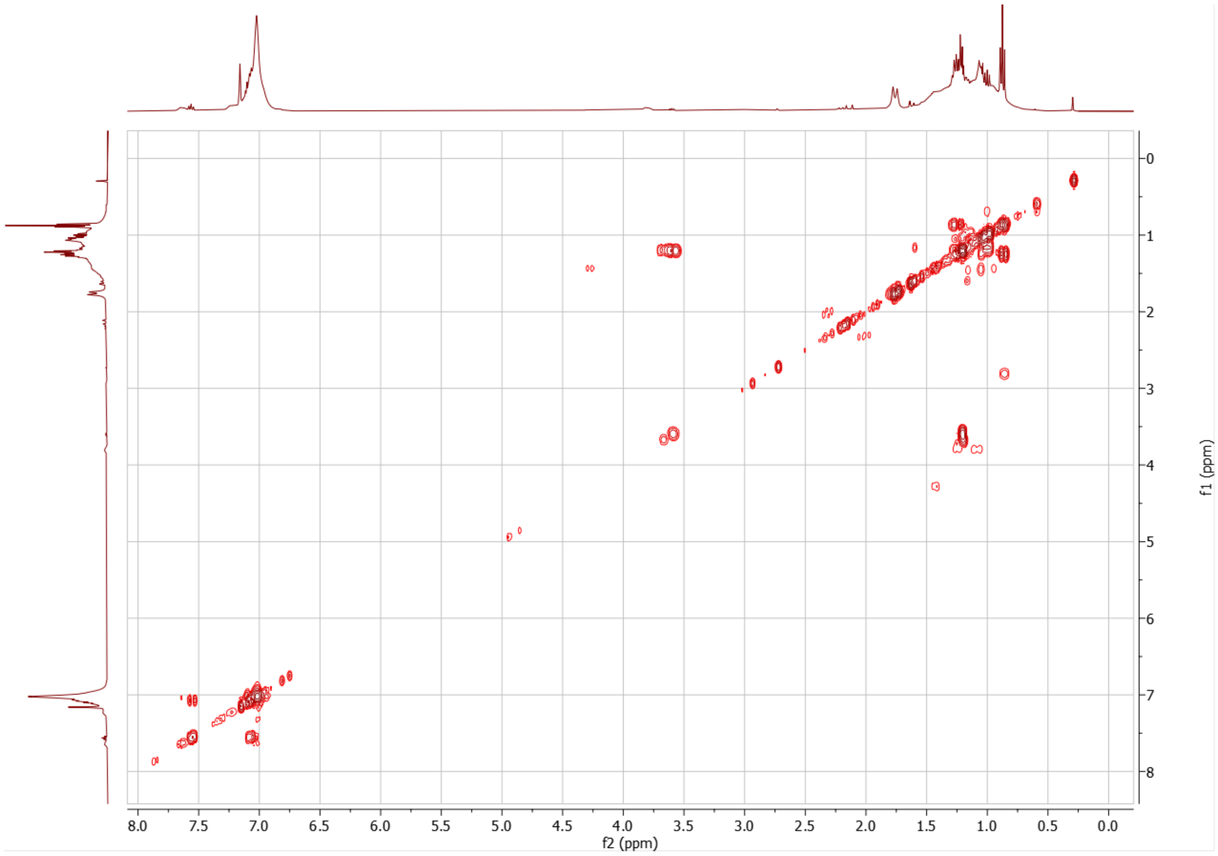

Figure S64. COSY NMR spectrum of compound **2-Ph** as a solution in C_6D_6 at ambient temperature.

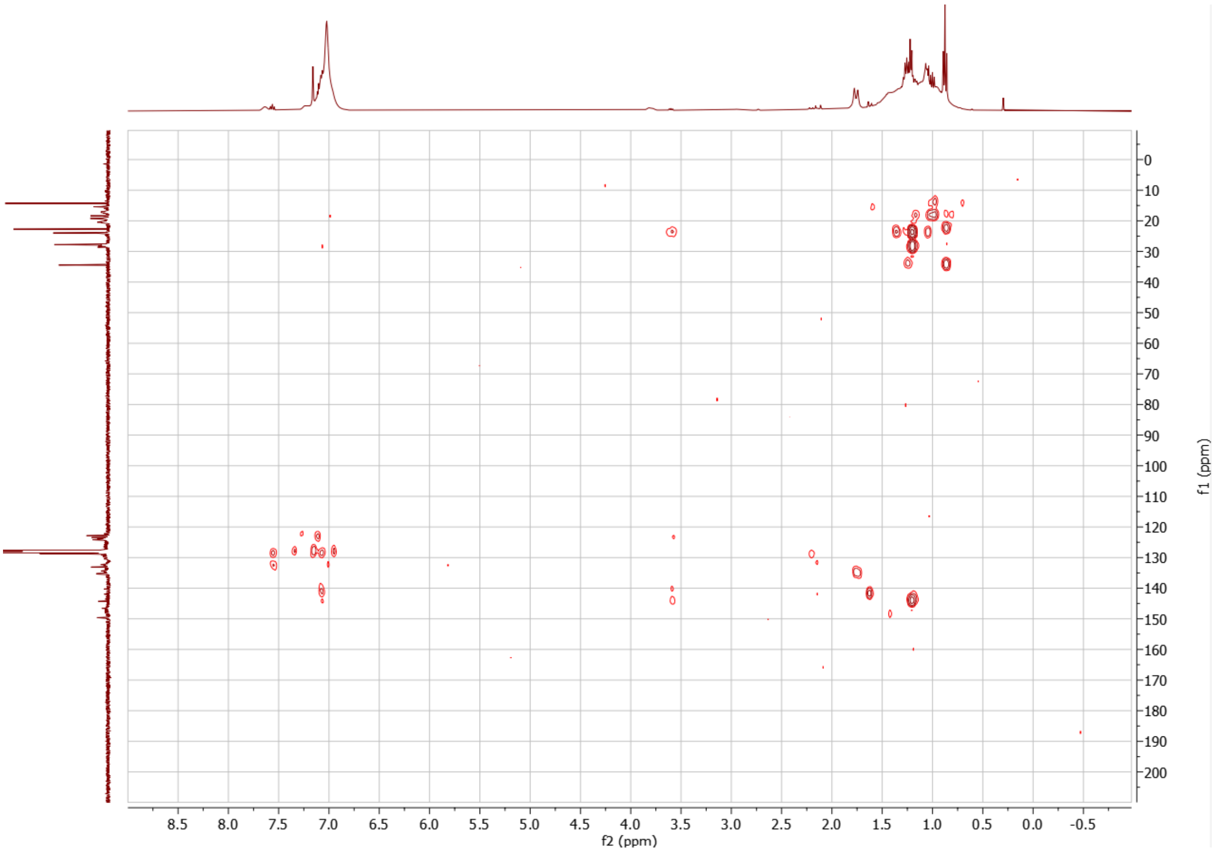

Figure S65. HMBC NMR spectrum of compound **2-Ph** as a solution in C_6D_6 at ambient temperature.

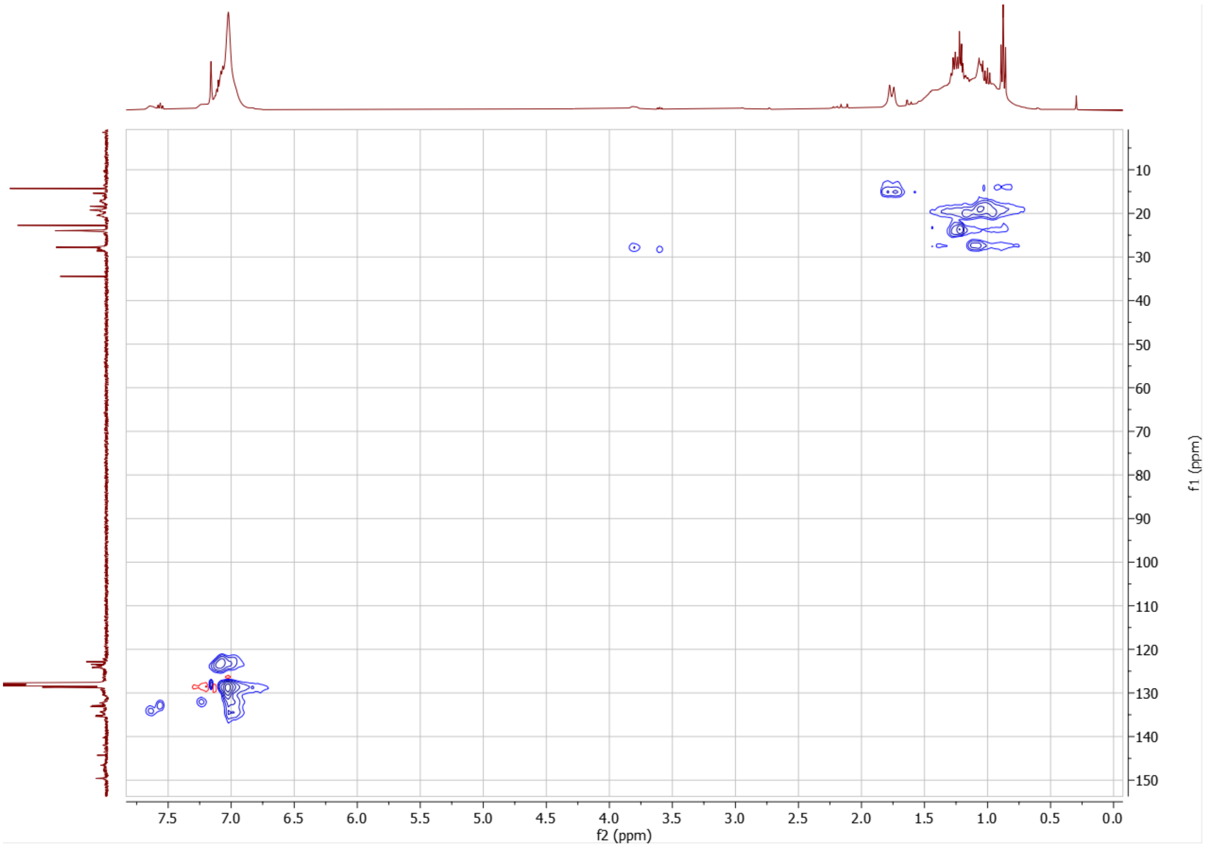

Figure S66. HSQC NMR spectrum of compound **2-Ph** as a solution in C_6D_6 at ambient temperature.

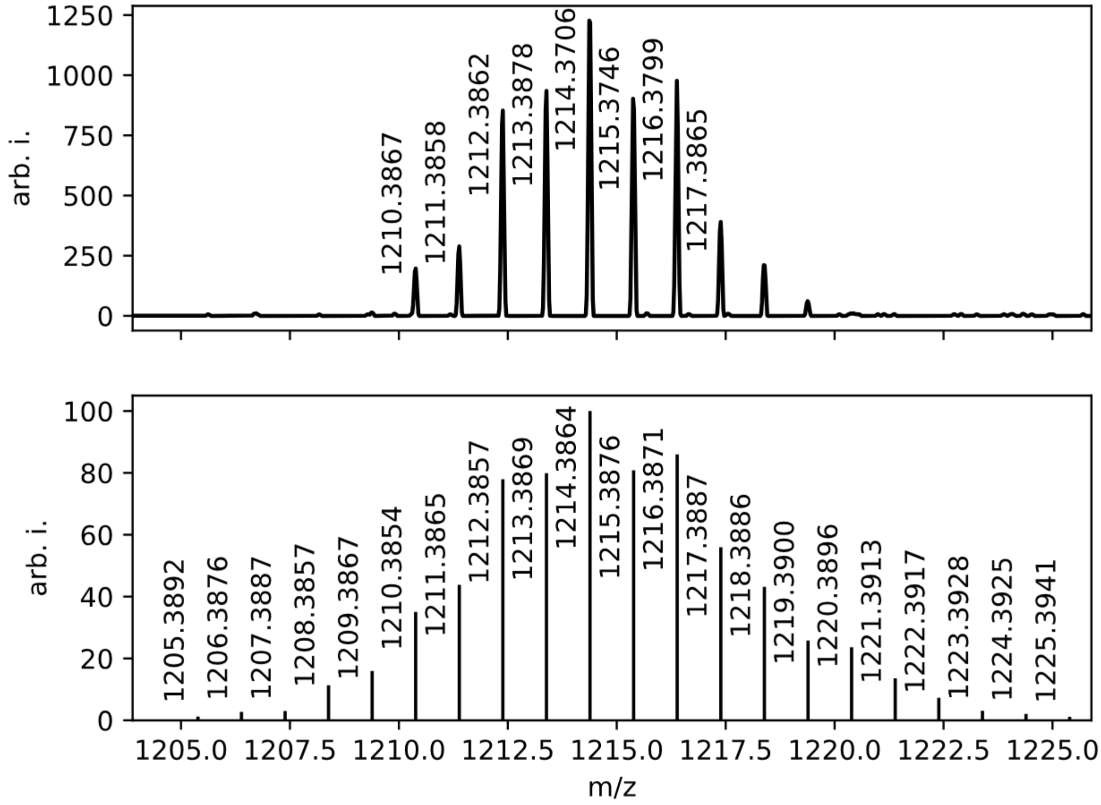

Figure S67. Cutout from LIFDI/MS of compound **2-Ph**; Top. found MS for $[M]^+$; Bottom. Calculated MS spectrum of $[M]^+$.

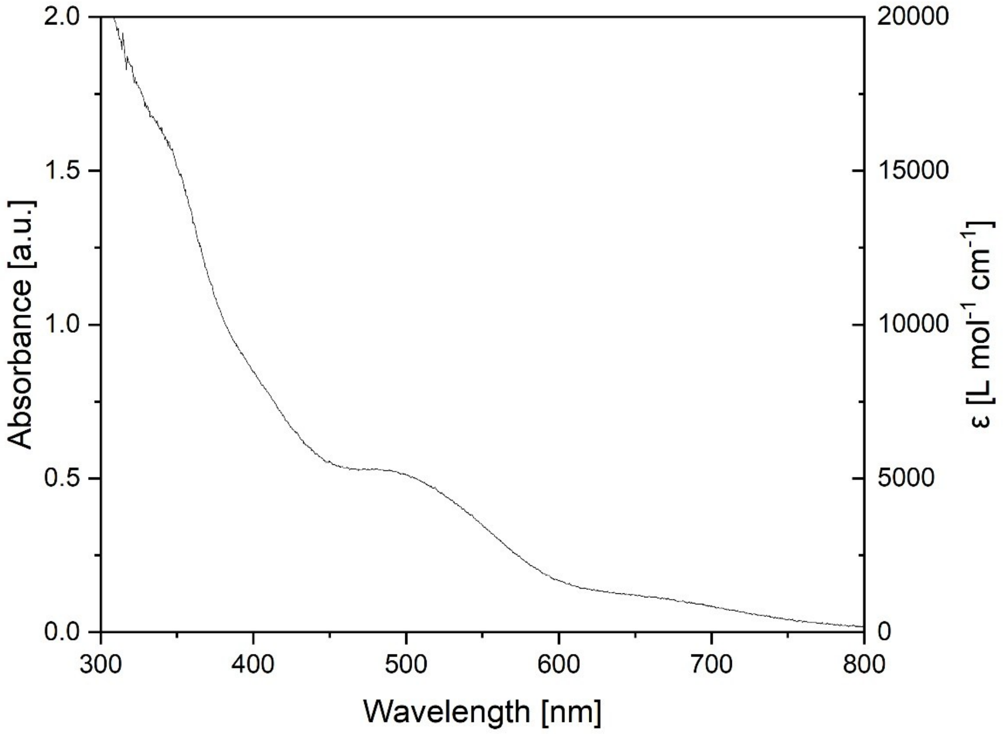

Figure S68. UV/vis spectrum of a 1.0x10^{-4} M solution of compound **2-Ph** in toluene at ambient temperature.

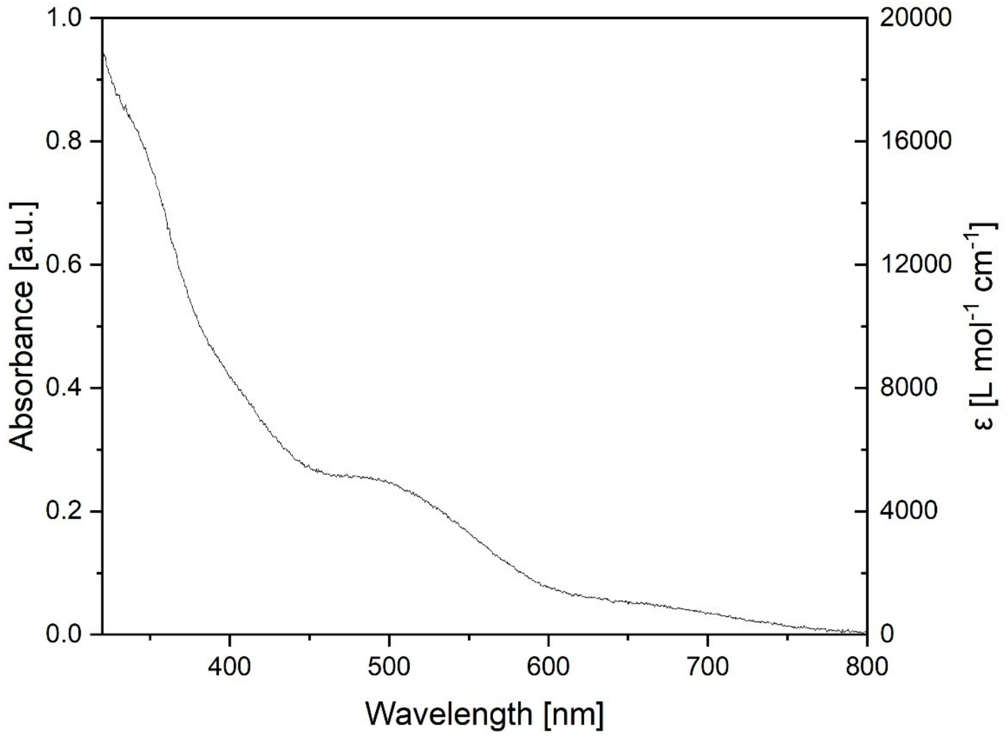

Figure S69. UV/vis spectrum of a 5.0x10^{-5} M solution of compound **2-Ph** in toluene at ambient temperature.

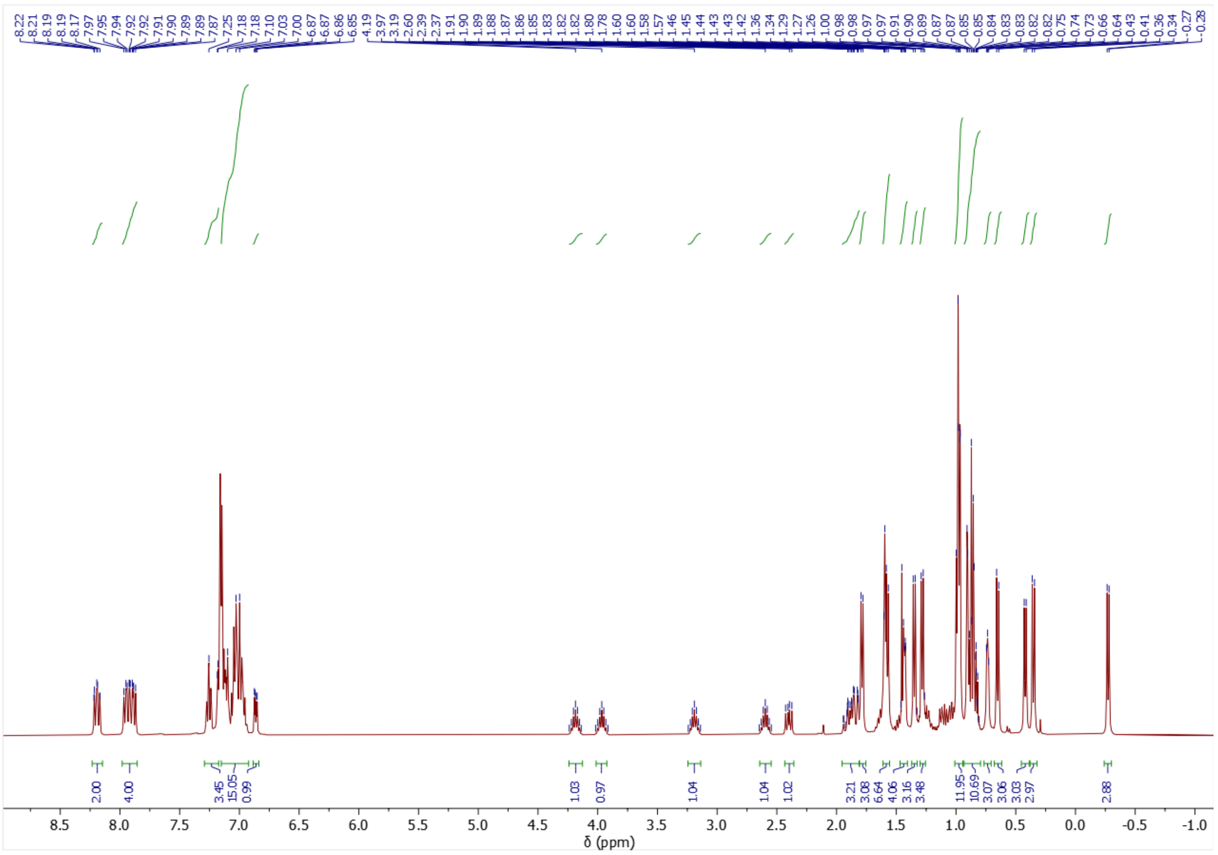

Figure S70. ^{1}H NMR spectrum of compound **3** as a solution in C_6D_6 at ambient temperature.

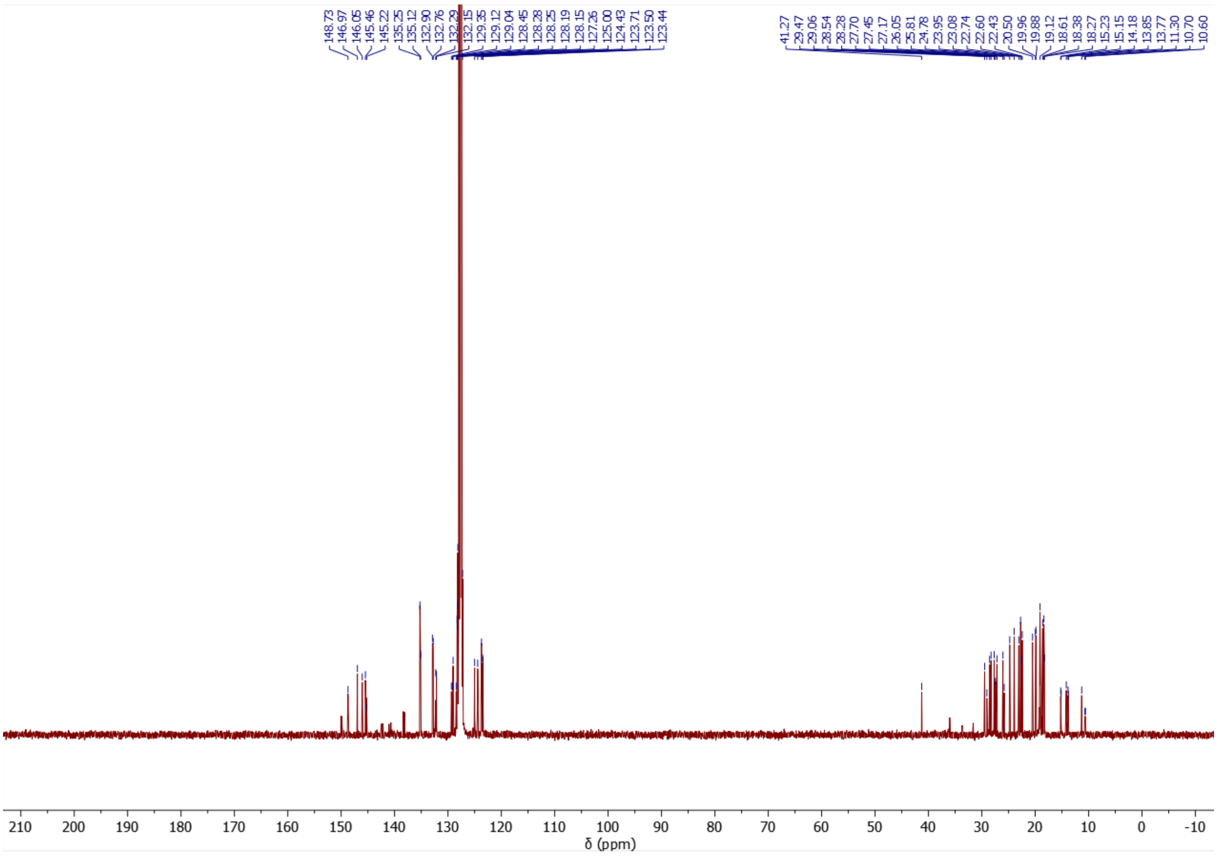

Figure S71. ^{13}C NMR spectrum of compound **3** as a solution in C_6D_6 at ambient temperature.

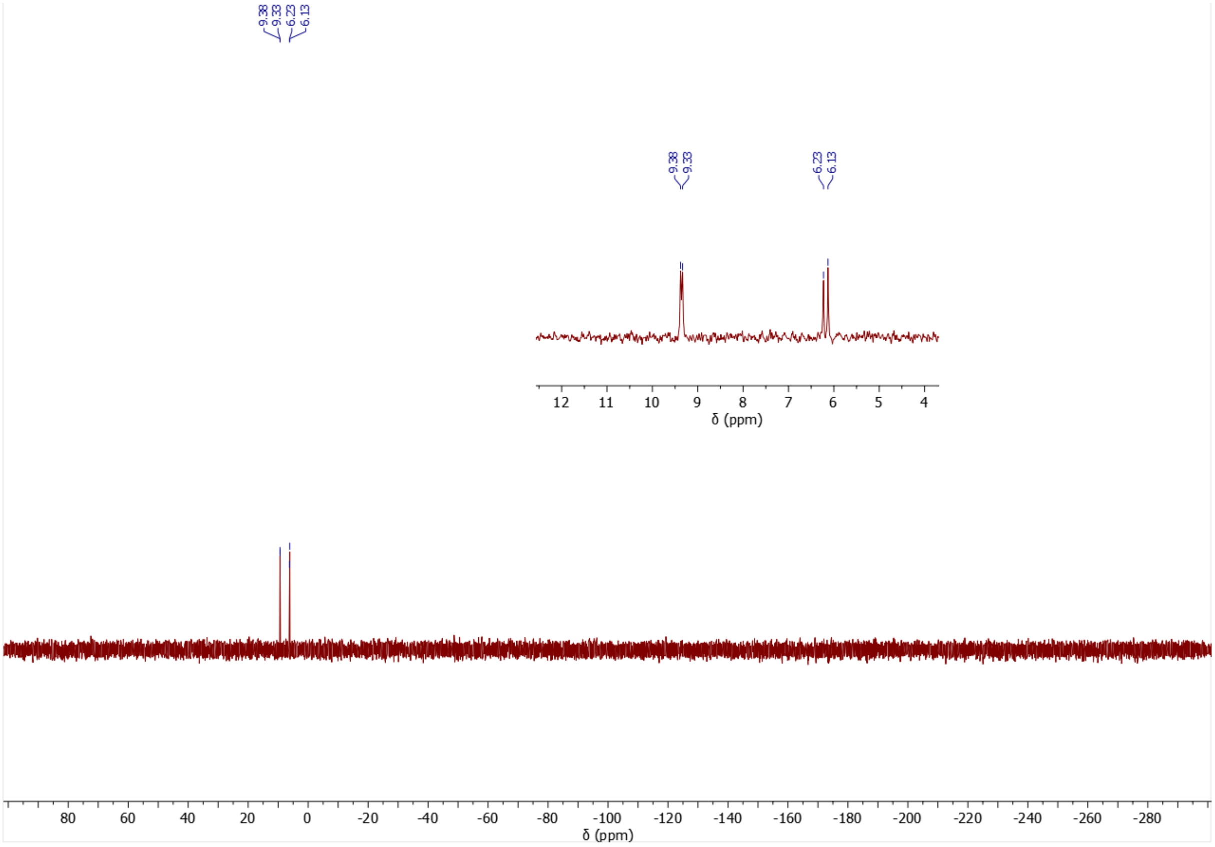

Figure S72. ^{29}Si NMR spectrum of compound **3** as a solution in C_6D_6 at ambient temperature.

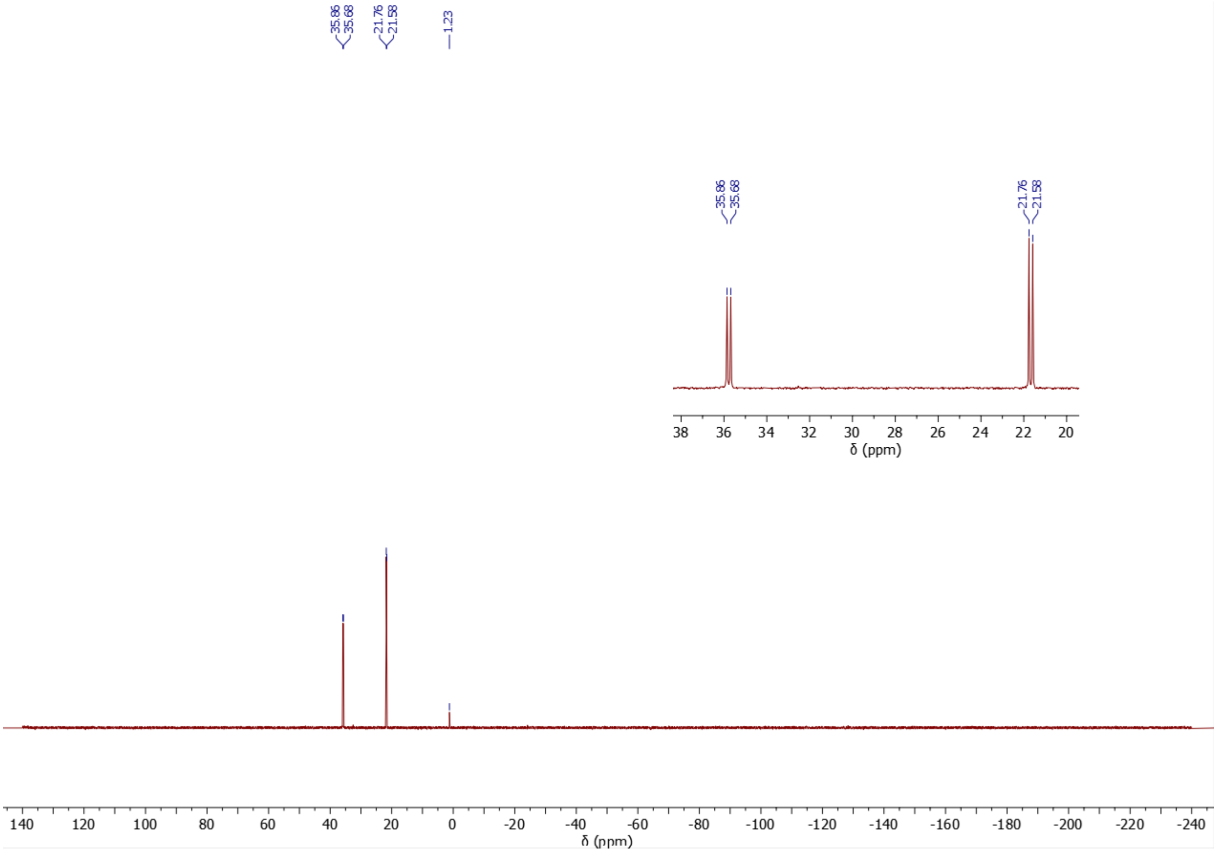

Figure S73. ^{31}P NMR spectrum of compound **3** as a solution in C_6D_6 at ambient temperature.

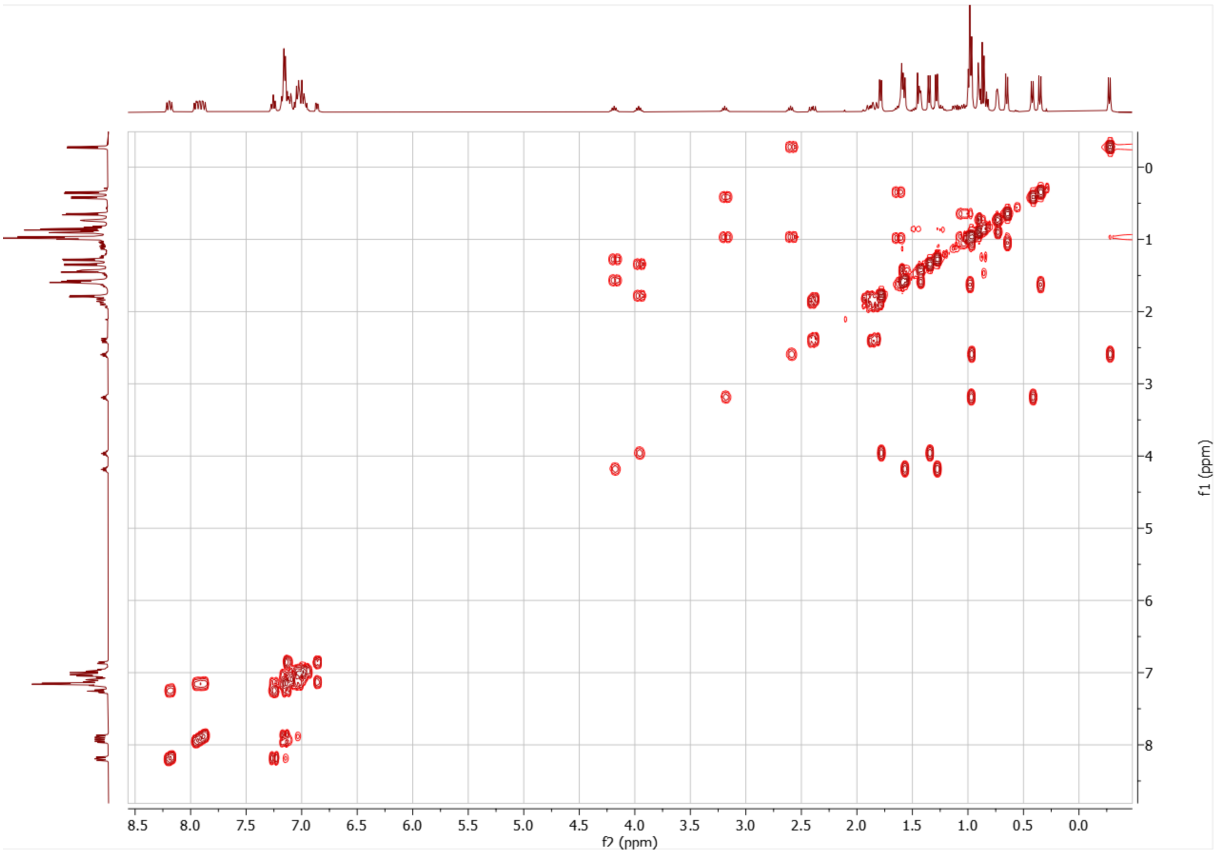

Figure S74. COSY NMR spectrum of compound **3** as a solution in C_6D_6 at ambient temperature.

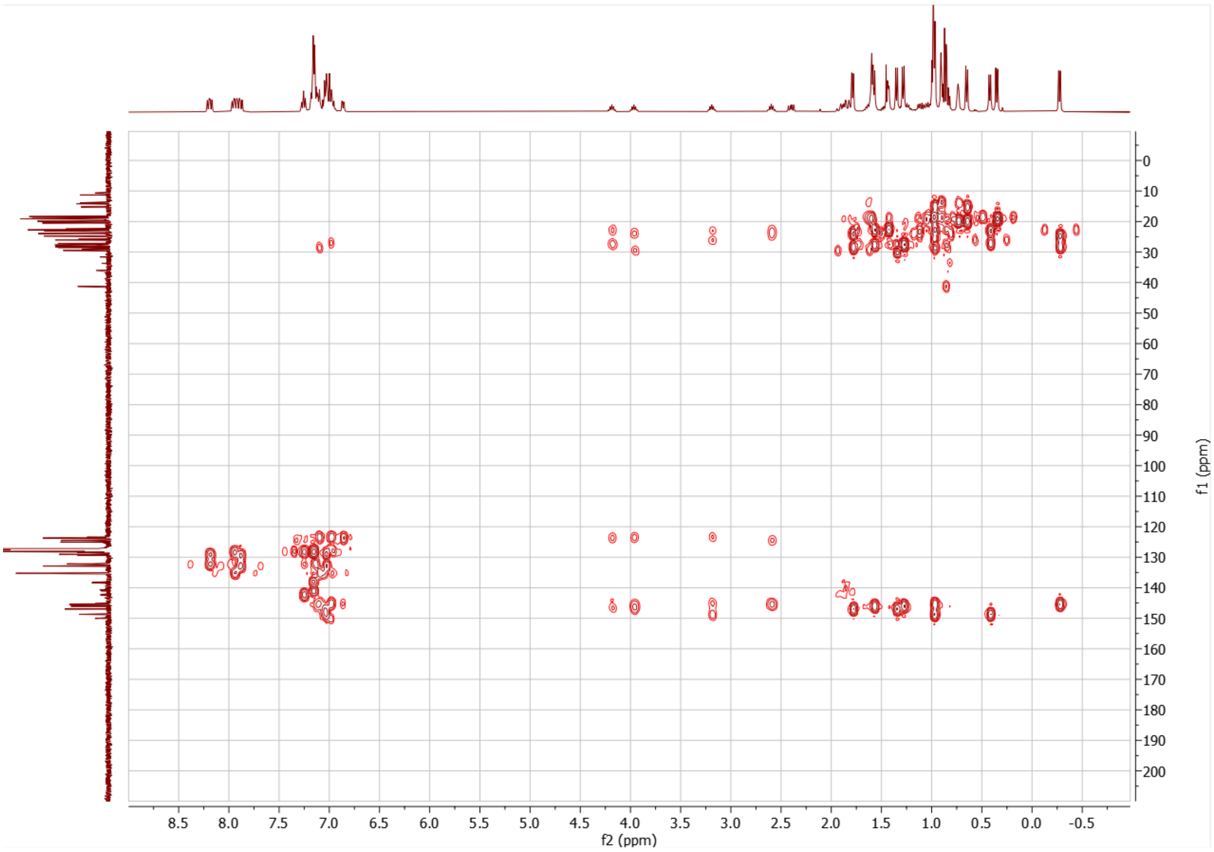

Figure S75. HMBC NMR spectrum of compound **3** as a solution in C_6D_6 at ambient temperature.

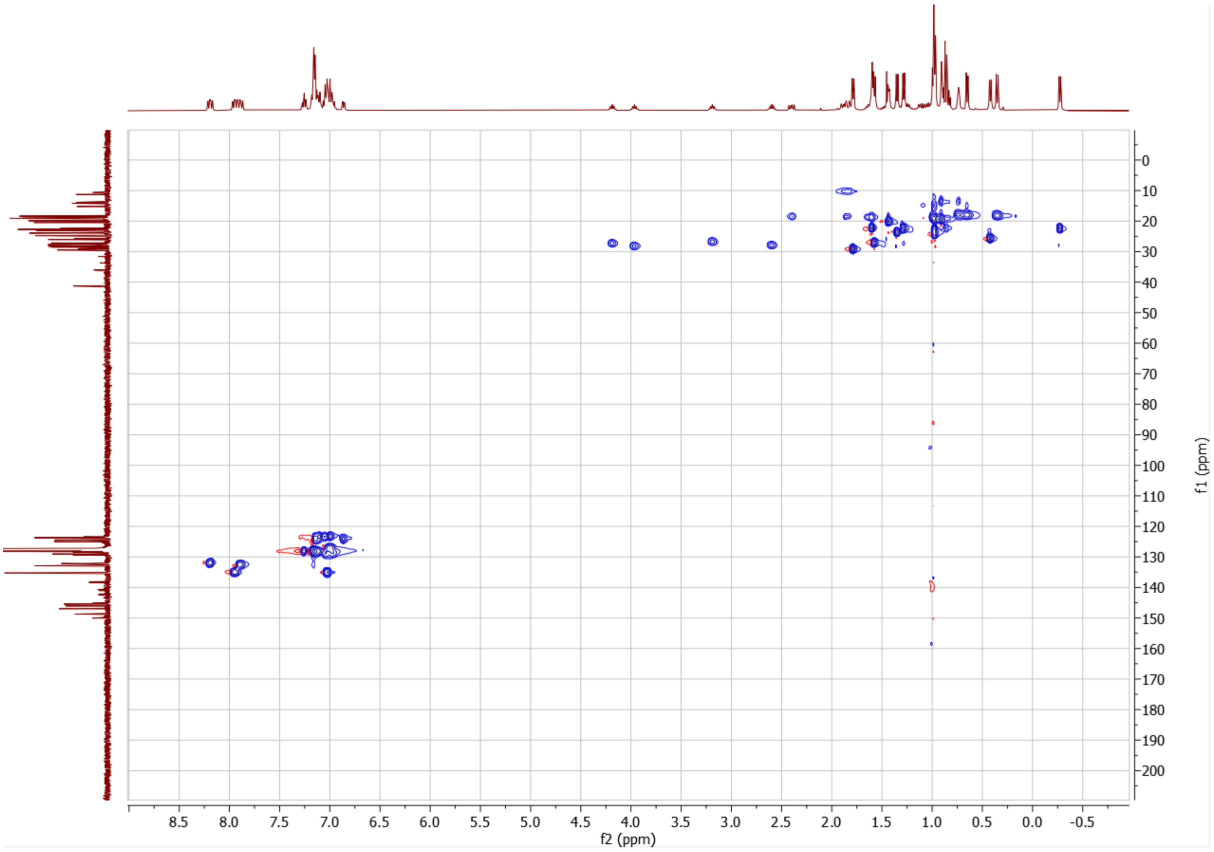

Figure S76. HSQC NMR spectrum of compound **3** as a solution in C_6D_6 at ambient temperature.

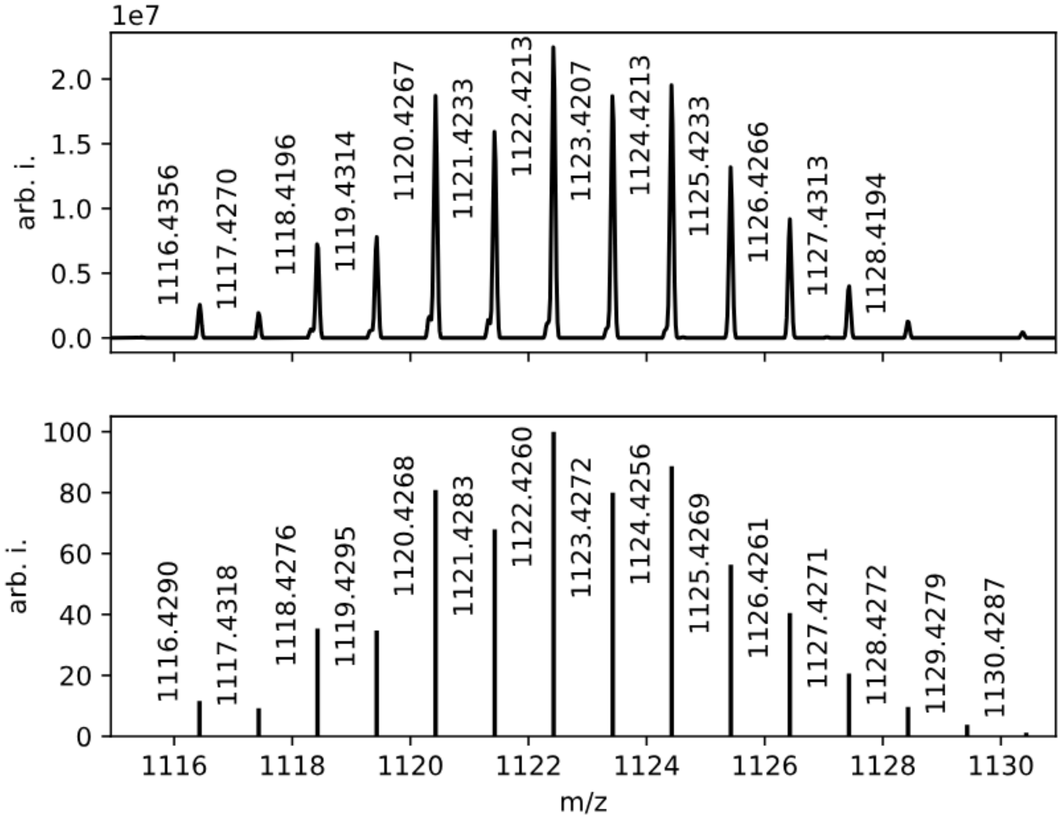

Figure S77. Cutout from LIFDI/MS of compound **3**; Top. found MS for [M]$^+$; Bottom. Calculated MS spectrum of [M]$^+$.

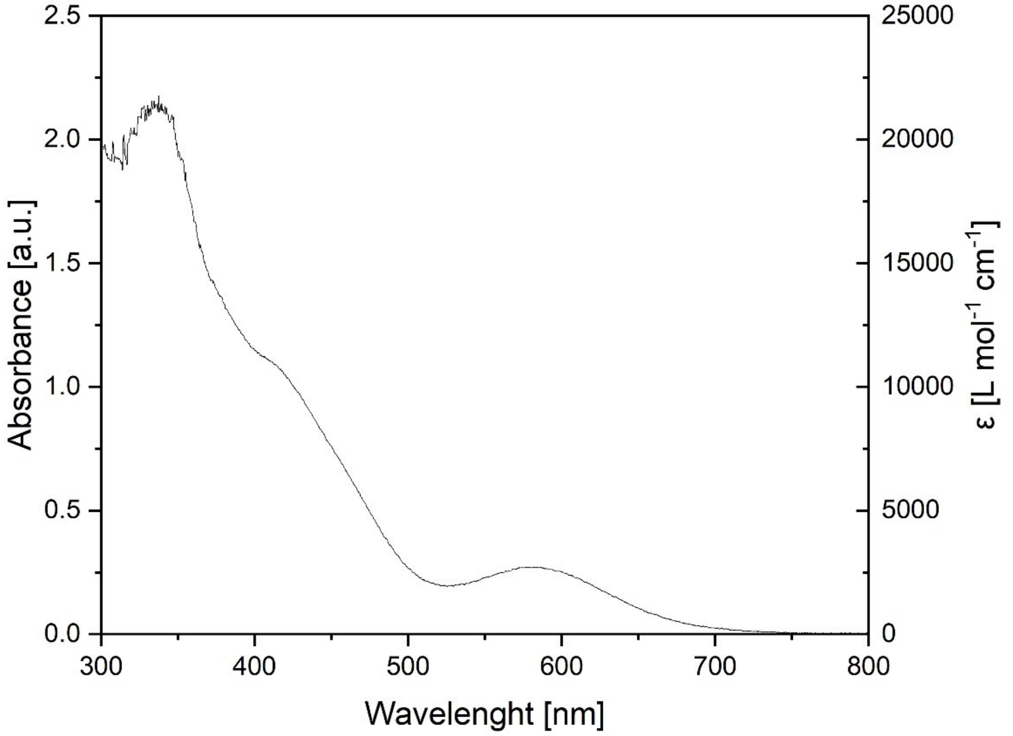

Figure S78. UV/vis spectrum of a 1.0x10^{-4} M solution of compound **3** in toluene at ambient temperature.

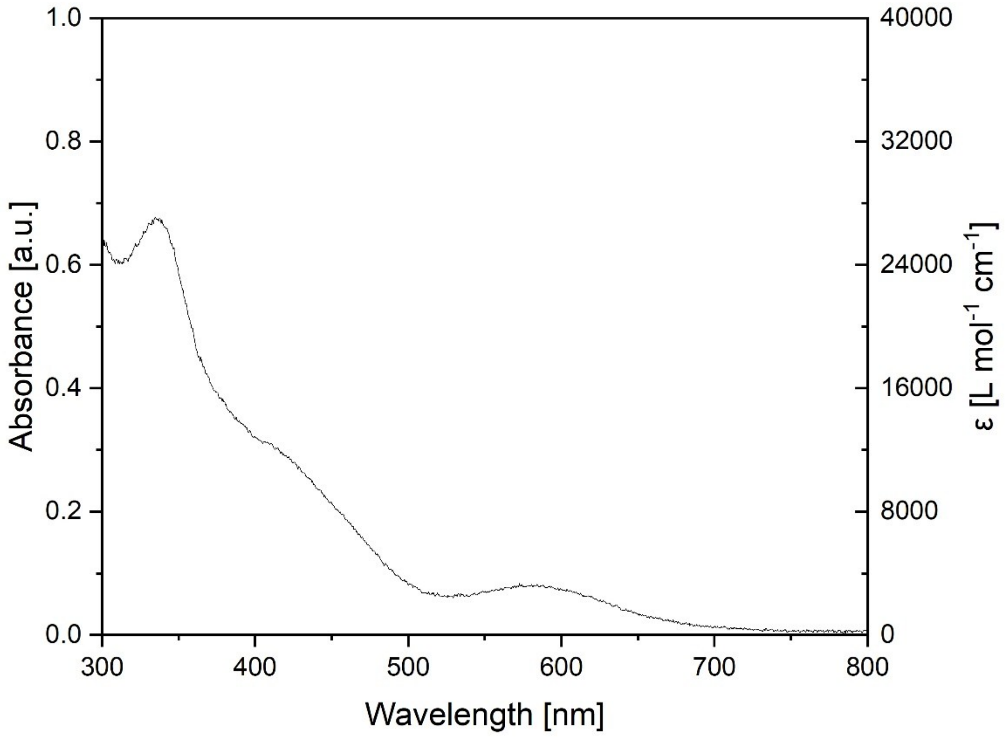

Figure S79. UV/vis spectrum of a 2.5x10^{-5} M solution of compound **3** in toluene at ambient temperature.

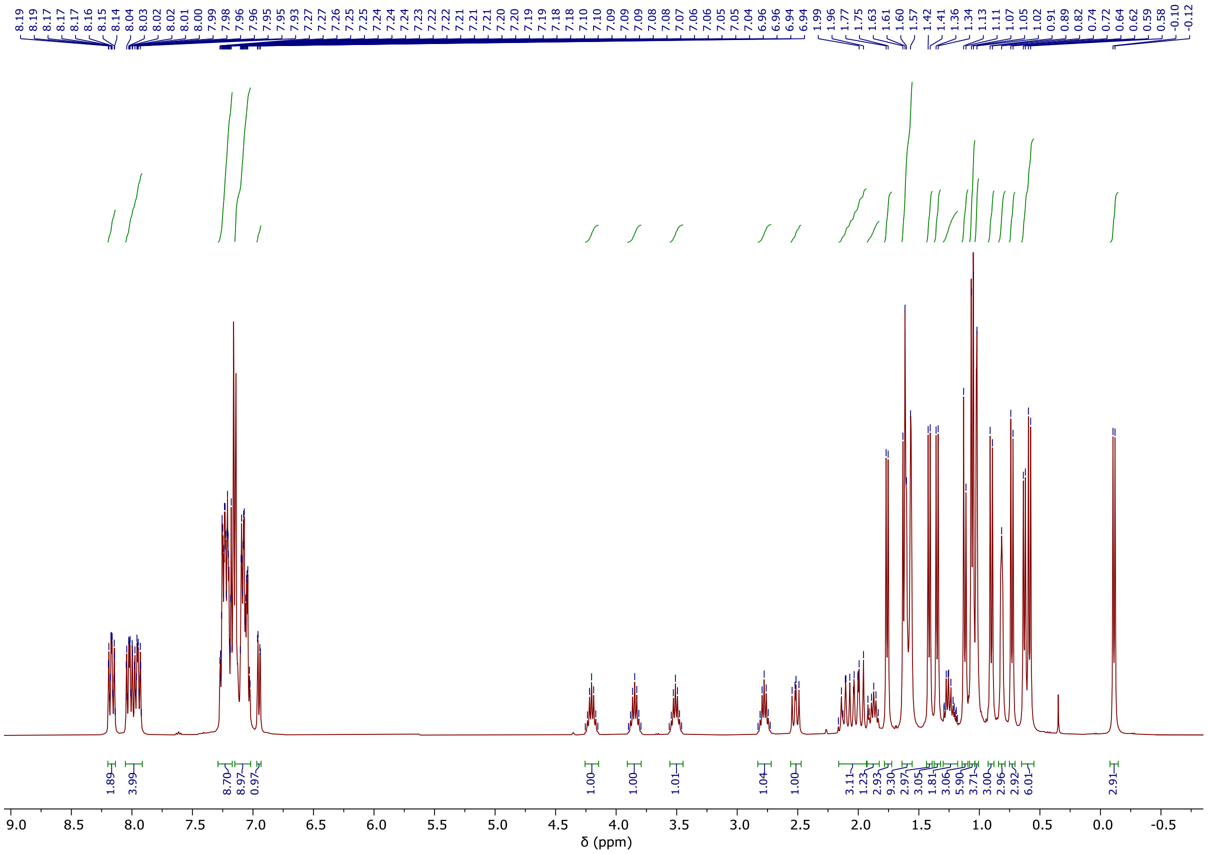

Figure S80. ^{1}H NMR spectrum of compound **4** as a solution in C_6D_6 at ambient temperature.

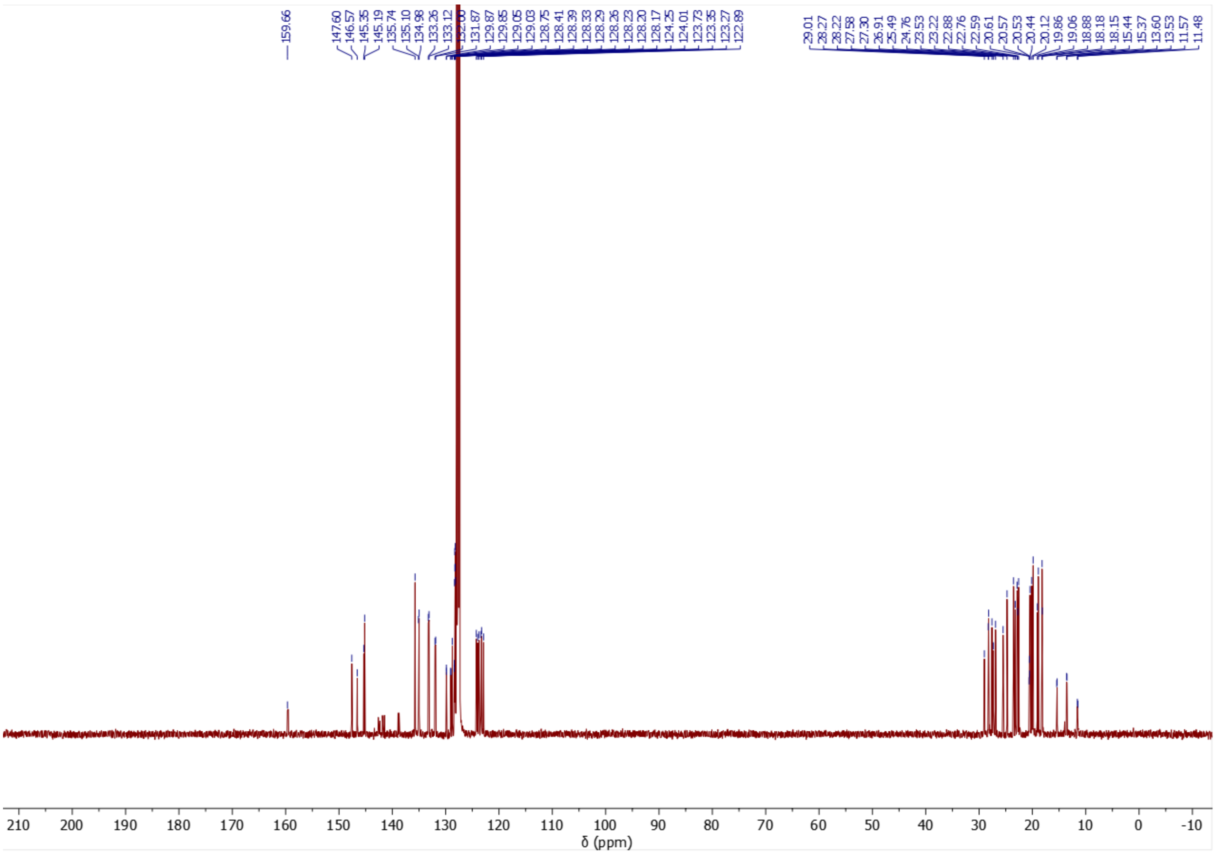

Figure S81. ^{13}C NMR spectrum of compound **4** as a solution in C_6D_6 at ambient temperature.

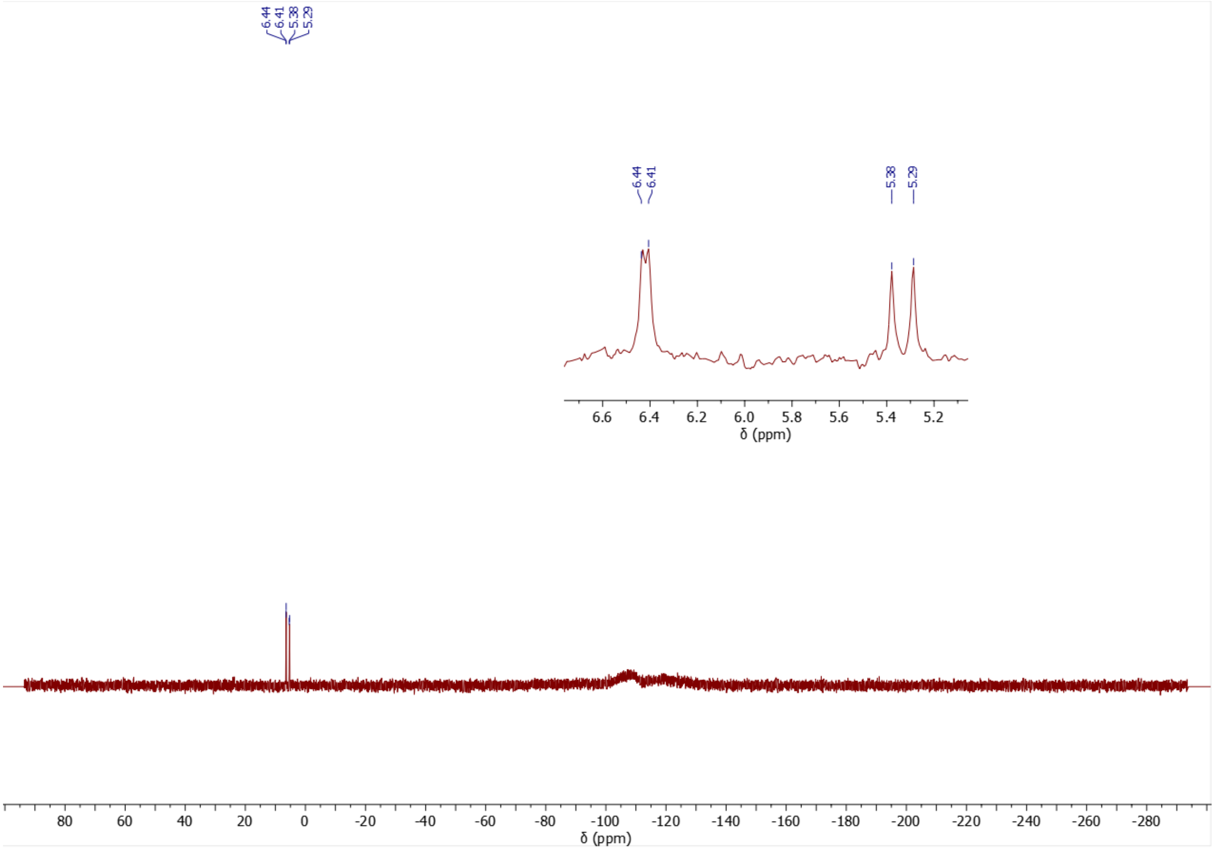

Figure S82. ^{29}Si NMR spectrum of compound **4** as a solution in C_6D_6 at ambient temperature.

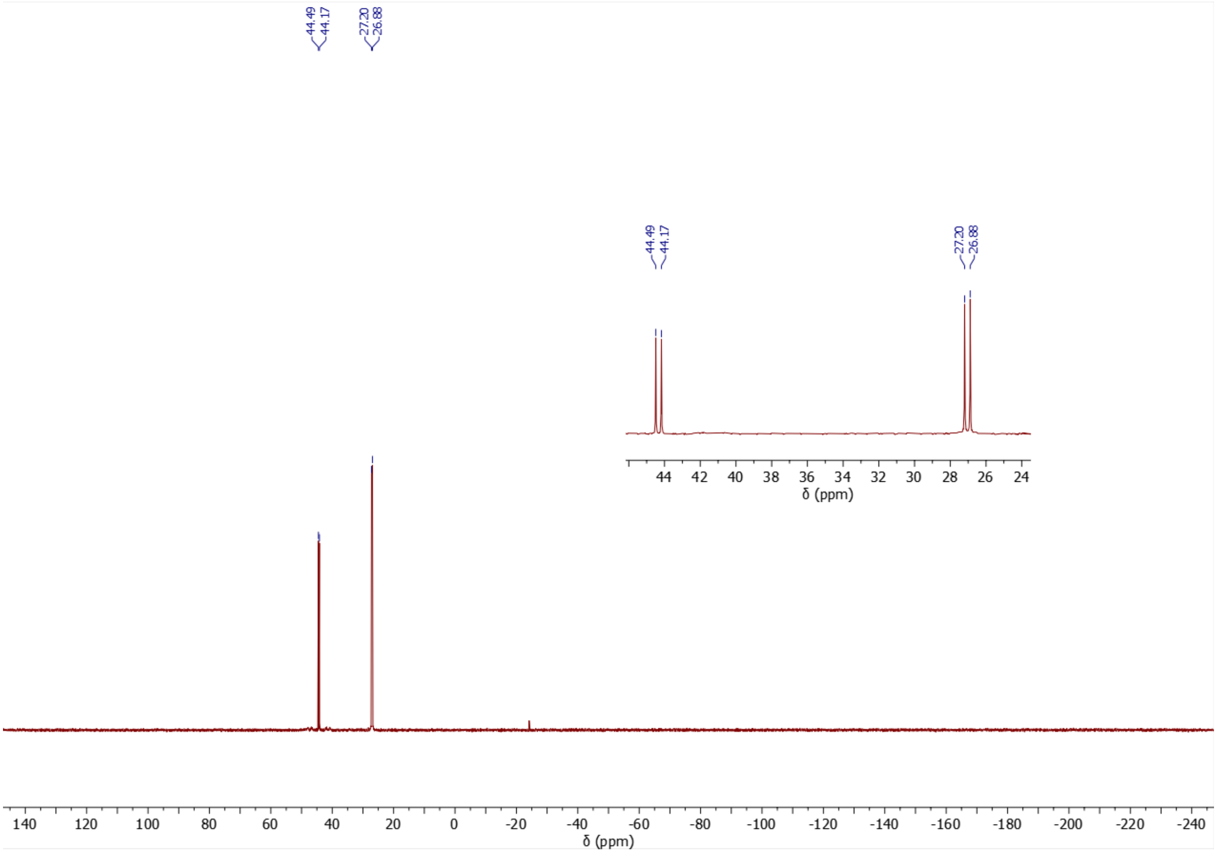

Figure S83. ^{31}P NMR spectrum of compound **4** as a solution in C_6D_6 at ambient temperature.

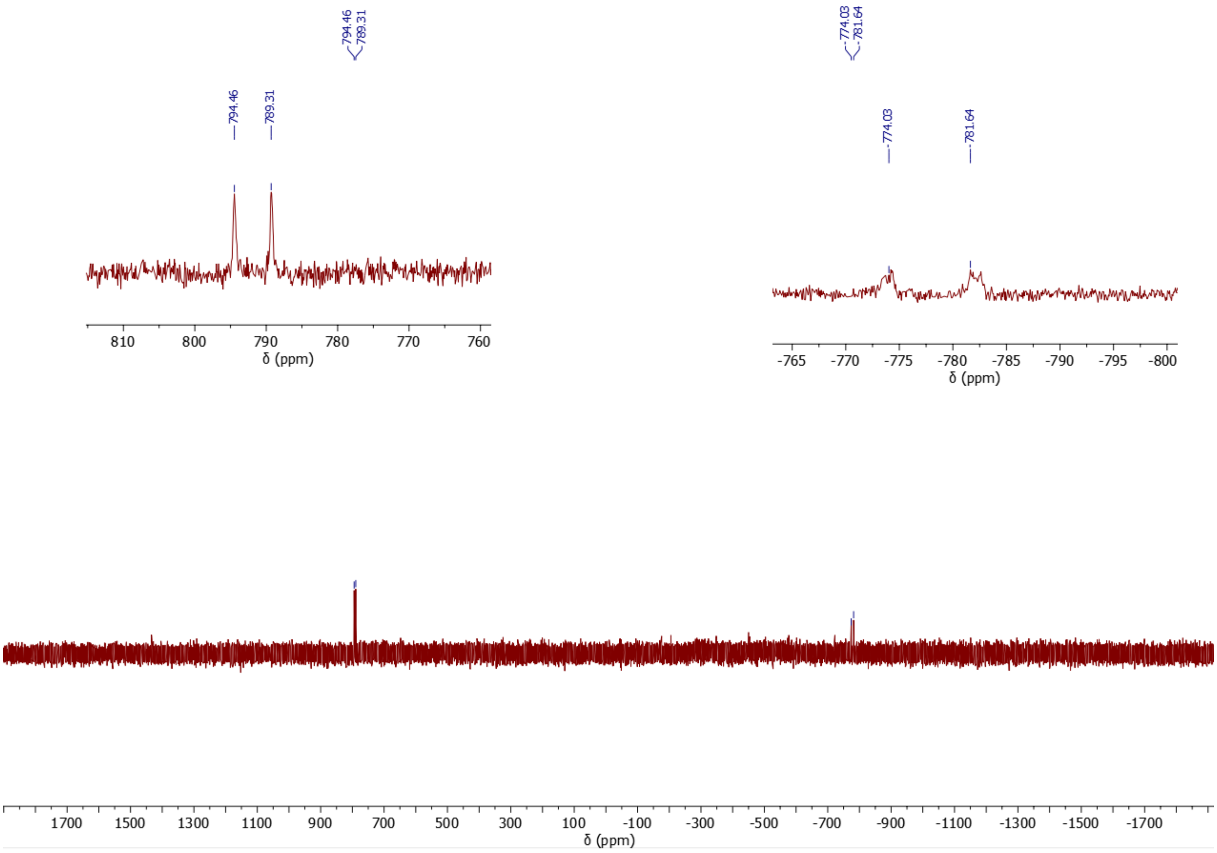

Figure S84. ^{119}Sn NMR spectrum of compound **4** as a solution in C_6D_6 at ambient temperature.

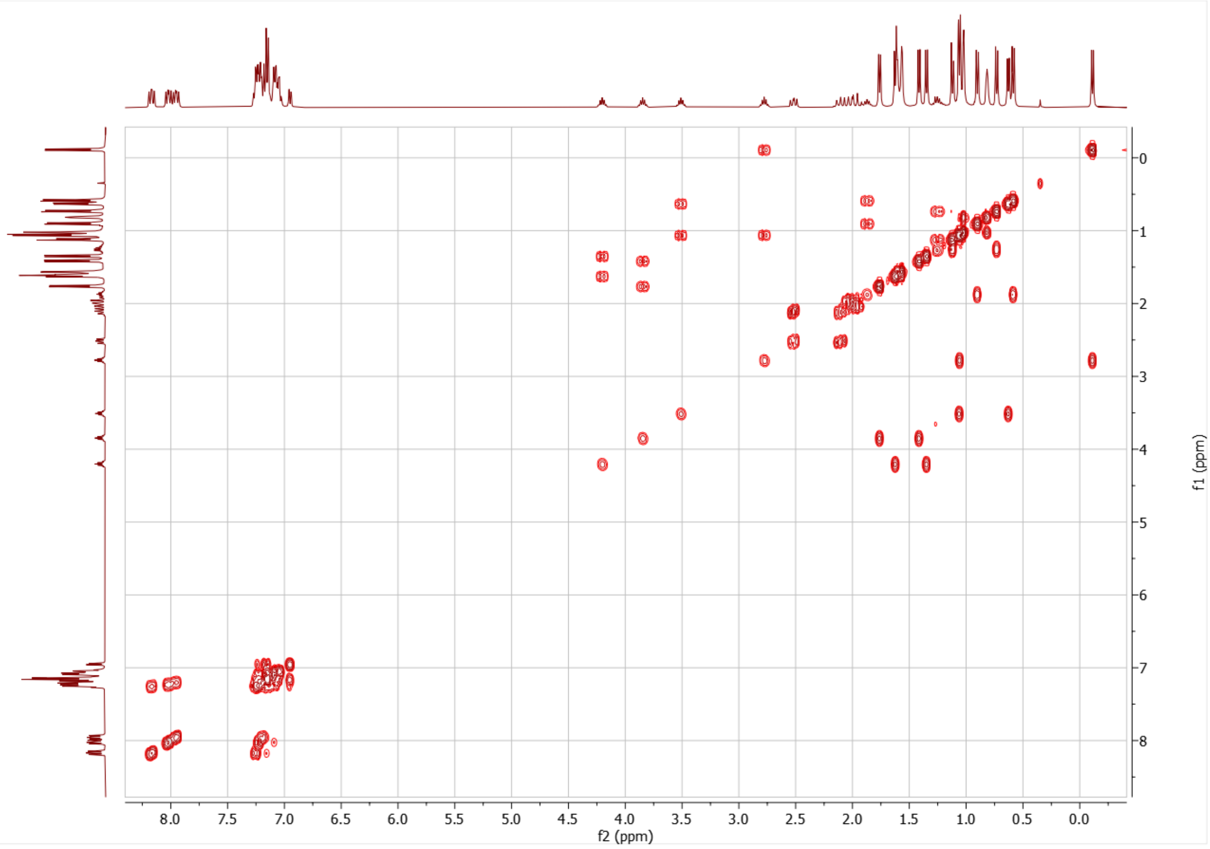

Figure S85. COSY NMR spectrum of compound **4** as a solution in C_6D_6 at ambient temperature.

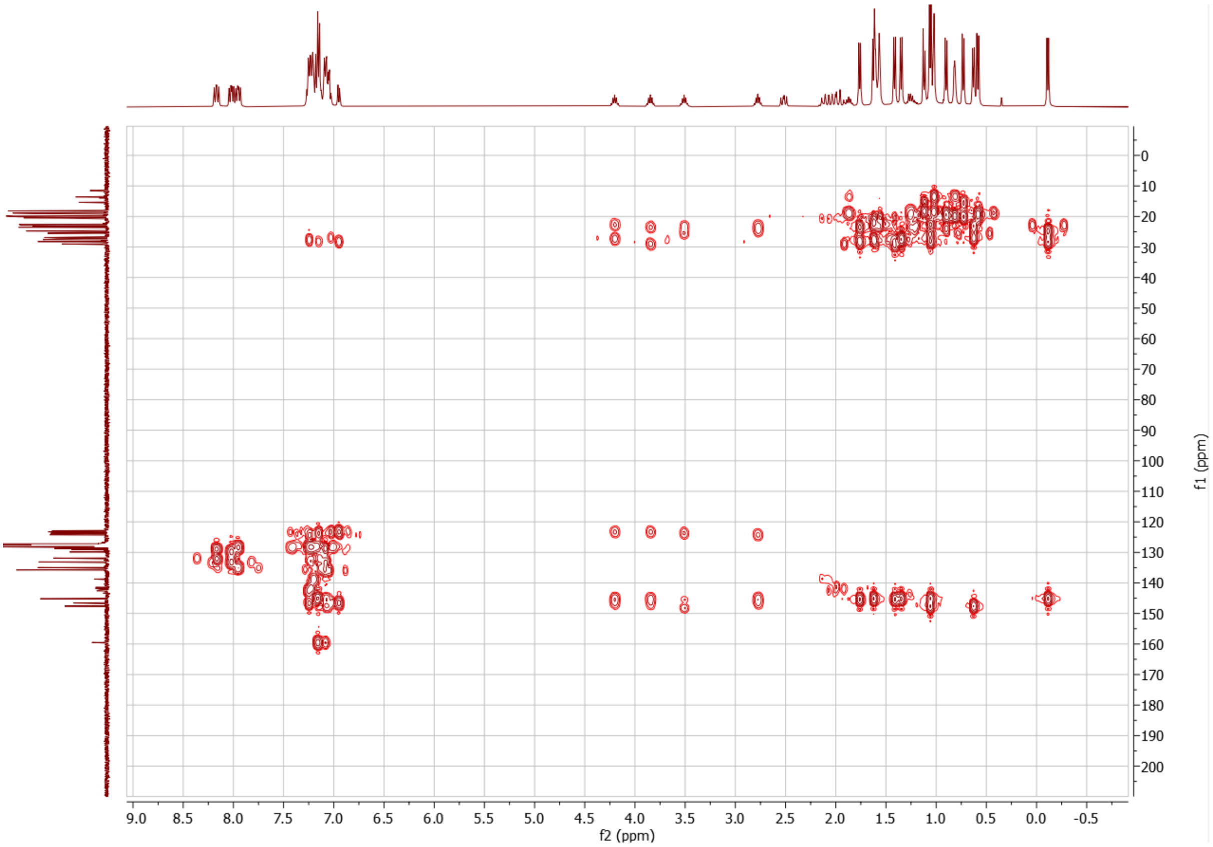

Figure S86. HMBC NMR spectrum of compound **4** as a solution in C_6D_6 at ambient temperature.

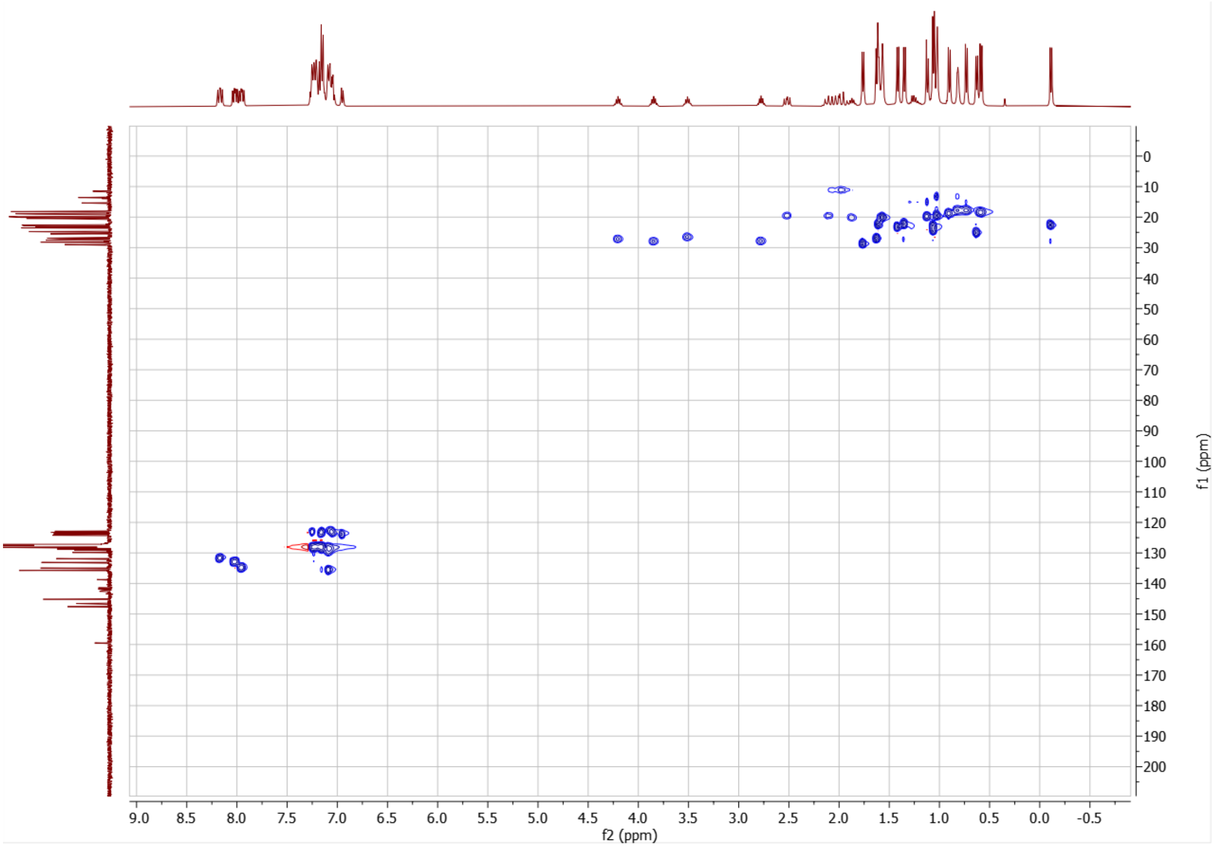

Figure S87. HSQC NMR spectrum of compound **4** as a solution in C_6D_6 at ambient temperature.

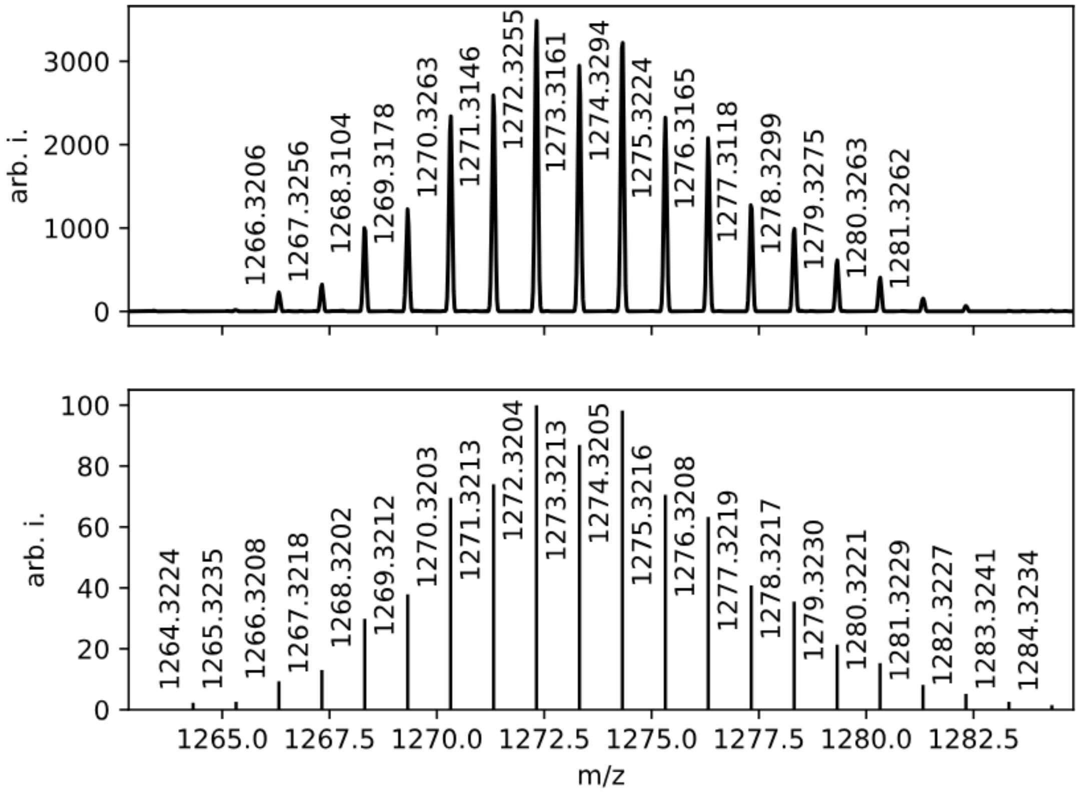

Figure S88. Cutout from LIFDI/MS of compound **4**; Top. found MS for $[M]^+$; Bottom. Calculated MS spectrum of $[M]^+$.

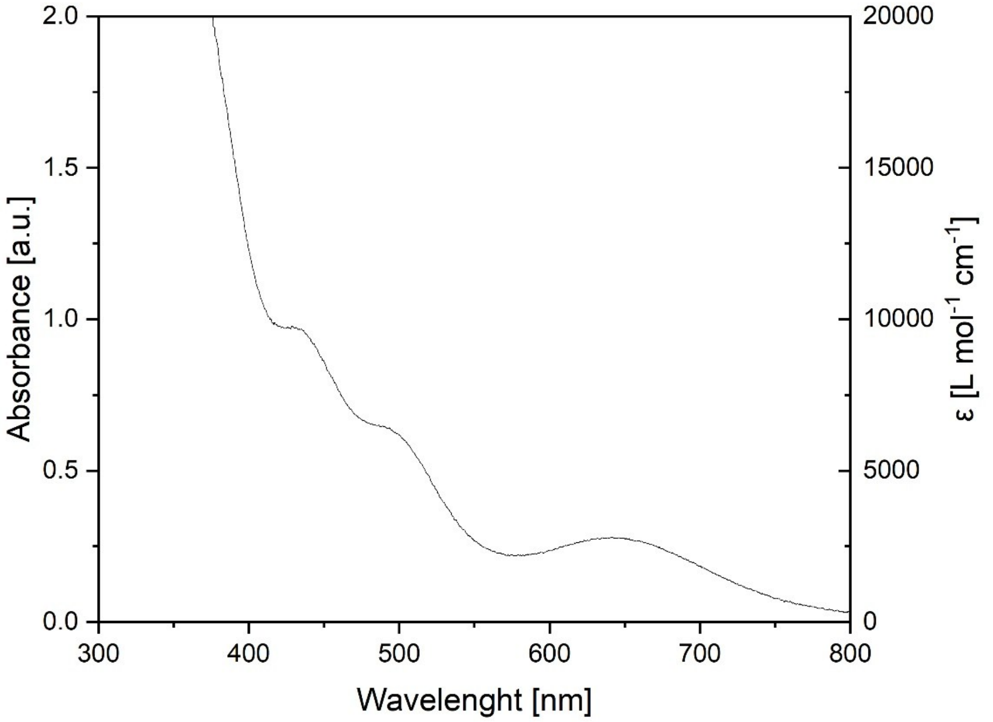

Figure S89. UV/vis spectrum of a 1.0x10^{-4} M solution of compound **4** in toluene at ambient temperature.

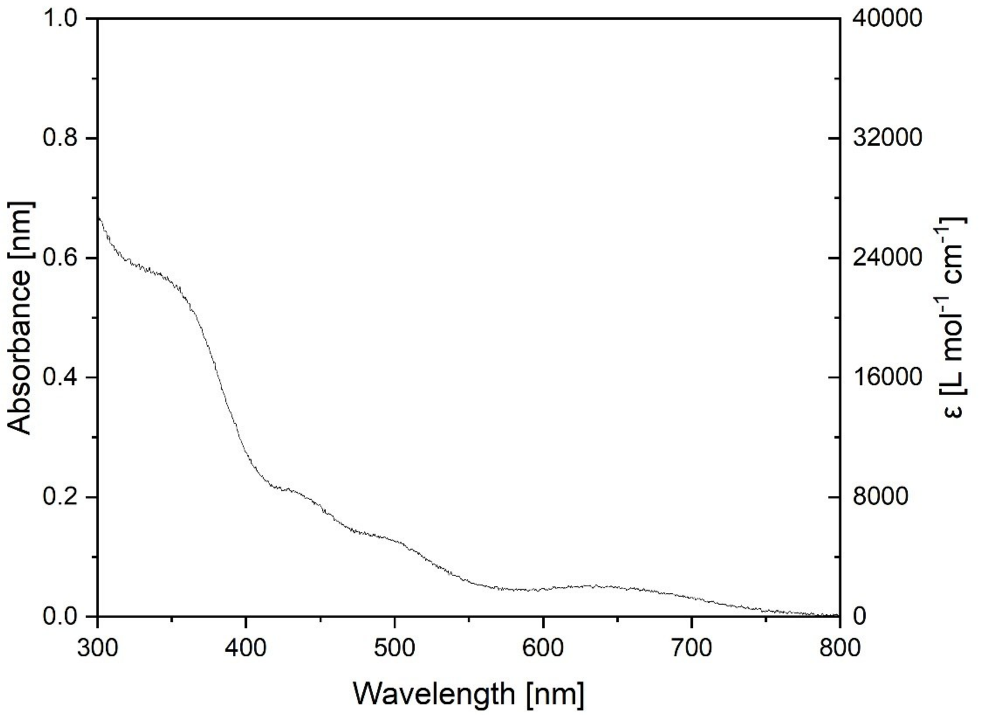

Figure S90. UV/vis spectrum of a 2.5x10^{-5} M solution of compound **4** in toluene at ambient temperature.

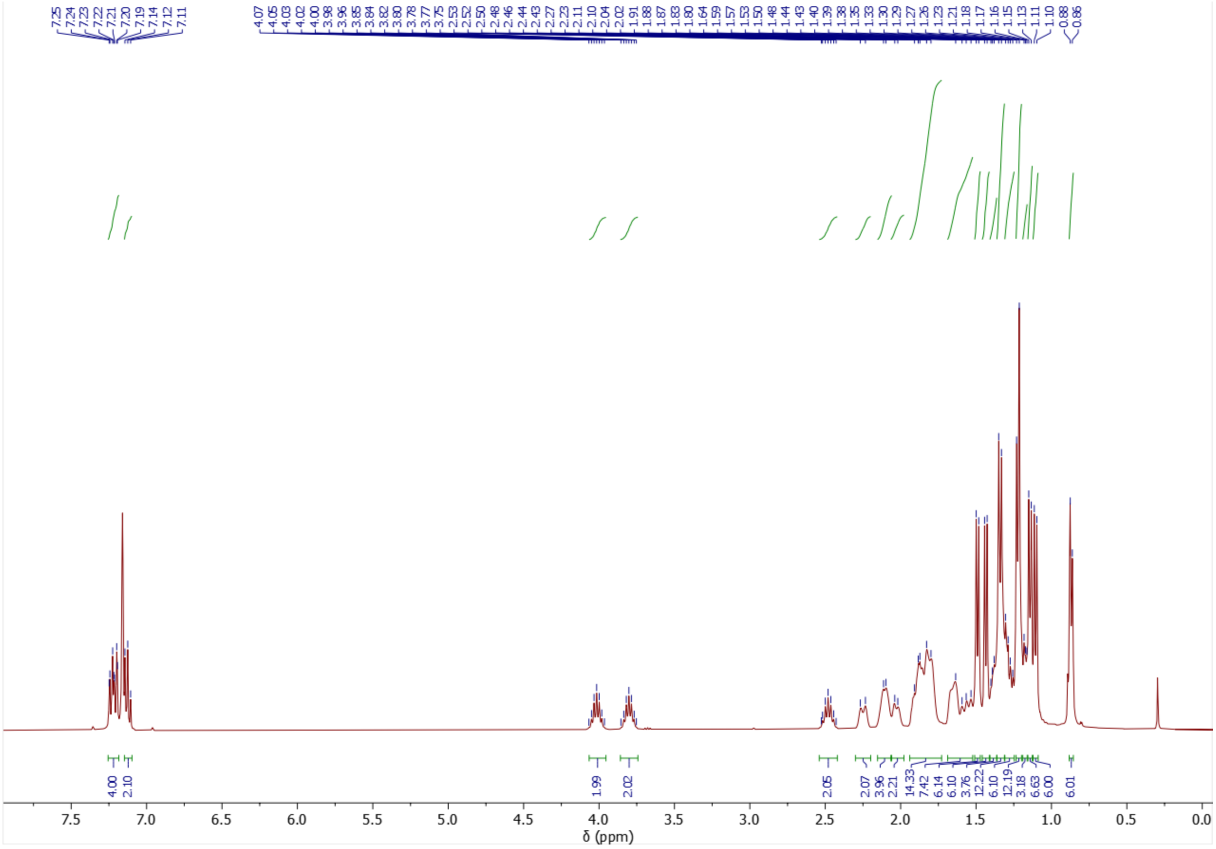

Figure S91. ^{1}H NMR spectrum of compound **5** as a solution in C_6D_6 at ambient temperature.

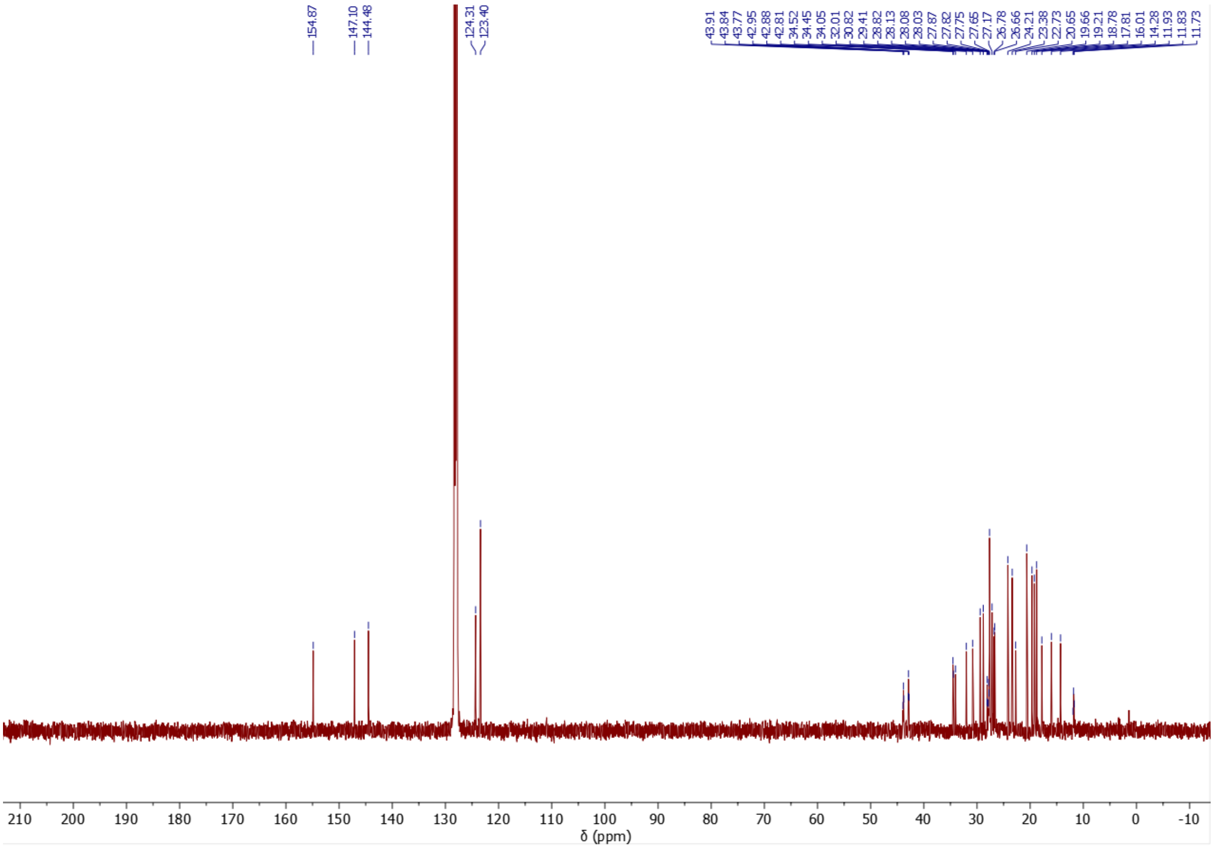

Figure S92. ^{13}C NMR spectrum of compound **5** as a solution in C_6D_6 at ambient temperature.

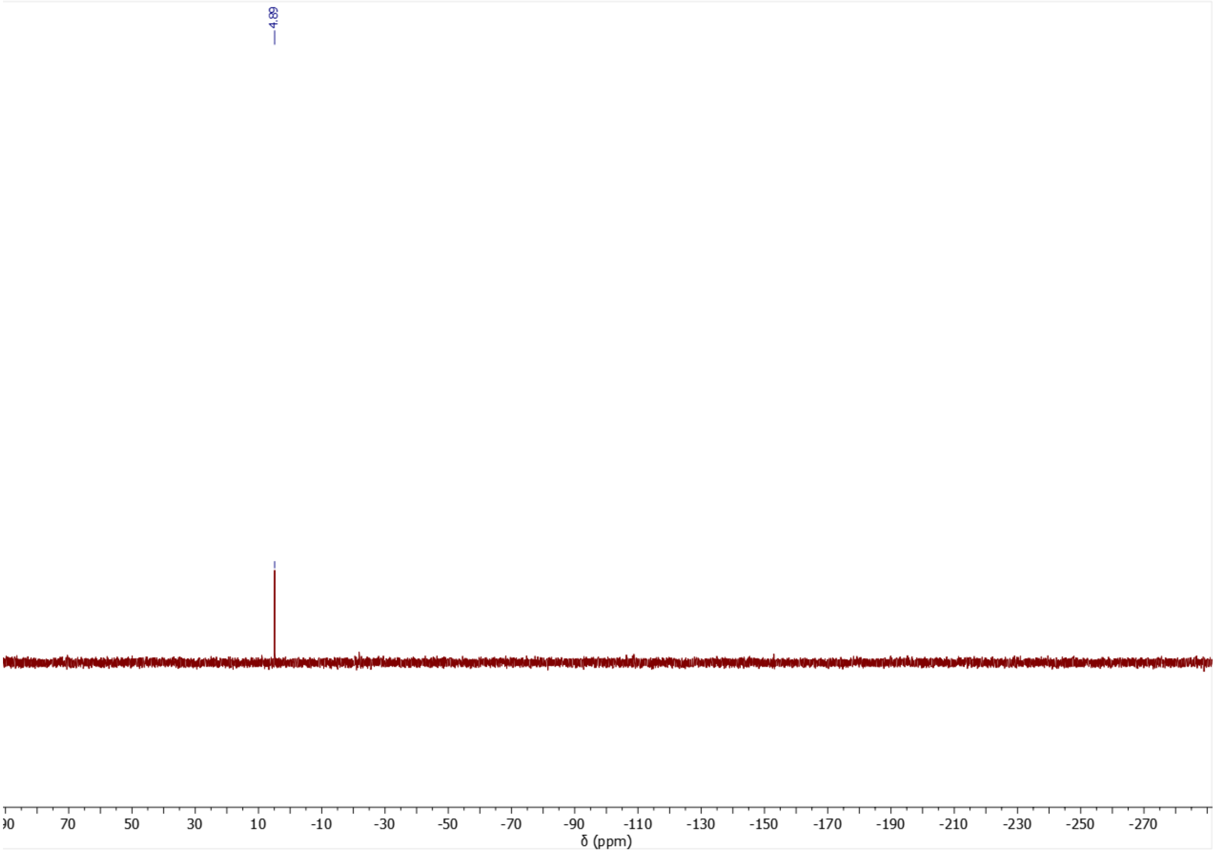

Figure S93. ^{29}Si NMR spectrum of compound **5** as a solution in C_6D_6 at ambient temperature.

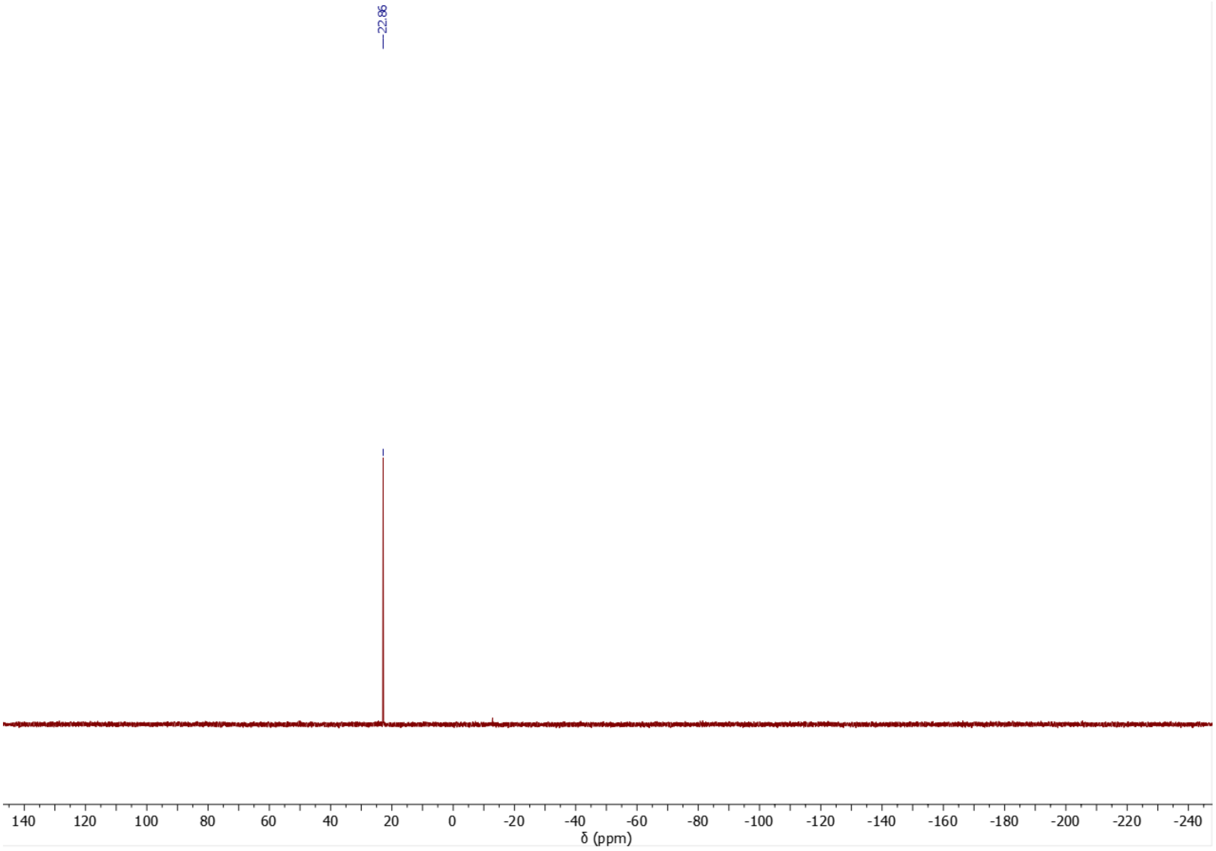

Figure S94. ^{31}P NMR spectrum of compound **5** as a solution in C_6D_6 at ambient temperature.

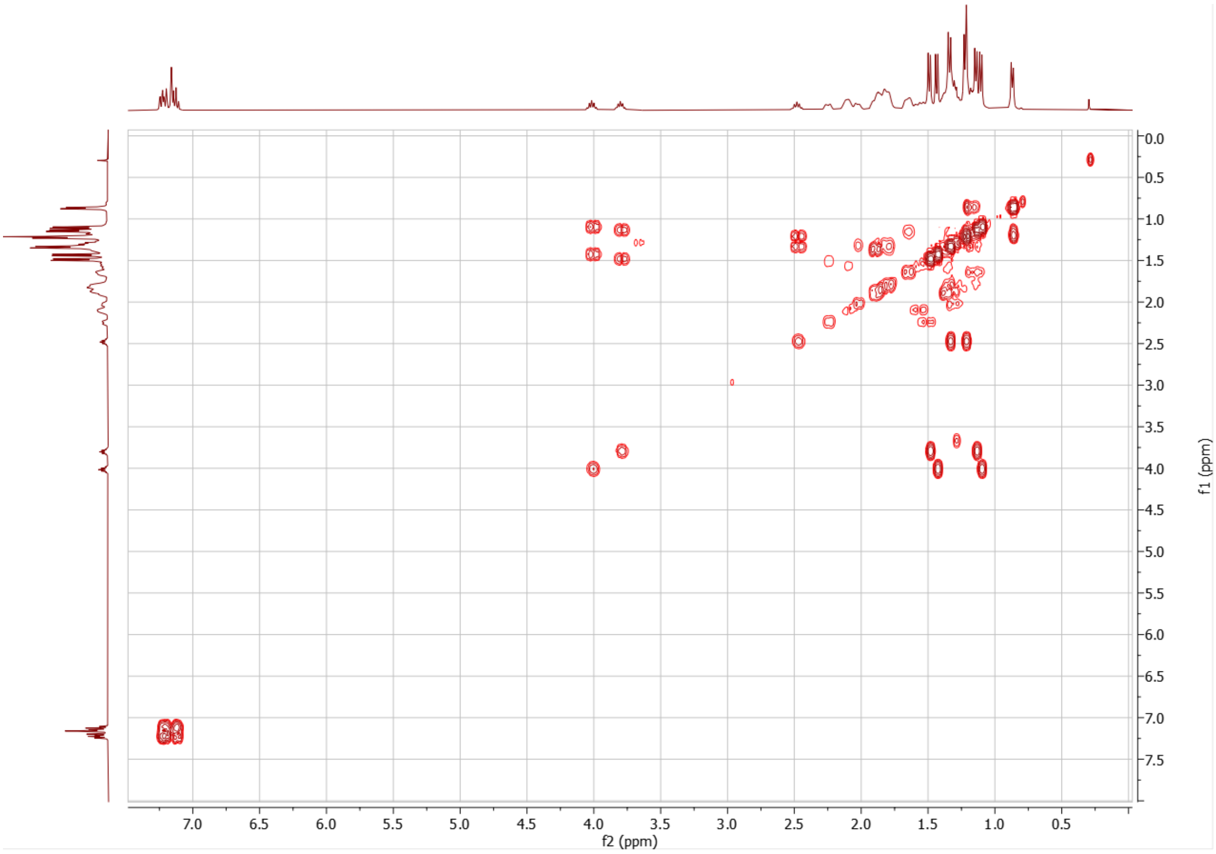

Figure S95. COSY NMR spectrum of compound **5** as a solution in C_6D_6 at ambient temperature.

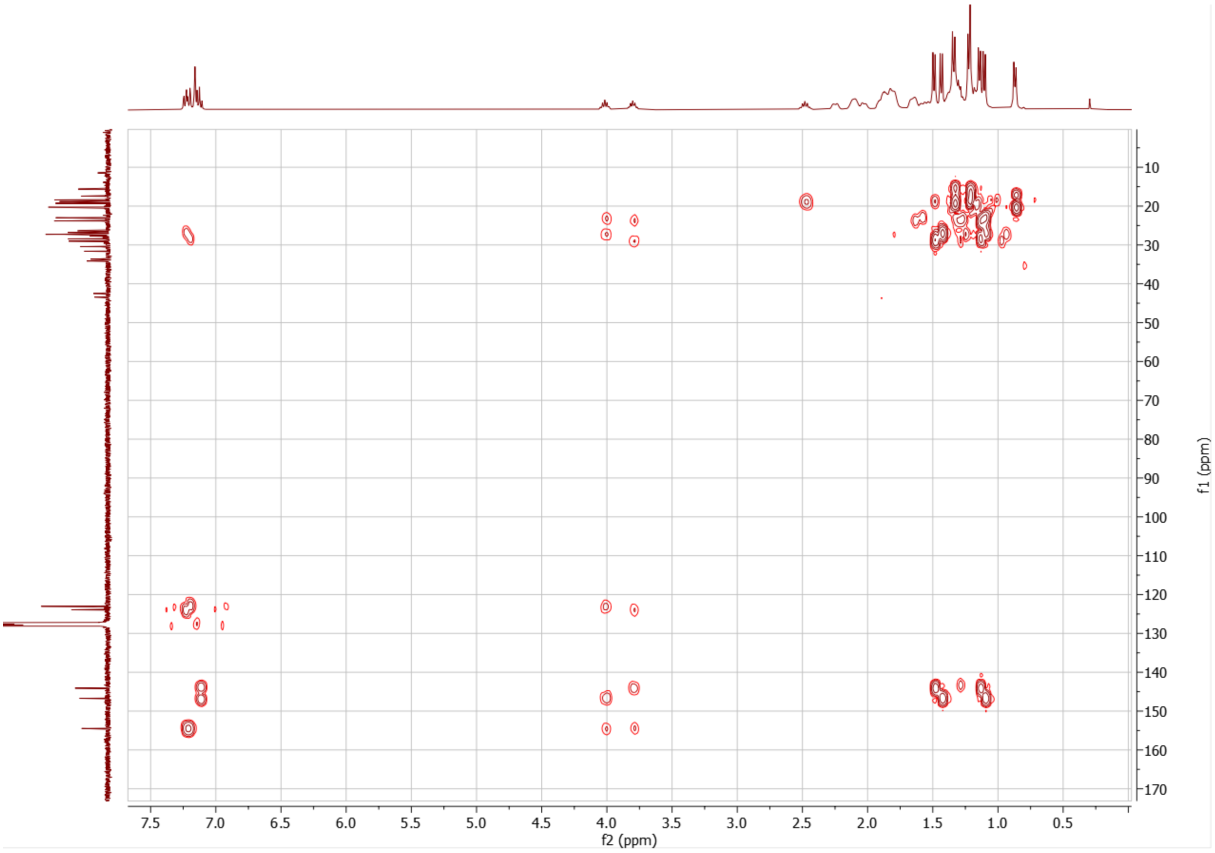

Figure S96. HMBC NMR spectrum of compound **5** as a solution in C_6D_6 at ambient temperature.

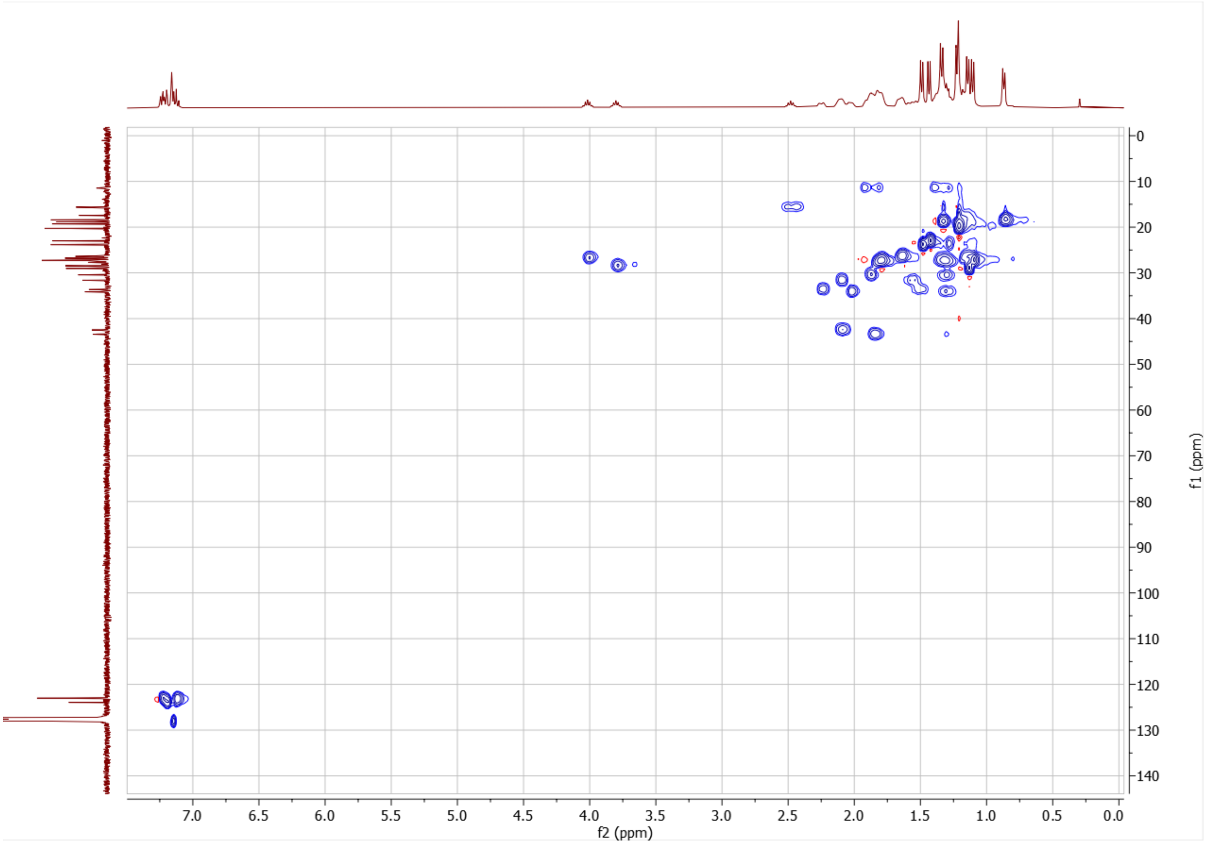

Figure S97. HSQC NMR spectrum of compound **5** as a solution in C_6D_6 at ambient temperature.

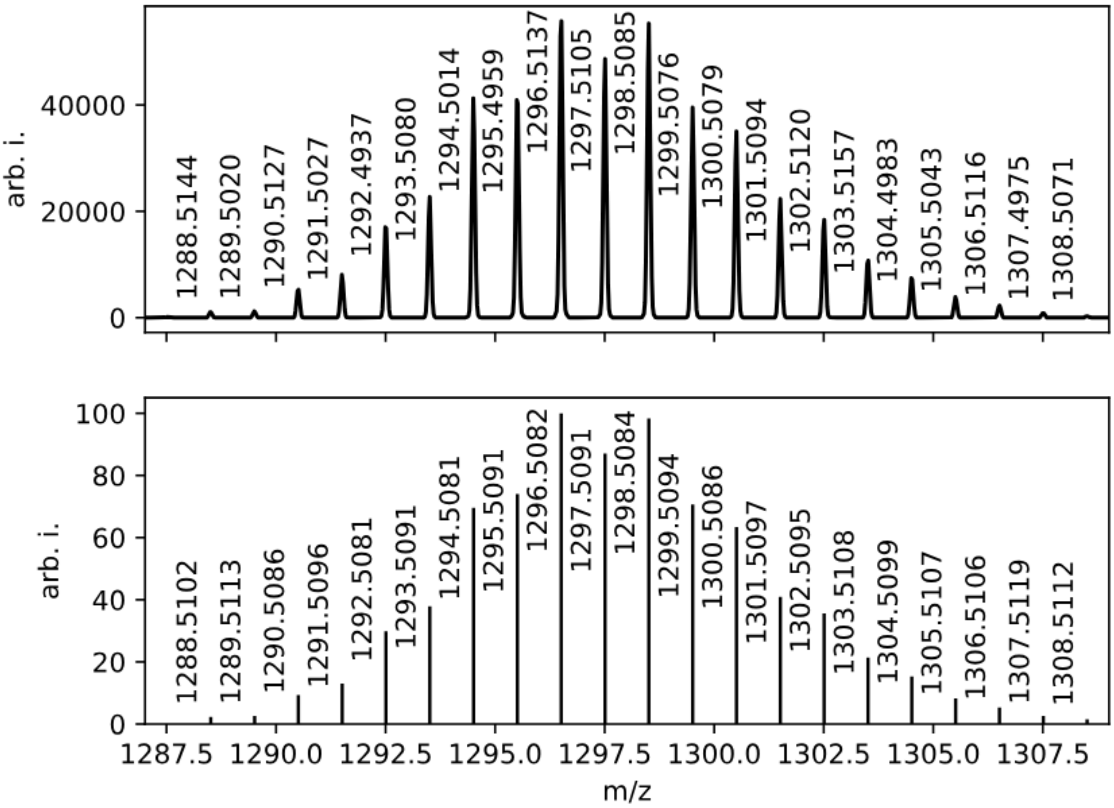

Figure S98. Cutout from LIFDI/MS of compound **5**; Top. found MS for $[M]^+$; Bottom. Calculated MS spectrum of $[M]^+$.

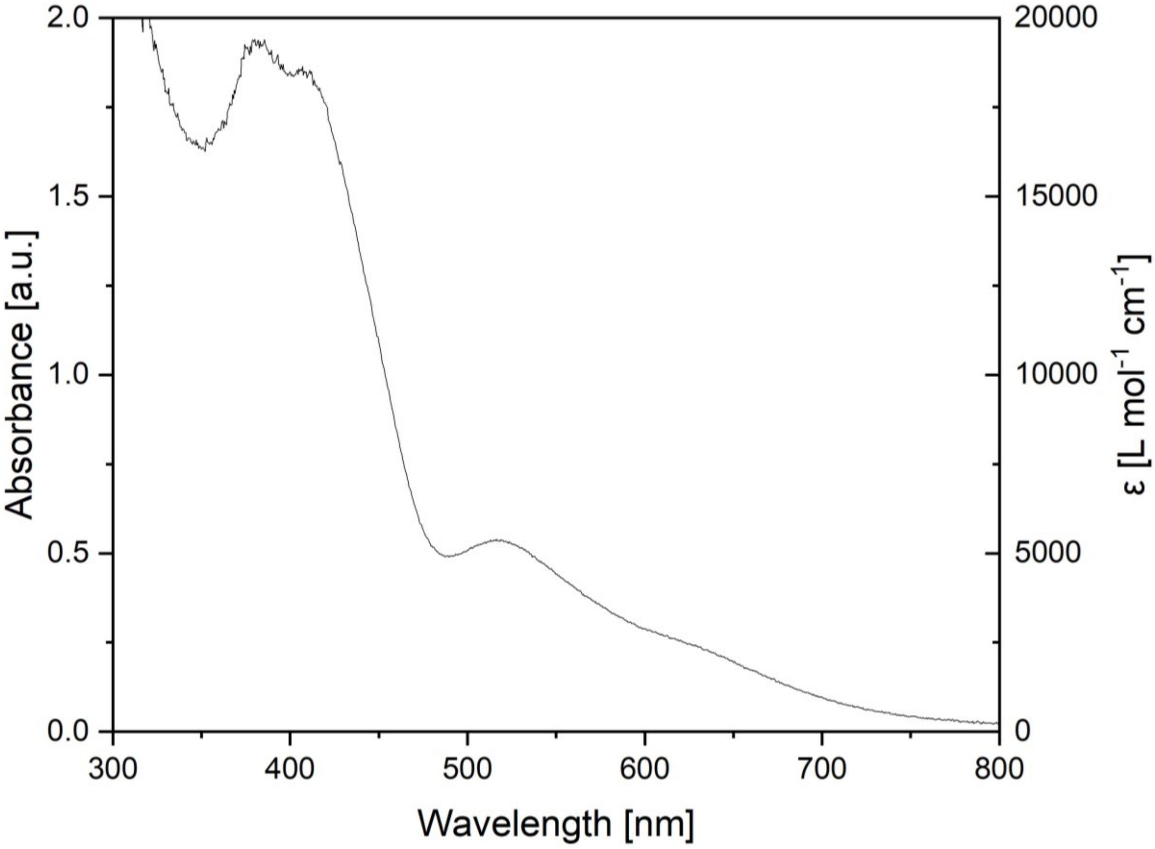

Figure S99. UV/vis spectrum of a 1.0x10^{-4} M solution of compound **5** in toluene at ambient temperature.

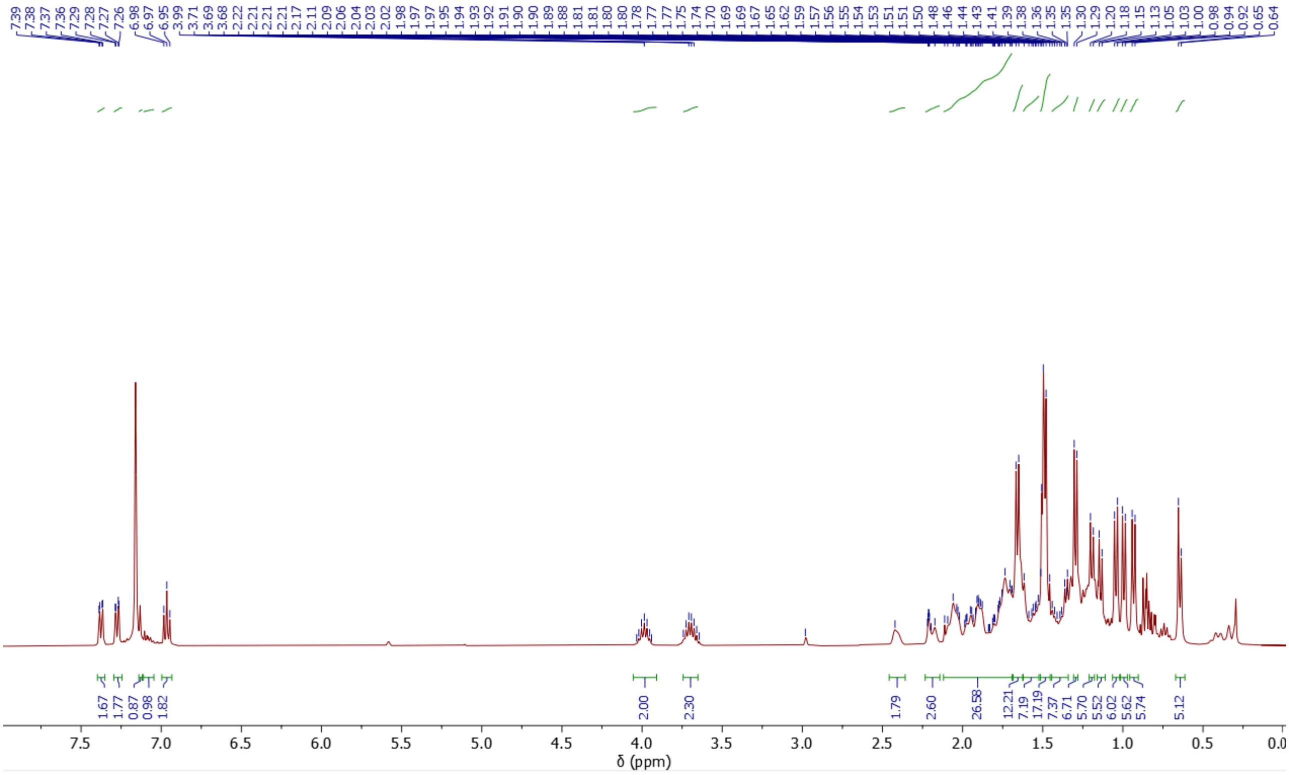

Figure S100. ^{1}H NMR spectrum of compound **6** as a solution in C_6D_6 at ambient temperature.

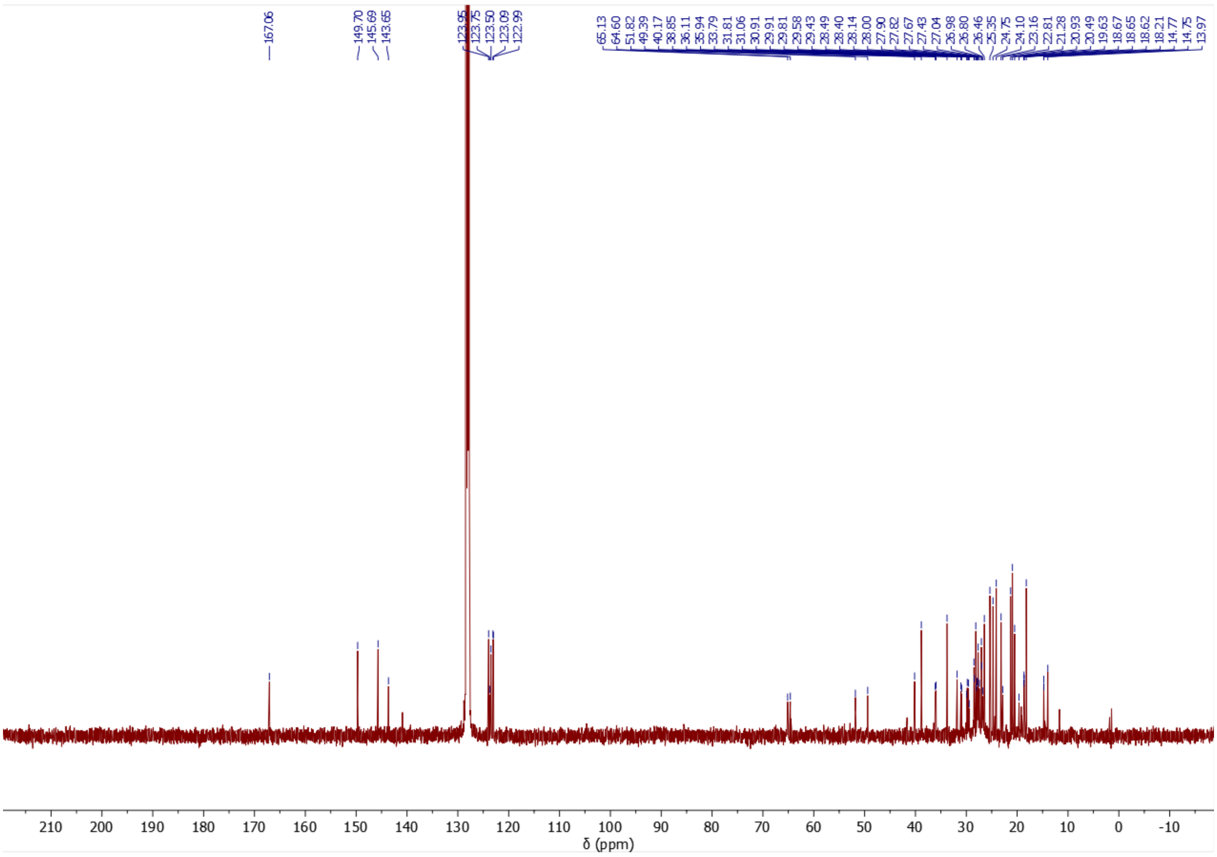

Figure S101. ^{13}C NMR spectrum of compound **6** as a solution in C_6D_6 at ambient temperature.

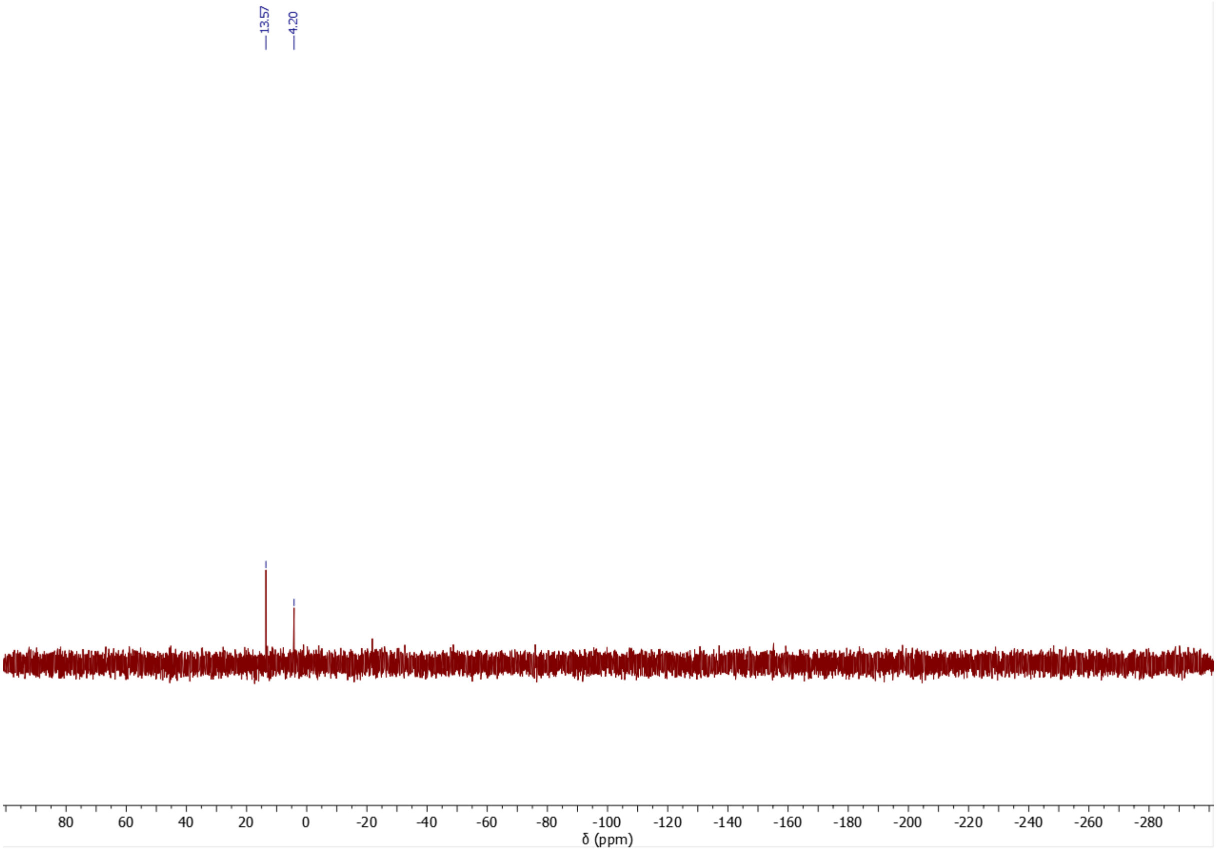

Figure S102. ^{29}Si NMR spectrum of compound **6** as a solution in C_6D_6 at ambient temperature.

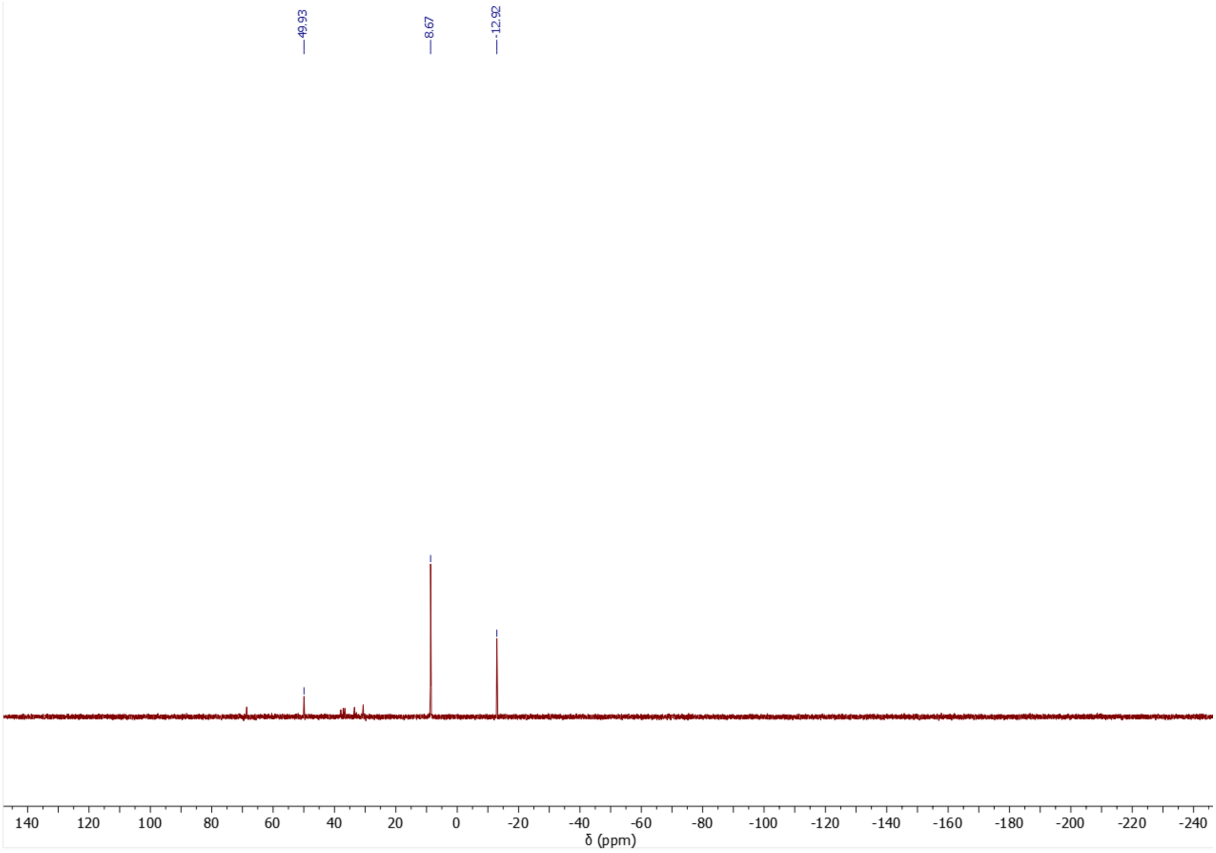

Figure S103. ^{31}P NMR spectrum of compound **6** as a solution in C_6D_6 at ambient temperature.

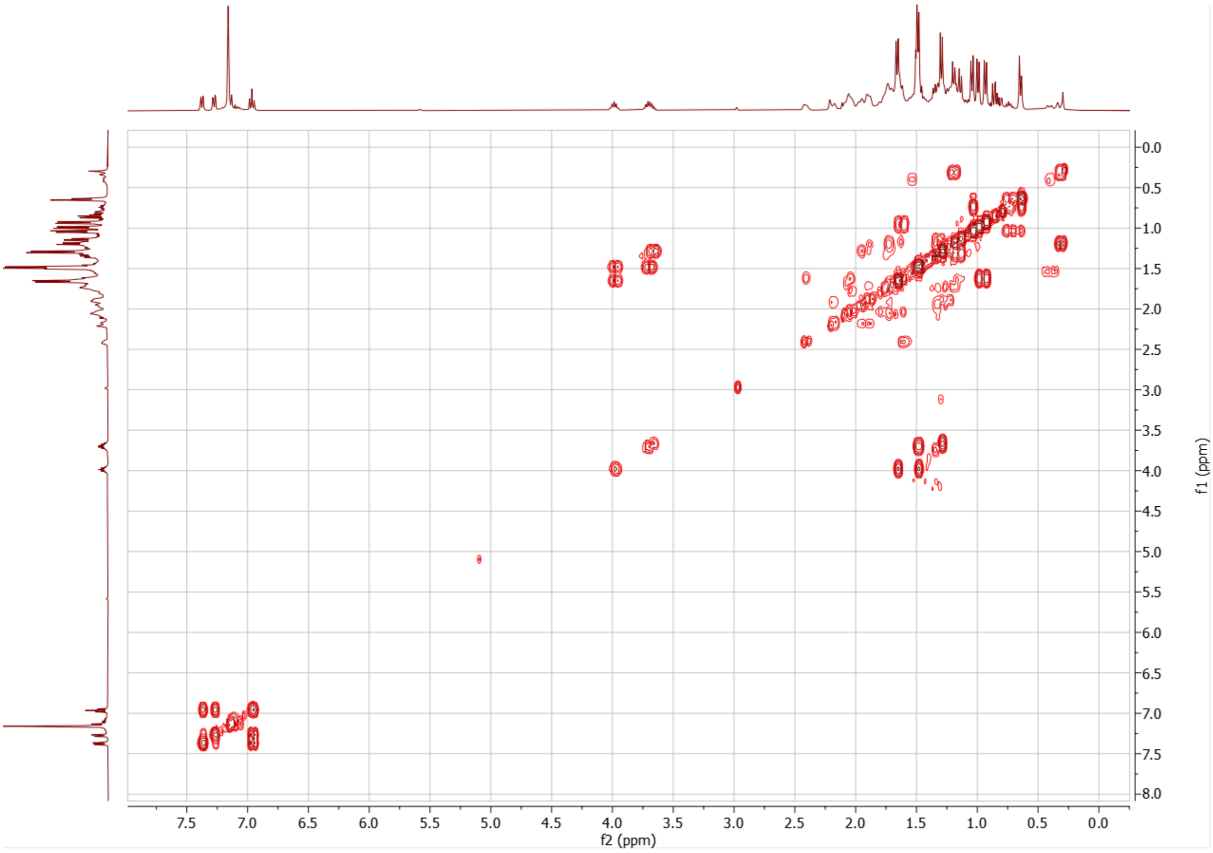

Figure S104. COSY NMR spectrum of compound **6** as a solution in C_6D_6 at ambient temperature.

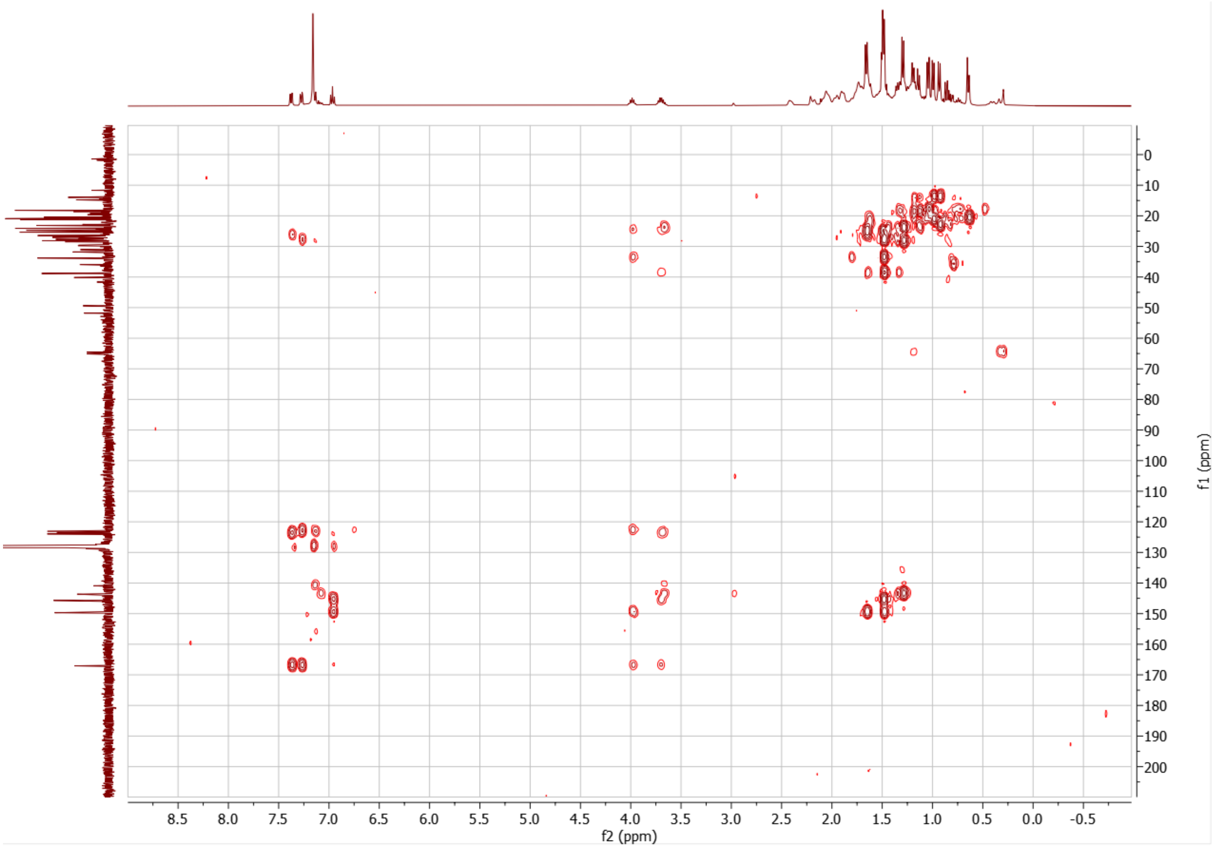

Figure S105. HMBC NMR spectrum of compound **6** as a solution in C_6D_6 at ambient temperature.

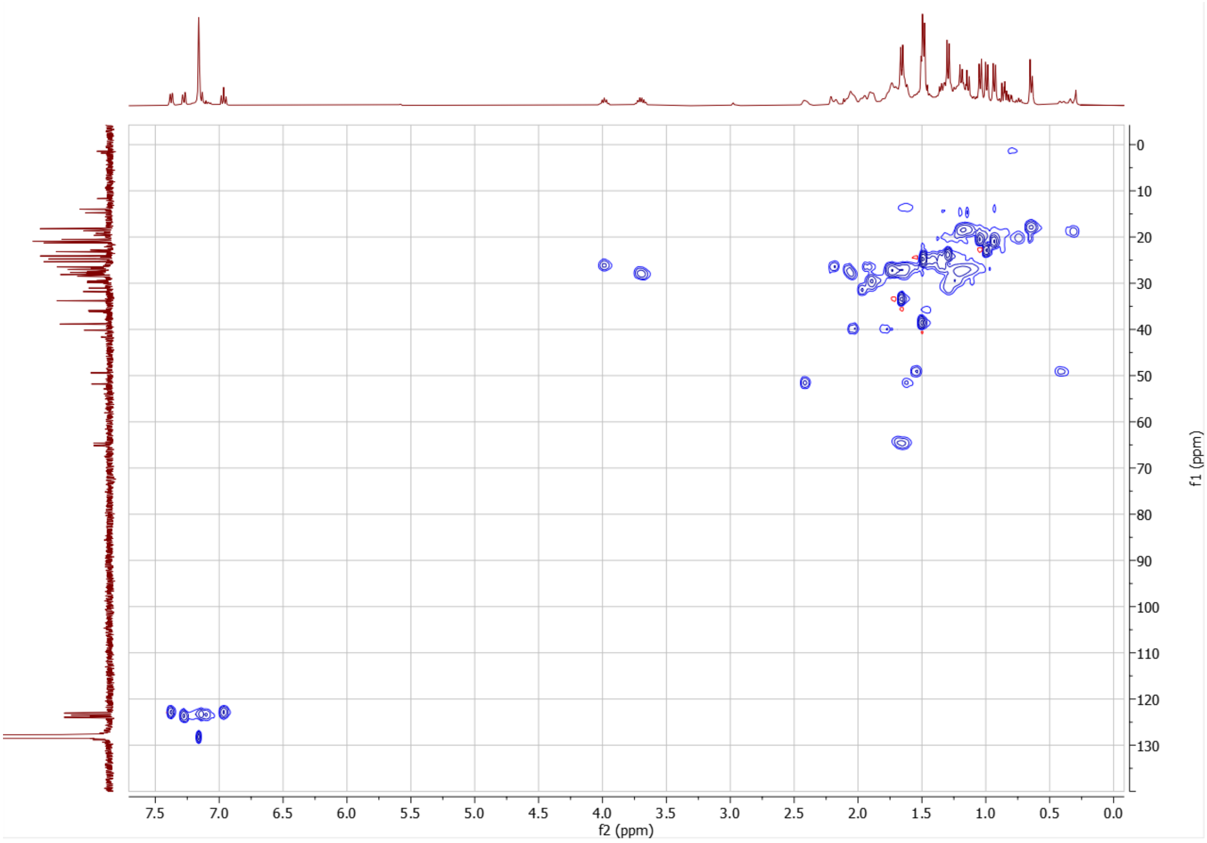

Figure S106. HMBC NMR spectrum of compound **6** as a solution in C_6D_6 at ambient temperature

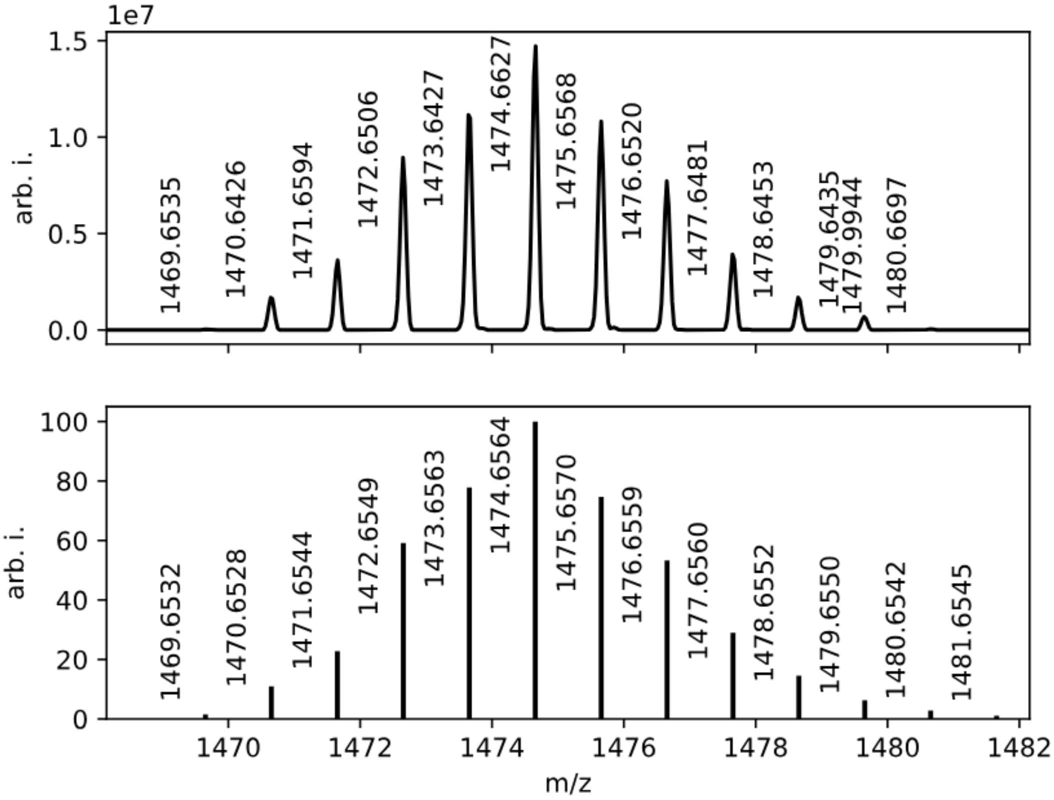

Figure S107. Cutout from LIFDI/MS of compound **6**; Top. found MS for $[M]^+$; Bottom. Calculated MS spectrum of $[M]^+$.

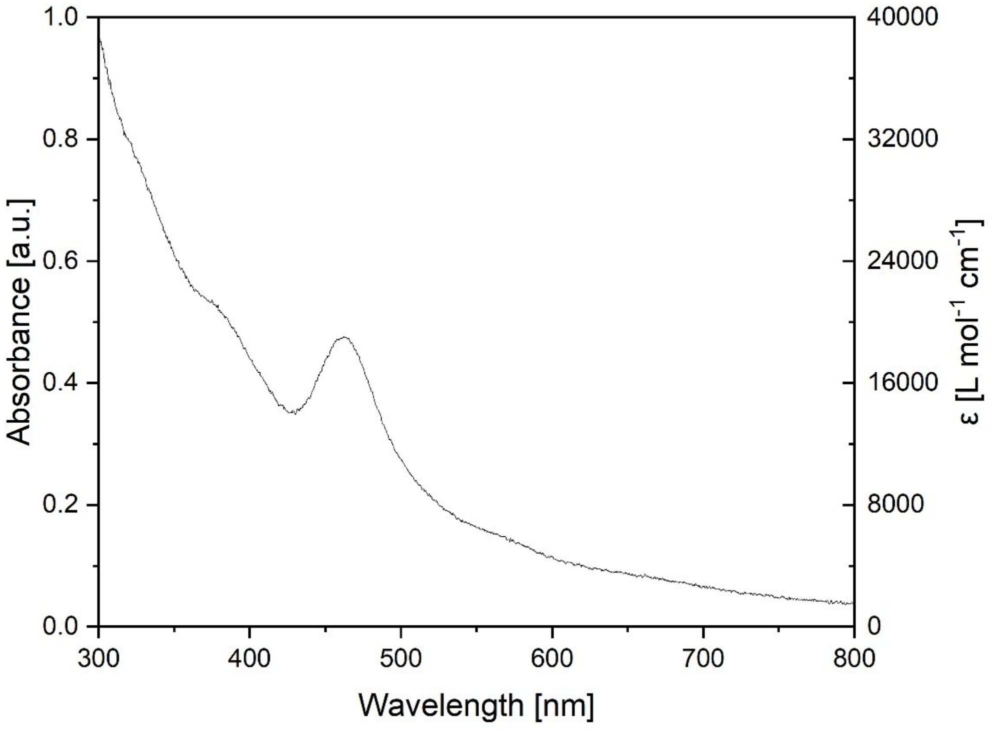

Figure S108. UV/vis spectrum of a $2.5x10^{-5}$ M solution of compound **6** in toluene at ambient temperature.

2. X-ray crystallographic details

Single crystals of **^{Me}L(Cl)Ge:**, **1-Me**, **2-Me, 2-Ph**, **2-Cy**, **3**, **4**, **5**, and **6** suitable for X-ray structural analysis were mounted in perfluoroalkyl ether oil on a nylon loop and positioned in a 150 K cold N_2 gas stream. Data collection was performed with a STOE StadiVari diffractometer (MoKα radiation) equipped with a DECTRIS PILATUS 300K detector. Structures were solved by Direct Methods (SHELXS-97),[10] or using SHELXT-16,[11] and refined by full-matrix least-squares calculations against F2 (SHELXL-2018).[12] The positions of the hydrogen atoms were calculated and refined using a riding model. All non-hydrogen atoms were treated with anisotropic displacement parameters. Crystal data, details of data collections, and refinements for all structures can be found in their CIF files, which are available free of charge *via* www.ccdc.cam.ac.uk/data_request/cif, and are summarized in Table S1 and S2.

Table S1. Summary of X-ray crystallographic data for compounds **^{Me}L(Cl)Ge:**, **1-Me**, **2-Me**, **2-Ph**, and **2-Cy**.

	**^{Me}L(Cl)Ge:**	**1-Me**	**2-Me**	**2-Ph**	**2-Cy**
empirical form.	$C_{21}H_{39}ClGeNPSi$	$C_{42}H_{78}Ge_2N_2P_2Si_2$	$C_{42}H_{71}N_2P_2Si_2Sn_2$	$C_{62}H_{86}N_2P_2Si_2Sn_2$, C_5H_{12}	$C_{62}H_{110}N_2P_2Si_2Sn_2$
formula wt	472.63	874.36	959.50	1286.97	1239.01
crystal syst.	triclinic	monoclinic	orthorhombic	monoclinic	triclinic
space group	*P-1*	*P2₁/n*	*Fdd2*	*P2₁/n*	*P-1*
a (Å)	10.446(2)	11.098(2)	37.871(8)	15.030(3)	10.569(2)
b (Å)	16.496(3)	14.759(3)	50.421(10)	19.540(4)	12.948(3)
c (Å)	30.367(6)	14.511(3)	10.413(2)	23.270(5)	15.327(3)
α (deg.)	77.44(3)	90	90	90	74.78(3)
β (δεγ)	86.39(3)	96.04(3)	90	100.60(3)	77.88(3)
γ (deg.)	88.18(3)	90	90	90	73.22(3)
vol (Å^3)	5096.6(19)	2363.6(8)	19884(7)	6717(2)	1917.1(8)
Z	8	2	16	4	1
ρ(calc) (g.cm^{-3})	1.232	1.229	1.282	1.273	1.073
μ (mm^{-1})	1.423	1.419	1.145	0.865	0.755
F(000)	2000	932	7920	2688	654
T (K)	150(2)	150(2)	150(2)	150(2)	150(2)
reflns collect.	65069	18562	36039	49671	26113
unique reflns	20024	4643	9674	13181	7495
R_{int}	0.0537	0.0428	0.1734	0.1286	0.0192
R1 [$I>2\sigma(I)$]	0.0667	0.0543	0.0777	0.0537	0.0245
*w*R2 (all data)	0.2016	0.1474	0.1832	0.1028	0.0625
CCDC No.	2528520	2528521	2528522	2528523	2528524

Table S2. Summary of X-ray crystallographic data for compounds **3**, **4**, **5**, and **6**.

	**3**	**4**	**5**	**6**
empirical form.	$C_{62}H_{86}Ge_2N_2NiP_2Si_2$	$C_{62}H_{86}N_2NiP_2Si_2Sn_2$	$C_{62}H_{110}N_2NiP_2Si_2Sn_2$	$C_{62}H_{110}N_2NiP_2Pb_2Si_2$, $2(C_6H_6)$
formula wt	1181.33	1273.53	1297.72	1630.94
crystal syst.	triclinic	triclinic	monoclinic	triclinic
space group	*P-1*	*P-1*	$P2_1/n$	*P-1*
a (Å)	11.434(2)	11.120(2)	20.757(4)	13.358(3)
b (Å)	12.403(3)	15.390(3)	14.935(3)	15.434(3)
c (Å)	45.227(9)	19.260(4)	22.893(5)	19.635(4)
α (deg.)	87.09(3)	86.70(3)	90	77.62(3)
β (δεγ)	87.20(3)	88.80(3)	110.94(3)	78.64(3)
γ (deg.)	73.53(3)	69.90(3)	90	73.03(3)
vol ($Å^3$)	6139(2)	3090.2(12)	6628(3)	3742.6(15)
Z	4	2	4	2
ρ(calc) ($g.cm^{-3}$)	1.278	1.369	1.300	1.447
μ (mm^{-1})	1.406	1.232	1.150	4.851
F(000)	2488	1316	2728	1660
T (K)	150(2)	150(2)	150(2)	150(2)
reflns collect.	23950	39157	86818	52013
unique reflns	14483	11486	13031	14679
R_{int}	0.0793	0.0725	0.0274	0.0271
R1 [$I>2\sigma(I)$]	0.0489	0.0573	0.0265	0.0307
*w*R2 (all data)	0.1015	0.1468	0.0662	0.0742
CCDC No.	2528525	2528526	2528527	2528528

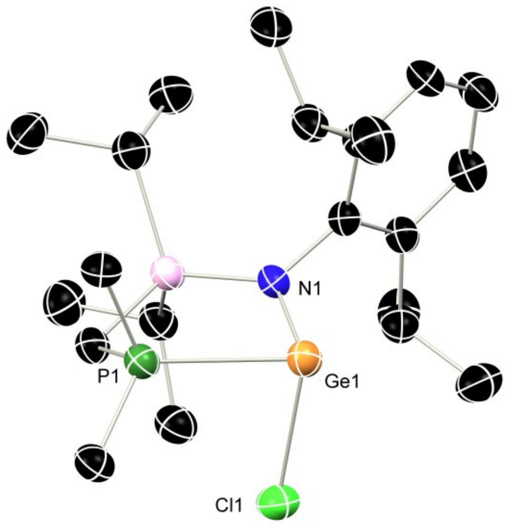

Figure S109. Molecular structure of $^{Me}L(Cl)Ge$:, with thermal ellipsoids at 30% probability, and hydrogen atoms removed for clarity.

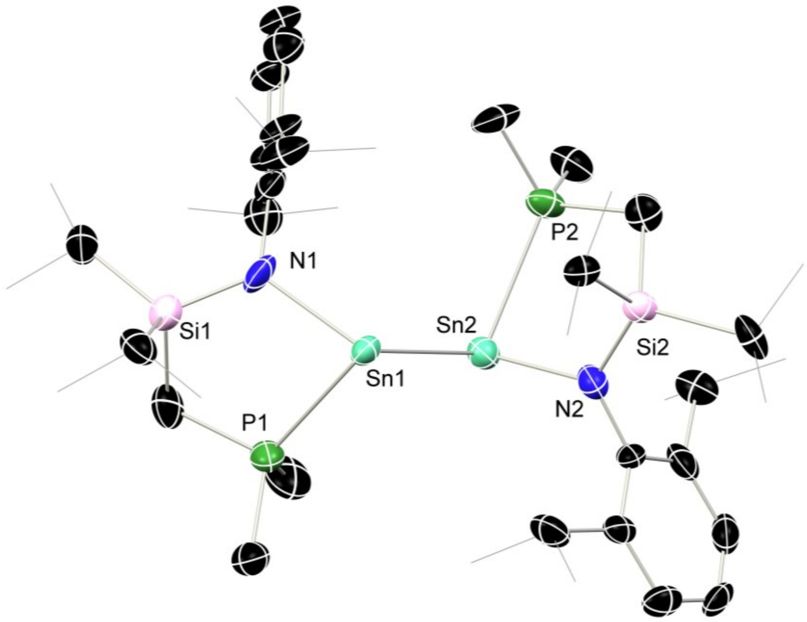

Figure S110. Molecular structure of ^{Me}L(Cl)Ge:, with thermal ellipsoids at 30% probability, and hydrogen atoms removed for clarity.

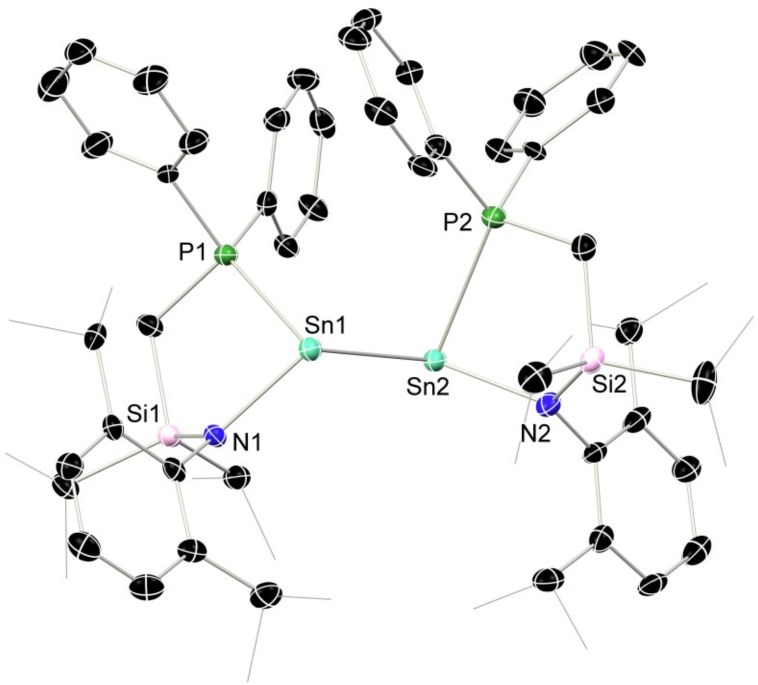

Figure S111. Molecular structure of ^{Me}L(Cl)Ge:, with thermal ellipsoids at 30% probability, and hydrogen atoms removed for clarity.

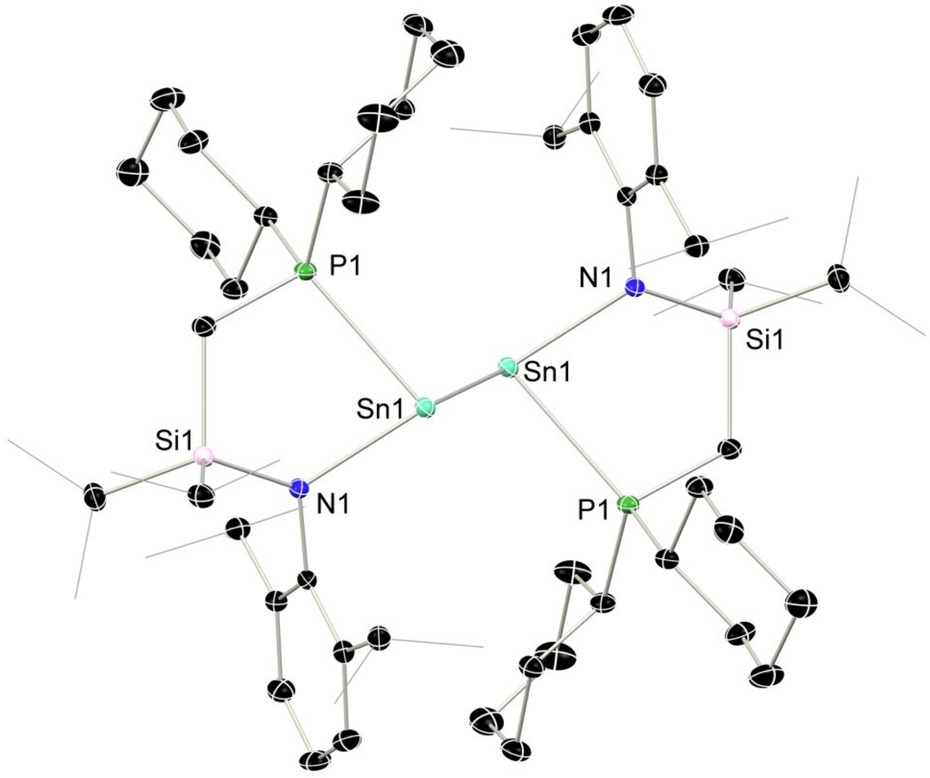

Figure S112. Molecular structure of ^{Me}L(Cl)Ge:, with thermal ellipsoids at 30% probability, and hydrogen atoms removed for clarity.

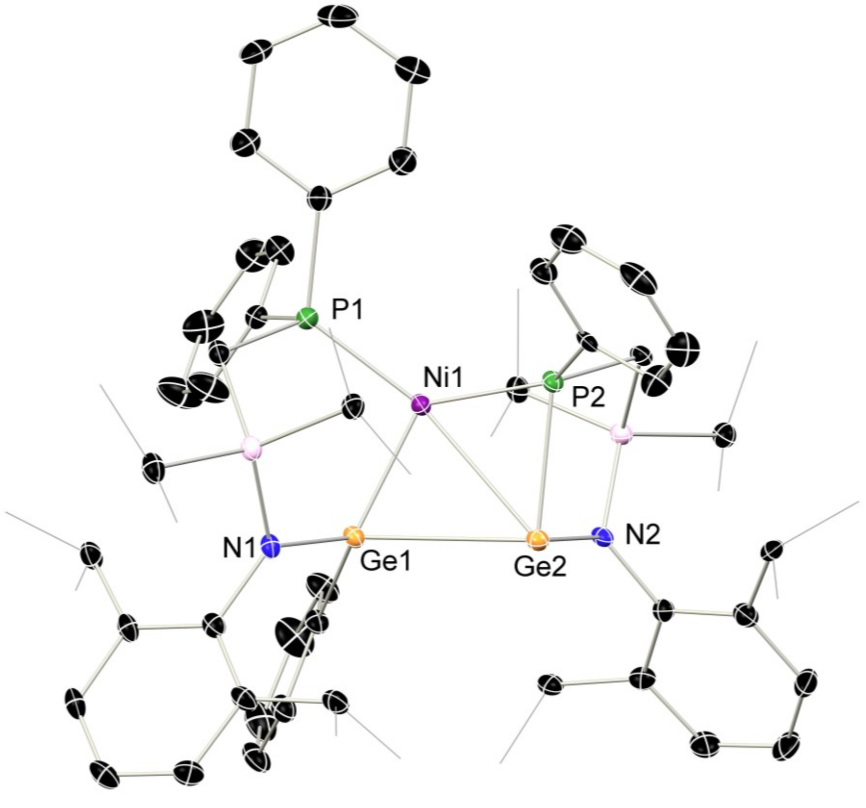

Figure S113. Molecular structure of ^{Me}L(Cl)Ge:, with thermal ellipsoids at 30% probability, and hydrogen atoms removed for clarity.

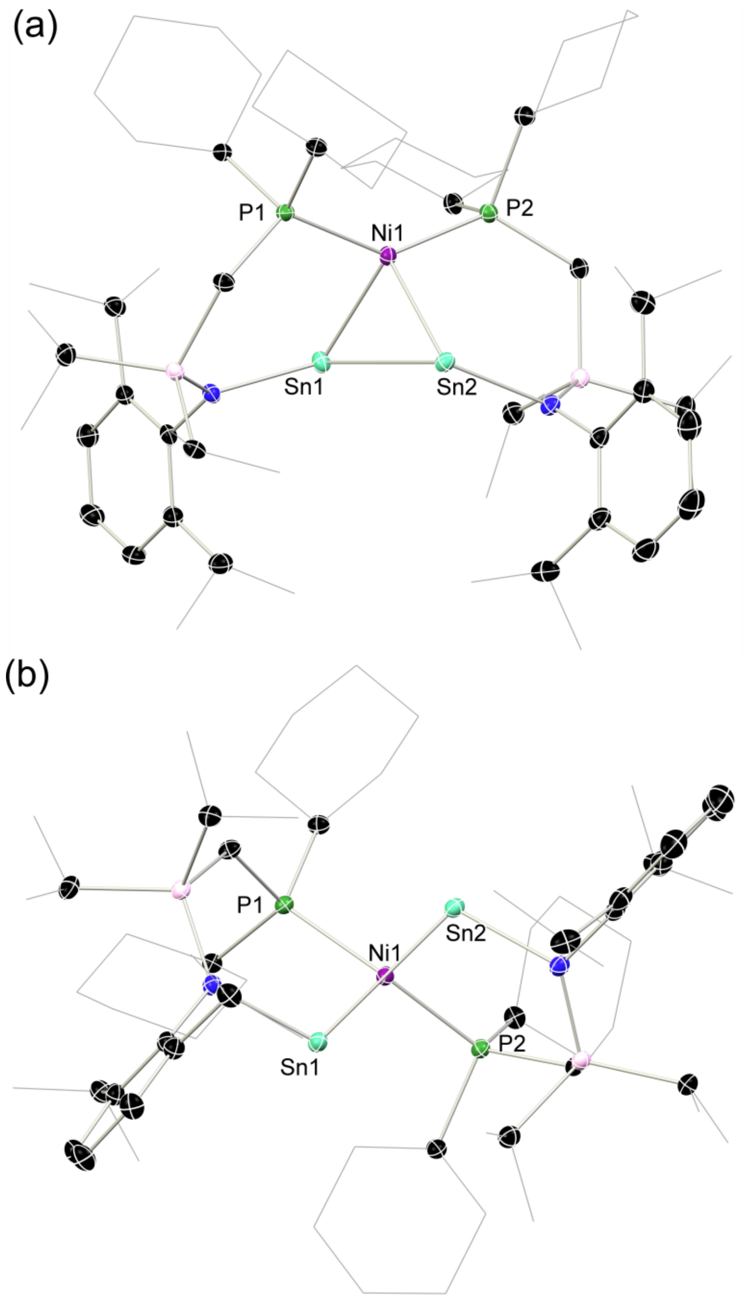

Figure S114. Molecular structure of $^{Me}L(Cl)Ge$:, with thermal ellipsoids at 30% probability, and hydrogen atoms removed for clarity. (a) View perpendicular to the [$NiSn_2$] plane, and (b) view parallel to the [$NiSn_2$] plane.

Response to CIFCheck Alerts:

Compound **2-Me**

One B-alert:

ALERT: PLAT342

PROBLEM: Low Bond Precision on C-C Bonds ....0.0325 Ang.

RESPONSE: Due to the incredibly high reactivity of crystalline samples of this compound, a degree of degradation during crystal picking, transfer, and data collection negatively affected data quality (*i.e.* Rint of ~17%). Still, the data is of good completeness (99.9%), and has an acceptable R factor of 7.7 %.

Compound **3**

Two B-alerts:

ALERT: PLAT230

PROBLEM: Hirshfeld Test Diff for C65--C66. 7.2 s.u.

RESPONSE: This is due to minor positional disorder in one Dipp group, which cannot be modelled due to weak residual density. This does not affect the overall quality of the data (99.2 % completeness, R factor = 4.89%)

ALERT: PLAT910

PROBLEM: Missing FCF Reflection(s) Below Theta(Min)[Deg]= 2.04 Note

RESPONSE: This is a minor and common issue, which does not affect the overall quality of the data (99.2 % completeness, R factor = 4.89%)

Compound **4**

Three B-alerts:

ALERT: PLAT220

PROBLEM: NonSolvent Resd 1 C Ueq(max)/Ueq(min) Range 6.5 Ratio

RESPONSE: This is due to positional disorder in the central [Sn2Ni] motif - due to the heavy Sn atoms minor disorder flags exaggerated problems. This disorder has been modelled, but leads to large displacement parameters.

ALERT: PLAT973

PROBLEM: Check Calcd Positive Resid. Density on Sn1 1.79 eA-3

RESPONSE: This is due to positional disorder in the central [Sn2Ni] motif - due to the heavy Sn atoms minor disorder flags exaggerated problems. This disorder has been modelled, but minor residual remains.

ALERT: PLAT973

PROBLEM: Check Calcd Positive Resid. Density on Sn2 1.52 eA-3

RESPONSE: This is due to positional disorder in the central [Sn2Ni] motif - due to the heavy Sn atoms minor disorder flags exaggerated problems. This disorder has been modelled, but minor residual remains.

4. Computational methods and details

All the geometry optimizations and frequency calculations reported in this paper were obtained with the ORCA 6.0.1 program.[13] Electron correlation was partially taken into account using the BP86[14],[15] functional in conjunction with the D3(BJ) dispersion correction suggested by Grimme *et al.*,[16],[17] the resolution-of-identity approach,[18] and the double-ζ quality plus polarization functions def2-SVP[19] basis set for all atoms. All species were characterized by frequency calculations: reactants and adducts exhibited positive definite Hessian matrices, while transition states showed a single negative eigenvalue in their diagonalized force constant matrices.

Wiberg Bond Indices (WBIs) and Second Order Pertubation Theory (SOPT) calculations were computed using the Natural Bond Orbital (NBO, v. 6.0) method at the same level.[20] Quantum Theory of Atoms and Molecules (QTAIM)[21] calculations were carried out at the same level using the optimized BP86-D3BJ/def2-SVP geometries at the BP86-D3BJ/6-31G*&WTBS (Sn, Pb) level.

The bonding situation in **5** and **6** was also analyzed with the Energy Decomposition Analysis (EDA) method to gain more insight into the E-E bond.[22],[23] Within this approach, the interaction between the selected fragments ([NiE···P] and [E····P] in this case), ΔE_{int} can be decomposed into the following physically meaningful terms:

$$\Delta E_{int} = \Delta E_{elstat} + \Delta E_{Pauli} + \Delta E_{orb} + \Delta E_{disp}$$

The term ΔE_{elstat} corresponds to the classical electrostatic interaction between the unperturbed charge distributions of the deformed reactants and is usually attractive. The Pauli repulsion ΔE_{Pauli} comprises the destabilizing interactions between occupied orbitals and is responsible for any steric repulsion. The orbital interaction ΔE_{orb} accounts for electron-pair bonding, charge transfer (interaction between occupied orbitals on one moiety with unoccupied orbitals on the other, including HOMO–LUMO interactions), and polarization (empty-occupied orbital mixing on one fragment due to the presence of another fragment). Finally, the ΔE_{disp} term takes into account the interactions which are due to dispersion forces. Moreover, the NOCV (Natural Orbital for Chemical Valence)[24] extension of the EDA method has been also used to further partition the ΔE_{orb} term. The EDA-NOCV approach provides pairwise energy contributions for each pair of interacting orbitals to the total bond energy.

The program package AMS 2020.101 was used for the EDA-NOCV calculations at the same BP86-D3 level,[25] in conjunction with a triple-ζ-quality basis set using uncontracted Slater-type orbitals (STOs) augmented by two sets of polarization functions with a frozen-core approximation for the core electrons.[26] Auxiliary sets of *s*, *p*, *d*, *f*, and g STOs were used to fit the molecular densities and to represent the Coulomb and exchange potentials accurately in each SCF cycle.[27] Scalar relativistic effects were incorporated by applying the zeroth-order regular approximation (ZORA).[28],[29],[30] This level of theory is denoted ZORA-BP86-D3BJ/TZ2P//RI-BP86-D3BJ/def2-SVP.

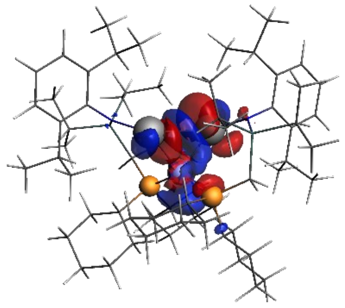	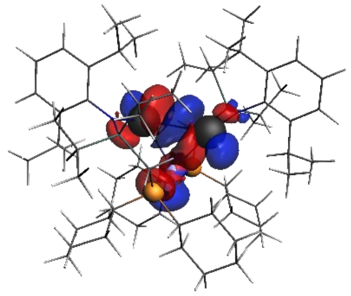
DE(r1) = -73.1 kcal/mol	DE(r1) = -80.6 kcal/mol

Figure S115. Contour plots of the main NOCV deformation densities r (isosurface value of 0.001 a.u.) and associated energies DE(r) in 5 (left) and 6 (right). The electronic charge flows from red to blue. All data have been computed at the ZORA-BP86-D3BJ/TZ2P//RI-BP86-D3BJ/def2-SVP level.

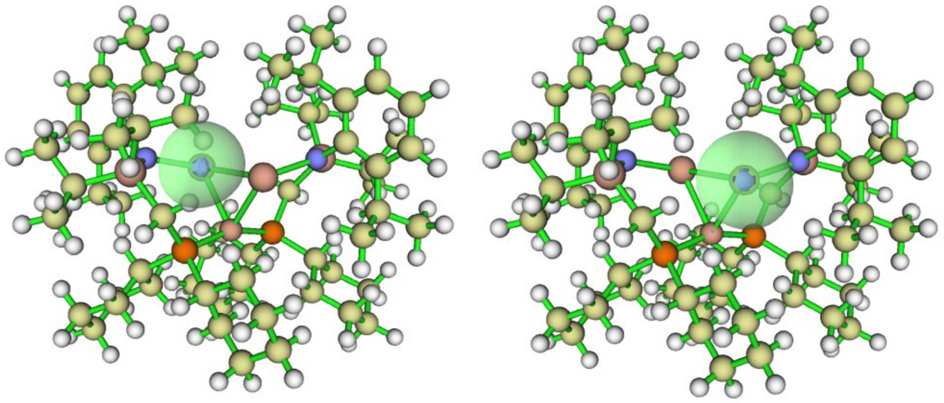

(**6**) LP(1) Pb1 (1.94 e) (**6**) LP(1) Pb2 (1.94 e)

Figure S116. Lone-pairs at the group 14 elements of compound **6** computed using the NBO method (isosurface value of 0.05 a.u.), The numbers in parentheses indicate the occupation of the orbital. All data have been computed at the BP86-D3BJ/def2-SVP//RI-BP86-D3BJ/def2-SVP level.

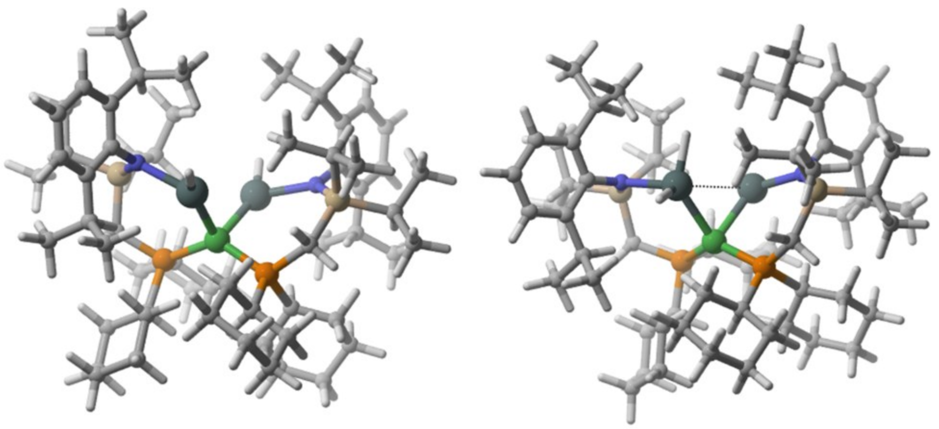

A **INT1**

Figure S117. Fully optimized geometries of species **A** and **INT1** involved in the computed reaction profile shown in Figure 4 in the main text. All data have been computed at the RI-BP86-D3BJ/def2-SVP level.

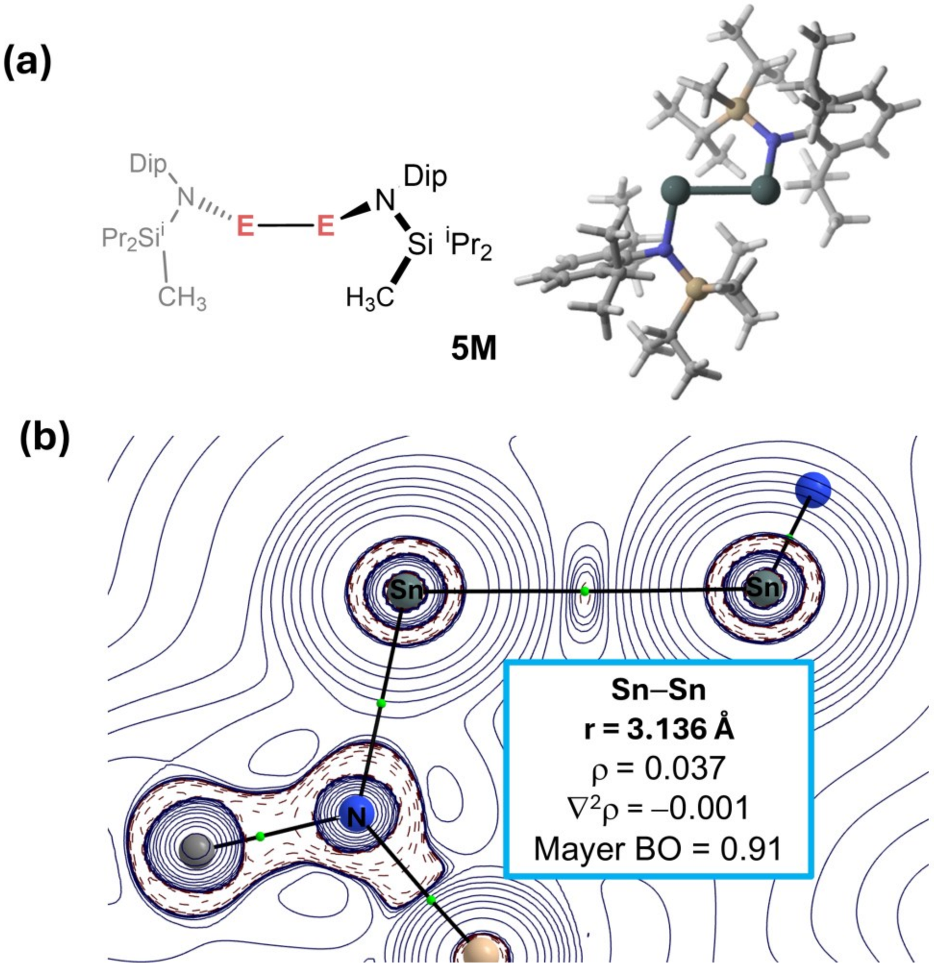

Figure S118. (a) Contour line diagrams $\nabla^2\rho(r)$ computed for **5M** in the N-Sn-Sn plane. Solid lines connecting the atomic nuclei are bond paths, while the small green spheres indicate the corresponding bond critical points, respectively.

Cartesian coordinates (in Å) and free energies (in a.u.) of all the stationary points discussed in the text.

A: G= -5735.65126

Sn	10.307125000	8.655746000	10.822168000
Sn	12.912412000	6.308596000	11.365712000
Ni	11.305252000	6.830906000	9.717671000
P	10.029280000	5.100226000	9.958878000
P	12.199216000	7.708448000	7.951575000
Si	12.708396000	10.527826000	9.450071000
Si	10.627758000	4.522630000	13.215465000
N	12.306094000	4.992060000	12.907222000
N	11.233180000	10.520130000	10.429516000
C	8.354465000	5.189120000	9.080038000
H	8.636202000	5.302874000	8.009377000
C	13.361551000	4.487326000	13.718997000
C	10.851340000	3.551038000	9.276670000
H	11.915308000	3.725755000	9.556323000
C	10.781340000	3.563685000	7.739327000
H	9.728018000	3.412732000	7.415080000
H	11.085319000	4.562802000	7.363989000
C	14.039418000	3.292585000	13.328776000
C	9.401600000	11.967112000	9.606383000
C	14.151534000	9.648733000	10.350325000
H	13.864178000	8.577372000	10.231521000
C	13.933147000	7.021056000	7.673744000
H	14.417438000	7.292394000	8.640684000
C	13.780068000	5.181243000	14.893741000
C	9.791199000	5.686349000	14.498717000
H	10.429781000	5.626703000	15.405960000
C	9.495011000	4.655412000	11.674099000
H	8.875962000	3.735285000	11.645092000
H	8.791999000	5.476383000	11.932145000
C	10.402752000	11.661629000	10.577087000
C	12.438009000	9.543987000	7.828693000
H	11.512137000	9.957038000	7.378444000
H	13.249314000	9.768416000	7.107104000
C	13.679580000	2.580416000	12.029462000
H	12.639981000	2.876066000	11.779716000
C	9.754919000	7.149731000	14.037311000
H	9.059667000	7.285801000	13.180977000
H	10.750022000	7.520912000	13.714093000
H	9.395403000	7.826769000	14.841962000
C	13.905881000	5.484895000	7.607160000
H	13.259614000	5.096335000	8.421726000
H	13.440568000	5.165820000	6.647686000
C	7.431949000	3.964252000	9.200538000
H	7.949264000	3.050294000	8.846587000
H	7.183813000	3.791021000	10.270970000
C	11.220173000	7.338630000	6.383730000
H	11.339225000	6.238372000	6.253285000
C	7.607760000	6.471195000	9.491292000
H	7.374430000	6.431206000	10.579445000
H	8.275312000	7.347741000	9.351264000

C	9.144905000	11.051808000	8.415701000
H	9.838733000	10.192257000	8.522730000
C	10.627587000	2.727951000	13.908876000
H	11.443080000	2.220619000	13.350069000
C	10.460251000	2.180377000	9.849649000
H	10.595954000	2.173470000	10.949782000
H	9.384862000	1.973706000	9.664298000
C	13.141689000	6.500625000	15.300529000
H	12.282513000	6.649914000	14.621963000
C	11.309483000	1.065301000	9.215322000
H	12.368324000	1.208392000	9.523227000
H	10.999967000	0.076286000	9.614169000
C	15.084006000	2.803550000	14.133408000
H	15.606559000	1.881059000	13.838864000
C	10.538789000	12.489588000	11.733096000
C	15.478828000	3.469016000	15.300906000
H	16.296805000	3.067872000	15.918516000
C	14.830610000	4.654311000	15.665819000
H	15.150158000	5.191761000	16.572493000
C	13.072540000	12.343682000	8.953557000
H	12.056738000	12.780400000	8.816364000
C	11.647262000	8.041266000	5.084536000
H	11.578661000	9.142089000	5.227557000
H	12.704941000	7.824572000	4.839799000
C	14.818418000	7.580833000	6.549041000
H	14.403873000	7.283416000	5.562108000
H	14.836292000	8.690498000	6.560979000
C	13.828064000	12.486541000	7.620539000
H	13.997253000	13.558194000	7.376716000
H	13.271054000	12.042057000	6.771092000
H	14.826422000	12.001770000	7.652969000
C	9.736070000	7.617627000	6.674335000
H	9.456179000	7.104116000	7.612684000
H	9.609719000	8.703582000	6.878586000
C	14.565661000	3.071330000	10.870254000
H	14.470789000	4.168941000	10.720521000
H	14.285068000	2.584643000	9.912943000
H	15.638256000	2.866746000	11.066947000
C	15.485622000	9.845262000	9.610324000
H	15.865303000	10.882042000	9.722266000
H	15.401334000	9.643688000	8.523153000
H	16.268237000	9.165361000	10.011515000
C	11.649119000	2.456276000	7.129820000
H	11.574979000	2.474393000	6.021770000
H	12.716026000	2.654722000	7.378678000
C	15.322471000	4.904209000	7.714657000
H	15.722757000	5.136609000	8.727092000
H	15.290342000	3.796425000	7.639970000
C	8.377445000	5.214588000	14.885089000
H	7.907885000	5.913569000	15.611325000
H	8.379445000	4.209465000	15.350745000
H	7.702405000	5.169014000	14.003243000
C	6.129565000	4.158475000	8.403883000
H	6.377653000	4.218025000	7.319747000
H	5.474637000	3.269753000	8.526798000
C	14.281614000	9.910928000	11.854636000
H	15.113455000	9.316333000	12.289756000
H	13.356442000	9.632001000	12.398473000
H	14.488165000	10.978310000	12.074754000
C	11.522526000	12.095866000	12.825881000

H	12.363946000	11.589971000	12.313932000
C	5.391124000	5.437096000	8.821918000
H	5.054081000	5.335633000	9.878625000
H	4.472915000	5.573410000	8.212323000
C	10.900512000	11.053919000	13.775042000
H	10.026154000	11.478498000	14.310549000
H	11.638837000	10.709917000	14.529409000
H	10.540520000	10.163227000	13.222232000
C	11.241719000	1.082159000	7.681658000
H	11.886387000	0.282997000	7.258571000
H	10.202020000	0.852253000	7.354751000
C	8.610413000	13.118579000	9.774685000
H	7.844280000	13.359470000	9.020315000
C	6.306934000	6.662758000	8.701339000
H	5.787253000	7.581392000	9.045599000
H	6.559874000	6.828821000	7.629997000
C	13.727870000	1.049969000	12.151814000
H	14.761054000	0.679803000	12.313176000
H	13.352803000	0.567500000	11.227680000
H	13.108523000	0.694294000	13.000116000
C	9.723449000	13.626583000	11.861277000
H	9.832151000	14.271273000	12.746004000
C	11.017728000	2.694463000	15.399667000
H	10.244226000	3.172037000	16.036993000
H	11.978857000	3.210812000	15.591836000
H	11.128762000	1.646773000	15.754610000
C	9.334333000	1.934289000	13.651149000
H	9.413551000	0.907404000	14.070214000
H	9.113712000	1.825301000	12.569832000
H	8.448085000	2.407358000	14.121990000
C	10.744791000	7.631718000	3.906486000
H	11.058719000	8.166967000	2.985124000
H	10.887394000	6.546340000	3.701064000
C	7.717848000	10.475728000	8.436430000
H	7.515461000	9.962484000	9.398452000
H	7.573240000	9.740952000	7.616899000
H	6.953020000	11.270447000	8.314765000
C	13.763386000	13.138002000	10.075113000
H	14.789145000	12.761801000	10.272242000
H	13.201055000	13.087922000	11.026016000
H	13.856588000	14.212068000	9.803762000
C	16.248632000	7.027686000	6.674396000
H	16.889446000	7.434485000	5.863604000
H	16.687468000	7.386177000	7.632549000
C	8.771194000	13.954234000	10.885429000
H	8.146519000	14.853228000	11.000069000
C	9.262412000	7.898253000	4.204290000
H	8.627907000	7.567415000	3.355020000
H	9.104632000	8.996307000	4.305258000
C	16.259622000	5.491339000	6.649014000
H	15.929383000	5.143469000	5.643668000
H	17.293142000	5.108808000	6.786504000
C	8.828860000	7.212935000	5.507951000
H	8.873762000	6.107069000	5.379725000
H	7.771067000	7.456353000	5.745274000
C	12.087163000	13.279443000	13.622542000
H	12.508791000	14.060997000	12.958172000
H	12.893984000	12.936147000	14.302170000
H	11.313504000	13.760879000	14.256754000
C	14.105998000	7.680235000	15.076186000

H	14.997795000	7.602035000	15.732992000
H	13.605733000	8.647420000	15.293435000
H	14.465200000	7.704981000	14.028419000
C	9.468316000	11.734077000	7.075959000
H	8.791200000	12.592899000	6.886988000
H	9.353726000	11.024712000	6.228770000
H	10.507046000	12.121161000	7.061937000
C	12.613347000	6.480357000	16.744527000
H	11.949088000	5.610488000	16.923088000
H	12.038843000	7.403681000	16.965833000
H	13.440909000	6.420736000	17.482123000
H	8.879819000	9.385566000	11.589284000
H	14.538020000	6.538813000	12.054671000

TS1: G= -5735.641486 (i = -192 cm-1)

Sn	10.753013000	8.630329000	11.097919000
Sn	12.890282000	6.586497000	11.673464000
Ni	11.356424000	6.791661000	9.670989000
P	10.126753000	5.018997000	9.950435000
P	12.086338000	7.768484000	7.863830000
Si	12.794503000	10.577523000	9.344996000
Si	10.521877000	4.465753000	13.241148000
N	12.154740000	5.033062000	13.009366000
N	11.432238000	10.519763000	10.470246000
C	8.483069000	5.184279000	9.034559000
H	8.780141000	5.269909000	7.966197000
C	13.265404000	4.584194000	13.773190000
C	10.936962000	3.469744000	9.270214000
H	12.004196000	3.620763000	9.549307000
C	10.859974000	3.484387000	7.732383000
H	9.803913000	3.339373000	7.415062000
H	11.166080000	4.482473000	7.354944000
C	14.022558000	3.449957000	13.334188000
C	9.452802000	11.845397000	9.796706000
C	14.341262000	9.765440000	10.120850000
H	14.073988000	8.680837000	10.115373000
C	13.816442000	7.050255000	7.688718000
H	14.323000000	7.466447000	8.587637000
C	13.690053000	5.281741000	14.949659000
C	9.529279000	5.544309000	14.488802000
H	10.115052000	5.502569000	15.432029000
C	9.493868000	4.574056000	11.630704000
H	8.905805000	3.637437000	11.549244000
H	8.744923000	5.369747000	11.832735000
C	10.542606000	11.606301000	10.685759000
C	12.389573000	9.590930000	7.751002000
H	11.461657000	10.045821000	7.349665000
H	13.171047000	9.781028000	6.986403000
C	13.640069000	2.694200000	12.068214000
H	12.588826000	2.966488000	11.846076000
C	9.473756000	7.009254000	14.034059000
H	8.867235000	7.125484000	13.110207000
H	10.483998000	7.416990000	13.823077000
H	9.004873000	7.664909000	14.799896000
C	13.797698000	5.522421000	7.863812000
H	13.206103000	5.265682000	8.770009000
H	13.272406000	5.055480000	7.001695000
C	7.482003000	4.024285000	9.153949000

H	7.944127000	3.073982000	8.817520000
H	7.205260000	3.880137000	10.221101000
C	11.188091000	7.400026000	6.251270000
H	11.389219000	6.315036000	6.087718000
C	7.827223000	6.520741000	9.436367000
H	7.586814000	6.502661000	10.522916000
H	8.563103000	7.343169000	9.298981000
C	9.155978000	10.915467000	8.627372000
H	9.888605000	10.085134000	8.683893000
C	10.570463000	2.651768000	13.884022000
H	11.396517000	2.181702000	13.308909000
C	10.513796000	2.107382000	9.839245000
H	10.641864000	2.095287000	10.940001000
H	9.434230000	1.930435000	9.644082000
C	12.926125000	6.487541000	15.478176000
H	12.106211000	6.675418000	14.759851000
C	11.341291000	0.974995000	9.206756000
H	12.400758000	1.089846000	9.524952000
H	11.004029000	-0.007444000	9.599115000
C	15.152788000	3.044755000	14.065653000
H	15.732144000	2.173454000	13.723224000
C	10.718744000	12.445708000	11.827569000
C	15.560961000	3.726709000	15.218766000
H	16.448771000	3.393099000	15.777503000
C	14.827975000	4.838206000	15.647797000
H	15.144283000	5.380528000	16.553071000
C	13.025985000	12.400723000	8.807650000
H	11.980889000	12.785035000	8.764084000
C	11.600346000	8.186790000	4.995223000
H	11.418568000	9.269226000	5.174433000
H	12.681966000	8.083273000	4.786719000
C	14.649559000	7.438443000	6.457839000
H	14.185954000	7.001601000	5.546631000
H	14.661177000	8.538630000	6.310401000
C	13.648140000	12.569918000	7.410372000
H	13.731812000	13.646302000	7.144405000
H	13.044704000	12.083692000	6.617364000
H	14.672075000	12.143824000	7.356350000
C	9.679261000	7.567659000	6.496809000
H	9.398199000	7.006342000	7.406253000
H	9.468956000	8.637226000	6.721917000
C	14.477227000	3.169716000	10.869271000
H	14.311204000	4.250035000	10.680475000
H	14.203590000	2.618063000	9.945332000
H	15.562767000	3.022900000	11.047859000
C	15.574753000	9.947837000	9.218917000
H	15.936090000	10.997134000	9.243431000
H	15.374039000	9.698032000	8.157495000
H	16.416902000	9.305278000	9.555618000
C	11.714650000	2.366727000	7.123123000
H	11.642815000	2.386294000	6.014890000
H	12.784083000	2.548135000	7.374351000
C	15.220211000	4.958248000	7.968821000
H	15.678983000	5.332028000	8.911198000
H	15.185381000	3.852805000	8.056002000
C	8.116432000	5.013257000	14.783783000
H	7.565264000	5.695491000	15.468228000
H	8.133019000	4.013502000	15.261166000
H	7.504828000	4.926534000	13.859192000
C	6.209563000	4.304109000	8.336111000

H	6.478106000	4.352701000	7.256250000
H	5.493999000	3.461486000	8.443960000
C	14.650485000	10.132223000	11.579669000
H	15.526659000	9.555888000	11.948717000
H	13.802141000	9.893335000	12.249211000
H	14.890117000	11.208672000	11.696720000
C	11.802271000	12.123427000	12.846546000
H	12.612575000	11.616296000	12.288157000
C	5.553156000	5.627699000	8.752537000
H	5.203472000	5.544508000	9.806629000
H	4.649744000	5.825497000	8.137783000
C	11.285156000	11.114188000	13.889882000
H	10.426720000	11.533610000	14.454328000
H	12.083142000	10.842507000	14.611259000
H	10.938209000	10.172849000	13.417324000
C	11.284808000	0.997701000	7.672330000
H	11.920379000	0.190235000	7.251395000
H	10.244164000	0.783932000	7.337681000
C	8.609128000	12.948193000	10.026714000
H	7.773744000	13.138680000	9.334297000
C	6.543849000	6.795199000	8.642343000
H	6.077865000	7.739769000	8.990747000
H	6.808652000	6.951937000	7.572746000
C	13.722596000	1.169218000	12.239651000
H	14.768579000	0.822842000	12.370521000
H	13.318097000	0.649381000	11.347916000
H	13.146495000	0.830318000	13.124461000
C	9.847817000	13.531727000	12.019298000
H	9.986852000	14.185013000	12.893373000
C	10.966868000	2.599814000	15.371877000
H	10.188925000	3.058312000	16.018416000
H	11.920642000	3.129633000	15.566767000
H	11.095209000	1.549603000	15.714588000
C	9.301213000	1.824997000	13.613296000
H	9.425386000	0.781376000	13.977342000
H	9.057895000	1.762558000	12.532851000
H	8.410328000	2.240908000	14.126579000
C	10.781618000	7.741369000	3.770297000
H	11.084138000	8.329714000	2.878048000
H	11.022117000	6.678644000	3.539713000
C	7.759810000	10.281737000	8.744479000
H	7.651264000	9.746364000	9.708843000
H	7.587611000	9.553622000	7.925450000
H	6.955493000	11.044245000	8.690609000
C	13.775962000	13.236357000	9.858550000
H	14.839589000	12.928873000	9.941602000
H	13.322558000	13.144361000	10.863290000
H	13.768415000	14.314968000	9.589879000
C	16.084580000	6.902164000	6.587100000
H	16.681852000	7.187857000	5.695426000
H	16.572753000	7.385509000	7.462755000
C	8.804780000	13.797105000	11.121259000
H	8.137804000	14.657424000	11.284011000
C	9.271920000	7.877640000	4.016088000
H	8.699219000	7.514862000	3.136532000
H	9.019544000	8.956495000	4.130497000
C	16.089423000	5.377975000	6.774959000
H	15.695363000	4.898127000	5.850103000
H	17.127517000	5.004599000	6.901690000
C	8.845524000	7.129444000	5.287403000

H	8.977906000	6.033603000	5.136531000
H	7.764889000	7.287815000	5.490690000
C	12.397292000	13.356863000	13.540216000
H	12.732783000	14.118801000	12.807803000
H	13.271543000	13.066035000	14.157815000
H	11.668468000	13.844204000	14.221169000
C	13.800803000	7.752360000	15.528156000
H	14.639348000	7.639481000	16.247374000
H	13.200091000	8.629821000	15.846810000
H	14.229159000	7.981110000	14.532526000
C	9.345842000	11.618000000	7.271828000
H	8.618167000	12.445986000	7.143434000
H	9.197689000	10.910500000	6.428449000
H	10.360244000	12.055453000	7.179276000
C	12.302331000	6.198071000	16.855993000
H	11.695361000	5.270904000	16.841603000
H	11.646497000	7.034572000	17.175841000
H	13.083677000	6.067366000	17.634121000
H	9.222349000	9.223302000	11.741203000
H	12.558705000	8.021825000	12.814556000

INT1: G= -5735.657903

Sn	10.758842000	8.759777000	11.468423000
Sn	12.791499000	6.306616000	11.317801000
Ni	11.082972000	6.919063000	9.762409000
P	10.039368000	5.047879000	10.068101000
P	12.180496000	7.712049000	8.010847000
Si	12.745568000	10.567470000	9.446102000
Si	10.527880000	4.340847000	13.240429000
N	12.144284000	4.898825000	12.815666000
N	11.373421000	10.592591000	10.527961000
C	8.415437000	5.287576000	9.139242000
H	8.720461000	5.376051000	8.074088000
C	13.284412000	4.482827000	13.564995000
C	10.842669000	3.517667000	9.328286000
H	11.907212000	3.638170000	9.630798000
C	10.793513000	3.593100000	7.793246000
H	9.739772000	3.485371000	7.454222000
H	11.130540000	4.595923000	7.460243000
C	14.025060000	3.325319000	13.171704000
C	9.498700000	11.933137000	9.625232000
C	14.247302000	9.777524000	10.337089000
H	13.995196000	8.689085000	10.350997000
C	13.911475000	6.989472000	7.828820000
H	14.373855000	7.271693000	8.803722000
C	13.726704000	5.247376000	14.688450000
C	9.654979000	5.366456000	14.612412000
H	10.334589000	5.330731000	15.492738000
C	9.399695000	4.510744000	11.714168000
H	8.826281000	3.570483000	11.591360000
H	8.654430000	5.291136000	11.975819000
C	10.481900000	11.689899000	10.634122000
C	12.458053000	9.528741000	7.857413000
H	11.538675000	9.945081000	7.400747000
H	13.269899000	9.724266000	7.128112000
C	13.637179000	2.527878000	11.933403000
H	12.588964000	2.802837000	11.699513000
C	9.457605000	6.835357000	14.218835000

H	8.785546000	6.943971000	13.340466000
H	10.408605000	7.340922000	13.964003000
H	8.994291000	7.419808000	15.042885000
C	13.884731000	5.452996000	7.769085000
H	13.226632000	5.056928000	8.569934000
H	13.434238000	5.129364000	6.804344000
C	7.381653000	4.154669000	9.236739000
H	7.817835000	3.197918000	8.884624000
H	7.095957000	4.000960000	10.299872000
C	11.268507000	7.305400000	6.414505000
H	11.405619000	6.206343000	6.295315000
C	7.806237000	6.639103000	9.564170000
H	7.573264000	6.615263000	10.651778000
H	8.562710000	7.446011000	9.445309000
C	9.257775000	10.929691000	8.507211000
H	9.979492000	10.103880000	8.669186000
C	10.646193000	2.508780000	13.817664000
H	11.430704000	2.075690000	13.162448000
C	10.383375000	2.142152000	9.835029000
H	10.490899000	2.081743000	10.935912000
H	9.303790000	1.996897000	9.614621000
C	13.011283000	6.529850000	15.082113000
H	12.060393000	6.537210000	14.520577000
C	11.196946000	1.016958000	9.173312000
H	12.252227000	1.094642000	9.514857000
H	10.828998000	0.028732000	9.520742000
C	15.154891000	2.947200000	13.918864000
H	15.725268000	2.054506000	13.619421000
C	10.537602000	12.567981000	11.763846000
C	15.573332000	3.683198000	15.034573000
H	16.458324000	3.366857000	15.607468000
C	14.861818000	4.830166000	15.405426000
H	15.196954000	5.421551000	16.271732000
C	13.041676000	12.355126000	8.819318000
H	12.006495000	12.732604000	8.651878000
C	11.715293000	8.006399000	5.121522000
H	11.629072000	9.106620000	5.256319000
H	12.780402000	7.802212000	4.900300000
C	14.819051000	7.533475000	6.712260000
H	14.416843000	7.222135000	5.724767000
H	14.837920000	8.641947000	6.708310000
C	13.798081000	12.454887000	7.483651000
H	13.916650000	13.517104000	7.176077000
H	13.270223000	11.934768000	6.658704000
H	14.818949000	12.022935000	7.548502000
C	9.776207000	7.570023000	6.671591000
H	9.485966000	7.049616000	7.603836000
H	9.631835000	8.653289000	6.871948000
C	14.486225000	2.956015000	10.725015000
H	14.373299000	4.043015000	10.530514000
H	14.181972000	2.415007000	9.804723000
H	15.565401000	2.764798000	10.899220000
C	15.550170000	9.938710000	9.537746000
H	15.895364000	10.993644000	9.543251000
H	15.440453000	9.638278000	8.476414000
H	16.370499000	9.326031000	9.971465000
C	11.634710000	2.481446000	7.154784000
H	11.582965000	2.547907000	6.047398000
H	12.702815000	2.630165000	7.431125000
C	15.300073000	4.872265000	7.898088000

H	15.686690000	5.114176000	8.913058000
H	15.265450000	3.764473000	7.833834000
C	8.308899000	4.742006000	15.024894000
H	7.795736000	5.374538000	15.781780000
H	8.423633000	3.732016000	15.463741000
H	7.616067000	4.653576000	14.160138000
C	6.121895000	4.490378000	8.420889000
H	6.394641000	4.553776000	7.342945000
H	5.381766000	3.667057000	8.509664000
C	14.419920000	10.214104000	11.798386000
H	15.277805000	9.690784000	12.274238000
H	13.517287000	9.991257000	12.399425000
H	14.607261000	11.303799000	11.882882000
C	11.400053000	12.235781000	12.973816000
H	12.079800000	11.421246000	12.657427000
C	5.506248000	5.824260000	8.866116000
H	5.155954000	5.729713000	9.919017000
H	4.608063000	6.061450000	8.257855000
C	10.506554000	11.698212000	14.110900000
H	9.835032000	12.496380000	14.492853000
H	11.121308000	11.329301000	14.958754000
H	9.872190000	10.860841000	13.762176000
C	11.167484000	1.101072000	7.640507000
H	11.793715000	0.297878000	7.198173000
H	10.128655000	0.922388000	7.280752000
C	8.694175000	13.084600000	9.696416000
H	7.950925000	13.272948000	8.904678000
C	6.530943000	6.963296000	8.776477000
H	6.094656000	7.915740000	9.139928000
H	6.801369000	7.129470000	7.709940000
C	13.705073000	1.008143000	12.149881000
H	14.747372000	0.658432000	12.298292000
H	13.303201000	0.467639000	11.269066000
H	13.119402000	0.698441000	13.038843000
C	9.711272000	13.704995000	11.795171000
H	9.770914000	14.386204000	12.658864000
C	11.161264000	2.408613000	15.266823000
H	10.417038000	2.797984000	15.992775000
H	12.102637000	2.973769000	15.415908000
H	11.362328000	1.350807000	15.544516000
C	9.374592000	1.666646000	13.611725000
H	9.546792000	0.612938000	13.922706000
H	9.051422000	1.640966000	12.550862000
H	8.517740000	2.043176000	14.205723000
C	10.841922000	7.576540000	3.928903000
H	11.168384000	8.109064000	3.010544000
H	11.001564000	6.491538000	3.734357000
C	7.850722000	10.317502000	8.612087000
H	7.707035000	9.847332000	9.605256000
H	7.698193000	9.539512000	7.835865000
H	7.058871000	11.083943000	8.480474000
C	13.677492000	13.250200000	9.893931000
H	14.721680000	12.946497000	10.118715000
H	13.104930000	13.215000000	10.839581000
H	13.709478000	14.312243000	9.566194000
C	16.246256000	6.982150000	6.861742000
H	16.897139000	7.386597000	6.058040000
H	16.671698000	7.344462000	7.824280000
C	8.809333000	13.985732000	10.761005000
H	8.180864000	14.888735000	10.798954000

C	9.350575000	7.828605000	4.193511000
H	8.737914000	7.483922000	3.333970000
H	9.178349000	8.925252000	4.281984000
C	16.253571000	5.446929000	6.841691000
H	15.936775000	5.095125000	5.833415000
H	17.282727000	5.060008000	6.997443000
C	8.894788000	7.147501000	5.492094000
H	8.952308000	6.041227000	5.372653000
H	7.830860000	7.384337000	5.706274000
C	12.255063000	13.408183000	13.481245000
H	12.903168000	13.824262000	12.685187000
H	12.910637000	13.077015000	14.313215000
H	11.629035000	14.238912000	13.869984000
C	13.809620000	7.763246000	14.625316000
H	14.805042000	7.805212000	15.115007000
H	13.263058000	8.701758000	14.850113000
H	13.986759000	7.739561000	13.528259000
C	9.507718000	11.532886000	7.114481000
H	8.793232000	12.353817000	6.897643000
H	9.384756000	10.770439000	6.315693000
H	10.529248000	11.955404000	7.031417000
C	12.673305000	6.599291000	16.579008000
H	12.132343000	5.690421000	16.912984000
H	12.031162000	7.478947000	16.790065000
H	13.583119000	6.696312000	17.207925000
H	9.050247000	9.129048000	11.647450000
H	11.341169000	9.057601000	13.097647000

TS2: G= -5735.627109 (i = -1001 cm-1)

Sn	10.320207000	8.780912000	11.204606000
Sn	12.772769000	6.372622000	11.219459000
Ni	11.000847000	6.896903000	9.579492000
P	9.864144000	5.114365000	10.092017000
P	12.332136000	7.733586000	8.039959000
Si	12.765829000	10.587836000	9.481458000
Si	10.499870000	4.411295000	13.227184000
N	12.098911000	4.931007000	12.740069000
N	11.334608000	10.571234000	10.477958000
C	8.226654000	5.290346000	9.154132000
H	8.527994000	5.296564000	8.083771000
C	13.271441000	4.531540000	13.444238000
C	10.687518000	3.569061000	9.426261000
H	11.707985000	3.666772000	9.861151000
C	10.825677000	3.697076000	7.900627000
H	9.818027000	3.650427000	7.429595000
H	11.237587000	4.700519000	7.664483000
C	14.013170000	3.378205000	13.031159000
C	9.486819000	11.924116000	9.524966000
C	14.241366000	9.814331000	10.420414000
H	13.989630000	8.724927000	10.445664000
C	14.062458000	7.024895000	7.863194000
H	14.525947000	7.289768000	8.842479000
C	13.767704000	5.307069000	14.541003000
C	9.726858000	5.379846000	14.702793000
H	10.479126000	5.308191000	15.518731000
C	9.283854000	4.719990000	11.786156000
H	8.584673000	3.860577000	11.739895000
H	8.674166000	5.601403000	12.077202000

C	10.438307000	11.668291000	10.564673000
C	12.561345000	9.550955000	7.879745000
H	11.642996000	9.935253000	7.394155000
H	13.388877000	9.762308000	7.173795000
C	13.575103000	2.541402000	11.837881000
H	12.530199000	2.838966000	11.617282000
C	9.503713000	6.864409000	14.385987000
H	8.709922000	7.008089000	13.620383000
H	10.410461000	7.368109000	13.999382000
H	9.176052000	7.428765000	15.285713000
C	14.037538000	5.489352000	7.777652000
H	13.391056000	5.077911000	8.580422000
H	13.582390000	5.178852000	6.810670000
C	7.197720000	4.163499000	9.344415000
H	7.629676000	3.187516000	9.048115000
H	6.932110000	4.079630000	10.420765000
C	11.421032000	7.271334000	6.463079000
H	11.663837000	6.194953000	6.313962000
C	7.606412000	6.665798000	9.472991000
H	7.393667000	6.728745000	10.563758000
H	8.348613000	7.464184000	9.271287000
C	9.300810000	10.944228000	8.374187000
H	10.021322000	10.120357000	8.551671000
C	10.547283000	2.538774000	13.675327000
H	11.222597000	2.109527000	12.904693000
C	10.147051000	2.191924000	9.833154000
H	10.119025000	2.098576000	10.935976000
H	9.102438000	2.068014000	9.477435000
C	13.060483000	6.576492000	14.986324000
H	12.116774000	6.616756000	14.413897000
C	11.015833000	1.071323000	9.235828000
H	12.024504000	1.117069000	9.699671000
H	10.594257000	0.080654000	9.507276000
C	15.188387000	3.025541000	13.717697000
H	15.753724000	2.137969000	13.393510000
C	10.435742000	12.516768000	11.719089000
C	15.657492000	3.776299000	14.802402000
H	16.577885000	3.479875000	15.328319000
C	14.945105000	4.912204000	15.200971000
H	15.313524000	5.516386000	16.045042000
C	13.062765000	12.383222000	8.869760000
H	12.029960000	12.747618000	8.662846000
C	11.743486000	8.035661000	5.171413000
H	11.554613000	9.119492000	5.331261000
H	12.814979000	7.940584000	4.905831000
C	14.954667000	7.594789000	6.747465000
H	14.535739000	7.310147000	5.757861000
H	14.977263000	8.703097000	6.772619000
C	13.868213000	12.494046000	7.564090000
H	13.985899000	13.558008000	7.262042000
H	13.378688000	11.968706000	6.719011000
H	14.890880000	12.073988000	7.667722000
C	9.923116000	7.367534000	6.793208000
H	9.749121000	6.782904000	7.727135000
H	9.665091000	8.421412000	7.032180000
C	14.411503000	2.891403000	10.595951000
H	14.331314000	3.973919000	10.370195000
H	14.067167000	2.328319000	9.702975000
H	15.486003000	2.662825000	10.754002000
C	15.566405000	9.966951000	9.655917000

H	15.911576000	11.021971000	9.660964000
H	15.487420000	9.656316000	8.594679000
H	16.373506000	9.358290000	10.118635000
C	11.703753000	2.582568000	7.321756000
H	11.772569000	2.681540000	6.217630000
H	12.738291000	2.699856000	7.714529000
C	15.456105000	4.912810000	7.886175000
H	15.850203000	5.140813000	8.901433000
H	15.423693000	3.806070000	7.806658000
C	8.417370000	4.751109000	15.212616000
H	7.974286000	5.367099000	16.025796000
H	8.563455000	3.731003000	15.617039000
H	7.652189000	4.685182000	14.408869000
C	5.918059000	4.430644000	8.532938000
H	6.166402000	4.412564000	7.447465000
H	5.188561000	3.610350000	8.701194000
C	14.371337000	10.274743000	11.878217000
H	15.215683000	9.761822000	12.387587000
H	13.451401000	10.056711000	12.453157000
H	14.555605000	11.365986000	11.950547000
C	11.255628000	12.159083000	12.951361000
H	11.947662000	11.353731000	12.637353000
C	5.297925000	5.789431000	8.885256000
H	4.968877000	5.776305000	9.949075000
H	4.386464000	5.971571000	8.277757000
C	10.329110000	11.589342000	14.045802000
H	9.617521000	12.363568000	14.403588000
H	10.917766000	11.234185000	14.917665000
H	9.738939000	10.732610000	13.665623000
C	11.161170000	1.199823000	7.712430000
H	11.819705000	0.396954000	7.319036000
H	10.165878000	1.051111000	7.234723000
C	8.666930000	13.065158000	9.592768000
H	7.952010000	13.265197000	8.778059000
C	6.315233000	6.919989000	8.685112000
H	5.883893000	7.899568000	8.977664000
H	6.563831000	6.999104000	7.602829000
C	13.603520000	1.032372000	12.128593000
H	14.638096000	0.661477000	12.279662000
H	13.173683000	0.457986000	11.282570000
H	13.024165000	0.784595000	13.040500000
C	9.595908000	13.643816000	11.745158000
H	9.612242000	14.303153000	12.627572000
C	11.214821000	2.309895000	15.045821000
H	10.590156000	2.705493000	15.874301000
H	12.206584000	2.798296000	15.112803000
H	11.362633000	1.225082000	15.242182000
C	9.212771000	1.779059000	13.572381000
H	9.367513000	0.690905000	13.743056000
H	8.734389000	1.882370000	12.576263000
H	8.474210000	2.120905000	14.324301000
C	10.863894000	7.536752000	4.010330000
H	11.085604000	8.117998000	3.090466000
H	11.127909000	6.478958000	3.783407000
C	7.893593000	10.321771000	8.399688000
H	7.690523000	9.854052000	9.384559000
H	7.789159000	9.536833000	7.621380000
H	7.104414000	11.081137000	8.220774000
C	13.645563000	13.287507000	9.966287000
H	14.685723000	12.999741000	10.227531000

H	13.041177000	13.242343000	10.891634000
H	13.672507000	14.350248000	9.640231000
C	16.384046000	7.040675000	6.866712000
H	17.022408000	7.457605000	6.059304000
H	16.822123000	7.391303000	7.827895000
C	8.732321000	13.940865000	10.682628000
H	8.092925000	14.836234000	10.717895000
C	9.366992000	7.620692000	4.347488000
H	8.757027000	7.229121000	3.506388000
H	9.080459000	8.689842000	4.470317000
C	16.395442000	5.505761000	6.826778000
H	16.068489000	5.166370000	5.817428000
H	17.428106000	5.121600000	6.965066000
C	9.041407000	6.867121000	5.646563000
H	9.220243000	5.777524000	5.501328000
H	7.968028000	6.979250000	5.907666000
C	12.086499000	13.323118000	13.514567000
H	12.756797000	13.763864000	12.750718000
H	12.718101000	12.974847000	14.357911000
H	11.443025000	14.140016000	13.904538000
C	13.875411000	7.824912000	14.610030000
H	14.855993000	7.840614000	15.130604000
H	13.329314000	8.754217000	14.873237000
H	14.078055000	7.851222000	13.519936000
C	9.619632000	11.564434000	7.004242000
H	8.913331000	12.383794000	6.756143000
H	9.546476000	10.806671000	6.194933000
H	10.642183000	11.991679000	6.982873000
C	12.712887000	6.570814000	16.483910000
H	12.184481000	5.640460000	16.775001000
H	12.058612000	7.431116000	16.735037000
H	13.620970000	6.648506000	17.118139000
H	11.231044000	8.824719000	12.832548000
H	11.968890000	8.029601000	12.227321000

5: G= -5734.496406

181
symmetry c1

Sn	10.335565000	8.588438000	11.002248000
Sn	12.863969000	6.560345000	11.430859000
Ni	11.224585000	6.786179000	9.614923000
P	10.024489000	5.028905000	9.984872000
P	12.132104000	7.768940000	7.903036000
Si	12.742088000	10.522099000	9.442081000
Si	10.519524000	4.569267000	13.233116000
N	12.157825000	5.105055000	12.874007000
N	11.318368000	10.433141000	10.468161000
C	8.391768000	5.149638000	9.033178000
H	8.714787000	5.250753000	7.973746000
C	13.244606000	4.627768000	13.664441000
C	10.872138000	3.487727000	9.335018000
H	11.931079000	3.647717000	9.637951000
C	10.832171000	3.509153000	7.794853000
H	9.783427000	3.373522000	7.451455000
H	11.154313000	4.508118000	7.433238000
C	13.954950000	3.447417000	13.287562000
C	9.437310000	11.861356000	9.691839000
C	14.266369000	9.702773000	10.254905000

H	14.043479000	8.611994000	10.182954000
C	13.856040000	7.038218000	7.736371000
H	14.335359000	7.388528000	8.679385000
C	13.659329000	5.346483000	14.828228000
C	9.624343000	5.669229000	14.535715000
H	10.255366000	5.606000000	15.449561000
C	9.404873000	4.660531000	11.681609000
H	8.766852000	3.753331000	11.662080000
H	8.713007000	5.507505000	11.880025000
C	10.450791000	11.543856000	10.647161000
C	12.430800000	9.583843000	7.798140000
H	11.517055000	10.039014000	7.366500000
H	13.244384000	9.783699000	7.071899000
C	13.585298000	2.675836000	12.027854000
H	12.545310000	2.966690000	11.774545000
C	9.539369000	7.141541000	14.115381000
H	8.858888000	7.281673000	13.247718000
H	10.526930000	7.553949000	13.819859000
H	9.144602000	7.783561000	14.932455000
C	13.831769000	5.501418000	7.797717000
H	13.206068000	5.177874000	8.656301000
H	13.343791000	5.099032000	6.882165000
C	7.445099000	3.941024000	9.126303000
H	7.957165000	3.015317000	8.795366000
H	7.154202000	3.778734000	10.187368000
C	11.208756000	7.388352000	6.307187000
H	11.381605000	6.296963000	6.158236000
C	7.657670000	6.447533000	9.414138000
H	7.382357000	6.417052000	10.492120000
H	8.342652000	7.314920000	9.308265000
C	9.182886000	10.978030000	8.476948000
H	9.892745000	10.130154000	8.555395000
C	10.585911000	2.768820000	13.913475000
H	11.412807000	2.297063000	13.342579000
C	10.443343000	2.123767000	9.896894000
H	10.569332000	2.107760000	10.997847000
H	9.364299000	1.950619000	9.700630000
C	12.963159000	6.637055000	15.230705000
H	12.030092000	6.675487000	14.642154000
C	11.267780000	0.989065000	9.263764000
H	12.324717000	1.091921000	9.591850000
H	10.917222000	0.007663000	9.646776000
C	15.039279000	3.017074000	14.073393000
H	15.588988000	2.109342000	13.781439000
C	10.561427000	12.335271000	11.833238000
C	15.439271000	3.717622000	15.217807000
H	16.289557000	3.360842000	15.818813000
C	14.748432000	4.878317000	15.583583000
H	15.062551000	5.439499000	16.477475000
C	13.014182000	12.358640000	8.955111000
H	11.973325000	12.737081000	8.832075000
C	11.629667000	8.145291000	5.036760000
H	11.497812000	9.236142000	5.208034000
H	12.702707000	7.991802000	4.812131000
C	14.734414000	7.509239000	6.565879000
H	14.306297000	7.136316000	5.610242000
H	14.751474000	8.616227000	6.494206000
C	13.748397000	12.567963000	7.619236000
H	13.831650000	13.650930000	7.379949000
H	13.224071000	12.085864000	6.769267000

H	14.782040000	12.163940000	7.643469000
C	9.708446000	7.596926000	6.574866000
H	9.429341000	7.042866000	7.491776000
H	9.531499000	8.670733000	6.803509000
C	14.463464000	3.111136000	10.842964000
H	14.359435000	4.201477000	10.659445000
H	14.178473000	2.575814000	9.912906000
H	15.536760000	2.910950000	11.041932000
C	15.550005000	9.955942000	9.446313000
H	15.878793000	11.012795000	9.527815000
H	15.423969000	9.736292000	8.366457000
H	16.386529000	9.323822000	9.815815000
C	11.690371000	2.388140000	7.198688000
H	11.643545000	2.416990000	6.089464000
H	12.755725000	2.556046000	7.475261000
C	15.251458000	4.933943000	7.926173000
H	15.663736000	5.248593000	8.910597000
H	15.218376000	3.824684000	7.941059000
C	8.225026000	5.135003000	14.891938000
H	7.717053000	5.803061000	15.621515000
H	8.260488000	4.123528000	15.341771000
H	7.566184000	5.077393000	13.998654000
C	6.178651000	4.158882000	8.279156000
H	6.470298000	4.211901000	7.205542000
H	5.503160000	3.282640000	8.377091000
C	14.459146000	9.999275000	11.745782000
H	15.323406000	9.432722000	12.155290000
H	13.564378000	9.707219000	12.330233000
H	14.643906000	11.075892000	11.937242000
C	11.536258000	11.914312000	12.923285000
H	12.372759000	11.405702000	12.406981000
C	5.449496000	5.452252000	8.667405000
H	5.074348000	5.359971000	9.711877000
H	4.556052000	5.602699000	8.025335000
C	10.882687000	10.867866000	13.844389000
H	10.017581000	11.300582000	14.388163000
H	11.604761000	10.471370000	14.588756000
H	10.492035000	10.004983000	13.260520000
C	11.231524000	1.021559000	7.729252000
H	11.861713000	0.207627000	7.312910000
H	10.193157000	0.825658000	7.377055000
C	8.624693000	12.991948000	9.898882000
H	7.852327000	13.240685000	9.153367000
C	6.390846000	6.660728000	8.576695000
H	5.877030000	7.589165000	8.902037000
H	6.684223000	6.820960000	7.514979000
C	13.631537000	1.153106000	12.228540000
H	14.666319000	0.790741000	12.397157000
H	13.246654000	0.624510000	11.334009000
H	13.022414000	0.839625000	13.100547000
C	9.725649000	13.451868000	12.000192000
H	9.821634000	14.065602000	12.908364000
C	10.994086000	2.740821000	15.399304000
H	10.208267000	3.179976000	16.049108000
H	11.933623000	3.298643000	15.583481000
H	11.155278000	1.696785000	15.746155000
C	9.322641000	1.926025000	13.663647000
H	9.454272000	0.894352000	14.057571000
H	9.083939000	1.830846000	12.584782000
H	8.426923000	2.349033000	14.163005000

C	10.773814000	7.721137000	3.829767000
H	11.081766000	8.292420000	2.928382000
H	10.972032000	6.648948000	3.602591000
C	7.767324000	10.375435000	8.508356000
H	7.610632000	9.808797000	9.448892000
H	7.609635000	9.680846000	7.656667000
H	6.985318000	11.160637000	8.450557000
C	13.660507000	13.178287000	10.084715000
H	14.710383000	12.867501000	10.269894000
H	13.106558000	13.076065000	11.037048000
H	13.681532000	14.260691000	9.831259000
C	16.164802000	6.966196000	6.715923000
H	16.795464000	7.312596000	5.870027000
H	16.614351000	7.391926000	7.640785000
C	8.770013000	13.796621000	11.034010000
H	8.129844000	14.680743000	11.175759000
C	9.274660000	7.912649000	4.100731000
H	8.674305000	7.569688000	3.231801000
H	9.063534000	8.999911000	4.218083000
C	16.170309000	5.433013000	6.802470000
H	15.821457000	5.013542000	5.831230000
H	17.204006000	5.055712000	6.951498000
C	8.843108000	7.181551000	5.380284000
H	8.936054000	6.081414000	5.230636000
H	7.771757000	7.377224000	5.599175000
C	12.111602000	13.075272000	13.744872000
H	12.554555000	13.857245000	13.095149000
H	12.904741000	12.708767000	14.428581000
H	11.339341000	13.562278000	14.376640000
C	13.800628000	7.858731000	14.818182000
H	14.781624000	7.872258000	15.337373000
H	13.271418000	8.807606000	15.046258000
H	14.011896000	7.839475000	13.727194000
C	9.461884000	11.707279000	7.151887000
H	8.760648000	12.554322000	7.002224000
H	9.343866000	11.021360000	6.285848000
H	10.489838000	12.121593000	7.124349000
C	12.582709000	6.685140000	16.717910000
H	12.007652000	5.784866000	17.016062000
H	11.958707000	7.577732000	16.930882000
H	13.475297000	6.743516000	17.375533000

6: G= -5691.708336

Pb	10.963563000	6.626496000	2.890209000
Pb	11.688138000	4.187098000	4.855857000
Ni	9.341310000	5.282463000	4.431374000
P	8.591993000	3.920004000	2.927184000
P	9.312651000	6.606391000	6.145154000
Si	11.211303000	2.363935000	1.711390000
Si	11.961386000	8.511367000	5.885751000
N	11.867826000	2.485317000	3.329603000
N	11.703236000	8.394266000	4.158365000
C	12.096931000	9.440381000	3.282458000
C	12.792023000	1.525425000	3.822490000
C	14.199192000	1.673709000	3.593564000
C	7.121648000	4.742605000	2.088338000
H	6.741588000	4.026525000	1.325370000
C	7.960926000	2.243929000	3.477972000

H	8.884228000	1.623700000	3.444772000
C	10.905656000	7.218311000	6.832824000
H	11.544093000	6.317712000	6.953191000
H	10.740294000	7.610175000	7.859729000
C	9.672746000	3.490862000	1.499051000
H	10.043602000	4.459901000	1.103479000
H	9.053609000	3.052823000	0.686480000
C	7.552994000	6.032395000	1.369536000
H	8.045751000	6.705588000	2.108632000
H	8.323943000	5.815150000	0.602270000
C	14.757242000	2.930531000	2.944438000
H	13.898579000	3.455642000	2.492012000
C	6.019764000	5.037177000	3.120226000
H	6.485333000	5.622768000	3.942194000
H	5.660237000	4.096398000	3.583981000
C	8.272911000	8.164493000	6.064313000
H	8.991243000	8.913106000	5.662566000
C	9.698843000	10.376466000	3.266348000
H	9.634932000	9.611840000	4.066469000
C	12.411865000	2.986155000	0.339871000
H	13.349644000	2.414986000	0.516543000
C	7.467387000	2.244470000	4.932438000
H	8.264461000	2.651136000	5.588733000
H	6.604657000	2.937047000	5.034739000
C	5.282403000	7.074219000	1.780319000
H	5.692060000	7.795077000	2.520908000
H	4.412264000	7.574917000	1.306018000
C	11.164080000	10.441425000	2.862598000
C	11.937096000	2.685150000	-1.092310000
H	10.946521000	3.143668000	-1.301505000
H	11.848533000	1.598993000	-1.289457000
H	12.646354000	3.098244000	-1.843025000
C	12.727628000	4.481816000	0.490481000
H	13.614551000	4.784271000	-0.106379000
H	12.931145000	4.767051000	1.544001000
H	11.881187000	5.112444000	0.142275000
C	13.429806000	9.492800000	2.757458000
C	14.409986000	8.360285000	3.016276000
H	13.976502000	7.760915000	3.835111000
C	11.511026000	10.293758000	6.451687000
H	10.603589000	10.528950000	5.852858000
C	8.599507000	5.621562000	7.582030000
H	8.578798000	6.297604000	8.466125000
C	12.346512000	0.410598000	4.601553000
C	8.838448000	9.875737000	2.089751000
H	8.898641000	10.571567000	1.227029000
H	7.770405000	9.780094000	2.377427000
H	9.193067000	8.883001000	1.741060000
C	10.887978000	0.267502000	5.006911000
H	10.323388000	0.974035000	4.366623000
C	6.364021000	6.766423000	0.736800000
H	6.717709000	7.701318000	0.253533000
H	5.931130000	6.135820000	-0.072755000
C	7.048137000	0.842012000	5.392901000
H	7.952424000	0.201009000	5.453250000
H	6.633511000	0.889209000	6.422043000
C	7.122502000	8.045361000	5.052590000
H	7.539212000	7.761387000	4.064221000
H	6.446539000	7.214920000	5.349817000
C	10.749005000	0.526310000	1.365163000

H	10.302727000	0.183531000	2.324645000
C	12.005448000	-0.329670000	1.108978000
H	11.743746000	-1.408765000	1.044955000
H	12.763765000	-0.218532000	1.907840000
H	12.489248000	-0.058052000	0.147131000
C	4.834810000	5.799950000	2.509985000
H	4.308262000	5.133190000	1.789774000
H	4.096901000	6.044444000	3.303615000
C	9.497751000	4.416660000	7.910044000
H	9.606528000	3.798298000	6.989082000
H	10.523717000	4.747286000	8.171130000
C	9.697483000	0.292546000	0.267500000
H	10.061435000	0.602473000	-0.732629000
H	8.754658000	0.842579000	0.457011000
H	9.434403000	-0.785707000	0.196196000
C	6.042242000	0.191900000	4.433033000
H	5.085022000	0.761002000	4.458421000
H	5.802553000	-0.839265000	4.767701000
C	7.171103000	5.156340000	7.250330000
H	6.508331000	6.029347000	7.082706000
H	7.215545000	4.618409000	6.278216000
C	6.928557000	1.605011000	2.535318000
H	6.000157000	2.216474000	2.525871000
H	7.307772000	1.593913000	1.493412000
C	8.915095000	3.546782000	9.030455000
H	9.581067000	2.676844000	9.209938000
H	8.895194000	4.131669000	9.978027000
C	13.759447000	8.099474000	6.440982000
H	14.400453000	8.775513000	5.833864000
C	13.276306000	-0.542168000	5.053624000
H	12.918707000	-1.398508000	5.646215000
C	11.135316000	10.457749000	7.933499000
H	11.985734000	10.230565000	8.607262000
H	10.295240000	9.801226000	8.235498000
H	10.822192000	11.503131000	8.148355000
C	7.779256000	8.675100000	7.427939000
H	7.088516000	7.931731000	7.881236000
H	8.629659000	8.784668000	8.131065000
C	9.182094000	11.716397000	3.815143000
H	9.809756000	12.078299000	4.654155000
H	8.139252000	11.627066000	4.182679000
H	9.182373000	12.506052000	3.036054000
C	15.091278000	0.691088000	4.057693000
H	16.168807000	0.812860000	3.864579000
C	14.145541000	6.650279000	6.109505000
H	15.240798000	6.482404000	6.192137000
H	13.846777000	6.356857000	5.081329000
H	13.662326000	5.930985000	6.805544000
C	14.488441000	7.441461000	1.784013000
H	13.477815000	7.085393000	1.493310000
H	15.122604000	6.550857000	1.978112000
H	14.903273000	7.978873000	0.905651000
C	11.589902000	11.481195000	2.017048000
H	10.863981000	12.248886000	1.706363000
C	6.314043000	9.345045000	4.942063000
H	6.949657000	10.120464000	4.466138000
H	5.448421000	9.194657000	4.263052000
C	15.336776000	3.866674000	4.021531000
H	14.586211000	4.071711000	4.814179000
H	15.649868000	4.839375000	3.587498000

H	16.215819000	3.410572000	4.523199000
C	13.813324000	10.556280000	1.922193000
H	14.844450000	10.589463000	1.536227000
C	15.785770000	2.656807000	1.837908000
H	16.715189000	2.201673000	2.239629000
H	16.074531000	3.601373000	1.331851000
H	15.382670000	1.965381000	1.070369000
C	12.603882000	11.302640000	6.045701000
H	12.278256000	12.345771000	6.253100000
H	12.855019000	11.240613000	4.969180000
H	13.539814000	11.137204000	6.619982000
C	10.695086000	0.721349000	6.467284000
H	11.281409000	0.083784000	7.161529000
H	9.629576000	0.673051000	6.775097000
H	11.046116000	1.766518000	6.600864000
C	14.642140000	-0.425788000	4.771290000
H	15.353072000	-1.189004000	5.122625000
C	12.909924000	11.561896000	1.559298000
H	13.226457000	12.391191000	0.908558000
C	7.495748000	3.075836000	8.687343000
H	7.545470000	2.390500000	7.813674000
H	7.067912000	2.483341000	9.523232000
C	5.848145000	9.860532000	6.310451000
H	5.112872000	9.145933000	6.746159000
H	5.313894000	10.827075000	6.195658000
C	6.578099000	0.182195000	2.993976000
H	7.490345000	-0.453216000	2.937510000
H	5.835998000	-0.269660000	2.302553000
C	7.033406000	10.008665000	7.275487000
H	7.738013000	10.778132000	6.887345000
H	6.688822000	10.368003000	8.267988000
C	15.804758000	8.831100000	3.452552000
H	16.345187000	9.346382000	2.631127000
H	16.428734000	7.968332000	3.765546000
H	15.744845000	9.538595000	4.304398000
C	10.345074000	-1.151351000	4.775613000
H	10.518606000	-1.483812000	3.732331000
H	9.254671000	-1.202252000	4.974235000
H	10.828731000	-1.891627000	5.445663000
C	6.580640000	4.261585000	8.350076000
H	6.428446000	4.874469000	9.267467000
H	5.574427000	3.903543000	8.044522000
C	14.033657000	8.396732000	7.925311000
H	13.348489000	7.829495000	8.591706000
H	13.920596000	9.470970000	8.170021000
H	15.069283000	8.104251000	8.206980000

5. References

[1] P. M. Keil, T. Szilvási, T. J. Hadlington, “Reversible metathesis of ammonia in an acyclic germylene–Ni ^{0} complex” *Chem. Sci.* **2021**, *12*, 5582–5590.

[2] P. M. Keil, T. J. Hadlington, “Protonation of Hydrido-Tetrylenes: H_2 Elimination vs. Tetrylium Cation Formation” *Zeitschrift anorg allge chemie* **2022**, *648*, e202200141.

[3] T. L. Kalkuhl, I. Fernández, T. J. Hadlington, “Cooperative hydrogenation catalysis at a constrained gallylene-nickel(0) interface” *Chem* **2025**, *11*, 102349.

[4] A. Schulz, T. J. Hadlington, “Crystalline Half-Parent Vinyl-tetrylenes: Synthesis and Coordination Chemistry” *Inorg. Chem.* **2025**, *64*, 24898–24909.

[5] J. M. Gilch, M. Iddrisu, P. M. Keil, T. Szilvasi, T. J. Hadlington, “Cooperative Metathesis of H-H/Sn-CAr bonds in stannylene-Ni0 systems” *Chem. Sci.* **2026**, 10.1039.D5SC09496H.

[6] S. J. Bonyhady, C. Jones, S. Nembenna, A. Stasch, A. J. Edwards, G. J. McIntyre, “β-Diketiminate-Stabilized Magnesium(I) Dimers and Magnesium(II) Hydride Complexes: Synthesis, Characterization, Adduct Formation, and Reactivity Studies” *Chemistry A European J* **2010**, *16*, 938–955.

[7] J. T. Patton, S. G. Feng, K. A. Abboud, “Chelating Diamide Group IV Metal Olefin Polymerization Catalysts” *Organometallics* **2001**, *20*, 3399–3405.

[8] A. J. Sicard, R. T. Baker, “Safe and Expeditious Preparation of Ni(cod)2 for Same-Day High-Throughput Screening” *Org. Process Res. Dev.* **2020**, *24*, 2950–2952.

[9] B. M. Lindley, B. P. Jacobs, S. N. MacMillan, P. T. Wolczanski, “Neutral Fe(IV) alkylidenes, including some that bind dinitrogen” *Chem. Commun.* **2016**, *52*, 3891–3894.

[10] G. M. Sheldrick, SHELXL-97, Program for Crystal Strucutre Refinement, Göttingen, 1997.

[11] G. M. Sheldrick, “*SHELXT* – Integrated space-group and crystal-structure determination” *Acta Crystallogr A Found Adv* **2015**, *71*, 3–8.

[12] G. M. Sheldrick, “Crystal structure refinement with *SHELXL*” *Acta Crystallogr C Struct Chem* **2015**, *71*, 3–8.

[13] F. Neese, “Software Update: The ORCA Program System—Version 6.0” *WIREs Comput Mol Sci* **2025**, *15*, e70019.

[14] A. D. Becke, “Density-functional exchange-energy approximation with correct asymptotic behavior” *Phys. Rev. A* **1988**, *38*, 3098–3100.

[15] J. P. Perdew, “Density-functional approximation for the correlation energy of the inhomogeneous electron gas” *Phys. Rev. B* **1986**, *33*, 8822–8824.

[16] S. Grimme, J. Antony, S. Ehrlich, H. Krieg, “A consistent and accurate ab initio parametrization of density functional dispersion correction (DFT-D) for the 94 elements H-Pu” *The Journal of Chemical Physics* **2010**, *132*, 154104.

[17] S. Grimme, S. Ehrlich, L. Goerigk, “Effect of the damping function in dispersion corrected density functional theory” *J Comput Chem* **2011**, *32*, 1456–1465.

[18] F. Neese, “An improvement of the resolution of the identity approximation for the formation of the Coulomb matrix” *J Comput Chem* **2003**, *24*, 1740–1747.

[19] F. Weigend, R. Ahlrichs, “Balanced basis sets of split valence, triple zeta valence and quadruple zeta valence quality for H to Rn: Design and assessment of accuracy” *Phys. Chem. Chem. Phys.* **2005**, *7*, 3297–3305.

[20] E. D. Glendening, C. R. Landis, F. Weinhold, “*NBO 6.0* : Natural bond orbital analysis program” *J Comput Chem* **2013**, *34*, 1429–1437.

[21] R. F. W. Bader, *Atoms in molecules: a quantum theory*, Clarendon Press, Oxford ; New York, **1990**.

[22] F. M. Bickelhaupt, E. J. Baerends in *Reviews in Computational Chemistry* (Eds.: K.B. Lipkowitz, D.B. Boyd), Wiley, **2000**, pp. 1–86.

[23] M. von Hopffgarten, G. Frenking, “Energy decomposition analysis” *WIREs Computational Molecular Science* **2012**, *2*, 43–62.

[24] M. P. Mitoraj, A. Michalak, T. Ziegler, “A Combined Charge and Energy Decomposition Scheme for Bond Analysis” *J. Chem. Theory Comput.* **2009**, *5*, 962–975.

[25] ADF2020, SCM, Theoretical Chemistry, Vrije Universiteit, Amsterdam, The Netherlands, http://www.scm.com.

[26] J. G. Snijders, P. Vernooijs, E. J. Baerends, “Roothaan-Hartree-Fock-Slater atomic wave functions” *Atomic Data and Nuclear Data Tables* **1981**, *26*, 483–509.
[27] J. Krijn, E. J. Baerends, *Fit Functions in the HFS-Method*, Internal Report (in Dutch), Vrije Universiteit Amsterdam, The Netherlands, 1984.
[28] E. van Lenthe, E. J. Baerends, J. G. Snijders, “Relativistic regular two-component Hamiltonians” *The Journal of Chemical Physics* **1993**, *99*, 4597–4610.
[29] E. van Lenthe, E. J. Baerends, J. G. Snijders, “Relativistic total energy using regular approximations” *The Journal of Chemical Physics* **1994**, *101*, 9783–9792.
[30] E. van Lenthe, A. Ehlers, E.-J. Baerends, “Geometry optimizations in the zero order regular approximation for relativistic effects” *The Journal of Chemical Physics* **1999**, *110*, 8943–8953.
